# Trends in guideline implementation: an updated scoping review

**DOI:** 10.1186/s13012-022-01223-6

**Published:** 2022-07-23

**Authors:** Sanne Peters, Krithika Sukumar, Sophie Blanchard, Akilesh Ramasamy, Jennifer Malinowski, Pamela Ginex, Emily Senerth, Marleen Corremans, Zachary Munn, Tamara Kredo, Lucia Prieto Remon, Etienne Ngeh, Lisa Kalman, Samia Alhabib, Yasser Sami Amer, Anna Gagliardi

**Affiliations:** 1 School of Health Sciences, The University of Melbourne, Melbourne, Australia; 2 Department of Public Health and Primary Care, University of Leuven, Leuven, Belgium; 3 University of Toronto, Toronto, Canada; 4 Department of guidelines, Haute Autorité de Santé, Paris, France; 5 Jawaharlal Institute of Postgraduate Medical Education and Research (JIPMER), Karaikal Campus, Puducherry, 609602 India; 6 Write InSciTe, South Salem, NY USA; 7 School of Nursing, Stony Brook University, Stony Brook, NY USA; 8 Society for Cardiovascular Angiography and Interventions, Washington, DC USA; 9 BICEP: A JBI Affiliated Group, Leuven, Belgium; 10 JBI, Faculty of Health and Medical Sciences, The University of Adelaide, Adelaide, SA Australia; 11 Cochrane South Africa, South African Medical Research Council, Cape Town, South Africa; 12 Aragon Health Sciences Institute (Instituto Aragonés de Ciencias de la Salud-IACS), Aragón, Spain; 13 School of Health and Well Being, Sheffield Hallam University, Sheffield, UK; 14 Research Organisation for Health Education and Rehabilitation-Cameroon (ROHER-CAM), Bamenda, Cameroon; 15 Healthcare Programs, Heart Foundation of Australia, Melbourne, Australia; 16 Chair of Arab-GIN, Riyadh, Saudi Arabia; 17 Clinical Practice Guidelines and Quality Research Unit, Quality Management Department, King Saud University Medical City, Riyadh, Saudi Arabia; 18 Research Chair for Evidence-Based Health Care and Knowledge Translation, King Saud University, Riyadh, Saudi Arabia; 19 Center for Evidence-Based Clinical Practice Guidelines, Alexandria University, Alexandria, Egypt; 20 Toronto General Hospital Research Institute, University Health Network, Toronto, ON Canada

**Keywords:** Guidelines, Implementation interventions, Implementation strategies, Quality improvement, Scoping review

## Abstract

**Background:**

Guidelines aim to support evidence-informed practice but are inconsistently used without implementation strategies. Our prior scoping review revealed that guideline implementation interventions were not selected and tailored based on processes known to enhance guideline uptake and impact. The purpose of this study was to update the prior scoping review.

**Methods:**

We searched MEDLINE, EMBASE, AMED, CINAHL, Scopus, and the Cochrane Database of Systematic Reviews for studies published from 2014 to January 2021 that evaluated guideline implementation interventions. We screened studies in triplicate and extracted data in duplicate. We reported study and intervention characteristics and studies that achieved impact with summary statistics.

**Results:**

We included 118 studies that implemented guidelines on 16 clinical topics. With regard to implementation planning, 21% of studies referred to theories or frameworks, 50% pre-identified implementation barriers, and 36% engaged stakeholders in selecting or tailoring interventions. Studies that employed frameworks (*n*=25) most often used the theoretical domains framework (28%) or social cognitive theory (28%). Those that pre-identified barriers (*n*=59) most often consulted literature (60%). Those that engaged stakeholders (*n*=42) most often consulted healthcare professionals (79%). Common interventions included educating professionals about guidelines (44%) and information systems/technology (41%). Most studies employed multi-faceted interventions (75%). A total of 97 (82%) studies achieved impact (improvements in one or more reported outcomes) including 10 (40% of 25) studies that employed frameworks, 28 (47.45% of 59) studies that pre-identified barriers, 22 (52.38% of 42) studies that engaged stakeholders, and 21 (70% of 30) studies that employed single interventions.

**Conclusions:**

Compared to our prior review, this review found that more studies used processes to select and tailor interventions, and a wider array of types of interventions across the Mazza taxonomy. Given that most studies achieved impact, this might reinforce the need for implementation planning. However, even studies that did not plan implementation achieved impact. Similarly, even single interventions achieved impact. Thus, a future systematic review based on this data is warranted to establish if the use of frameworks, barrier identification, stakeholder engagement, and multi-faceted interventions are associated with impact.

**Trial registration:**

The protocol was registered with Open Science Framework (https://osf.io/4nxpr) and published in JBI Evidence Synthesis.

**Supplementary Information:**

The online version contains supplementary material available at 10.1186/s13012-022-01223-6.

Contributions to the literature

- By including guidelines on any clinical topic, this review of 118 studies published between 2014 and 2021 provides a comprehensive picture of implementation planning practices.
- Compared to an earlier version, this updated review found that guidelines are implemented with a broader range of interventions types and that more interventions are selected and tailored based on frameworks, pre-identifying barriers, and stakeholder engagement.
- However, even studies that did not employ these approaches achieved impact, raising important questions about their value that can only be answered through a future systematic review based on this data.

## Background

Clinical practice guidelines include recommendations that are based on the best available evidence and are intended to optimize patient care [1, 2]. Given that guidelines support evidence-informed decision making and reduce practice variations, they are essential for planning, delivering, and improving high-quality health care [3]. However, policy and practice are not consistently informed by evidence-based clinical practice guidelines, which can lead to suboptimal care and inappropriate use of health care resources [4–7].

It is known that guideline implementation is a complex process that is often hindered by a variety of individual-, organisational-, and system-level barriers [8–11]. Common barriers identified across countries include, for example, limited knowledge of and negative attitudes toward existing guidelines, and lack of managers’ support for guideline implementation [12, 13].

Despite many barriers, guideline implementation is increasingly recognized as a process crucial to improving healthcare quality. Existing reviews have focused on specific clinical disciplines such as nursing and occupational therapy [14, 15], medical areas including cancer and venous thromboembolism prevention [16, 17], barriers of guideline implementation [18, 19], or specific topics such as the role of middle managers, nudge strategies, and de-implementation strategies [20–22].

Considerable knowledge is now available on how to optimize guideline implementation and uptake. Research shows that implementation interventions selected and tailored according to pre-identified barriers, theory, and/or stakeholder engagement can optimize guideline implementation and uptake [8, 23–25]. For example, a Cochrane review by Baker et al. of 26 randomized controlled trials (RCTs) revealed that tailoring interventions to overcome barriers was more likely to improve professional practice compared to no intervention or dissemination of guidelines [23]. Flottorp et al. employed rigorous methods to compile and establish consensus on a framework of 57 barriers of guideline implementation organized in 7 broad categories that implementers can use to help identify barriers [8]. Kim et al. first synthesized published research, then interviewed international guideline developers to compile strategies for integrating patient preferences in guidelines, an approach shown to improve relevance and uptake of recommendations [24]. Gagliardi et al. conducted a series of studies that identified and then elaborated on the concept of guideline implementability, referring to content included in or with guidelines such as implementation instructions or tools that can help users to implement recommendations [25]. Squires et al. conducted a meta-review of 25 reviews demonstrating that single interventions were as capable as multifaceted interventions of achieving positive impact [26]. To examine if and how guidelines were implemented based on these principles, the Guidelines International Network Implementation Working Group published a scoping review in 2015 on trends in guideline implementation [27]. The review included 32 studies published between 2004 and 2013. Most included studies employed educational meetings or materials targeted at patients and/or healthcare professionals rather than a range of implementation interventions selected and tailored according to pre-identified barriers, theory, and/or stakeholder engagement, approaches proven to optimize guideline implementation and uptake [8, 23–25]. The study also revealed inconsistent impact on patient and healthcare professional knowledge or behaviour, or clinical outcomes, possibly due to sub-optimal implementation. Moreover, the review included studies of guideline implementation in only four health topics (arthritis, diabetes, colorectal cancer, and heart failure), which resulted in few eligible studies and limited ability to identify trends in guideline implementation over time.

In the 8 years that have passed since literature searches were conducted for the 2015 scoping review, research continues to show that many patients do not access or experience guideline-recommended care. For example, 11 to 45% of American asthma specialists and primary care clinicians complied with asthma guidelines [28], 43 to 62% of 369,251 people from 20 countries achieved diabetes guideline targets [6], 44% of 30,113 Americans at high risk of hepatitis C virus received testing as per guidelines [29], and 36% of 414,851 Americans at 31 institutions did not receive recommended perioperative antibiotic prophylaxis [30]. Hence, further knowledge is needed to understand if guideline implementers are employing aforementioned strategies known to improve use of guidelines and realize associated benefits. The purpose of this study was to update and expand the 2015 scoping review [27]. The aim was to assess trends in guideline implementation, including the implementation strategies or interventions (hereafter, “interventions”) used, the implementation planning approaches employed for selecting and/or tailoring interventions, and the impact on patient or healthcare professional knowledge, behavior, or clinical outcomes.

The following research questions were investigated:

- What approaches were used for implementation planning (i.e., pre-identified barriers, use of frameworks, or stakeholder engagement)?
- What interventions have been used to implement guidelines in any healthcare context?
- Do implementation planning approaches (pre-identify barriers, use of frameworks, stakeholder engagement) or multi-faceted interventions appear to lead to positive impact?

## Methods

### Approach

The scoping review methodological approach was guided by Arksey and O’Malley’s framework and the JBI Manual for Evidence Synthesis and is reported according to the Preferred Reporting Items for Systematic Reviews and Meta-Analyses Scoping Review (PRISMA-ScR) recommendations [31–35], see supplementary file 1 for the completed PRISMA-ScR checklist. A detailed protocol of this scoping review was published in JBI Evidence Synthesis [36]. The authors are members of the Guidelines International Network Implementation Working Group. The purpose of a scoping review is to explore what data is available on a certain topic, as well as whether there is sufficient data available for a more robust systematic review.

### Eligibility criteria

Supplementary file 2 details inclusion and exclusion criteria. In brief, studies were eligible if they evaluated the impact of guideline implementation interventions. Guidelines were defined as documents intended to optimize patient care that include recommendations informed by the best available evidence and an assessment of the benefits and harms of alternative care options [1, 2]. In case studies reported a local guideline, the research team investigated whether these clearly reflected and referenced (inter)national guidelines or were developed according to recognized methods, such as a literature review of the available evidence. Guidelines were considered for inclusion where they target patients aged 18 or older (and/or family or carers) or clinicians (physicians, nurses, allied health) of any specialty. Studies were eligible if they were conducted in primary or secondary/tertiary (hospital inpatient, outpatient, emergency) healthcare settings and published in English, French, or German (languages that could be translated by members of the research team). All authors contributed to development of the eligibility criteria, and two reviewers (AG and SP) further refined criteria based on review of the first 100 search results.

### Search strategy

The search strategy (supplementary file 3) was based on that used for the 2015 scoping review [27] and was updated based on input from the research team and a medical librarian, in accordance with Peer Review of Electronic Search Strategies (PRESS) criteria [37]. AG executed searches in MEDLINE, EMBASE, AMED (all Ovid), CINAHL (EBSCOhost), Scopus, and the Cochrane Database of Systematic Reviews. Articles published from 2014 to January 2021 were included to capture relevant studies published subsequent to the execution of searches for the 2015 scoping review [27].

### Study selection

One researcher (AG) uploaded search results into Covidence (Veritas Health Innovation, Melbourne, Australia) to remove duplicates. To prepare for screening, all screeners reviewed the 2015 publication [27], updated screening criteria, and an Excel file in which AG annotated screening decisions for the first 100 search results. Titles and abstracts were independently screened by two reviewers (SP, SB, AR, JM, PG, ES, MC, ZM, or AG) against the eligibility criteria. Selected titles and abstracts were additionally screened by a third reviewer (AG or SP). Potentially relevant papers were retrieved and imported into Covidence. The full text of selected papers was assessed by two independent reviewers (AG and SP), who noted reasons for exclusion.

### Data extraction and analysis

Data was extracted from included papers by one researcher (SP, SB, AR, JM, PG, ES, MC, ZM, TK, EN, LPR, LK, or AG) and verified by a second researcher (KS). KS discussed any uncertainties or discrepancies with a third independent reviewer (AG or SP). The data extraction template, based on that used in the 2015 review [27] and a few additional items added by the research team, included study characteristics, guideline topic, study objective(s), implementation planning approaches for selecting implementation interventions (including the underpinning theories and frameworks, tailoring to pre-identified barriers, stakeholder engagement, or co-design processes), characteristics of the intervention (target group, single versus multi-faceted, type, content, format, delivery mode, timing, and involved personnel), and impact of interventions. Guideline topics were categorized according to the ICD-11 for Mortality and Morbidity Statistics (ICD-11 MMS) version 02/2022 disease categories [38]. Theories and frameworks were grouped as per Nilsen’s literature review in Implementation Science [39]. Guideline implementation interventions were labeled according to the modified Mazza et al. taxonomy [40] that was expanded in the 2015 scoping review [27]. The taxonomy categorizes 51 interventions organized into five groups: professional, financial, organisational, structural changes, and regulatory [27, 41]. We extracted outcomes as reported by the authors to understand the impact of the intervention employed on each study, where the impact referred to improvements on patient or healthcare professional cognitive (e.g., beliefs, knowledge), behavioral (e.g., prescribing, self-management), or clinical (e.g., physiological measures) outcomes. As noted above in Approach, one purpose of a scoping review is to describe literature on a given topic, and in so doing, identify whether a future systematic review involving complex statistical analyses is feasible. Therefore, we described impact according to three broad categories: positive impact—studies that achieved improvements in all outcomes reported; mixed impact—studies that achieved improvements in some but not all outcomes reported; and no impact—studies that did not achieve improvement in any reported outcomes. Included studies were not appraised for methodological quality or risk of bias as this is not customary for scoping reviews. However, we indirectly addressed study quality by assessing and reporting research design, use of models, theories or frameworks, and thoroughness by which interventions were described.

Data analysis included developing summary statistics and frequency counts. KS developed summary tables and SP used this information to do descriptive statistics in IBM SPSS statistics (version 28.0.1.0). To identify possible associations between implementation planning and impact/outcomes that could be evaluated in a future systematic review, we counted the number of studies that did or did not achieve improvement in reported outcomes.

## Results

### Search results

**Fig. 1:**
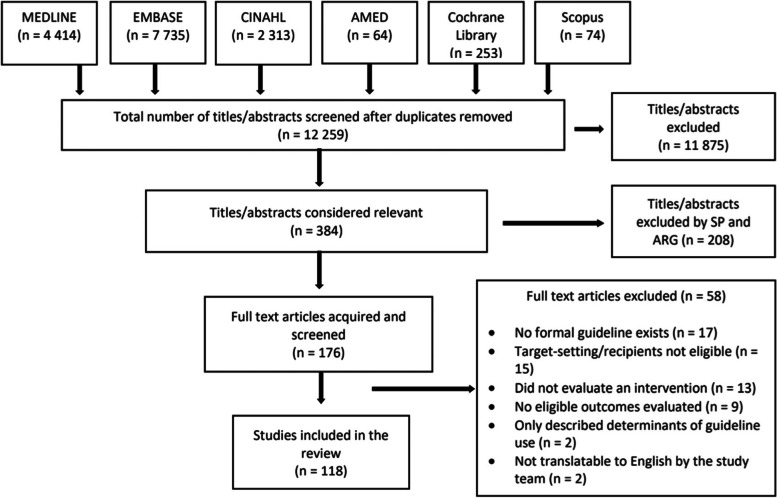
PRISMA flow diagram of search results

The literature search resulted in 15,853 articles (Fig. 1). After removal of duplicates, 11,875 studies were not eligible and 384 were retrieved as potentially relevant. Of these, 208 articles were excluded by two additional reviewers because they did not meet the inclusion criteria. Of the 176 full-text articles acquired and screened, 58 were excluded due to a variety of reasons described in the PRISMA flow diagram, such as the absence of a formal guideline and the impact of the intervention not evaluated. As a result, 118 studies were eligible for review. Details of all included studies are available in supplementary file 4, references [42–159].

### Characteristics of included studies

The highest number of studies were conducted in the USA (44, 37.3%), followed by the Netherlands (12, 10.2%), Australia (11, 9.3%), the UK (8, 6.8%), and Canada (7, 5.9%). With respect to research design, most studies involved an RCT (39, 33.1%), (cross-sectional) pre- and post-design (31, 26.3%) or a cohort study (18, 15.3%). Regarding study objectives, the majority of the eligible studies were undertaken to promote compliance with existing guidelines for quality improvement (106, 89.8%). Twelve studies (10.2%) implemented a newly developed or updated guideline. The guidelines in the included studies addressed the following 16 clinical topics: Diseases of the circulatory system (25, 21.2%); neoplasms (12, 10.2%); endocrine, nutritional, or metabolic diseases (12, 10.2%); mental, behavioral, or neurodevelopmental disorders (9, 7.6%); diseases of the respiratory system (8, 6.8%); injury, poisoning, or certain other consequences of external causes (7, 5.9%); factors influencing health status or contact with health services (7, 5.9%); certain infectious or parasitic diseases (6, 5.1%); diseases of the musculoskeletal system or connective tissue (6, 5.1%); diseases of the genitourinary system (4, 3.4%); diseases of the nervous system (3, 2.5%); diseases of the digestive system (3, 2.5%); external causes of morbidity or mortality (2, 1.7%); diseases of the skin (1, 0.8%); pregnancy, childbirth, or the puerperium (1, 0.8%); and symptoms, signs, or clinical findings, not elsewhere classified (12, 10.2%).

### Implementation planning approaches

Table 1 summarizes the number of studies that selected or tailored implementation interventions based on pre-identified barriers, the use of frameworks and/or employed stakeholder engagement.

**Table 1:** Implementation planning approaches for selecting and tailoring interventions

Implementation planning approach	Details about the planning approach	References	Total studies (***n***, % of studies using the approach)
**Theories & frameworks used** 25/118 (21.2%)	**Process models**		
	• Implementation of change model by Grol and Wensing [160]	[62, 67, 85, 86, 105]	5 (20%)
	• Knowledge-to-Action Framework [161]	[58, 151]	2 (8%)
	**Determinant frameworks**		
	• Theoretical Domains Framework [162]	[48, 68, 70, 74, 85, 105, 132]	7 (28%)
	• Consolidated Framework for Implementation Research (CFIR) [163]	[56, 137]	2 (8%)
	• Promoting Action on Research Implementation (PARiHS) framework [164]	[74]	1 (4%)
	**Classic theories**		
	• Social cognitive theories	[61, 62, 86, 93, 114, 135, 155]	7 (28%)
	• Theory of reasoned action [165]	[56, 86, 117, 134, 152]	5 (20%)
	• Theory of diffusion [166]	[68, 86, 135]	3 (12%)
	**Implementation theories**		
	• Normalization process theory [167]	[85, 105]	2 (8%)
	• Capability, opportunity, motivation—behavior (COM-B) model/behavior change wheel [168]	[52, 85, 103, 105, 132]	5 (20%)
	**Evaluation frameworks**		
	• Reach, Effectives, Adoption, IMplementation (RE-AIM) framework [169]	[94]	1 (4%)
**Barriers pre-identified** 59/118 (50%)	Through the literature	[49, 52, 54, 56, 58, 60–62, 72, 73, 76, 87, 89, 95, 98, 99, 101, 102, 111, 116, 119, 121–123, 125, 131, 134, 147, 148, 151, 153, 156, 158]	33 (55.9%)
	Surveys/questionnaires	[48, 56, 62, 67, 68, 70, 76, 78, 79, 89, 106, 107, 118, 123, 145, 151]	16 (27.1%)
	Group discussions	[42, 45, 93, 103, 114, 118, 123, 132, 137, 141, 150]	11 (18.6%)
	Interviews	[44, 48, 68, 70, 79, 89, 118, 123, 132, 144]	10 (16.9%)
	Focus groups	[56, 62, 78, 97, 103, 116, 118, 123, 141]	9 (15.3%)
	Observations	[76, 99, 103, 131, 141]	5 (8.5%)
	Delphi technique	[86]	1 (1.7%)
	Method not reported	[87, 93, 100, 119, 130, 151, 152]	7 (11.9%)
**Intervention tailored to pre-identified barriers** 38/118 (32.2%)	Behaviour change wheel [170]	[52, 85, 103, 105, 132]	5 (13.2%)
	Method not reported	[42, 45, 56, 60, 62, 67, 68, 70, 76, 78, 79, 86, 89, 98, 100, 102, 106, 107, 111, 114, 116, 118, 119, 121, 123, 130, 131, 134, 137, 141, 151, 152, 158]	33 (86.8%)
**Stakeholder engagement** 42/118 (35.6%)	**Co-design with professionals:**		
	• Group discussions	[45, 52, 55, 58, 59, 65, 77, 84, 94, 97, 103, 119, 125, 128, 131, 132, 137, 141, 147, 149–151, 153]	23 (54.8%)
	• Interviews	[79]	1 (2.4%)
	• Focus groups	[99]	1 (2.4%)
	• Method not reported	[42, 87, 102, 118, 127, 148, 158, 159]	8 (19%)
	**Co-design with professionals & patients**		
	• Group discussions	[85, 89, 93, 123, 152]	5 (11.9%)
	• Method not reported	[106, 126]	2 (4.8%)
	**Co-design with patients**		
	• Group discussions	[98, 105]	2 (4.8%)

#### Theories and frameworks

Of the 118 eligible studies, 25 (21.2%) of those employed a least one theory or framework, as described by Nilsen [39]. Seven studies (28%) used a “process model,” which can help to understand all specific steps involved in the process of translating research into practice. Ten studies (40%) employed a “determinant framework,” which can help to explore all barriers and enablers that influence implementation outcomes. Fifteen studies (60%) utilised a “classic theory,” which is a theory that originates from fields external to implementation science, such as psychology, but can be applied to provide understanding of aspects of implementation. Seven studies (28%) used an “implementation theory,” that has been developed by implementation researchers to help explore explanations of certain implementation aspects. Only one study (4%) employed an “evaluation framework,” which focuses on the evaluation of implementation outcomes. Of the 11 distinct theories and frameworks, most frequently used were the theoretical domains framework TDF (7, 28% of 25), to identify implementation barriers and enablers [48, 68, 70, 74, 85, 105, 132], and social cognitive theories (7, 28%), to provide understanding and explanation of aspects of implementation [61, 62, 86, 93, 114, 135, 155]. Seven (28% of 25) studies used more than one theory or framework. Besides the theories and frameworks as categorized by Nilsen [39], the Plan-Do-Study-Act (PDSA) cycle was used in 11 studies [42, 45, 51, 55, 65, 90, 94, 103, 114, 140, 143], the UK Medical Research Council framework for complex interventions [171] and a program logic model were both used in 1 study, respectively [93] and [118].

#### Pre-identified barriers and tailoring

Fifty-nine (50% of 118) studies identified one or more barriers. Most frequently, barriers were identified by the literature (33, 55.9% of 59), surveys (16, 27.1%), group discussions (11, 18.6%), and interviews (10, 16.9%). Twenty-three (39%) studies used more than one method to identify barriers. Seven (11.9%) studies identified barriers but did not specify how that was done. Of the 59 studies that pre-identified barriers, 38 (64.4% of 59) of those reported that they tailored their intervention to address the barriers. Of those, most did not report which method they used to map pre-identified barriers to implementation interventions, while 5 (13.2% of 38) studies referenced the Behaviour Change Wheel [170].

#### Stakeholder engagement

Stakeholder engagement was employed in 42 (35.6% of 118) studies. Most of the studies referred to co-designing components of their implementation interventions with professionals (33, 78.6% of 42). A smaller number of studies included engagement with patients (2, 4.8%) or with both professionals and patients (7, 16.7%). The majority of the stakeholder engagement sessions were based on group discussions (30, 71.4%). Ten studies (23.8%) did not specify the method that they used to engage stakeholders. In most studies, detailed information was lacking about the extent of stakeholder engagement and how the stakeholder engagement informed intervention selection or design. There were only 3 studies (7.1%) in which the intervention was entirely based on stakeholder engagement [84, 131, 150].

Various implementation planning approaches were used in complementary ways. Twelve of the included studies (10.2%) combined pre-identified barriers with tailoring of the intervention and stakeholder engagement [42, 45, 79, 89, 98, 106, 118, 119, 131, 141, 158]. Nine of the included studies (7.6%) employed a theory or framework, pre-identified barriers, and tailoring [48, 56, 62, 67, 68, 70, 86, 114, 134]. Eight studies entailed pre-identified barriers and stakeholder engagement [87, 97, 99, 125, 147, 148, 150, 153]. One study included a theory or framework, pre-identified barriers, and stakeholder engagement [93], while another study incorporated a framework and stakeholder engagement [94]. Nine of the included studies (7.6%) used all implementation planning approaches in their study [52, 58, 85, 103, 105, 132, 137, 151, 152].

#### Implementation interventions

Table 2 summarizes the implementation interventions used in included studies according to the modified Mazza et al. taxonomy [40]. The majority of the studies involved a multi-faceted (88, 74.6%) rather than a single (30, 25.4%) intervention. Multi-faceted interventions included a mean of 5 interventions (range 2 to 13). Overall, 40 of the 52 distinct interventions types were employed from the modified Mazza taxonomy of guideline implementation strategies. The most frequently used types of interventions were educating groups of professionals about guideline intent and benefits (52, 44.1%), information/communication technology (48, 40.7%), and providing feedback to professionals on compliance (40, 33.9%).

**Table 2:** Implementation approaches and interventions used in eligible studies

Intervention type (modified Mazza framework)	As single intervention (*n*, %) References	As part of a multi-faceted approach (*n*, %) References	Total studies (***n***, %)
**Professional**			
Educate groups about guideline intent/benefits	**6, 5.1%** [76, 81, 90, 101, 121, 145]	**46, 39%** [42, 46, 49, 52, 55, 56, 58, 61, 62, 65, 67, 71, 72, 78, 80, 84, 86, 91, 93, 96–100, 103, 107, 112, 114, 118–120, 123, 128–131, 133, 134, 137, 141, 143, 150–152, 155, 157]	52, 44.1%
Provide feedback on compliance		**40, 33.9%** [42, 49, 52, 58, 59, 62, 65, 72–74, 80, 89, 94, 99, 103, 106, 108, 110, 112–115, 119, 120, 125, 128–130, 132–134, 137, 139, 143, 144, 150–153, 155]	40, 33.9%
Print material (summary, algorithm, referral forms, etc.)	**2, 1.7%** [69, 101]	**36, 30.5%** [42, 46, 50, 51, 53, 55, 56, 58, 60, 62, 66, 67, 70, 72, 78, 80, 91, 92, 94, 96, 116, 118, 119, 123, 125, 130, 132, 137, 139, 143, 150–153, 155, 157]	38, 32.2%
Present guideline materials at meetings	**4, 3.4%** [76, 81, 101, 126]	**33, 28%** [42, 46, 56, 58, 61, 65, 67, 68, 72, 74, 78, 84, 86, 91, 95, 107, 111, 112, 114, 119, 120, 123, 125, 128, 130, 135, 137, 141, 143, 150, 151, 155, 157]	37, 31,4%
Distribute guideline material	**2, 1.7%** [44, 117]	**26, 22%** [58, 60, 63, 67, 68, 74, 77, 85, 86, 88, 100, 105, 113, 116, 118–120, 125, 132, 133, 137, 139, 143, 152, 155, 157]	28, 23.7%
Provide feedback from healthcare professionals		**21, 17.8%** [50, 51, 53, 62, 71, 73, 80, 89, 94, 99, 108, 110, 112, 116, 119, 137, 141, 142, 151, 155, 157]	21, 17.8%
Educate individuals about guideline intent/benefits	**2, 1.7%** [117, 126]	**15, 12.7%** [66–68, 70, 84, 92, 93, 106, 108, 113, 116, 132, 135, 150, 157]	17, 14.4%
Provide reminders to individuals/groups about intent/benefits	**2, 1.7%** [44, 87]	**15, 12.7%** [42, 55, 62, 67, 74, 78, 80, 97, 99, 113, 123, 130, 133, 134, 157]	17, 14.4%
Tailor guideline	**1, 0.8%** [154]	**12, 10.2%** [55, 58, 67, 80, 94, 99, 103, 106, 118, 132, 135, 141]	13, 11%
Recruit an opinion leader who recommends implementation		**11, 9.3%** [62, 68, 103, 108, 110, 114, 123, 131, 137, 139, 151]	11, 9.3%
Enable self-audit (training, material)		**9, 7.6%** [62, 99, 110, 112, 114, 132, 137, 144, 150]	9, 7.6%
Provide alerts when practice deviates	**4, 3.4%** [48, 87, 146, 154]	**5, 4.2%** [83, 112, 115, 137, 144]	9, 7.6%
Provide feedback about patients (outcome data, self-report)		**5, 4.2%** [50, 52, 60, 73, 108]	5, 4.2%
Achieve consensus that guideline should be implemented		**4, 3.4%** [99, 114, 137, 141]	4, 3.4%
Advertise guideline material		**4, 3.4%** [66, 100, 119, 159]	4, 3.4%
**Patient/consumer**			
Education (single or group)		**26, 22%** [43, 51, 54, 57, 62, 63, 71, 72, 78, 83, 85, 97, 102, 105, 106, 111, 116, 118, 120, 123, 128, 133, 139, 142, 152, 156]	26, 22%
Print material (summary, etc.)		**23, 19.5%** [51, 53, 55, 62, 67, 68, 78, 85, 88, 98, 102, 105, 106, 111, 116, 118, 120, 123, 132, 134, 142, 152, 156]	23, 19.5%
Counselling		**13, 11%** [43, 53, 54, 57, 71, 85, 102, 105, 106, 116, 123, 152, 156]	13, 11%
Reminder		**3, 2.5%** [72, 78, 99]	3, 2.5%
**Financial**			
**Health professional**			
Grant or allowance to group/institution (not tied to compliance)		**7, 5.9%** [45, 72, 94, 103, 139, 144, 153]	7, 5.9%
Grant or allowance to individual (not tied to compliance)	**1, 0.8%** [126]	**5, 4.2%** [45, 114, 128, 130, 155]	6, 5%
Incentive (individual financial reward or benefit for compliance)	**1, 0.8%** [122]	**3, 2.5%** [56, 94, 135]	4, 3.4%
Incentive (group or institutional financial reward or benefit)		**1, 0.8%** [79]	1, 0.8%
**Patient**			
Grant or allowance (not tied to compliance)		**1, 0.8%** [83]	1, 0.8%
**Organizational**			
**Health professional**			
Create an implementation/multidisciplinary team		**21, 17.8%** [45, 49, 51, 52, 54, 55, 59, 62, 71, 73, 84, 94, 97, 99, 114, 131, 132, 139, 141, 151, 153]	21, 17.8%
Reallocated or new role		**10, 8.5%** [43, 50, 112, 114–116, 139, 151, 152, 156]	10, 8.5%
Communication between distant health professionals		**9, 7.6%** [43, 45, 52, 71, 97, 99, 111, 139, 141]	9, 7.6%
Additional human resources (number/type)	**2, 1.7%** [47, 124]	**5, 4.2%** [54, 83, 108, 111, 132]	7, 5.9%
**Patient**			
Consumer feedback, suggestions, complaints		**3, 2.5%** [106, 132, 152]	3, 2.5%
**Structural changes**			
Information/communication technology	**15, 12.7%** [64, 75, 87, 104, 109, 127, 136, 138, 140, 146–149, 154, 158]	**33, 28%** [42, 50, 51, 55, 58, 62, 63, 67, 72, 73, 77, 79, 80, 86, 88, 89, 95, 103, 105–108, 110, 111, 116, 128, 130, 132, 133, 137, 143, 144, 159]	48, 40.7%
Quality improvement, performance measurement system	**10, 8.5%** [82, 104, 122, 127, 136, 138, 146, 149, 154, 158]	**15, 12.7%** [73, 80, 89, 96, 97, 99, 110, 112, 114, 120, 130, 132, 133, 139, 144]	25, 21.2%
Method of service delivery	**2, 1.7%** [64, 140]	**19, 16.1%** [45, 46, 50, 51, 57, 65, 78, 85, 98, 99, 105, 106, 108, 111, 119, 128, 132, 143, 159]	21, 17.8%
Integration of services	**3, 2.5%** [148, 149, 154]	**7, 5.9%** [45, 51, 111, 116, 139, 144, 159]	10, 8.5%
Organizational structure (including reorganization)		**3, 2.5%** [77, 111, 139]	3, 2.5%
Physical structure, facilities or equipment		**2, 1.7%** [137, 139]	2, 1.7%
**Total studies**	**30**	**88**	**118**

#### Impact on knowledge, behavior, and outcomes

The majority of studies (66, 55.9%) achieved positive impact, referring to improvements in all outcomes reported, and 31 (26.3%) studies achieved mixed impact, referring to improvement in some but not all outcomes reported. Overall, 97 (82.2%) studies achieved positive impact on one or more reported outcomes.

The most frequently reported impact was healthcare professionals’ behavior (e.g., medication prescribing) (60, 50.8%), see Table 3. Twenty-nine studies (24.6%) targeted both patient/family and healthcare professional related outcomes.

**Table 3:** Outcome measures and type of impact reported in included studies

Target group	Outcome measures	Type of impact reported in included studies (***n***, %) References			Total studies (***n***, %)
		Positive (all reported outcomes improved)	Mixed (some reported outcomes improved)	No change (no outcomes improved)	
Patient/family	Patient outcomes (e.g., reduced cholesterol)	**3, 2.5%** [57, 66, 99]	**---**	---	3, 2.5%
	Behavior (e.g., medication adherence)	**2, 1.7%** [75, 102]	---	**1, 0.8%** [69]	3, 2.5%
	Multiple outcomes	**1, 0.8%** [147]	**1, 0.8%** [130]	**1, 0.8%** [116]	3, 2.5%
Healthcare professional	Knowledge, attitudes, beliefs	**3, 2.5%** [81, 126, 137]	**1, 0.8%** [145]		4, 3.4%
	Behavior (e.g., medication prescribing)	**35, 29.7%** [42, 46, 48–51, 54, 55, 59, 63, 71, 80, 82, 100, 103, 108–110, 112, 113, 115, 119, 125, 127, 128, 132, 133, 136, 138, 140, 144, 146, 153, 154, 157]	**13, 11%** [45, 53, 77, 79, 86, 94, 97, 105, 107, 124, 129, 134, 150]	**12, 10.2%** [47, 58, 65, 67, 91–93, 96, 101, 114, 118, 151]	60, 50.8%
	Institutional/health system outcomes (e.g., reduced mortality or length of hospital stay)	---	---	**1, 0.8%** [84]	1, 0.8%
	Multiple outcomes	**12, 10.2%** [56, 61, 62, 73, 74, 117, 121, 135, 139, 148, 149, 159]	**2, 1.7%** [44, 52]	**1, 0.8%** [88]	15, 12.7%
Both patient/family and healthcare professionals	Multiple outcomes	**10, 8.5%** [76, 78, 83, 87, 120, 122, 131, 141, 142, 158]	**14, 11.9%** [43, 60, 64, 68, 70, 72, 85, 90, 98, 104, 111, 152, 155, 156]	**5, 4.2%** [89, 95, 106, 123, 143]	29, 24.6%
	**Total**	**66, 55.9%**	**31, 26.3%**	**21, 17.8%**	**118, 100%**

The interventions did not report any iatrogenic effects or unintended consequences. Two studies reported negative effects on one of their secondary outcome measures [68, 89].

#### Factors influencing impact

Implementation planning approaches and multi-faceted interventions do not appear to be possibly associated with positive impact (see Table 4), given that there is not a big difference in number of studies with overall positive impact versus mixed or no impact. Studied that used a theory or framework showed only slightly more frequently mixed or no change in study results (12.7%) in comparison to overall positive study impact (8.5%). The same can be said about pre-identified barriers, 26.3% of studies demonstrated mixed or no change, and 23.7% of studies had overall positive impact. The difference between overall positive study impact and mixed or no study impact is slightly bigger for tailoring intervention to pre-identified barriers (12.7 versus 19.5%). Stakeholder engagement revealed in about half of the studies overall positive impact (18.6 versus 16.9%). Single interventions seemed to lead more frequently to overall positive impact (17.8 versus 7.6%), while multi-faceted interventions revealed in about half of the studies overall positive impact (38.1 versus 36.4%).

**Table 4:** Implementation planning approaches/single- versus multi-faceted interventions and study impact

Approach	Overall positive impact (*n*, %)	Mixed or no change (*n*, %)	Total (*n*, %)
Theory or framework used	10 (8.5%)	15 (12.7%)	25 (21.2%)
Pre-identified barriers	28 (23.7%)	31 (26.3%)	59 (0.5%)
Intervention tailored to pre-identified barriers	15 (12.7%)	23 (19.5%)	38 (32.2%)
Stakeholder engagement	22 (18.6%)	20 (16.9%)	42 (35.6%)
Single intervention	21 (17.8%)	9 (7.6%)	30 (25.4%)
Multi-faceted intervention	45 (38.1%)	43 (36.4%)	88 (74.6%)

## Discussion

This review included 118 studies published from 2014 to January 2021 that were largely conducted in high-income countries to improve compliance with existing guidelines or related outcomes across 16 broad disease categories. With respect to implementation planning approaches, 21% studies employed one or more of 11 distinct theories or frameworks, 50% pre-identified barriers using one or more approaches, and 36% reported engaging patients and/or professionals in planning processes. With respect to implementation interventions, a total of 40 intervention types were employed from among the 52 included in the modified Mazza taxonomy of guideline implementation strategies [40], most commonly educating professionals about guidelines (44%), information systems or technology (41%), and informing professionals of their compliance (34%). The majority of studies employed multi-faceted interventions (75%) with a range of 2 to 13 interventions. With respect to impact, 82% of studies achieved improvements in one or more reported outcomes, most often on professional behavior. Beneficial outcomes seemed to be achieved regardless of whether implementation planning was employed, the intervention was single versus multi-faceted, or type of intervention used. However, possible associations are not clear and a future systematic review is needed to more definitively establish that area.

In comparison with the first version of this review published in 2015 [27], this updated review included many more studies across a greater number of disease categories (32 studies in 2015 versus 118 in this review). The studies included in this review more often employed one or more implementation planning approaches compared with 19% that did so in our 2015 review [27]. Similar to the 2015 review, most studies in this review employed multi-faceted strategies. However, unlike the 2015 review in which most studies used educational meetings or material, studies in this review employed a broad range of types of interventions, and information technology and audit & feedback were nearly as common as educational interventions. Similar to the original review, this review found that most studies achieved positive impact, and this was not associated with the use of implementation planning approaches, type of intervention, or multi-faceted approaches.

Previous reviews on guideline implementation identified common implementation interventions or strategies, which included dissemination, education and training, social interaction, decision support systems, and standing orders [172]. A review by Chan et al. specifically focused on the impact of four types of interventions directed at professionals: reminders, educational outreach visits, audit & feedback and incentives, revealing largely positive impact achieved by educational outreach and audit & feedback, and mixed results for reminders and incentives [173]. In contrast to these studies, our review included many more studies and explored the full range of interventions or strategies that have been targeted to patients and/or healthcare professionals. Another added value of our review is that we included a variety of disease categories, while other reviews focused on strategies used to implement guidelines on specific topics, such as nursing guidelines [14, 174]. Our review also included many more studies, namely 118, in comparison to other implementation-focused reviews, which included 41 to 69 studies [14, 172–174].

Spoon et al. found in their systematic review that 43% of studies used barrier assessment to select and tailor interventions [174], which is in line with the 50% of included studies in our review. A systematic review by Cassidy et al. found that most studies combined educational meetings with educational materials, but did not assess how interventions were chosen or factors that influenced impact [14]. In contrast to previously published reviews, our review identified other factors thought to influence impact such as implementation planning approaches and multi-faceted interventions.

A notable finding of this review is that, compared to our 2015 review, more efforts to promote the use of guidelines are informed by implementation planning approaches including the use of theory or frameworks, pre-identification of barriers, and stakeholder engagement in implementation planning. This concurs with another scoping review that focused specifically on use of theory in guideline implementation planning, of 175 included studies, 47% employed theory, and of those, 76% used theory to inform surveys or interviews that identified barriers of guideline use as a preliminary step in implementation planning [12]. This could be attributed to the original Cochrane review that revealed the importance of tailoring interventions [175] and a worldwide trend in engaging stakeholders in both research and quality improvement [176, 177]. This could also be attributed to decades of accumulated research on how to optimize guideline implementation, which has influenced the consciousness and practices of guideline implementers. Similarly, this review showed that the types of interventions employed have broadened beyond educational meetings and materials compared with our 2015 review. This is also likely a reflection of greater awareness of the need for implementation planning to select and tailor interventions that best match a given healthcare context [175], and knowledge of the fact that educational interventions generally have a small impact on professional behavior or outcomes [178]. Given that the vast majority of included studies achieved positive impact in one or more outcomes, this review reinforces the relevance and utility of these approaches for selecting and tailoring interventions, although publication bias could also be at play. Most implementation studies included in our review did not address the costs and economic impact of the interventions, which is an important consideration when informing policy planning decisions.

This review revealed, as have many other reviews [14, 27, 172–174], that most guideline implementers employed multi-faceted interventions. This continuing trend is remarkable because a 2014 meta-review by Squires et al. showed that single strategies were capable of achieving positive impact [26], as did our 2015 review [27] and this updated review. This belief may be perpetuated by reviews of studies that largely employed multi-faceted interventions, many of which achieved positive outcomes, and conclude that multi-faceted interventions are essential [179], and by the belief that interventions should address every barrier identified. However, outside of the context of funded research, most guideline developers possess few resources to implement guidelines, and the vast majority of guidelines are disseminated and not implemented, leading to low rates of compliance with guidelines, of which very few benefit from becoming the subject of implementation research. Thus, further research is needed to generate insight on how to prioritize barriers and corresponding interventions as a means of simplifying both implementation planning and implementation, ultimately making it easier and less costly for guideline implementers to promote uptake of their guidelines.

This review featured both strengths and limitations. With respect to strengths, we used rigorous scoping review methods including triplicate screening by international experts in guideline implementation and duplicate data extraction [31–34, 37] and complied with reporting standards for scoping reviews [180] and for search strategies [37]. By including guidelines on any clinical topic, we expanded the breadth of the findings beyond our original 2015 scoping review [27] and reviews of guideline implementation by others [172, 174]. We also included non-English language studies, those few were identified. With respect to limitations, as with most reviews, our search strategy may not have identified all relevant studies. Although we screened over 12,000 titles/abstracts, our eligibility criteria may have been overly stringent. Studies that achieved improvements in all reported study outcomes are not necessarily more meaningful than those studies that achieved improvements in only some, but not all, of the reported outcomes. We did not undertake complex statistical analyses to quantify study impact or identify determinants of the positive impact of interventions. However, this scoping review identified that sufficient literature is available to do so and we will undertake such analyses in a future systematic review. That systematic review will appraise the methodological quality of included studies, something not required of a scoping review. Additionally, given the lack of risk of bias assessment or a process to establish certainty in the synthesised results, the results of our analyses should be interpreted judiciously and be viewed as indicatory as opposed to confirmatory. The majority of studies were conducted in a few high-income countries so the findings may not be relevant to low- or middle-income countries.

## Conclusion

This review of 118 studies found that more studies used processes to select and tailor interventions, and a wider array of types of interventions, in comparison to a similar review published in 2015. Given that most studies achieved impact, this might reinforce the need for implementation planning approaches, such as pre-identifying barriers, using theory or frameworks, tailoring interventions, and engaging stakeholders in co-design process. However, even studies that did not employ implementation planning approaches achieved impact. Similarly, both single versus multi-faceted interventions achieved impact. Thus, a future systematic review based on this data is warranted to establish if barrier identification, use of theory/frameworks, tailored interventions, stakeholder engagement, and multi-faceted interventions are associated with impact.

## Supplementary Information

**Additional file 1.** Preferred Reporting Items for Systematic reviews and Meta-Analyses extension for Scoping Reviews (PRISMA-ScR) Checklist.

**Additional file 2.** Eligibility criteria.

**Additional file 3.** Search strategy.

**Additional file 4. **Data extraction table.

## Data Availability

All data is available in supplementary files.

## Abbreviations

PRISMA-ScR: Systematic Reviews and Meta-Analyses Scoping Review
ICD-11 MMS: ICD-11 for Mortality and Morbidity Statistics
TDF: Theoretical domains framework
PDSA: Plan Do Study Act
