# Supplementary material for: Trends in guideline implementation: an updated scoping review
Source: Implement Sci. 2022 Jul 23;17:50. doi: 10.1186/s13012-022-01223-6 (PMC9308215; doi:10.1186/s13012-022-01223-6)
Supplement: Supplementary file 2 — Additional file 2. Eligibility criteria. [file 13012_2022_1223_MOESM2_ESM.docx]

**Additional file 2: Eligibility criteria**

**POPULATION**

- Target of implementation can be patients (and/or family or care partners), or clinicians (physicians, nurses, allied health) of any specialty practising in primary or secondary/tertiary (hospital inpatient, outpatient, emergent) care; for example, pharmacists or physiotherapists are eligible if the guideline pertains to primary care team or hospital-based practice
- Guidelines pertain to adults aged 18+ with any disease or clinical condition cared for in above-named settings by specified clinicians
- Those involved in planning or undertaking guideline implementation: can be researchers, guideline developers or implementers, other non-profit organizations (e.g. professional societies, charities, foundations, government, governmental agency) or healthcare leaders/managers or clinicians
  - **Not eligible:**
    - Targets of implementation/evaluation were largely trainees (e.g. medical students, interns, residents, fellows)
    - Clinicians that typically practice outside of primary or secondary/tertiary care in community settings (e.g. dentists, community-based pharmacists, community-based physiotherapists)
    - Guideline recommendations targeted to pediatric patients or those in long-term or institutional care, or home care

**INTERVENTION**

- Guidelines refer to: documents intended to optimize patient care that include recommendations that are informed by a systematic review of evidence and an assessment of the benefits and harms of alternative care options
- Guidelines include new, updated (the most recent version) or adapted guidelines provided standard, rigorous development methods were used (based on citation of a clinical practice guideline and/or standard guideline development manual)
- Guidelines include those developed by non-profit organizations: academic groups, government, governmental agencies, professional societies, charities/foundations
- Guidelines on any clinical topic spanning prevention, screening, diagnosis, treatment and overall management
- Studies in which a guideline is implemented; this can occur in two contexts: when a guideline is newly developed, or for an existing guideline to increase/improve adherence among patients or clinicians
- The study can focus on implementing one or more recommendations from a particular guideline, but must explicitly mention the guideline of interest
- Studies describe and evaluate one or more interventions to implement a guideline
- Interventions include but are not limited to an updated version of the Mazza taxonomy of guideline implementation strategies that emerged from our prior review [SEE modified Mazza taxonomy]; this is more comprehensive than Cochrane EPOC and more specific to guideline implementation than ERIC
- Interventions may be single-faceted (one strategy; for example, an educational meeting; NOTE: an educational meeting that employs didactic and interactive components is not multi-faceted) or multi-faceted (two or more strategies; for example, an educational meeting + audit & feedback)
  - Not eligible:
    - Intervention is the guideline only (compared with group that did not get the guideline)/no intervention to implement the guideline, and authors refer to disseminating the guideline or evaluating outcomes after a guideline was implemented without describing an intervention
    - “Guideline” refers to an organizational protocol (e.g. hospital-imposed rules for ordering/administering medication) rather than a guideline developed using standard methods
    - Aims to study “guideline” implementation, but instead, authors described implementation of a literature/systematic/other types of reviews, recommendations generated via consensus (e.g. Delphi, modified Delphi, RAND appropriateness criteria technique) rather than through standard guideline development processes, or tools potentially derived from guidelines such as clinical/critical paths/pathways, standard operating procedures, etc.

**COMPARISONS/PUBLICATION TYPE**

- Studies published in any language translatable to English by the study team
- Studies quantitatively assess the impact of guideline implementation or use
- May assess guideline use or impact before/after an intervention, or compare guideline use or impact with control/usual care, or between two or more interventions either before/after or only after
- Research design is observational (retrospective or prospective before-after cohort), randomized trial, or case study/program evaluation where “before” data are collected retrospectively from administrative data/medical records and “after” data are collected prospectively
- Studies labelled as a “pilot” or “feasibility” study are eligible if they employ a research design noted above and assess eligible Outcomes
- When more than one article describes the same study, we will include the most recent or thorough article, or multiple articles if they report different outcomes
  - **Not eligible:**
    - Focuses only on guideline development or evaluates guideline development processes
    - Focuses only on describing risk factors or prevalence of the condition and/or its complications or treatment sequelae
    - Describes or evaluates the impact of clinical interventions (e.g. screening, testing, treatment)
    - Describes [lack of] adherence to guidelines, practice patterns, or behaviour/practice requiring de-implementation (audits, population-based studies)
    - Describes determinants (enablers, barriers) of guideline use (i.e. adherence, compliance)
    - Focuses only on describing the intervention but does not evaluate its impact
    - Evaluation focuses on whether/how guideline characteristics/implementability influence guideline use/impact rather than the impact of an intervention
    - Evaluation focuses on education about guidelines in general rather than a particular guideline
    - Qualitative studies or the qualitative component of studies with primarily a quantitative research design (**will be reviewed in a future IWG synthesis)
    - Anecdotal reports or recommendations that, in general, discuss the need for guidelines, or improved implementation of, or adherence to guidelines
    - Systematic reviews, guidelines, commentaries, letters, editorials, conference abstracts/proceedings/papers, protocols

**OUTCOMES**

- Any outcomes reported by eligible studies pertaining to guideline use or impact
- Likely outcomes may include: changes or improvements in clinician behaviour (e.g. an action such as prescribing, using a certain diagnostic test or treatment/procedure), or patient behaviour (e.g. adherence to medication or lifestyle behaviour), or clinical/patient outcomes (e.g. physiological change or improvement)
  - **Not eligible:**
    - Economic analysis/cost of guideline implementation, use or under-/over-use
    - Appraise the quality of guidelines (e.g. AGREE II or rapid appraisal instrument) or discrepancies across guidelines on the same topic
    - Studies that only assess self-reported awareness or knowledge of a guideline or its recommendations
    - Studies that assess self-reported treatment based on simulated cases or scenarios, or observe clinician behaviour based on simulated patients
    - Implementation strategy is not sufficiently described such that it can be categorized/mapped to the taxonomy of guideline implementation interventions
