# Supplementary material for: Trends in guideline implementation: an updated scoping review
Source: Implement Sci. 2022 Jul 23;17:50. doi: 10.1186/s13012-022-01223-6 (PMC9308215; doi:10.1186/s13012-022-01223-6)
Supplement: Supplementary file 3 — Additional file 3. Search strategy. [file 13012_2022_1223_MOESM3_ESM.docx]

**Additional file 3: Search strategy**

MEDLINE (Ovid).

Search conducted on January 8, 2021.

| **#** | **Search Statement** | **Results** |
| --- | --- | --- |
| 1 | Guideline Adherence/ | 32854 |
| 2 | Patient Compliance/ | 58359 |
| 3 | Practice Patterns, Physicians'/ | 61539 |
| 4 | "outcome and process assessment, health care"/ or outcome assessment, health care/ or process assessment, health care/ | 106641 |
| 5 | "diffusion of innovation"/ | 17820 |
| 6 | Information Dissemination/ | 17321 |
| 7 | Translational Medical Research/ | 11244 |
| 8 | implementation science/ | 565 |
| 9 | evidence-based practice/ or evidence-based medicine/ or evidence-based nursing/ | 87829 |
| 10 | (guideline: adj6 (utliz: or utilis: or implement: or disseminat: or uptake or adopt: or impact: or translat: or outcome: or change: or improve: or adhere: or compl:)).ti,ab. | 55728 |
| 11 | (guideline: adj6 (effect: or intervention: or strategy or strategies or approach: or technique:)).ti,ab. | 23380 |
| 12 | or/1-11 | 422949 |
| 13 | *Practice Guidelines as Topic/ | 43487 |
| 14 | 12 and 13 | 17358 |
| 15 | limit 14 to (humans and yr="2014-Current") | 5826 |
| 16 | limit 15 to ("all infant (birth to 23 months)" or "all child (0 to 18 years)") | 817 |
| 17 | 15 not 16 | 5009 |
| 18 | limit 17 to (address or case reports or comment or editorial or guideline or interview or lecture or letter or news or practice guideline) | 595 |
| 19 | 17 not 18 | 4414 |
