# Supplementary material for: Trends in guideline implementation: an updated scoping review
Source: Implement Sci. 2022 Jul 23;17:50. doi: 10.1186/s13012-022-01223-6 (PMC9308215; doi:10.1186/s13012-022-01223-6)
Supplement: Supplementary file 4 — Additional file 4. Data extraction table. [file 13012_2022_1223_MOESM4_ESM.docx]

| **Study****Country****Guideline topic** | **Research Design****Participants** | **Objective** | | **Implementation planning approach** | **Intervention Design** | **Overall Impact****Results** |
| --- | --- | --- | --- | --- | --- | --- |
| Lastname first author  Year  Country  Guideline topic | Research design  Number and type of participants | Context: specify new guideline or quality improvement  Specify brief objective | | Theory: specify formal theory, model or framework or NR  Tailored to pre-identified barriers: specify or NR  Co-designed with patients or professionals: specify or NR | Target: specify patients or professionals  Intervention: specify single or multifaceted  Type: SEE modified Mazza taxonomy  Content:  Format:  Delivery:  Timing:  Personnel:  Control: (if used) | Specify: Positive / Mixed / No change  Details of results |
| Fally [1]  2021  USA  Antibiotic use in community-acquired pneumonia | Cross-sectional, pre- post  Physicians affiliated with respiratory medicine and/or infectious disease departments in emergency or internal medicine contexts overseeing 423 patients in baseline period and 348 patients in follow-up period | Context: Quality improvement  To determine whether an antibiotic stewardship program had an impact on lessening the duration of antibiotic treatment | | Theory: Plan-Do-Study-Act (PDSA) cycles  Tailored to the following barriers identified by the study team in another publication (Davidoff et al., 2015) but selected for applicability to community-acquired pneumonia through seminars and meetings (from study protocol, Fally et al, 2020):   - Lack of information on disease - Lack of clarity on rationale and method for guideline adherence - Lack of practical skills - Ineffective processes, unclear responsibilities - Lack of electronic health record tools   One component of the intervention co-designed in part with a professional: Senior nurse in one of the study sites who designed a hands-on training | Target: Professionals  Intervention: Multifaceted  Type: PROFESSIONAL: Present guideline material at meetings + Educate groups about guideline intent/benefits + Provide feedback on compliance + Print material (posters, pocket cards, newsletter) + Provide reminders to groups about guideline intent/benefits (newsletter); STRUCTURAL CHANGES: Information/communication technology  Content: Community-acquired pneumonia pathogenesis, epidemiology, assessment; appropriate antibiotic prescription, antibiotic choice; audit and feedback based on chart review;  Format: Education sessions (physicians and nurses), personalized face-to-face and email feedback (physicians), Powerpoint presentations, pocket cards, newsletters, posters, order sets in electronic health record  Delivery: In person, electronic health record, print material  Timing:  4-month baseline period, 8-month stewardship program, 4-month follow-up period  15-45-minute education sessions every 1-2 months  Personnel: Education sessions delivered by a member of the implementation team (qualifications NR)  Control: Baseline period data | Modest positive changes  Larger proportions of patients were given reduced antibiotic treatment at follow-up period; corresponding odds ratios with respect to the follow-up period:   - Antibiotics for ≤7 days: 22.2% vs. 34.5%, OR 1.84 (95% CI 1.34-2.54) - Intravenous antibiotics for ≤3 days: 55.3% vs. 58.9%, OR 1.16 (95%CI 0.87-1.54) - Empiric antibiotics according to guideline: 58.6% vs. 73.6%, OR 1.96 (95%CI 1.45-2.68)   *P*-values NR for all of the above |
| Ingram [2]  2021  USA  Medication management in reduced ejection fraction heart failure | Longitudinal, pre- post through retrospective chart review  36 outpatients who had experienced heart failure | Context: Quality improvement  To investigate the impact of pharmacist-led medication management for outpatient heart failure on time taken to achieve target medication doses, and impact on other patient outcomes | | Theory: NR  Tailored to pre-identified barriers: NR  Co-designed with patients or professionals: NR | Target: Patients and professionals  Intervention: Multifaceted  Type: PATIENT/CONSUMER: Education (single) + Counselling; ORGANIZATIONAL (Health professional): Reallocated or new role (pharmacists in heart failure management) + Communication between distant health professionals  Content: Pharmacist visits consisting of:  (i) individualized patient education on heart failure, medication, and lifestyle changes  (ii) pharmacists themselves initiating, modifying, or discontinuing medication based on symptom questionnaire, and laboratory and physical assessments  (iii) pharmacists performing physical examinations related to heart failure after training with cardiologists  Format: Outpatient heart failure clinic visits, lab tests, physical assessments  Delivery: In person  Timing: Referrals to pharmacist from 26 April 2016-31 December 2016 (8 months) were reviewed  Personnel: Pharmacists trained under a collaborative practice agreement  Control: Pre-enrollment data for patients | Mixed results  Average time to achieve target dose of medications was 4.9 visits over 12.7 weeks;   - achievement of target doses was accomplished in 5.7 visits over 15.7 weeks for patients who were not on any guideline-directed medication at the time of enrollment in the study; - target doses were achieved in 4.6 visits over 11.7 weeks for those who had already begun partial titration of a guideline-directed medication with another provider at the time of study enrollment   Average left-ventricular ejection fraction was 30.5% at baseline and 45% after titration, *P* = NR  76% of patients had a left ventricular ejection fraction of ≥35% after study enrollment vs. only 43% of patients at baseline,  *P* = NR  A 13-week pre-enrollment period was compared against a 13-week period immediately after each patient’s 1^st^ pharmacist visit to determine the effect of the intervention on hospital admissions:   - 12 pre-enrollment all-cause hospital admissions vs. 3 post- 1^st^ pharmacist visit admissions, *P* = 0.041 - 5 pre-enrollment heart-failure-related hospital admissions vs. 2 admissions during period following 1^st^ pharmacist visit,   *P* = NR  Two 12-month periods were compared: pre-intervention and a period extrapolated from the 13 weeks following enrollment. These periods were compared for hospital admissions:   - 39 pre-enrollment all-cause hospital admissions vs. 12 extrapolated post-enrollment admissions, *P* = 0.011 - 15 pre-enrollment heart-failure-related hospital admissions vs. 8 extrapolated post-enrollment admissions,   *P* = 0.152 |
| Akamike [3]  2020  Nigeria  Anti-retroviral drugs for HIV | Cohort, pre- post  Pre-intervention: 33 healthcare workers at HIV clinic affiliated with a Nigerian university of whom 25 were medical doctors  Post-intervention: 28 healthcare workers | Context: Quality improvement  To appraise the implementation of national HIV guidelines, and to determine the effect of educational material on health worker knowledge and adherence to guidelines | | Theory: NR  Four ‘key informant’ interviews with clinicians were conducted prior to the intervention to gather information about challenges in guideline implementation. Some barriers identified were:   - Technical difficulties - Lack of printed copy of guideline - Frequent turnover among healthcare workers   Tailored to pre-identified barriers: NR  Co-designed with patients or professionals: NR | Target: Professionals  Intervention: Single  Type: PROFESSIONAL: Distribute guideline material + Provide reminders to individuals/groups about intent/benefits  Content: Information  on the national HIV management guidelines, which providers were urged to read on multiple occasions  Format: Leaflets summarizing key elements of national guidelines  Delivery: Distributed in-person and placed at clinic’s consultation rooms for easy pick-up  Timing: Intervention took place over one month  Personnel: NR | Mixed results, interpret with caution due to possible bias in framing of knowledge items  Participants tested on knowledge items and surveyed on practice-related items; practice-related items were self-assessed  Survey of participants showed an increase in scores (*p* = NR for all) in the following knowledge items:  - Baseline investigation (2.2%)  - Frequency of assessment of CD4 (type of cell) count (11.6%)  - World Health Organization-recommended “staging” (11.9%)  - Frequency of TB screening (13.5%)  - Drug toxicity monitoring (4.4%)  - Knowledge of monitoring toxicity (11.1%)  Survey showed no change or decrease in scores (*p* = NR for all) for the following knowledge items:  - Knowledge level (0%)  - Conditions for beginning antriretroviral therapy  (-5.5%)  Practice-related items:  Decreases in:   - Commencement of antiretroviral therapy based on WHO staging or CD4 (-9%) - Assessment of WHO clinical staging at every visit (-2%)   No difference in:   - Assessment for adherence - Request for CD4 count every 6 months - Monitoring of drug toxicity   Improvements in:   - Screening for TB at each visit (+15%) - Prescription of isoniazid for those eligible (+4.9%) - Prescription of cotrimoxazole (+2.1%) |
| Azizoddin [4]  2020  USA  Distress screening and referrals for patients with cancer | Cohort, pre- post  At least 8 (total number NR) oncologists and oncology fellows + 6 nurses serving 253 unique primarily male army veterans with cancer | Context: Quality improvement  To determine whether a multifaceted intervention improved screening rates for psychosocial distress, and referrals to appropriate professionals among veterans with cancer | | Theory: “Continuous improvement” concept and iterative process adapted from Plan-Do-Study-Act (PDSA) (Taylor et al., 2014), motivational interviewing  A barrier pre-identified in the study during informal monthly clinical meetings was lack of time.  Tailored to pre-identified barriers:  The brevity of the training modules developed for the intervention were tailored to this barrier.  Barriers to the effectiveness of the study were also identified post-hoc.  Co-designed with professionals: Components of the intervention were adjusted based on ongoing feedback solicited from clinicians during informal monthly clinical meetings | Target: Professionals  Intervention: Multifaceted  Type: FINANCIAL (Health Professional): Grant or allowance to group/institution (not tied to compliance) + Grant or allowance to individual (not tied to compliance); ORGANIZATIONAL (Health professional): Create an implementation/multidisciplinary team + Communication between distant health professionals; STRUCTURAL CHANGES: Method of service delivery + Integration of services  Content:  1. Organizing stakeholders  - 4 clinic sessions that assessed patient’s oncology and hematology needs; triage, assessing and chemotherapy infusion  2. Educating clinical staff  -Physicians: Distress screening and how to refer to supportive care services  -Nurses: Distress screening, suicide assessment  3. Conducting distress screening using “Patient Screening Questions for Supportive Care” tool  4. Documentation  -documentation tool to facilitate transfer of screening data onto EMR, open-note section for supportive care service provider  5. Creating action and referral pathways:  -Quality improvement personnel/oncologists explored future steps with patients and had the option of involving other healthcare professionals for further care  Format:  Audit period: Informal meetings  1. Clinic sessions for patients  2. In-person trainings, 3 & 4. Paper screening tool for patient + screening questionnaire and open-note section for provider  5. Referrals  Delivery: In person, hospital computer system, written material  Timing:  Intervention period: 1 year (February – December 2017)  1. Four half-day-long clinic sessions for patients  2. Three 1-hour training sessions  3. 5-10-minute tool completion  5. Referral, if needed, immediately after completion of screening tool  Personnel: Palliative care physician, psychologist, and two psychology students in supervised practice (Quality improvement personnel) | Mixed results    Distress screening rate: 23- 50% of new oncology patients  110 existing patients screened (total patients NR)  Of 63 patients with mild to severe symptoms of anxiety and depression, 23 (36%) were connected in the previous 12 months with psychosocial care; within 6 months of screening 49 (77%) established care with new supportive care service provider(s) as a result of screening and referrals  Though the design of this study is pre- post, no pre- data were reported, nor any associated statistics |
| Azubuike [5]  2020  USA  Lung cancer screening | Cohort, pre- post  Pre- and post-intervention: A 27-patient cohort  Patients of 1 healthcare provider in a clinic staffed by 4 healthcare providers (nurse practitioners and 2 physicians) and 2 medical assistants | Context: Quality improvement  To determine the effect of a phone script and staff education on rates of low-dose CT scans ordered to screen for lung cancer, and knowledge of guidelines pertaining to low-dose CT scans for lung cancer | | Theory: NR  Tailored to pre-identified barriers: NR  Co-designed by patients or professionals: NR | Target: Professionals  Intervention: Multifaceted  Type: PROFESSIONAL: Present guideline material at meetings + Educate groups about guideline intent/benefits + Print material (telephone screening tool + flyers); STRUCTURAL CHANGES: Method of service delivery  Content: Recognition of high-risk patients by project leader; use of telephone screening tool; ordering low-dose CT per guidelines; telephone script with diagnostic questions  Format: Education session, telephone screening tool  Delivery: In person, printed material  Timing: Pre-intervention period: December 2017-February 2018  Intervention period: December 2019-February 2020  1-hour education session  Personnel: Telephone screening tool named after first author, a doctor of nursing practice and registered nurse; project leader qualifications NR  Control: Pre- data | Positive impact  There was a significant difference in the number of low-dose CT scans ordered from the pre-intervention period (n = 0) to the post-intervention period (n = 8;  *P* = 0.0043)  19 of the 27 patients contacted in the post-intervention period decline the low-dose CT scan  There was no difference in provider knowledge between periods |
| Cassagnol [6]  2020  USA  Statin therapy for atherosclerotic cardiovascular disease | Retrospective chart review  Two comparison groups:  Group that had visited an internal medicine clinic + cardiologist clinic: 57 patients  Group that had visited internal medicine clinic only: 211 patients | Context:  Quality improvement  To examine the impact of a cardiologist on the use of guideline-directed statin therapy for atherosclerotic cardiovascular disease in the ambulatory setting | | Theory: NR  Tailored to pre-identified barriers: NR  Co-designed with patients or professionals: NR | Target: Professionals  Intervention: Single  Type: ORGANIZATIONAL (Health professional): Additional human resources (cardiologist)  Content: Treatment by cardiologist  Format: Clinic visit  Delivery: In person  Timing: Chart review of patients with at least one visit to an internal medicine and/or cardiology clinic between May 2016-April 2017 (12 months)  Personnel: Internists and cardiologists | No impact  Although more patients in the internal medicine clinic + cardiology clinic group were prescribed statins (73.6%) compared to the internal medicine clinic only group (50.7%,  *P* = 0.002), there was no statistical difference in appropriate statin use per the guideline between groups  The internal medicine + cardiology clinic group had higher cardiac risk compared to the internal medicine clinic only group as measured by the following risk factors:   - History of clinical atherosclerotic cardiovascular disease: 35.1% vs. 18%,   *P* < 0.001   - Diabetes: 43.7% vs. 30.3%,   *P* = 0.016   - Hypertension: 80.7% vs. 59.2%, *P* = 0.003 - Smoking history: 47.4% vs. 26.1%, *P* = 0.021 - Mean age: 62.1 years vs. 55.5 years, *P* < 0.001 - Non-white ethnicity: 49.6% vs. 29.4%, *P* = 0.021 |
| Ciprut [7]  2020  USA  Prostate cancer imaging | Cohort, pre- post  Veterans diagnosed with low-risk prostate cancer    Pre-intervention: 313 men  Post-intervention 120 men | Context: Quality improvement  To determine whether a clinical reminder order check decreases rate of inappropriate prostate cancer imaging | | Theory: Theoretical Domains Framework  Semi-structured qualitative interviews previously conducted by the study team with patients and physicians, found that clinicians are primarily responsible for initiating imaging, and that clinicians preferred a decision-support tool integrated with their electronic medical record  (Makarov et al., 2016)  The intervention was tailored to these enablers of guideline implementation  Tailored to pre-identified barriers: NR  Co-designed by patients or professionals: NR | Target: Professionals  Intervention: Single  Type: PROFESSIONAL: Provide alerts when practice deviates  Content: Alert when imaging order placed for men with low-risk prostate cancer  Format: Clinical reminder order check integrated with electronic medical record – pop-up alert requires an entry in the text field to override  Delivery: Hospital electronic medical record  Timing: Intervention over 8 months (April 2015-November 2015)  Personnel: NR | Positive impact  Inappropriate imaging of men with low-risk prostate cancer was reduced from 35% (110/133 of those who received imaging) to 19% (23/133) from  pre-intervention to post-intervention period (95%CI 0.73, 0.91, *P* = 0.001)  In qualitative responses, the most common reasons for alert override were “unclear” (67%) |
| Daud [8]  2020  Malaysia  Type 2 diabetes mellitus | Cluster RCT, matched-pairs  888 participants with type 2 diabetes in ten public primary care clinics  Intervention group: 471 participants in five clinics  Control group:  417 participants in five clinics  5 health professionals in intervention, 5 in control group | Context: Quality improvement  To evaluate the effectiveness of a multifaceted intervention (educational meeting and material, site visit) in improving primary care providers’ adherence to type 2 diabetes guidelines | | Theory: Chronic Care Model  Tailored to pre-identified barriers: NR  Barriers pre-identified in the literature pertaining to diabetes management in Malaysia were:   - High patient load - Short consultation time - Cost constraints   (Ramli and Taher, 2008)  Co-designed by patients or professionals: NR | Target: Patients and professionals  Intervention: Multifaceted  Type: PROFESSIONAL: Educate groups about guidelines intent/benefits + Provide feedback on compliance; ORGANIZATIONAL (Health professional): Create an implementation/multidisciplinary team  Content: Creating/strengthening the Chronic Disease Management Team for diabetes care, utilizing clinical practice guidelines and empowering patients with self-management skills  Format: Workshops, booklet, 2 site visits  Delivery: In person  Timing: Total course of study was 1 year  Over 1 year: facilitation and support; two site visits at 3 and 9 months, one workshop at 6 months  Personnel: 2 facilitators  Control: Usual care, access to guideline on internet | Positive results  Significant improvements in the absolute change in the proportion of primary care provider adherence in:   - Recording of BMI (0.6% vs. −1.8%, *p* < 0.001) - Performing foot examination (2.4% vs. 0.6%, *p* < 0.001) - Performing funduscopy/fundus photography (1.5% vs. 0.3%, *p* < 0.001) - Monitoring renal profile (0.9% vs. −0.6%, *p* = 0.001) - Measuring urine protein (1.2% vs. 0.6%, *p* < 0.001) - Giving lifestyle modification and self-management advice (1.2% vs. −0.3%, *p* < 0.001)   in the intervention group compared to the control group at 1-year follow-up  However, significantly greater improvement in absolute change in the proportion of primary care provider in:   - Performing ECG (0.6% vs. −0.9%, *p* = 0.002)   in the control group compared to the intervention group at 1-year follow-up |
| Gupta [9]  2020  UK  Assessment and management of vitamin D adequacy in melanoma patients | Cross-sectional, pre- post  213 patients over 3 stages of audit cared for by plastic surgeons, dermatologists, oncologists, skin cancer nurse specialists and medical secretaries | Context: Quality improvement  To determine how compliant current practice was with melanoma guidelines, and to see if audit and feedback, coupled with the creation of a new clinical pathway, would improve adherence to melanoma guidelines pertaining to the management of vitamin D status | | Theory: NR  Tailored to pre-identified barriers: NR  Co-designed by patients or professionals: NR | Target: Professionals  Intervention: Multifaceted  Type: PROFESSIONAL: Provide feedback about patients (outcome data) + Provide feedback from healthcare professionals + Print material (letter); ORGANIZATIONAL (Health professional): Reallocated or new role (member of skin cancer nurse specialist team); STRUCTURAL CHANGES: Information/communication technology + Method of service delivery  Content: Audit with feedback about patient data; information given to patients about role of vitamin D and recommendations to take supplements; introduction of clinical pathway with a shared list of patients with melanoma, weekly screening of vitamin D results, letter to patient and GP if patient had suboptimal vitamin D levels  Format: Multidisciplinary meetings, chart review, patient registry available in electronic health record, individualized letters and blood reports to GP  Delivery: In person, print material, electronic health record  Timing: Retrospective audit: 1 November 2018-30 April 2019 (6 months)  Reaudit round 1: 11 June-31 October 2019 (4.5 months)  Reaudit round 2: 1 December 2019-31 May 2020 (6 months)  Personnel: Skin cancer nurse specialist in charge of weekly vitamin D result screening, consultants, medical secretaries  Control: Pre- data | Positive impact  Full compliance with secondary care guidelines for the management of vitamin D status in patients with melanoma by reaudit round 2  During the retrospective audit:   1. Skin cancer nurse specialist present at first consult after diagnosis: 43/47 (91.5%) 2. Vitamin D levels checked: 5/47 (10.6%), but not at diagnosis 3. Received vitamin D-related advice at diagnosis: 0% 4. Communication of vitamin D inadequacy to patient: 0% 5. Communication of vitamin D inadequacy to GP: 0%   Reaudit round 1:   1. Skin cancer nurse specialist present at first diagnosis after consultation or shortly thereafter: 66/72 (91.6%) 2. Vitamin D levels checked at diagnosis: 52/72 (72%) 3. Received vitamin D-related advice at diagnosis: 66/72 (92%) 4. Communication of vitamin D inadequacy to patient: 0% 5. Communication of vitamin D inadequacy to GP: 30/72 (42%)   Reaudit round 2:  All of the above measures at 100%  *P*-values NR |
| Holmes [10]  2020  USA  Prevention of cancer-related venous thromboembolism in ambulatory care | Cross-sectional, pre- post  918 sequential outpatients with cancer receiving cancer-directed therapy were assessed; 213 were found to be at high risk and were referred for the intervention | Context: Quality improvement  To develop an effective model to improve venous thromboembolism education and risk assessment rates, and to determine the effect of an electronic-health-record-based program run by a multidisciplinary team on rates of venous thromboembolism risk assessment, education, and prophylaxis | | Theory: Plan-Do-Study-Act (PDSA) cycles  Tailored to pre-identified barriers: NR  Co-designed by patients or professionals: NR | Target: Professionals  Intervention: Multifaceted  Type: PROFESSIONAL: Provide feedback from healthcare professionals; PATIENT/CONSUMER: Education + Print material; ORGANIZATIONAL (Health professional): Create an implementation/multidisciplinary team; STRUCTURAL CHANGES: Information/communication technology + Method of service delivery + Integration of services  Content:  Planning and preparation: PDSA cycles, meetings and updated patient metrics  Part 1: Identification of patients starting outpatient cancer-directed therapy, risk assessment for venous thromboembolism, patient education on signs and symptoms, offering and placing referral to a specialist  Part 2: Appropriate medication and monitoring of patient  Part 3: Individualized bleeding and thrombosis risk assessment during patient consultation, patient-specific action plan, 1-month anticoagulant prescription  Part 4: Assessment of drug interactions, anticoagulant options, drug-specific information  Format: Patient consultation, risk assessment tool integrated with electronic health record, data capture and electronic reporting system, electronic alert, electronic-health-record-based communication tool for health professionals  Delivery: In person, electronic  Timing: 3.5-year study duration with monthly meetings throughout: 1-year planning and preparation phase, 6-month implementation phase, 2-year post-implementation phase  Personnel: Primary oncology nurse, oncology physician, oncology advanced practice providers, thrombosis physician specialist, hematologist, pharmacist, monthly meetings with all members listed above plus study team  Control: Pre-implementation data | Positive impact  Risk assessment and patient education rate was <5% before intervention  During implementation phase, average monthly risk assessment and patient education rate was 81.6% (standard deviation [SD], 11.9; range, 63.6% - 97.7%, compared to pre-intervention rate)  During year 1 of the post-implementation phase, average monthly risk assessment and patient education rate was 94.1% (SD, 5.7; range, NR), and went up to 95.3% (SD, 4.0, range NR) during year 2, with the overall rate being 94.7% (SD, 4.9, range 82.1% - 100.0%) over the 2-year post-implementation period  213 out of 918 (23.2%) sequential outpatients about to initiate cancer-directed therapy were found to be at high risk for venous thromboembolism.  Of high-risk patients, 70.9% were referred to a team member part of the intervention program; 94.4% of those referred arrived for their first visit and 93.8% of those referred received an individually tailored anticoagulation therapy  *P*-values NR |
| Levi [11]  2020  Australia  Intravenous thrombolysis for acute ischemic stroke | Cross-sectional cluster RCT with pre- post data  Intervention group, intervention period: 10 hospitals with an average of 28.1 physicians and nurses/hospital; 3160 stroke cases  Intervention group, follow-up period: 10 hospitals with an average of 22.1 physicians and nurses/hospital; 2527 stroke cases  Control group, intervention period: 10 hospitals with an average of 28.6 physicians and nurses/hospital; 3116 stroke cases  Control group, follow-up period: 10 hospitals with an average of 20.1 physicians and nurses/hospital; 2667 stroke cases | Context: Quality improvement  To investigate  whether a multicomponent, multidisciplinary collaborative intervention could:   1. Increase the proportion of all stroke patients receiving thrombolysis at intervention hospitals, compared to control hospitals 2. Maintain best-practice targets for stroke outcomes 3. Ensure that the rate of adverse events for symptomatic intracranial hemorrhage did not exceed best-practice limits | | Theory: Behavior Change Wheel theoretical framework  Barriers pre-identified in the literature that were mentioned in a paper published alongside the present one (Hasnain et al., 2019) pertaining to intravenous thrombolysis rates were:   - Inefficient in-hospital practices for managing emergency stroke patients - Lack of appropriate infrastructure - Inadequate staffing - Inadequate hospital capacity   (Eissa et al., 2017; Ehlers et al., 2002)  The intervention was tailored to both barriers and enablers identified in Hasnain et al., 2019.  Situational analysis and readiness for change among the sites involved in the present study were assessed through interviews and surveys on barriers and facilitators to thrombolysis. These barriers were identified as a part of the intervention, not in advance.  Co-designed with professionals: Quality improvement teams unique to each site identified site-specific barriers, generated solutions, discussed how to implement solutions, and created their own targets to increase thrombolysis rates | Target: Professionals  Intervention: Multifaceted  Type: PROFESSIONAL: Educate groups about guideline intent/benefits + Provide feedback on compliance + Provide feedback about patients (outcome data on thrombolysed cases); ORGANIZATIONAL (Health professional): Create an implementation/multidisciplinary team (intra-site) + Communication between distant health professionals (inter-site)  Content: Seven interventions comprising situational analysis, target-setting, performance feedback, motivation from primary agent for change, review of thrombolysis rates, inter-site collaborative problem-solving, action planning, performance monitoring, intra-site problem-solving, professional development in clinical decision-making for thrombolysis, performance monitoring, performance feedback  Sites could choose among interventions  Format: Phone calls, site visits, pre-workshop site meetings, workshops, online training modules, teleconferences, telephone performance monitoring, telephone performance feedback  Delivery: In-person, online, telephone  Timing:  Baseline period: January 2011-August 2013 (32 months)  Intervention period: September 2013-December 2014 (16 months)  Follow-up period: January 2015-December 2015 (12 months)  Bi-monthly performance feedback and inter-site teleconferences  Personnel: Clinical lead, site champions, intra-hospital or inter-hospital professionals  Control: Usual care | Mixed results  Average rates of intravenous thrombolysis were roughly equivalent in intervention and control hospitals at baseline, *P*-values NR:  Intervention hospitals: 285/5331 strokes (**5.3%**, 95%CI 4.7%, 5.9%)  Control hospitals: 314/5883 strokes (**5.6%**, 95%CI 5.0%, 6.2%)  Average intravenous thrombolysis rates increased in both intervention and control hospitals during the intervention period, *P*-values NR:  Intervention hospitals: 281/3160 strokes (**8.9%**, 95%CI 7.9%, 9.9%)  Control hospitals: 257/3116 strokes (**8.2%**, 95%CI 7.3%, 9.2%)  Intervention hospitals maintained intravenous thrombolysis rates in the follow-up period, but control hospitals did not quite do so, *P*-values NR:  Intervention hospitals: 221/2527 strokes (**8.7%**, 95%CI 7.6%, 9.8%)  Control hospitals: 210/2667 strokes (**7.9%**, 95%CI 6.9%, 8.9%)  However, when comparing the intervention and control hospitals, there were only statistically significant differences in their respective rates (rates NR) of intravenous thrombolysis at the very end of the intervention period (*P* < 0.05) and at the very beginning of the follow-up period (*P* < 0.05), with higher rates in intervention hospitals  Physicians’ attitude scores increased from baseline to follow-up (change in group mean score=1.4, 95% CI 0.3-2.6; *P* < 0.05) but not attitudes among nurses (*P* > 0.5) |
| Lipscomb [12]  2020  USA  Breast cancer screening for high-risk women and their first-degree relatives | RCT  95 breast cancer survivors and 86 first-degree relatives of the survivors  50 survivors and 35 relatives in high-intensity group (n = 85); 45 survivors and 38 relatives in low-intensity group (n = 83) | Context: Quality improvement  To determine the impact of high-intensity and low-intensity interventions on increasing screening rates for breast cancer survivors  and their first-degree relatives | | Theory: NR  Tailored to pre-identified barriers: NR  Barriers to getting breast cancer screening were discussed with patients and first-degree relatives during the telephone counselling session that was a part of this intervention  Co-designed with patients or professionals: NR | Target: Patients and first-degree relatives + Professionals  Intervention: Multifaceted  Type: PROFESSIONAL: Provide feedback from healthcare professionals + Print material; PATIENT/CONSUMER: Print material + Counselling;  Content:  High-intensity group: Information on importance of breast cancer screening, assessment of barriers in regular screening and recommendations to address such  Low-intensity group:  Information on breast cancer  Format:  High-intensity group:  Educational brochure, telephone counselling session + script of session, info packet and reminder form mailed to primary care physician  Low-intensity group: Educational brochure only  Delivery: Printed material, phone  Timing:  Data collected at 3 months and 12 months  Telephone counselling: 30 minutes  Personnel: Study outreach team composed of graduate research assistants in public health, and study coordinator (qualifications NR)  Control: Low-intensity: educational brochure in mailbox | Mixed results  At three months, survivors in the high-intensity arm  were significantly more likely than survivors in the low-intensity arm to report having received  a mammogram (45.5% vs. 16.0%, *p* = 0.02). However, at 12 months, there was no significant difference  between the interventions (66.7% vs. 79.2%, *p* = 0.31).  Thus, there was a rapid increase in screening in the high-intensity arm closely following the delivery of the multicomponent intervention. Between the three-month and 12-month reporting points, there were gains in both arms, but notably more so in the low-intensity arm. |
| Marszalek [13]  2020  USA  Long-term opioid therapy for chronic non-cancer pain | Cluster RCT with pre- post data  50 veterans with chronic pain receiving opioid therapy  Intervention condition: 25 veteran patients who were randomly selected from a pool of patients who had opted for the program offered by the intervention  Control condition: 25 veteran patients randomly selected from a pool of patients who had either opted not to be enrolled in the program or whose primary care provider had opted them out | Context: Quality improvement  To examine the effectiveness of an interdisciplinary care management team in increasing adherence to 6 guideline items in long-term opioid therapy for chronic non-cancer pain | | Theory: NR  A barrier to guideline adherence among primary care providers that was pre-identified in the literature was:   - Insufficient time to provide naloxone education and prescribing for long-term opioid therapy patients   (Lockett et al., 2018)  Tailored to pre-identified barriers: NR  Co-designed with patients or professionals: NR | Target: Patients and professionals  Intervention: Multifaceted  Type: PATIENT/CONSUMER: Education (group) + Counselling; ORGANIZATIONAL (Health professional): Additional human resources (4 nurses/nurse practitioners, 1 psychologist) + Create an implementation/multidisciplinary team  Content: Review of indices addressed by guideline in patient’s chart, Patient education (recognition and response to overdose, medication storage, mixing drugs), development of patient treatment plan  Format: Chart review, patient education via Powerpoint, electronic m  Delivery: In person, computer program  Timing: Intervention over 6 months  Clinic appointments at least 60 minutes twice/week with intervention team member  Personnel: Intervention team comprising 5 primary care providers (2 nurse practitioners, 2 nurses, 1 psychologist)  Control: Usual care | Positive impact  Results showed increased concordance with guidelines in the intervention group vs. comparison group at 6 months:   - Urine drug screen rate (56% higher, *P* < 0.001, 95% CI 0.33, 0.79) - Prescription drug monitoring program queries (76% higher, *P* < 0.001, 95% CI 0.59, 0.94) - Informed consent rate (48%,   *P* < 0.001, 95% CI 0.32, 0.80)   - Naloxone education/prescriptions (80% higher, *P* < 0.001, 95% CI 0.64, 0.96)   Results showed increased concordance with guidelines in the intervention group post-intervention vs. pre-intervention:   - Urine drug screen rate (44% higher, effect size F = 11.43, *P* = 0.002, partial η^2^ = 0.32) - Prescription drug monitoring program queries (72% higher, effect size F = 61.71, *P* < 0.001, partial η^2^ = 0.72) - Informed consent rate (36%, effect size F = 7.58, *P* = 0.01, partial η^2^ = 0.24)) - Naloxone education/prescriptions (76% higher, effect size F = 76.00, *P* < 0.001, partial η^2^ = 0.76)   Guideline-concordant non-significant decrease in mean morphine-equivalent daily dose in intervention group pre- vs. post-intervention (M = 53.62 vs. M = 40.20, *P* = 0.23, 95% CI 2.04, 25.00, Cohen’s *d* = 0.47) |
| McGuinness [14]  2020  UK  HIV testing in young people and adults with an HIV indicator condition (community-acquired pneumonia) | Cohort, pre- post  177 patients with confirmed diagnosis of community-acquired  pneumonia across four medical wards at a hospital | Context: Quality improvement  To determine whether a trainee-led quality improvement program would increase the rate of HIV testing  offered to patients with community-acquired pneumonia | | Theory: Plan-Do-Study-Act (PDSA), model for improvement methodology (Institute of Healthcare Improvement, 2017)  Tailored to pre-identified barriers: NR  Barriers were identified and targeted over the course of the intervention through PDSA cycles  Co-designed with a professional quality improvement working group: a respiratory consultant, six trainee medical doctors, three respiratory  infection specialist nurses and an HIV specialist nurse | Target: Patients and professionals  Intervention: Multifaceted  Type: PROFESSIONAL: Educate groups about guideline intent/benefits + Tailor guideline + Provide reminders to individuals about intent/benefits + Print material (A1 size posters in offices);  PATIENT/CONSUMER: Print material (informational pamphlet); ORGANIZATIONAL (Health professional): Create an implementation team; STRUCTURAL CHANGES: Information/communication technology  Content:  PDSA cycle 1: Discussion of viability/willingness among staff/utility of offering HIV tests  PDSA cycle 2: Amendment of inclusion criteria  PDSA cycle 3: Nurse review of HIV test status in those with community-acquired pneumonia  PDSA cycle 4: Design of patient-geared educational pamphlets  PDSA cycle 5: Importance of HIV testing during HIV patient admission stage  PDSA cycle 6: Staff-facing to poster as reminder to offer HIV testing  PDSA cycle 7: Adding HIV test to electronic order set  Format: Educational session, posters, pamphlets, presentation, electronic medical record, email reminder, teaching sessions  Delivery: Electronic medical system, online, in person, in-group  Timing: PDSA cycles over 12 months  Personnel: Respiratory consultant/mentor, junior doctors, and nurses | Positive impact  No *P*-values reported  Nine data measurement points: 1 at pre-PDSA cycle 1 + 2 post-PDSA cycle 7 + 6 points between PDSA cycles  Primary outcome:  Patients with community-acquired pneumonia who were offered an HIV test increased from 28% (7/25 patients) at pre-PDSA cycle 1 to 76.4% (13/25 patients)  during the final round of data collection.  Secondary outcomes did not improve:  There was no improvement in the number of HIV test results available on electronic medical record system |
| Nguyen [15]  2020  Vietnam  Tobacco use treatment | Pre- post  89 “village health workers” and groups of 5-6 healthcare providers  including one physician and 3-5 nurses/midwives | Context: Quality improvement    To determine the effect of education and reminders on village health workers’ delivery of proper tobacco use treatment | | Theory: Theory of Planned Behavior; Consolidated Framework for Implementation Research (CFIR) (Damschroeder et al., 2011); Weiner’s model for implementation effectiveness (Weiner et al., 2009; Weiner, 2009; Weiner et al., 2011); Motivational interviewing  Barriers to implementing tobacco cessation in low-to-middle-income countries pre-identified in previous studies by the study team, and in the literature, were inadequate training of providers, lack of systems, and lack of staffing (Shelley et al., 2014; Van Minh et al., 2017)  Another barrier pre-identified in a study previously conducted by the study team was that smoker’s quitlines, which are effective in wealthier countries, may not be as feasible in low-to-middle-income countries (Shelley, Tseng et al., 2014)  Barriers were identified by the study team in a previous study. They conducted four focus groups with village health workers, and coded the themes that emerged into four categories: knowledge and beliefs about Vietnam’s tobacco control program, attitudes towards expanding village health workers’ role in delivering tobacco cessation services, challenges to such, and suggestions of how to address the barriers  (Shelley et al., 2014)  The intervention was tailored to the barriers pre-identified in Shelley et al., 2014.  Barriers to helping smokers quit were also assessed after participating in intervention. They include lack of training/knowledge and lack of resources.  Co-designed with patients or professionals: NR | Target: Professionals  Intervention: Multifaceted  Type: PROFESSIONAL: Present guideline materials at meetings + Educate groups about guideline intent/benefits + Print material; FINANCIAL (Health professional): Incentive (individual financial reward or benefit for compliance): Village health workers were given 20 dollars/month  Content:  Tobacco-use treatment, how to conduct motivational interviewing; delivering screening, advising, and cessation assistance; reminder  Format: Training session, booster training session  Delivery: In-person, printed materials (posters)  Timing:  Intervention over 12 months  4-day initial training, 1-hour booster training at 3-month-mark  Personnel: Trained research assistants trained the village health workers | Positive results  Village health workers’ adherence to tobacco use treatment guidelines increased significantly.  Of all 89 village health workers, proportion that:   - Asked half or more of their patients about patients’ current smoking status significantly improved from 3.4% at baseline to 32.6% at 12 months (*p* < 0.001) - Advised half or more of their patients to quit smoking significantly improved from 4.5% at baseline to 48.3% at 12 months (*p* < 0.001) - Assisted patients in quitting smoking significantly improved from 1.1% at baseline to 38.2% at 12 months (*p* < 0.001)   Mean score in the following measures significantly increased from baseline to 12 months:   - Attitudes (toward providing smoking cessation): 31.2 to 33.0 (*p* < 0.001) - Self-efficacy: 7.3 to 8.6   (*p* < 0.001)   - Norms (standards pertaining to delivering smoking cessation): 5.9 to 6.5 (*p* <0.001)   Village health workers’ self-efficacy and attitudes improved significantly after the intervention.  The findings suggest that, with training and support  systems, village health workers can extend their role to include smoking cessation services. |
| Roberts [16]  2020  UK  Provision of appropriate treatment options for lower-limb osteoarthritis | Pre- post  323 patients with knee and/or hip osteoarthritis referred for physiotherapy and rehabilitation from general practices to two hospital sites | Context: New guideline  To determine whether the intervention improved patients’ functional performance and osteoarthritis-related lower limb pain | | Theory: NR  Tailored to pre-identified barriers: NR  Co-designed with patients or professionals: NR | Target: Patients  Intervention: Multifaceted  Type: PATIENT: Education (group) + Counselling; STRUCTURAL CHANGES: Method of service delivery  Content: Education on pain management, pacing and grading of activities, various exercises with a graded approach to each, advice on lifestyle, goal-setting and self-management  Format: Six in-person education/exercise sessions with 10 stations to go through  Delivery: In-person  Timing:  One 60-minute-long session once/week over 6 weeks  Data reviewed over 3 years  Personnel: Two physiotherapy assistants | Positive results, but no *p* values  Clinically significant (*p* = NR) reduction in pain scores  62.6% (176/281) of patients who had attended 4-6 sessions saw their pain scores reduce, but only 35.7% (15/42) of patients who had attended 1-3 sessions saw a reduction in pain scores, *p* = NR  94.3% (265/281) of patients who had attended 4-6 sessions improved in at least one (of 4) functional outcomes, but only 50% (21/42) of patients who had attended 1-3 sessions improved in at least one (of 4) functional outcomes, *p* = NR  86.5% (243/281) of patients who had attended 4-6 sessions improved in 2+ (of 4) functional outcomes, but only 21.4% (9/42) of patients who had attended 1-3 sessions improved in 2+ (of 4) functional outcomes, *p* = NR  72.2% (203/281) of patients who had attended 4-6 sessions improved in 3+ (of 4) functional outcomes, but only 21.4% (9/42) of patients who had attended 1-3 sessions improved in 3+ (of 4) functional outcomes, *p* = NR  33.5% (94/281) of patients who had attended 4-6 sessions improved in all 4 functional outcomes, but only 4.8% (2/42) of patients who had attended 1-3 sessions improved in all 4 functional outcomes, *p* = NR |
| Rust [17]  2020  USA  Obesity prevention and management in primary care | Retrospective pre-, prospective post  Pre-intervention audit: 51 randomly selected patient charts  Post-intervention audit: 114 patient charts | Context: Quality improvement  To determine the impact of education and an algorithm on staff adherence to obesity prevention and management guidelines | | Theory: Knowledge-to-Action framework’s action cycle phases (Graham et al., 2006)  Barriers that were pre-identified through the literature (by whom NR) that the authors thought were applicable to this clinic are:   - Lack of time - Skepticism - Lack of knowledge of CPG - Age of providers (older provider less likely to utilize recommendations)   Other barriers identified in the literature, pertaining to pediatric obesity, were:   - Other priorities during patient’s visit - Lack of reimbursement for time spent counselling   (Coleman et al., 2012)  Yet more barriers identified in the literature pertaining to obesity were:   - Inconsistent primary team integration of guidelines - Difficulty acknowledging unhealthy weight as a chronic condition, resulting in absence of preventative measures   (Hayes et al., 2017)  Barriers were identified over the course of the study through action cycle phases.  Tailored to pre-identified barriers: NR  Co-designed by professionals: A collaborative of healthcare professionals was involved in the development of evaluation tools and the integration of the guideline into the electronic health record | Target: Professionals  Intervention: Multifaceted  Type: PROFESSIONAL: Distribute guideline material + Present guideline material at meetings + Educate groups of guideline intent/benefits + Provide feedback on compliance + Print material (copy of algorithm) + Tailor guideline; STRUCTURAL CHANGES: Information/communication technology  Content: Obesity prevention and management guideline, importance of prevention and management, supporting data, presentation of clinic’s pre-intervention patient data, specific clinic-level recommendations for implementing guidelines, focus on patient readiness for chance, goal-setting, and weight management strategies; the chosen guideline is delivered through an algorithm that allows weighting of guideline recommendations by provider, and allows for exercise of provider judgement; algorithm itself with clinic-specific goals; integration of guideline into electronic health record; certain guideline measures incorporated into paper medical forms  Format: Education session, hard copy of guidelines, Electronic-health-record-integrated CPG  Delivery: In person, printed material, electronic health record  Timing:  Post-intervention audit: Algorithm use measured within 1^st^ month of education session, patient records audited 12 weeks post-intervention  Personnel: Team lead, clinical practice manager, two physicians  Control: Pre- data | Unclear changes  *P­*-values NR  No pre-intervention data included for comparison, but authors state that there was a “notable improvement” in guideline adherence post-intervention. Therefore, only post- data is reported below.  Among patients who were overweight (n = 56), those:   - Assessed for comorbid conditions: 100% - Assessed for readiness to lose weight: 30.4% - Set at least 1 goal: 41.1% - Discussed weight management strategy: 89.3% - Management strategy was appropriate: 100%   Among patients with obesity (n = 58):   - Assessed for comorbid conditions: 100% - Assessed for readiness to lose weight: 37.9% - Set at least 1 goal: 91.4% - Discussed weight management strategy: 98.3% - Management strategy was appropriate: 100%   When this data was stratified further by body mass index, readiness for change was documented in only 20% of patients whose body mass index was ≥40  Participants were assessed for attitudes, beliefs etc., which were reported in unpublished raw data |
| Segala [18]  2020  Italy  Appropriateness of perioperative antibiotic  prophylaxis | Pre-post  14 surgical departments overseeing  735 patients (for whom 789 prescriptions)  Pre-intervention: 407 patients  Post-intervention: 382 patients | Context: Quality improvement  To evaluate the impact of a 6-year-long antimicrobial stewardship program focusing on indication, selection and dosing, timing, and duration of surgical prophylactic prescriptions | | Theory: NR  Tailored to pre-identified barriers: NR  Co-designed with professionals: Local surgical antibiotic prophylaxis recommendations were revised by the antimicrobial stewardship team, which was composed of an Infectious Diseases specialist, surgeon, pharmacist, and member of the teaching hospital’s Clinical Governance staff | Target: Professionals  Intervention: Multifaceted  Type: PROFESSIONAL: Provide feedback on compliance;  ORGANIZATIONAL (Health professional): Create a multidisciplinary team  Content: Structured audit meetings with personnel from each department, Discussion of surgical antibiotic prophylactic prescriptions with all personnel in charge of prescribing  Format: Structured audit meetings, feedback  Delivery: In-person  Timing:  Audit meetings at least 1-hour,  Discussion 4 months after first survey period (in April 2014)  Evaluation 6 years after implementation  Personnel: Antimicrobial stewardship team consisting of Infectious Diseases specialist, surgeon, pharmacist, member of Clinical Governance staff | Positive results  Overall appropriateness of  guideline adherence significantly improved from 36.6% (n = 149) at baseline to 57.9% (n = 221) post-intervention (*p* < 0.0001)  With respect to surgical antibiotic prophylactic prescriptions, all measures improved significantly:  Indication appropriateness was 58.5% (n = 238) at baseline vs. 93.2% (n = 356) post-intervention  (*p* < 0.0001)  Selection and dosing appropriateness was 58.5% (n = 238) at baseline vs. 80.6%  (n = 308) post-intervention  (*p* < 0.0001)  Timing appropriateness was 92.4% (n = 376) at baseline vs. 97.6%  (n = 373) post-intervention  (*p* = 0.001)  Duration appropriateness was 71% (n = 289) at baseline vs. 80.1%  (n = 306) post-intervention  (*p* = 0.002) |
| Silverberg [19]  2020  Canada  Management of mild traumatic brain injury in primary care | Pilot cluster RCT  114 randomized clinics with 137 family physicians caring for 148 patients with persistent symptoms | Context: Quality improvement  Evaluating the need for treatment, feasibility of an intervention—including using patient recall to measure physician behaviour, agreement between patient recall of treatment measures and chart review of physician behaviours, and patient clinical outcomes—and determining parameters for a larger trial | | Theory: NR  Barriers identified from the literature were (Rashidian et al., 2008; Cabana et al., 1999):   - Time constraints - The challenging nature of assessing mild traumatic brain injury due to its many diverse symptoms   Another barrier identified from the literature was (Chrisman et al., 2011; Stoller et al., 2014):   - Doctors’ reduced confidence in their own knowledge/skills and recollection of practice guidelines due to the small number of mild traumatic brain injury patients they see   The intervention was tailored to all the above pre-identified barriers and enablers of guideline implementation by prioritizing the assessment of certain symptoms, tailoring the questions in the screening tool to the patient, and administering the tool at the point of care  Co-designed by patients or professionals: NR | Target: Professionals  Intervention: Multifaceted  Type: PROFESSIONAL: Distribute guideline material + Provide feedback about patients (outcome data) + Print material (follow-up letter)  Content: Screening patients for depression, anxiety, insomnia, headaches; follow-up letter to physician with screening results, associated (patient-tailored) management recommendations and treatment algorithms distilled from guidelines  Format: Paper screening and follow-up letters  Delivery: Print material  Timing: Patients completed baseline questionnaires between July 2017 and October 2018  Follow-up was at 1 month and 3 months  Personnel: Lay interviewer  Control: Follow-up letter to physician instructed physicians on where to access guidelines, screening results not reported | Mixed results  As reported in the study: per patient recall of their physician’s actions, rates of guideline-compliant treatment behaviours were 8-12 percentage points higher in the intervention group vs. control group for depression/anxiety, but not always higher in the intervention group for headaches and sleep issues  However, no formal statistical methods were undertaken to compare patient recall of physician actions. Some actions showed no difference between groups, or a better turnout in the control group  18 patient charts from the intervention period were audited  Patient recall rates and physician documentation of referrals and prescriptions were compared  Absolute agreement rates between patient recall and physician behaviour are reported for referrals and prescriptions (with intraclass correlations and *p*-values):  Mood-related: 81% (ICC = 0.65,  *p* = 0.024)  Sleep-related: 81% (ICC = 0.48,  *p* = 0.101)  Headache-related: 75%  (ICC = 0.53, *p* = 0.072)  Patient outcomes: A score based on a post-concussion symptoms questionnaire was computed for each patient  The main effects for group and time were significant. Post-concussion symptom scores reduced from month 1 to month 3 (B = −5.6, 95% CI: −7.9 to −3.3), *p* = NR  Being in the intervention group vs. the control group (B = −4.0, 95% CI: −7.3 to −0.7) was also associated with fewer or less severe post-concussion symptoms, *p* = NR |
| Tramontt [20]  2020  Brazil  Promotion of healthy eating in primary care | Cohort, pre- post with non-equivalent control  Two non-randomized cohorts (allocated by geographical location):  Intervention group: 12 professionals  Control group: 11 professionals | Context: Quality improvement  To evaluate the effect of a guideline-based educational workshop on knowledge of nutritional guidelines, and self-efficacy and collective efficacy in advising guideline-concordant eating | | Theory: Adult Learning Theory, Interprofessional Collaborative Practice Model  Barriers identified through the literature were (Kris-Etherton et al*.*, 2014; Food and Agriculture Organization of the UN, 2011; Lucas et al*.*, 2019):   - Insufficient training about nutrition - Challenges in addressing food-related issues with patients - Systemic barriers (NR) in professional practice that make it difficult to guide patients about food   Tailored to pre-identified barriers: NR  Co-designed by patients or professionals: NR | Target: Professionals  Intervention: Multifaceted  Type: PROFESSIONAL: Present guideline material at meetings + Educate groups about guideline intent/benefits  Content: Educational workshops with a focus on encouraging interaction and engagement with the material:  i) Guideline comprehension – addressing and expanding on guideline recommendations, articulating existing knowledge among practitioners with guideline recommendations, e.g., each professional encouraged to reflect on their own understanding of healthy eating; how to choose foods; obstacles to healthy eating  ii) Guideline implementation – how to implement the guidelines in clinical practice  Format: Four interactive educational workshops  Delivery: In person  Timing: Four 4-hour workshops (16 hours) over 2 days  Knowledge tested pre-intervention and then again after 60 days  Control: Usual work routine  Personnel: Facilitators (qualifications NR) led the workshops | Positive impact  There was a significant change in the knowledge scores of the intervention group before the workshops vs. after the workshops: 10.83 vs. 12.83, 2.0 difference (95%CI 0.49 to 3.51),  *p* = 0.007  The control group also showed an improvement in knowledge scores, though not statistically significant: 12.25 vs. 13.64, 0.82 difference (95%CI -0.34 to 1.97), *p* = 0.073  Self-efficacy was significantly improved in the intervention group (*p* < 0.001) and showed a borderline significant improvement in the control group (*p* = 0.051)  Collective efficacy did not show improvements in either group |
| Trogrlic [21]  2020  USA  Delirium treatment in ICUs | Pre- post  6 ICUs comprising 81 physicians and 409 nurses; 4449 patients  Local implementation expert teams: 2-11 professionals per ICU | Context: Quality improvement  To explore:   - The exposure of healthcare workers to a delirium guidelines implementation program - The effect of the program on guideline adherence at the ICU level - The impact of the program on knowledge, barriers, and experiences with the implementation | | Theory: Implementation Model of Grol and Wensing (Grol and Eccles, 2013); framework for perceived barriers by Cabana et al. (1999) and Grol et al. (2005) (from adjunct qualitative paper, Trogrlic et al., 2017); Social learning theory, Social influence theory (from Trogrlic et al., 2019)  Barriers pre-identified in the literature pertaining to delirium guideline were:   - Low confidence in the ability of screening tools to identify delirium (from Trogrlic et al., 2017: Flagg et al., 2010) - Lack of knowledge of delirium (from Trogrlic et al., 2017: van den Boogaard et al., 2009; Gesin et al., 2012) - Low perceived importance of delirium among professionals (from Trogrlic et al., 2017: Devlin et al., 2008) - Fear of adverse events - Communication and care coordination challenges - Workload concerns - Documentation burden   (from Trogrlic et al., 2017: Balas et al., 2012)  Barriers were also pre-identified in the literature relating to guideline implementation in general  Barriers, as well as other variables, that were pre-identified by the study team through electronic surveys, focus groups, and a subsequent barrier analysis were:   - Insufficient knowledge of screening - No delirium protocol with a link to screening results   (from Trogrlic et al., 2019)  Interventions were tailored to these pre-implementation barriers using the Grol and Wensing model and other change theories (from Trogrlic et al., 2019)  Barriers were also assessed over the course of the intervention  Co-designed by patients or professionals: NR | Target: Professionals  Intervention: Multifaceted  Type: PROFESSIONAL: Educate groups about guideline intent/benefits + Provide reminders to groups about guideline intent/benefits (poster) + Provide feedback from healthcare professionals + Provide feedback on compliance + Recruit an opinion leader who recommends implementation + Enable self-audit + Print material (posters); PATIENT/CONSUMER: Education + Print material (poster and booklet); ORGANIZATIONAL (Health professional): Create an implementation/multidisciplinary team; STRUCTURAL CHANGES: Information/communication technology  Content: Rates of delirium screening adherence and delirium incidence; severity and impact of delirium in the long and short terms, importance of screening; consolidation of cross-clinic protocol in keeping with delirium guidelines; encouraging guideline adherence; tailored advice on the management of specific patients; informing and involving family members of patients  Format: Audit and feedback, print material (reminder posters), e-learning website with instructional videos, educational meetings, educational outreach visits, academic detailing, consensus groups, inter-professional education, local opinion leaders, electronic reminders, daily rounds and visits, informational patient poster, patient leaflet  Delivery: In person, print material, online, mobile app  Timing:  Study duration of 36 months  Phase I: Baseline data collection (4 months), followed by usual care (10 months)  Phase II: Implementation (4 months) and post-implementation of screening tools (4 months)  Phase III: Guideline implementation (8 months) and post-implementation of guideline (4 months)  Time spent on two e-learnings was 45 minutes/person  Personnel: Local opinion leaders (intensivists or nurses)  Control: Pre-intervention data | Positive changes  Significant guideline-concordant improvements in 6/7 primary outcome measures from Phase I to Phase II to Phase III (from Trogrlic et al., 2019):   1. Delirium screening: 35% to 93% (*P* < 0.001) to 96%   (*P* < 0.001)   1. Sedation assessments 2. Number of days in light sedation, 3. Use of continuous intravenous benzodiazepine sedation: 36% to 31% (*P* < 0.001) to 17%   (*P* < 0.001 relative to Phase I)   1. Physiotherapy performed 2. Performing mobilization when feasible   from Phase I to Phase II (upon introduction of screening tool); and Phase II to Phase III (upon guideline intervention) in all except for one of above six measures  Improvement was sustained 6 months after active implementation had ended.  Secondary (clinical) outcome measures (from Trogrlic et al., 2019):  Delirium decreased over all three periods from 5.6 days to 2.9 days (Beta: -2.6 days; 95% CI, -3.5 to -1.6 days; *P* < 0.001); and to 3.3 days after Phase III (Beta: -2.2 days; 95%CI, -3.2 to -1.3 days;  *P* < 0.001)  Implementation of delirium screening resulted in 6% more patients with delirium who were detected in Phase III compared with Phase I (OR 1.4**;** 95%CI 1.2 to 1.7; *P* < 0.001)  Beliefs, attitudes, practices, knowledge, and barriers and facilitators to guideline adherence among ICU nurses and physicians were assessed, with results reported in a previous study by the study team |
| Vani [22]  2020  USA  Use of statins and antithrombotic agents  in patients with atherosclerotic cardiovascular disease | Pre- post  Hospital providers (nurse practitioners, physician assistants, graduate medical education trainees) overseeing 11553 patients  Pre-intervention: 5985 patients  Post-intervention: 5568 patients | Context: Quality improvement  To determine the impact of  clinical decision  support and guideline distribution on appropriateness of medication for atherosclerotic cardiovascular disease | | Theory: NR  Tailored to pre-identified barriers: NR  Co-designed with patients or professionals: NR | Target: Patients and professionals  Intervention: Multifaceted  Type:  PROFESSIONAL: Distribute guideline; PATIENT/CONSUMER:  Education; STRUCTURAL CHANGES: Information/communication technology  Content:  Clinical decision support system:  Best practice alert 1: Notification of recent hemoglobin and lipid results with option to repeat testing  Best practice alerts 2 and 3: Recommendations for statin and antithrombotic therapy at discharge, link to aid with appropriate prescription of above medications  Embedded in the electronic health record of selected patients with diagnosis of atherosclerotic cardiovascular disease who did not have an existing prescription for high-intensity statins or antithrombotics  Patient education materials: Individualized blood values, individualized descriptions of atherosclerotic cardiovascular disease diagnoses, diets conducive to risk reduction, physical activity recommendations,  and other lifestyle resources  Guideline distribution  Format: Best practice alerts in electronic health record, educational materials, guideline itself  Delivery: Electronic hospital system, printed material  Timing: Data collected over 2 years  Pre- and post-discharge medication data collected for each patient  Personnel: NR | Positive results  Patient education was provided after best practice alerts and prescriptions, and did not contribute to the intervention per se.  Pre-implementation vs. post-implementation, a 4.0% increase was identified in  prescriptions for statins at discharge (*p* < 0.001), a 3.6% increase in high-intensity  statins at discharge (*p* < 0.001), and 3.1% increase in antithrombotic  agents at discharge (*p* < 0.001) over all admission types (inpatient, observational, and outpatient) |
| Wu [23]  2020  USA  Quality of care in non-small cell lung cancer | Retrospective cohort, prospective cohort  Retrospective cohort: 157 patients with non-small cell lung cancer  Prospective (trial) cohort: 76 patients with non-small cell lung cancer | Context: Quality improvement  To investigate the effect of a patient-oriented decision support tool on adherence to 6 measures of guideline adherence for non-small cell lung cancer | | Theory: NR  Tailored to pre-identified barriers: NR  Co-designed by patients or professionals: NR | Target: Patients and professionals  Intervention: Single  Type: STRUCTURAL CHANGES: Information/communication technology + Method of service delivery  Content: Entry of patient disease characteristics into the web-based application by patients themselves; patients were assisted in exploring of guideline-recommended treatment options; patient involvement in course of their treatment; decision tool displayed during consultation with doctor; research coordinator presence during consultation  Format: Doctor’s consultation, web-based guideline tool  Delivery: In person, electronic  Timing: Retrospective cohort information collected 8 months before trial  Prospective cohort enrolled 23 February 2015- 28 September 2017  Personnel: Patients were assisted with navigating web-based interface by trained research coordinator  Control: Retrospective cohort | Mixed results  Of 6 quality indicators, only 2 changed, of which 1 bore a difference because of a statistical difference between the prospective (trial) and retrospective cohorts  Of self-reported active smokers, more smokers in the trial cohort were offered smoking cessation counselling (4/5, 80.0%) vs. in the retrospective cohort (1/24, 4.2%;  *P* < 0.001)  Fewer patients with stage IB or IIB disease in the trial cohort were offered adjuvant chemotherapy following surgery in the trial cohort (0/7, 0%) vs. in the retrospective cohort (7/11, 63.6%, *P* < 0.01), but a post hoc analysis attributed this to no patients in the trial cohort having negative margins whereas 4/6 patients in the retrospective cohort did  Use of the tool was associated with less decision-making conflict  (*P* < 0.001) and greater satisfaction among patients (*P* = NR) |
| Zgierska [24]  2020  USA  Opioid prescribing for chronic non-cancer pain | Non-randomized stepped-wedge quality improvement project  215 volunteer clinicians (e.g., physicians, nurses, physician assistants) with at least partial completion of Quality Improvement intervention | Context: Quality improvement  To investigate whether the intervention impacted clinician adherence to the policy recommendations derived from the opioid prescribing guidelines | | Theory: Plan-Do-Study-Act (PDSA) cycles  Tailored to pre-identified barriers: NR  Co-designed with the input of an interdisciplinary team of health leaders and clinicians | Target: Professionals  Intervention: Multifaceted  Type: PROFESSIONAL: Present guideline materials at meetings + Educate groups about guideline intent/benefits + Provide feedback on compliance; STRUCTURAL CHANGES: Method of service delivery  Content: National opioid-prescribing guidelines, and the health system's opioid policy recommendations; decision-making when providing opioid therapy for chronic pain, guideline and health system policy recommendations for opioid therapy management. Practice facilitation sessions focused on optimizing clinical workflows.  Format: one academic detailing session, two educational modules, six practice facilitation (PF) sessions  Delivery: In-person, online  Timing: 3 sessions:  1) 1-hour education session; 2) Two 20 question online modules;  3) Six 1-hour practice facilitation sessions, delivered over 4-6 months  Personnel: Project physicians and project’s trained practice facilitators (qualifications NR) | No change  No significant difference between intervention and comparison groups in patient population as a whole, and those treated with ≥90mg/day of morphine equivalent opioid dose:   - completed treatment agreement (*p* = 0.816;   *p* = 0.783)   - completed depression screening (*p* = 0.526;   *p* = 0.528)   - completed opioid misuse risk assessment (*p* = 0.417;   *p* = 0.582)   - documented PDMP check   (*p* = 0.301; *p* = 0.263)   - co-prescribed benzodiazepines in at least 1/3 past months   (*p* = 0.143; *p* = 0.486)   - % of adult clinic population   (*p* = 0.547; *p* = 0.224)   - Morphine-equivalent dose in those prescribed ≥90mg/day in past 90 days (*p* = 0.270;   *p* = 0.356)  No difference between intervention and comparison groups in average morphine-equivalent dose in past 90 days  (*p* = 0.937)  No significant difference in intervention vs comparison group in completed urine drug testing (*p* = 0.277)  Significant increase in completed urine drug testing in intervention clinic versus comparison clinic in those prescribed morphine-equivalent dose of ≥90mg/day (*p* = 0.020)  Staff were asked about their confidence, attitudes, and barriers and facilitators in managing patients with opioid-treated chronic pain, results NR |
| Abbood [25]  2019  Iraq  Post-operative fluid prescribing | Pre-post  Hospital surgeons (number NR)  Pre-intervention group: 84 patients  Post-intervention group: 112 patients | Context: Quality improvement  To evaluate the current practice of post-operative fluid prescribing, and to assess the effectiveness of a pharmacist-led education to improve post-operative fluid prescribing | Theory: NR  Tailored to pre-identified barriers: NR  Co-designed with patients or professionals: NR | | Target: Professionals  Intervention: Multifaceted  Type: PROFESSIONAL: Educate individuals about guideline intent/benefits + Print material + Advertise guideline material  Content: NICE 2013 post-operative fluid prescribing guideline  Format: Educational seminars; posters  Delivery: NR  Timing: 2-month pre-intervention observation phase,  1-month intervention phase, 2-month post-intervention observation phase  Personnel: Research pharmacist  Control: NA | Positive impact  For post-operative Day 1 and Day 2, the proportion of patients categorized as within the range of recommended fluid compared to those receiving amounts below the range or above the range increased significantly from pre-intervention to post-intervention, *p* < 0.001  Fewer patients had electrolyte abnormalities (hypernatremia or hyponatremia, hyperkalemia, hypokalemia) post-intervention compared to pre-intervention.  Normal sodium levels: 91.1% vs. 75% post-intervention vs. pre-intervention (*p* = 0.009)  Normal potassium levels:  98.2% vs. 92.9% (*p* = 0.046)  Post-operative serum creatinine levels improved post-intervention compared to pre-intervention (0.65 vs. 0.625, *p* < 0.05) |
| Bernhardssonn [26]    2019  Sweden  Physiotherapy for subacute lower back pain, subacute neck pain, and subacromial pain | Non-randomised controlled study  256 physiotherapists at follow-up  Intervention group at follow-up: 168 physiotherapists  Control group at follow-up: 88 physiotherapists | Context: New guideline  Examine the effect of a tailored, multicomponent guideline implementation intervention on  self‐reported clinical practice for three musculoskeletal conditions | Theory: Grol and Wensing implementation of change model (Grol et al., 2005)  Tailored to pre-identified barriers: Tailored to barriers and facilitators identified by the study team in a previous study (Bernhardsson et al., 2014), through a self-developed questionnaire about attitudes to, knowledge of, and barriers, with respect to both evidence-based practice and guidelines in physical therapy  Self-reported barriers to the use of the guideline identified by physiotherapists were, in order of prevalence:   - Lack of time - Not knowing where to find the guideline - Generality/non-specificity of guidelines - Takes too long to read - No/too few guidelines exist - Too formulaic - Lack of support from colleagues - Lack of interest   (Bernhardsson et al., 2014)  More barriers related to attitudes and knowledge can be found in Bernhardsson et al., 2014.  Co-designed with patients or professionals: NR | | Target: Professionals  Intervention: Multifaceted  Type: PROFESSIONAL: Distribute guideline material + Present guideline material at meetings + Educate groups about guideline intent/benefits + Educate individuals about guideline intent/benefits + Provide reminders to individuals about intent/benefits + Print material (guideline) + Tailor guideline; PATIENT/CONSUMER: Print material (leaflets); STRUCTURAL CHANGES: Information/communication technology  Content and Format: Guidelines (printed and electronic formats); 3‐hour implementation seminar: education on evidence-based practice, presentation of guidelines, interactive group discussions focused on guideline recommendations and current practice; a specially developed guideline website; email reminders; patient information leaflets; email and telephone support  Format: multiple – see above  Delivery: In person, online, print material, email, telephone  Timing: Seminar held over 3 months on 9 separate occasions  Personnel: Primary care physiotherapists  Control: Care as usual | No change  The study showed no relevant significant differences  in the use of various treatment methods for lower back pain, neck pain, and subacromial pain, after the intervention was implemented  Low back pain  No significant differences between intervention and control groups in:   - 7 of 8 guideline-recommended treatments   There was a significantly greater proportion of people in the **control group** who were treated with 1 guideline-recommended treatment: spinal manipulation (22.6% vs. 8.5%, a 14.1% (95% CI 4.2% to 25.4%) difference)  Of 15 non-guideline-recommended treatments, only body awareness training was offered at a significantly greater rate to patients in the intervention group (22.6%) vs. to those in the control group (6.0%; a difference of 16.6%, 95% CI 6.6% to 24.8%)  Neck pain  No significant differences between intervention and control groups in all 5 guideline-recommended treatments  Of 18 non-guideline-recommended treatments, only body awareness training was offered at a significantly greater rate to patients in the **intervention group** (23.6%) vs. to those in the control group (7.1%; a difference of 16.5%, 95% CI 6.2% to 25.0%)  Subacromial pain  No significant differences between intervention and control groups in all 5 guideline-recommended treatments  No significant differences between intervention and control groups in all 16 non-guideline-recommended treatments |
| Bosch [27]  2019  Australia  Management of mild traumatic brain injury in the emergency department | Cluster RCT  Intervention group: 14 emergency departments  Control group: 17 emergency departments  1943 patients in chart audit | Context: Quality improvement  To evaluate the effect of a targeted, theory-informed implementation intervention to increase the uptake of 3 clinical practice recommendation regarding the management of patients with mild head injuries | Theory: Theoretical Domains Framework, Model of Diffusion of Innovation in Service Organizations  Barriers and enablers were pre-identified by the study team through in-depth semi-structured face-to-face and telephone interviews.  The barriers and enablers were many (Bosch et al., 2016; Tavender et al. 2014), and the intervention was tailored to them.  Co-designed by patients or professionals: NR | | Target: Professionals  Intervention: Multifaceted  Type: PROFESSIONAL: Distribute guideline material + Present guideline materials at meetings + Educate individuals about guideline intent/benefits + Recruit an opinion leader who recommends implementation; PATIENT/CONSUMER:  Print material (summary, etc.)  Content: Key recommendations and underlying evidence, intervention components and how to overcome barriers  Format: Stakeholder meeting, training workshop, PowerPoint presentations, screening tools, patient information booklets  Delivery: In-person, printed material  Timing: 1-hour stakeholder meeting followed by training workshop (one-day-long). Trainers then had 3 months to train staff.  Personnel: Local opinion leader, content experts, senior clinicians were part of implementation group  Control: Guideline dissemination + data collection reminder + education on importance of documentation, instructed to do what they would normally do if they were made aware of a relevant guideline | Mixed results  Clinical practice outcomes:  At 2-month follow-up, a significantly greater proportion of intervention patients compared to control patients had:   - Appropriate post-traumatic amnesia screening (117/893 (13%) vs. 12/1050 (1.1%), adjusted OR 20.1, 95%CI 6.8 to 59.3, *p* <0.001) - Post-traumatic amnesia screening tool used with them (152/893 (17%) vs. 15/1050 (1.4%), adjusted OR 19.7, 95%CI 6.6 to 58.1, *p* <0.001) - Memory-clinical assessment (303/893 (34%) vs. 272/1050 (26%), adjusted OR 1.6, 95%CI 1.2 to 2.1, *p* = 0.001)   No significant difference between intervention and control groups in CT scans for those meeting clinical criteria (352/491 (72%) vs. 337/494 (68%), adjusted OR 1.2, 95%CI 0.8 to 1.6, *p* = 0.375), total CT scans (446/893 (50%) vs. 458/1050 (44%), adjusted OR 1.2, 95%CI 0.9 to 1.6, *p* = 0.142) and provision of written patient information (160/785 (20%) vs. 175/944 (19%), adjusted OR 1.2, 95%CI 0.8 to 1.8, *p* = 0.302)  A significantly greater proportion of patients in the intervention vs. control group were:   - Safely discharged based on post-traumatic amnesia screening and provision of patient information (45/785 (6%) vs. 2/944 (0.2%), adjusted OR 27.6, 95%CI 6.9 to 110.5,   *p* < 0.001)   - Safely discharged based on post-traumatic amnesia screening, CT scan, and provision of patient information (14/402 (3.5%) vs. 0/413 (0%), OR 1.8, 95%CI 1.1 to 3.0, *p* = 0.022)   Patient outcomes:  No significant differences between intervention and control groups, respectively, in:   - Mean anxiety scores (3.4 in 125 patients vs. 4.3 in 218 patients, *p* = 0.216), - Mean post-concussion symptom (13-item Rivermead scale) scores (4.7 in 125 patients vs. 6.7 in 218 patients, *p* = 0.167) - Proportion of patients who did not return to normal activities (16/126 (13%) vs. 41/218 (19%), *p* = 0.368) - Mean quality of life scores (0.80 in 123 patients vs. 0.78 in 208 patients, *p* = 0.053)   after the intervention  Intervention group patients were more than twice as likely to re-present with brain injury symptoms compared to those in the control group (adjusted OR 1.92, 95%CI 1.08 to 3.40,  *p* = 0.026), however is a small difference of uncertain significance. |
| Chen [28]  2019  New Zealand  Adult  inpatients with urinary tract infections in a tertiary hospital | Pre- post cross-sectional study  Pre-intervention period: clinical pharmacists  identified 101 episodes of urinary tract infections in 99 patients  Intervention period: clinical pharmacists  identified 40 episodes of urinary tract infections in 40 patients | Context: Quality improvement  To determine the reasons for low guideline adherence, and to investigate the effects of a pharmacist-led, non-confrontational, inexpensive educational intervention on guideline adherence among adult inpatients with urinary tract infections | Theory: NR  Tailored to pre-identified barriers NR  Co-designed with patients or professionals: NR | | Target: Patients  Intervention: Single  Type: PROFESSIONAL: Print material (sticker)  Content: A sticker outlining the urinary tract infection antibiotic guidelines was  placed in each patient’s medical records after initiation of antibiotics for the condition. The sticker summarised the common clinical laboratory  findings in patients with asymptomatic colonisation, cystitis, uncomplicated, complicated and catheter-associated pyelonephritis according to locally agreed definitions  Format: Sticker  Delivery: Printed material (sticker)  Timing:  Pre-intervention: 30 March 2015 and 5 June 2015 (2 months);  Intervention - 1 July 2015 and 9 September 2015 (2 months)  Personnel: Pharmacists  Control: Pre-intervention period | No change  Adherence rate of initial antibiotic treatment to treatment guidelines was not significantly different in intervention and pre-intervention periods, respectively (12/33 (36%) vs 24/83 (29%) episodes, *p* = 0.5 for both)  Placing educational information  into a patient’s clinical record did not correspond with a clinically meaningful improvement in antibiotic prescribing by health professionals |
| Dhopte [29]  2019  Canada  Non-specific neck pain in Canadian  chiropractic setting | Pilot cluster RCT  32 chiropractors who had either fully or partially completed study measures overseeing 17 patients who completed study measures  Intervention: 16 chiropractors, 7 patients  Control: 16 chiropractors, 10 patients | Context: New guideline  To investigate the feasibility and impact on  protocol adherence and management of non-specific neck pain of two  methods of delivering an educational intervention:  Intervention group: A  complex knowledge-translation intervention + dissemination of practice  guidelines  Control group: Passive  dissemination of a practice guideline alone | Theory: Theoretical Domains Framework; Brief Action Planning model  Tailored to pre-identified barriers: Tailored to barriers pre-identified in telephone interviews informed by Theoretical Domains Framework; interventions developed after identifying 9 key theoretical domains (barrier analysis):  (1) Social influence  (2) Environmental context and resources  (3) Reinforcement  (4) Skills  (5) Behavioural regulation  (6) Knowledge  (7) memory, attention, and decision-making processes  (8) Social/professional role and identity  (9) Beliefs about consequences  (from study protocol, Dhopte et al., 2016)  Co-designed by patients or professionals: NR | | Target: Professionals  Intervention: Multifaceted  Type: PROFESSIONAL: Educate individuals about guideline intent/benefits + Print material (guideline)  Content: Overview of utility of guideline, key recommendation contained in new guideline on non-specific neck pain, patient self-management strategies and introduction to Brief Action Planning (from study protocol, Dhopte et al., 2016)  Format: 3 webinars, a patient-geared online self-management video based on Brief Action Planning, two online case scenarios, hard copy of practice guideline  Delivery: Online, printed material  Timing: NR  Personnel: NR  Control: Copy of a clinical practice guideline | Mixed results  Retention in study poor, clinical outcomes in very small included group showed benefit (started with 47 chiropractors who recruited 29 patients; at 3-month mark, only 32 chiropractors and 17 participants were left)    Patients in the intervention group reported significant reductions in pain (mean 1.6, 95% CI 0.26 to 2.94, *P* = 0.027) and disability scores (9.8, 95% CI 3.68 to 15.91, *P* = 0.033) from baseline to 3-month follow-up  Data gathered on self-reported knowledge and self-efficacy was limited due to the very small number of respondents to the end-of-study questionnaire  All patients who returned questionnaires on satisfaction were satisfied or very satisfied with their care |
| Dreijer [30]  2019  Netherlands  Antithrombotic stewardship | Prospective pre- post  1886 hospitalized patients  Intervention period: 945 patients  Control (usual care) period: 941 patients | Context: Quality improvement  To determine the effect of hospital-based multidisciplinary antithrombotic stewardship on adherence to anticoagulant guidelines | Theory: NR  Tailored to pre-identified barriers: NR  Co-designed with patients or professionals: NR | | Target: Professionals  Intervention: Multifaceted  Type: PROFESSIONAL:  Educate groups about guideline intent/benefits + Provide feedback from healthcare professionals;  ORGANIZATIONAL (Health professional): Create an implementation/multidisciplinary team + Communication between distant health professionals; PATIENT/CONSUMER:  Education (single) + Counselling  Content: Structured medication review at admission, patient counselling, medication reconciliation at discharge with proper transition to appropriate healthcare provider, consultation offered by multidisciplinary team, consolidation of key guideline points from other guidelines into local guideline, educating hospital staff  Format: Chart review, patient counselling, medication reconciliation, consultation, meetings, new local guideline, meetings  Delivery: In person, printed material  Timing: 9-month period of usual care with 3-month follow-up; 3-month implementation phase, 9-month post-implementation phase with 3-month follow-up  Personnel: Multidisciplinary antithrombotic team  Specialized thrombosis nurse who is case manager, hematologist, pediatric hematologist, medical leader within regional thrombotic service, hospital pharmacist/clinical pharmacologist, cardiologist, anesthesiologist, pulmonologist, neurologist,  Unique to 1^st^ study site: surgeon, quality officer  Unique to 2^nd^ study site: Optional pulmonologist, dermatologist, clinical chemist, pediatrician, emergency physician and orthopedic surgeon  Chart review by hospital pharmacist or clinical pharmacologist  Control: Usual care, hospital had existing pop-up practice alerts with respect to appropriate medication indication | Positive results  Overall adherence to antithrombotic guidelines increased significantly during the intervention period (unadjusted OR 1.76 [95% CI 1.21 to 2.05], predictor-adjusted OR 1.58 [95% CI 1.21 to 2.05])  Significant (guideline-concordant) increase in:   - Low-molecular-weight heparin prescription vs. renal function and bodyweight (OR 1.58, 95% CI 1.16 to 2.14, *p* = NR)   No difference during usual care and intervention periods in adherence to guidelines related to:   - vitamin K antagonist and interacting drugs (OR 0.82, 95% CI 0.29 to 2.36); - direct oral anticoagulant and interacting drugs, renal function, age and body weight (OR 1.04, 95% CI 0.50 to 2.15); - pre-operative International Normalized Ratio values (OR 1.31, 95% CI 0.80 to 2.18) |
| Gulayin [31]  2019  Argentina  Statin use for hypercholesterolemia in patients at medium to high risk for cardiovascular disease | Cluster RCT  357 primary care physicians from 10 primary care centres from the public healthcare system  Intervention group: 179 physicians  Control group: 178 physicians | Context: Quality improvement  To test whether a multifaceted educational intervention improves treatment and control of hypercholesterolemia in low-income primary care settings | Theory: Chronic Care Model  Barriers pre-identified in the literature pertaining to the management of hypercholesterolemia include:   - Organizational barriers in primary care - Confusing/conflicting guidelines - Errors and omissions by primary care physicians - Communication problems between primary and secondary care   Healthcare-level barriers:   - Lack of access to medications - Cost of medications - Poor insurance coverage   Provider-level barriers:   - Non-adherence to guidelines - Willingness to accept high cholesterol - Failure to prioritize hypercholesterolemia in the midst of multiple other chronic medical issues   Patient-level barrier:   - Reluctance to take medication   (Hickling et al., 2005)  Pre-identified barriers pertaining to preventative care at the primary care level include:   - Multiple demands on physician times - Lack of reimbursement for counselling   (Jaén et al., 1994)  Tailored to pre-identified barriers: NR  Co-designed by professionals:  The content of the educational outreach visits (which were one component of the intervention) were tailored to practitioner-identified barriers to appropriate statin prescribing, but barriers were not identified prior to the start of the intervention (not identified prior to training workshops) | | Target: Patients and professionals  Intervention: Multifaceted  Type: PROFESSIONAL: Present guideline materials at meetings + Educate groups about guideline intent/benefits + Provide feedback on compliance + Print material (educational flyers); PATIENT: Education + Reminder; FINANCIAL (Health professional): Grant or allowance to group/institution (not tied to compliance); STRUCTURAL CHANGES: Information/communication technology  Content: Training workshop: Education on cardiovascular risk assessment and management; diagnosis, treatment and monitoring of patients with dyslipidemia, components of chronic care model, how to tackle adherence issues in patients with chronic diseases  Educational outreach visits: practice exercises, prescription audit and feedback, recommendations to improve practice administration, on-site training for pharmacist assistant at first visit  mHealth application: Decision-support smartphone tool  Patient text messages: Encouragement to adopt healthy lifestyles, visit care provider, adhere to statin and other medication usage  Format: Training workshop, educational outreach visits, smartphone application, text messages  Delivery: In-person, SMS text messages  Timing:  Intervention over 1 year  2-day training workshop,  3 educational outreach visits at 3 months, 6 months, 9 months  Personnel: Research nurses  Control: Usual care | Mixed results  No adjusted net differences (compared to baseline) between intervention and control groups in cholesterol levels at the 6-month mark (-6.0, 95%CI -15.6 to 3.6, *p* = 0.2224) or 12-month mark (-0.8, 95%CI -15.6 to 14.1, *p* = 0.9207); or in cardiovascular disease risk scores at the 12-month mark (-1.5, 95%CI -4.3 to 1.2, *p* = 0.2802).  However, there was a significant difference in favour of the intervention group at the 6-month mark in adjusted net differences (compared to baseline) in cardiovascular disease risk scores (-4.0, 95%CI -6.5 to -1.5, *p* = 0.0019).  A significantly higher proportion of participants were receiving an appropriate statin dose in the intervention group at both 6-month (37.1%, *p* = 0.0007, adjusted 28.8%, *p* = 0.0031) and 12-month (41.5%, *p* = 0.0001, adjusted 38.5%, *p* < 0.0001) measurement points |
| Huang [32]  2019  USA  Opioid prescribing and monitoring for chronic pain in primary care | Retrospective, pre- post  519 patients with chronic pain requiring opioids, overseen by 35 primary care providers | Context: Quality improvement  To evaluate the impact of a multicomponent, team-based opioid management system with electronic health record support on adherence to chronic opioid prescribing and monitoring guidelines | Theory: NR  Barriers to the safe and effective management of chronic pain pre-identified in the literature were:   - Time - Resources   (Krebs et al., 2011; Khodaee and Deffenbacher, 2016; Lin et al., 2017; Rutkow et al., 2015; Irvine et al., 2014)  Tailored to pre-identified barriers: NR  Co-designed by patients or professionals: NR | | Target: Professionals  Intervention: Multifaceted  Type: PROFESSIONAL: Provide feedback on compliance + Provide feedback about patients (outcome data) + Provide feedback from healthcare professionals; STRUCTURAL CHANGES: Information/communication technology + Quality improvement, performance management system (risk-assessment algorithm); ORGANIZATIONAL (Health professional): Create implementation/ multidisciplinary team  Content: Patient registry with reminders for primary care providers; Standardization of chronic opioid-prescribing policies; Development of a risk-assessment algorithm to assess opioid misuse; Team-based case management; Electronic health record dashboard tool with fields for communication of anomalies and nonadherence, containing streamlined access to ordering tests, patient agreements, protocols, and records  Format: Online hospital system, new protocol, risk-assessment tool, case management, electronic tool  Delivery: In person, online  Timing: 1 year (September 2015-September 2016)  Personnel: 2 physician assistants added to implementation team to serve as population health specialists and case managers  Control: NR | Positive results  Increased adherence to opioid management guidelines  Significant improvement in the following from pre-intervention  (n = 519) to post-intervention  (n = 480):   - Percentage of patients on chronic opioid therapy (1.6% to 1.3%, *p* = 0.01) - Average number of visits/year for patients on chronic opioid therapy (4.1 (SD 3.6) to 6.0 (SD 4.1), *p* < 0.0001) - Percentage of patients on chronic opioid therapy with no primary care visit in the past year (9% to 0.2%,   *p* < 0.0001)   - Percentage of patients on chronic opioid therapy with a signed controlled substance agreement in past year (46% to 76%, *p* < 0.0001) - Percentage of patients on chronic opioid therapy with no controlled substance agreement on file ever (13% to 3%, *p* < 0.0001) - Percentage of patients with urine drug screen in the past year and the past 6 months (29% to 73%, *p* < 0.0001) - Percentage of patients checked by clinician using the state prescription monitoring programs in past year (45% to 97%, *p* < 0.0001) and in the past 60 days (38% to 63%,   *p* < 0.0001)  No significant differences in:   - Average number of patients on chronic opioid therapy per attending primary care provider (14.2 (SD 12.8 vs. 13.1 (SD 12.0), *p* = 0.72) - Percentage of patients on ≥ 90mg morphine-equivalent dose/day (32% vs. 29%, *p* = 0.37) |
| Jolliffe [33]  2019  Australia  Brain injury rehabilitation | Pre- post  58 medical, nursing and allied health staff in a stroke unit | Context: Quality improvement  To evaluate the impact of fortnightly audit and feedback  cycles on adherence to acquired brain injury rehabilitation guidelines | Theory: Theoretical Domains Framework, Promoting Action on Research Implementation (PARiHS) framework,  Template for Intervention Description and Replication - TIDieR  Tailored to pre-identified barriers: NR  The intervention was tailored to organization (method NR)  Co-designed by patients or professionals: NR | | Target: Professionals  Intervention: Multifaceted  Type: PROFESSIONAL: Present guideline materials at meetings (general and tailored to the audit finding) + Distribute guideline material + Provide feedback on compliance + Provide reminders to groups about intent/benefits;  Content: Audit and feedback within usual clinical meetings, guidelines, educational summary of guidelines in poster  Format: Electronic guideline, Electronic and hard copy poster, face-to-face (group) feedback presentation, face-to-face or email (individual) feedback observational audits  Delivery: In-person, online, printed material  Timing: September 2014 and March 2016, Fortnightly (every 2 weeks) cycles of audit and feedback for 14 months  Personnel: Project manager, occupational therapist, research assistant | Positive results  Audit and feedback program led to a significant increase in adherence to clinical practice guideline recommendations  There was a significant increase in median CPG indicator adherence at the post-intervention mark (13-15 months) vs. at baseline (0-2 months): increase of 45.2, 95%CI 38.5, 50.3, *p* = 0.0001  However, there was also a significant decrease in the median CPG indicator adherence at follow up (18-19 months) vs. at the post-intervention mark (13-15 months): decrease of 7.0, 95%CI -0.5, -14.0, *p* = 0.0102  Adherence rates decreased at follow-up, but they were still significantly different from baseline levels of guideline adherence  Model of care indicators and other guideline recommendations were also carefully audited by the study’s authors |
| Lee [34]  2019  Taiwan  Medication adherence after acute coronary syndrome | Retrospective, cross-sectional pre- post    282 patients with acute coronary syndrome  Pre-intervention: 76 patients  Post-intervention: 202 patients | Context: Quality improvement  To evaluate the effects of reinforcement of patient and family education on the usage of  guideline-recommended secondary prevention medications for patients who have had a heart attack | Theory: NR  Tailored to pre-identified barriers: NR  Co-designed by patients or professionals: NR | | Target: Patients  Intervention: Single  Type: STRUCTURAL CHANGES: Information/communication technology  Content:  Symptoms, risk factors, exercise, smoking cessation, diet, evidence-based medications + purpose, directions, interactions and adverse effects of guideline-indicated medications  Format: Patient and family education program embedded in hospital information system  Delivery: Online  Timing: February 2015 to April 2017, post-implementation - after January 4, 2016  Personnel: Trained study coordinator  Control: Pre-intervention – no educational information/ standard or care | Positive results  Reinforcement of patient education was associated with significant improvements in patient medication adherence  Patient adherence to prescribed medication post vs. pre-intervention:   - All drugs (39.32% vs. 14.47%, *p* < 0.001) - Antiplatelet therapy combined with aspirin (79.6% vs. 47.37%, *p* < 0.001) - Statins (74.76% vs. 34.21%, *p* < 0.001) - Beta-blockers (81.07% vs. 46.05%, *p* < 0.001) - Antiplatelet therapy combined with aspirin (79.6% vs. 47.37%, *p* < 0.001) - ACE inhibitors/ARBs (62.62% vs. 38.16%, *p* < 0.001) |
| Marcial [35]  2019  USA  Diabetes care and outcomes in primary care | Pre- post-  50 multidisciplinary primary care providers with approximately 1500 patients at post-intervention point | Context: Quality improvement + New guideline (new additions to existing guideline made in 2017)  To determine whether educational intervention improved provider attitudes and knowledge of guidelines, and clinical outcomes in Hispanic-origin patients with diabetes | Theory: Chronic Care Model  Tailored to pre-identified barriers:  Barriers pre-identified in the literature pertaining to diabetes include:   - Patient and provider relationships - Time-consuming delivery of proper care - Impractical CPGs - Primary care provider attitudes towards using CPG   (Johnson et al., 2015)  Barriers pre-identified in the literature pertaining to diabetes care in minority groups is:   - Low education - Language barriers - Poor literacy - Low acculturation - Cultural values and beliefs like machismo and fatalism   (Greenwood et al., 2015)  The guideline itself lists the following as barriers:   - Provider lack of knowledge on care across cultures - Provider lack of general cultural knowledge   (American Diabetes Association, 2017)  Barriers identified by the study team through anecdote were:   - Lack of Medicare reimbursement for diabetes-related hospitalizations - Absence of health education for patients - Provider-reported lack of time to provide patient education   Barriers identified by the study team through the Diabetes Attitude Survey and electronic clinical quality measures were:   - Provider lack of knowledge regarding diabetes management - Poor attitude and lack of knowledge among providers with respect to diabetes management - Lack of communication among caregivers - Self-management instructions not given by provider - Family members not involved as care partners   The educational intervention was tailored to address the barriers identified through the Diabetes Attitude Survey and electronic clinical quality measures administered by the study team  Co-designed with patients or professionals: NR | | Target: Professionals  Intervention: Single  Type: PROFESSIONAL: Present guideline material at meetings + Educate groups about guideline intent/benefits  Content: Education about importance of adhering to guidelines, diabetes self-management, lifestyle changes for patients, resources available to patients within community  Format: Education session  Delivery: In person  Timing: 2-hour education session  Personnel: NR, presumed project director (qualifications NR) | Positive impact  Post-intervention improvement in provider knowledge and attitudes as well as patient outcomes  Diabetes Attitude Scale: Significant improvements in all five subscale scores pre- vs. post-intervention, *p* < 0.001 for all:   - Patient autonomy (2.66 vs. 2.96) - Psychosocial impact of diabetes mellitus (2.24 vs. 3.04) - Value of tight control (2.64 vs. 2.98) - Seriousness of diabetes (2.51 vs. 2.85) - Need for special training (2.66 vs. 3.10)   19 measures of patient care quality were assessed, including measurable patient-related problems, new guideline standards, and measurable provider-related problems  Averages of aggregate care quality scores were significantly differently in the pre- vs. post-intervention periods: 20.53 vs. 64.32 (t = 9.31, *p* < 0.001) |
| McAdam-Marx [36]  2019  USA  Pneumococcal vaccination rates in primary care | Retrospective study using cross-sectional and historical cohorts  Patients were part 3 clinic groups: Family Medicine Clinics Group A (FM Group A), Family Medicine Clinics Group B (FM Group B), or Internal Medicine Clinics Group C (IM Group C)  Those included aged 19-64:  Baseline period: 18851patients  Interim period: 21872 patients  Follow-up: 25110 patients  Of these, those *at high risk* (possibly immunocompromised and vaccine-naïve):  Baseline period: 2658 (14%)  Interim period: 3449 (16%)  Follow-up period: 3612 (14%)  Remainder of those from each period aged 19-64 were *at risk* (with certain chronic conditions):  Baseline period: 16193  Interim period: 18423  Follow-up period: 21498  Adults 65+ included:  Baseline period: 12057 patients  Interim period: 14518 patients  Follow-up period: 16689 patients    *Immunocompetent adults aged 65+*: Baseline period: 9480  Interim period: 11318  Follow-up period: 13341  *Immunocompromised adults aged 65+*:  Baseline period: 2577  Interim period: 3200  Follow-up period: 3348 | Context: Quality improvement  To assess the impact of  health maintenance notifications and best practice alerts, with and without workflow redesign, on  pneumococcal vaccination rates in 4 categories of adults with varying degrees of pneumococcus susceptibility | Theory: NR  Tailored to pre-identified barriers: NR  Co-designed with professionals through process mapping and workflow redesign: The workflow redesign component of the intervention was undertaken by clinics themselves | | Target: Professionals  Intervention: Multifaceted  Type: PROFESSIONAL: Distribute guideline material; STRUCTURAL CHANGES: Organizational structure (including reorganization) + Information/communication technology  Content:   1. Workflow redesign: A clinic-specific process mapping and redesign to determine the best point at which to introduce the decision support tool, and adjustment of workflows accordingly 2. Health maintenance notification: Notification prompts staff to discuss pneumococcal vaccination with patient, and contains links to guidelines and order sets 3. Best practice alert: Follow-through reminder after health maintenance notification   Format: Workflow redesign, health maintenance notification, best practice alert  Delivery: In person, electronic medical record  Timing:  Baseline period  August 1, 2013-July 31, 2014  All 3 groups: Practice as usual  Interim period  May 1, 2015-April 30, 2016  FM Group A: Clinical workflow redesign  FM Group B and  IM Group C: Practice as usual  Follow-up period  May 1, 2016-July 31, 2017  FM Group A: Clinical workflow redesign + health maintenance notification + best practice alert  FM Group B: Health maintenance notification + best practice alert  IM Group C: Health maintenance notification  Personnel: NR  Control: Health maintenance notifications only | Mixed results  The best practice alert both with and without workflow redesign improved pneumococcal vaccination rates for *high-risk adults aged 19-64*, increased likelihood that *immunocompromised adults aged 65+* would receive their 1^st^ dose of a 2-dose series and that *immunocompetent adults aged 65+* would receive their 2^nd^ dose, but no across-the-board improvements among other adult groups for 1^st^ or 2^nd^ dose of the vaccine  Significant differences in proportion of vaccine-eligible *high-risk adults aged 19-64* who received 1st dose of vaccine in **all 3 periods**, among all 3 groups (FM Group A, FM Group B, IM Group C) (*p* < 0.0001 for all)  Significant differences in proportion of vaccine-eligible *high-risk adults aged 19-64* who received 2nd dose of vaccine in **all 3 periods**, among all 3 groups (FM Group A: *p* < 0.0001, FM Group B: *p* < 0.0004, IM Group C: *p* < 0.0005)  Significant differences in proportion of vaccine-eligible *immunocompetent adults aged 65+* who received 2nd dose of vaccine only in **all 3 periods**, among all 3 groups (FM Group A, FM Group B, IM Group C) (*p* < 0.0001 for all)  Significant differences in proportion of vaccine-eligible *immunocompetent adults aged 65+* who received 1st dose of vaccine between the **interim and follow-up periods** in IM Group C only, *p* < 0.0001  Significant differences in proportion of vaccine-eligible *immunocompromised adults aged 65+* who received 1st dose of vaccine only in **all 3 periods**, among all 3 groups (FM Group A, FM Group B, IM Group C) (*p* < 0.0001 for all)  Significant differences in proportion of vaccine-eligible *immunocompromised adults aged 65+* who received 2nd dose of vaccine in **all 3 periods** in IM Group C only, *p* < 0.0011 |
| Moseng [37]  2019  Norway  Osteoarthritis in primary care | Stepped-wedge cluster RCT  6 municipalities (clusters) comprising  40 GPs and 37 physiotherapists treating 393 patients with hip and/or knee osteoarthritis aged ≥ 45 years  Patients recruited during intervention phase: 284 patients  Patients recruited during control phase: 109 patients | Context: Quality improvement  To assess the feasibility and costs of a tailored strategy for osteoarthritic care, and to assess the strategy’s impact on the uptake of core guideline recommendations | Theory: NR  Barriers were pre-identified through 3 focus groups with 8 GPs, 6 physiotherapists, and 3 patient representatives (Østeras et al., 2015). Barriers were then classified into themes.  Themes pertaining to all three groups were:   - Awareness and knowledge   Themes pertaining to GPs and physiotherapists were:   - Motivation to change - Attitude - Behavioural routines   Themes pertaining to GPs and patients only were:   - Accessibility/availability   The intervention was tailored to all pre-identified barrier themes  Co-designed by patients or professionals: NR | | Target: Patients and professionals  Intervention: Multifaceted  Type: PROFESSIONAL: Present guideline material at meetings (physiotherapists and GPs) + Educate groups about guideline intent/benefits (physiotherapists and GPs) + Print material (GP guideline summary & written information; physiotherapist guideline summary & patient education program manuscript) + Provide reminders to groups about intent/benefits (stationery with reminders); PATIENT/CONSUMER: Education + Print material (educational osteoarthritis booklet) + Reminder (informational booklet, checklist, exercise diary); STRUCTURAL CHANGES: Method of service delivery  Content:  Physiotherapist-geared:   - Workshop: - Update on osteoarthritis, clinical features, recommended treatment, provision of individualized care delivery - Education in delivery of patient-geared: education program, exercise intervention, performance tests - Print material: - Summary of international guidelines for non-pharmacological osteoarthritis care   GP-geared:   - Workshop: - Recommended osteoarthritis care, information on physiotherapist treatment program, appropriate time for surgical referral - Print material: - Written information on osteoarthritis care - Summary of international guidelines for osteoarthritis care   Patient-geared:   - Educational sessions and exercise groups - Written material + reminders - Information about osteoarthritis, treatment, and self-management - Checklist of recommended care items - Exercise diary to track exercise   Format: Multidisciplinary workshops, Powerpoint presentation, manuscript, guideline summaries, written information on treatment, booklets, exercise diaries, stationery reminders, patient education sessions, exercise groups  Delivery: In person, PowerPoint presentation, printed material  Timing:  1.5-hour GP workshop, 1-day physiotherapist workshop, 3-hour group-based patient education sessions, 8 -12 weeks of 2x/week individual exercise and 2x/week group exercise for patients  Personnel: Those who delivered GP- and physio-geared interventions NR; Patient-geared interventions: GPs and physiotherapists  Control: Same staffing, no intervention | Positive results  Study evaluated fidelity to implementation strategy and uptake of recommendations – only the latter is reported below  Significantly greater uptake of all treatment components (information, exercise and weight management) in intervention phase as measured by patient report (adjusted OR [95% CI], *P*-value):   - Participation in patient education: 82.2 [24.6 to 274.7], *P* < 0.001 - Received information on osteoarthritis: 3.5 [1.6 to 8.0], *P* < 0.05 - Resistance exercise performed: 5.0 [2.1 to 12.3],   *P* < 0.001   - Cardiorespiratory exercise performed: 5.7 [2.5 to 13.1],   *P* < 0.001   - Referral to support services for weight management: crude OR 3.5 [1.3 to 9.5], *P* < 0.05   Significantly more control phase patients received only passive exercise treatments: crude OR 0.05 [95% CI 0.01 to 0.4], *P* < 0.05  Physiotherapist knowledge: ≥90% of physiotherapists answered in line with treatment recommendations on 7/8 questions  Physiotherapist attitudes were also measured |
| Orchard [38]  2019  Australia  Atrial fibrillation screening and proper medical management in general practice | Cross-sectional, pre-post  30 general practitioners and 16 nursing staff screened 1805 patients | Context: Quality improvement  To examine the impact of electronic prompts in promoting opportunistic atrial fibrillation screening, and electronic decision support on the treatment of atrial fibrillation | Theory: NR  Barriers to guideline adherence, and to using the smartphone app were pre-identified in the pilot studies corresponding to this paper (Orchard et al., 2014; Orchard et al., 2016). Barriers were determined through semi-structured in-person interviews with GPs, practice nurses, practice managers, and patients, which were then compiled into a barrier and enabler analysis.  Important barriers identified included:   - Lack of remuneration for using smartphone app - Lack of time during flu vaccination (atrial fibrillation screening was implemented during flu shot season) - The need for a reminder prompt for screening - Gaps in evidence-based treatment for those with atrial fibrillation   Study was tailored to pre-identified barriers  Co-designed with professionals through the semi-structured interviews involved in tailoring, as many of the barriers and enablers identified were heavily relied upon to inform the development of the final smartphone app | | Target: Professionals  Intervention: Multifaceted  Type: FINANCIAL (Health professional): Incentive (group or institutional financial reward or benefit) – (AUD$1000 to cover setup, $10/patient for first 500 patients screened); STRUCTURAL CHANGES: Information/communication technology  Content:  Atrial fibrillation screening app; prompt incorporated into electronic medical record; treatment algorithm built into yet another software that provider cardiovascular decision support  Format: Smartphone screening app, Pop-up prompt, Electronic decision support  Delivery: Online, electronic health record  Timing: 2-12 month screening period  Personnel: NR | Mixed results  97% of those diagnosed with atrial fibrillation during the study and those diagnosed before the study needed oral anticoagulants  Significantly more of those diagnosed with atrial fibrillation during the study vs. before the study (both screen-detected and clinically detected) were actually prescribed oral anticoagulants: 85% (54/64) vs. 71% (933/1306), *P* < 0.001, respectively  No significant difference between those diagnosed before vs. during the study in proportion inappropriately prescribed antiplatelet medication only (213 (16%) vs. 3 (5%), *p* = NR), or those given no therapy (160 (12% vs. 7 (11%), *p* = NR)  Adherence to guidelines was higher in patients during the study period but the decision support software was not used in the majority of patients. |
| O’Sullivan [39]  2019  USA  Perioperative prophylactic antibiotic use | Quasi-experimental interrupted time series  Anesthesia providers, surgeons, nurses, pharmacists who work regularly in the operating room; n = NR  13695 surgical procedures evaluated | Context: Quality improvement  To determine the impact of a multifaceted intervention on adherence to perioperative prophylactic antibiotic re-dosing recommendations for surgical procedures lasting more than 4 hours | Theory: NR  Tailored to pre-identified barriers: NR  Co-designed by patients or professionals: NR | | Target: Professionals  Intervention: Multifaceted  Type: PROFESSIONAL: Tailor guideline + Print material + Educate groups about guideline intent/benefits + Provide reminders to individuals/groups about intent/benefits + Provide feedback on compliance + Provide feedback from healthcare professionals; STRUCTURAL CHANGES: Quality improvement, performance measurement system + Information/communication technology  Content: Information about antibiotic re-dosing, reminder before re-dosing time, electronic and hard copy guide made available  Format: Educational sessions; electronic and print versions of antibiotic administration guide; electronic alerts in hospital electronic health record system  Delivery: In person, electronic health record, print material  Timing: Electronic health record reminders delivered at 15 minutes prior to scheduled antibiotic re-dosing time; educational presentations within 4-month window  Personnel: Anesthesia and pharmacy providers | Positive impact  At the end of the multifaceted intervention, appropriate re-dosing rates had increased significantly with compliance at ~80% and inappropriate re-dosing of antibiotics without apparent justification was reduced from ~17% prior to the multifaceted intervention to ~1.2%  Improving trend in antibiotic re-dosing prior to intervention (incidence rate ratio, [95% CI]):  1.004 per week [1.003-1.004],  *P* < 0.001)  At the end of intervention, further significant increase in appropriate re-dosing rates (incidence rate ratio, [95% CI]): 1.158 [1.056–1.268]; *P* = 0.002)  No further significant change observed after the intervention |
| Takaesu [40]  2019  Japan  Treatment  guidelines for schizophrenia and major depressive disorder | Pre- post  344 psychiatrists who had attended ≥1 educational session in the program | Context: Quality improvement  To evaluate the effect of a one-day educational program on knowledge of treatment guidelines for depression and schizophrenia | Theory: NR  Tailored to pre-identified barriers NR  Co-designed by patients or professionals: NR | | Target: Professionals  Intervention: Single  Type: PROFESSIONAL: Present guideline materials at meetings + Educate groups about guideline intent/benefits  Content: Educational program on the treatment guidelines  Format: Lectures, discussion of 2 cases  Delivery: In person  Timing: 1-day educational program,  Knowledge assessment retaken at the end of the day-long program  Educational programs ran from October 2016-March 2018  Personnel: NR  Control: Baseline | Positive effect  All measures of knowledge of the guidelines was significantly improved  after the programs for schizophrenia (7 knowledge measures) and major depressive disorder (8 knowledge measures)  Schizophrenia guideline, Average % correct answers at baseline and post-program:  Total clinical knowledge: 90.3% vs. 98.1% (*P* = 5.3 x 10^-51^)  Depression guideline, Average % correct answers at baseline and post-program:  Total clinical knowledge: 84.4% vs. 93.5% (*P* = 1.3 x 10^-52^) |
| Wilkins [41]  2019  New Zealand  Appropriate pharmacotherapy following myocardial infarction | Pre- post  Physician prescribers: number NR  Pre-intervention group: 100 patients  Intervention group: 477 patients | Context: Quality improvement  To assess if the introduction of a pre-discharge medication checklist improved adherence to myocardial infarction treatment guidelines | Theory: NR  Tailored to pre-identified barriers: NR  Co-designed with patients or professionals: NR | | Target: Professionals  Intervention: Single  Type: STRUCTURAL CHANGES: Quality improvement, performance measurement system  Content: Recommended medications with contraindications for prescribing  Format: Checklist  Delivery: Printed material  Timing: Before patient discharge  Pre-intervention: May-September 2014  Intervention: September 2014-July 2015  Personnel: Two physicians ensured checklist was completed | Positive impact  The introduction of the pre-discharge medication checklist led to significant improvements in all five guideline-recommended secondary prevention measures:  Significant differences in adherence to guideline-recommended prescribing of:   - Aspirin: (90% vs. 96.6%, *P* = 0.004) - ADP receptor antagonist: (84% vs. 96.4%, *P* = 0.0001) - Beta blocker: (79% vs. 87.3%, *P* = 0.03) - Statin: (88% vs. 96.0%, *P* = 0.002) - ACE inhibitor/ARB: (58% vs. 69.6%, *P* = 0.03)   pre- (n = 100) vs. post-  (n = 447) implementation of pre-discharge medication checklist |
| Carter [42]  2018  USA  Proper management of cardiovascular disease, cardiovascular disease metrics in patients; cancer prevention and immunizations | Cluster RCT  12 family medicine offices without pharmacists in rural/small communities serving 259 patients with at least 3 cardiovascular disease states or risk factors  Intervention group (6 family medicine offices with 128 patients)  Control group (6 family medicine offices with 134 patients) | Context: Quality improvement  To determine whether a centralized, web-based, pharmacist-managed cardiovascular risk service will improve adherence to cardiovascular treatment guidelines and risk factor control in primary care | Theory: Physician-pharmacist collaborative model (Carter et al., 2015; Gums et al., 2015) (with remote pharmacist as the novel feature in this intervention)  Tailored to pre-identified barriers: NR  Co-designed with patients or professionals: NR | | Target: Patients AND professionals  Intervention: Multifaceted  Type: PROFESSIONAL: Provide alerts when practice deviates;  PATIENT/CONSUMER: Education (single); FINANCIAL (Patient): Grant or allowance (not tied to compliance);  ORGANIZATIONAL (Health professional): Additional human resources (3 off-site pharmacists)  Content: Pharmacist identification and addressing of problems leading to poor disease control, patient education related to diet, physical activity, and alcohol and tobacco use; Pharmacist monitoring of:   1. various laboratory values and cardiovascular-disease-specific readings 2. recommendations and/or tests ordered based on a)   Patient education  Format: Patient education when required by telephone; Monitoring through electronic medical record access  -notes, tests and medication changes made directly in 4 offices’ electronic medical record (with physician consent)  -recommendations by fax in 2 offices  Delivery: telephone; online, fax  Timing: Patient education every 1-2 weeks and, with gradual resolution, every 1-2 months; Real-time pharmacist recommendations (if office allowed), or fax recommendations over 12-month course of study  Personnel: Experienced pharmacists, family physicians; Nurse practitioners or physician assistants as study coordinators  Control: Usual care | Positive impact  A score based on adherence to cardiovascular-disease-prevention guidelines and cardiovascular disease metrics was computed for the control group and intervention group, at baseline and at 12 months  Control group: No change in score from baseline to 12 months (64.7% to 63.1%, *p* = 0.21)  Intervention group: Significant improvement in score from 63.3% to 67.8% at baseline and 12 months, respectively (*p* = 0.02)  Benefit of intervention estimated at 5.0%±2.4% (95%CI -0.5% to 10.4%; *p* = 0.07)  12 metrics and areas identified with scope for pharmacist recommendations  No significant differences between intervention and control groups at baseline and 12 months in:  i) Recommendation areas:   1. Appropriate blood pressure treatment (*p* = 0.56) 2. Symptom and activity assessment (*p* = 0.21) 3. Rate of advising smokers to quit (*p* = 0.22) 4. Tobacco replacement therapy recommended (*p* = 0.18) 5. Dilated eye examination recommended (*p* = 0.38) 6. Diabetic foot examination recommended (*p* = 0.80) 7. Microalbumin order recommended (*p* = 0.17)   ii) Metrics:   1. Uncontrolled hypertension (*p* = 0.97) 2. Uncontrolled diabetes mellitus (*p* = 0.10)   Significant difference in intervention and control groups at baseline and 12 months in:   1. Screening for alcohol use (*p* =0.0008) 2. Appropriate statin recommended (*p* < 0.001) 3. Body mass index: screening and follow-up (*p* < 0.001) |
| Dodek [43]  2018  Canada  Glucose control in critically ill (those receiving intravenous insulin) | Observational study  272 clinician members in a ‘community of practice’ from 16 intensive care units | Context: Quality improvement  To describe the development, implementation and initial evaluation of a glucose control improvement initiative targeted to critical care clinicians within an entire Canadian province | Theory: NR  Tailored to pre-identified barriers: NR  Co-designed by professionals: The ‘community of practice’ was a multidisciplinary stakeholder group consisting of dietitians, pharmacists, nurses, respiratory therapists and physicians who were actively involved in implementing changes, and sharing quality metrics, tools and resources | | Target: Professionals  Intervention: Multifaceted  Type: PROFESSIONAL: Educate individuals about guideline intent/benefits + Educate groups about guideline intent/benefits +  Present guideline materials at meetings;  ORGANIZATIONAL: Create an implementation/multidisciplinary team  Content: A provincial healthcare council standardized glycemic control guidelines in nine high-priority areas; engaged stakeholders through virtual meetings, electronic newsletters, and online tool for the community of practice to share resources and have discussions; glucose control website with protocols and other resources; two virtual learning sessions; held a conference to share strategies and discuss issues; during data collection, new tools and methods of data collection  Format: Virtual meetings, electronic newsletters, online tool, website, virtual learning sessions, conference, data collections changes included flowsheets, sliding scale orders, protocols, staff education, introduction of new instruments  Delivery: In person, online  Timing: Intervention from 2011-2013  Data collection from April 2012-April 2016  Personnel: Critical Care Working Group, a multidisciplinary group of experts + clinical and quality leads from a provincial healthcare council + local critical care physician to provide provincial leadership in initiative | No change  No major trends over time in glucose control and no association with any process changes for each hospital.  Paper does not report any statistics  Lack of sustained improvement suggests the need for more active and durable interventions. |
| Dziedzic [44]  2018  UK  Osteoarthritis in primary care | Cluster RCT  8 practices with 525 adults > 45 years with peripheral joint pain  Intervention group: 4 practices with 288 adults enrolled  Control group: 4 practices with 237 adults enrolled | Context: Quality improvement  To ascertain the effect of new mode of osteoarthritis consultation and patient-geared guidebook on a primary patient osteoarthritis outcome measures, uptake of guideline-recommended treatments and self-management, and other secondary outcome measures | Theory: Whole Systems Informing Self-Management Engagement (WISE) Model, Behaviour Change Wheel, Theoretical Domains Framework, Normalisation Process Theory (from study protocol Dziedzic et al. 2014), Calgary-Cambridge Framework (from study protocol Dziedzic et al. 2014), Grol’s approach to translating evidence to practice (from study protocol Dziedzic et al. 2014; Grol, 1997)  Tailored to pre-identified barriers: NR  The intervention itself was co-designed by patients and professionals through consensus rounds on what they considered important elements of an osteoarthritis consultation  Lay advisors from a local arthritis support group and a Research User Group (composed of people who have chronic musculoskeletal conditions) co-designed the osteoarthritis guidebook that formed part of the intervention, through 3 group discussions (Grime and Dudley, 2014) | | Target: Patients and professionals  Intervention: Multifaceted  Type: PROFESSIONAL: Distribute guideline material; PATIENT: Education + Counselling + Print material (guidebook); STRUCTURAL CHANGES: Method of service delivery  Content:  Patient-geared:  Explanation of osteoarthritis in layperson language, prescription of first-line analgesics as appropriate, support for self-management, promotion of core treatments, accounts of how other people live with osteoarthritis, provision of guidebook; explanation of guidebook, negotiation of patient-specific goals, discussion of other lifestyle changes  General practitioner and nurse-geared:  Practice updates on diagnosis, advice, patient self-care, overview of guidebook  Format: Enhanced consultations, practice-based sessions, training sessions, guidebook  Delivery: In person, printed material  Timing: 6-month run-in period  General-practitioner-geared: 4 practice-based sessions (Three 2-hour and one 1-hour sessions)  Nurse-geared: 4 training days  Patient consultation with nurse within 3 months of general practitioner consultation  Personnel: Study coordinators  Control: No training, guidebook or dedicated nurse-led osteoarthritis clinic | Mixed results   - Clinical effectiveness: No significant mean differences between intervention and control practices in primary outcome measure: physical outcome score of patients at:   🡪 3 months (-0.29 (95% CI -1.86, 1.29), effect size -0.03 (95% CI -0.17, 0.11), *p* = 0.722);  🡪 6 months (-0.37 (95% CI -2.32, 1.57), effect size -0.03 (95%CI -0.21, 0.14) *p* = 0.706;  🡪 or 12 months (-0.90 (95%CI -3.75, 1.96), effect size -0.08 (95%CI -0.33, 0.17), *p* = 0.539)   - Uptake of guideline recommendations and self-management:   Significant (guideline-concordant) differences pre-intervention and at 6 months in intervention vs. control practices (% difference [95% CI], *p*-value) in:  Some treatments offered by providers (self-reported):   - Information/advice about exercises: 20.5% [7.9%, 28.3%], *p* = 0.004 - Paracetamol for pain: 10.7% [0.6%, 20.7%], *p* = 0.037   Some treatments used by patients (self-reported; % difference [95% CI], *p*-value):   - Education, advice and access to information: 23.2% [11.9%, 32.4%], *p* < 0.001 - Muscle-strengthening exercises: 16.0% [4.4%, 27.5%], *p* = 0.007 - Use of oral NSAIDs: -15.6% [-28.3%, -3.5%], *p* = 0.010 - Reliance on walking aids: -13.9% [-24.6%, -1.6%], *p* = 0.027 - Community pharmacy: 10.2% [0.3%, 23.3%], *p* = 0.043   No differences in 11 other self-reported treatments offered by providers  No differences in 12 other self-reported treatments used by patients   - No differences between intervention and control group pre-intervention and at 6 months in all but two (of 13) secondary outcome measures: mean difference in physical activity for the elderly scores (-18.3 [95% CI -34.0, -2.6], effect size -0.23 [95% CI -0.42, -0.03], *p* = 0.022); mean difference in patient enablement score (1.34 [95% CI 0.59, 2.10), effect size 0.42 [95% CI 0.18, 0.65], *p* < 0.001) in favour of the intervention group |
| Etxeberria [45]  2018  Spain  Type 2 diabetes, hypertension, and dyslipidemia management | Cluster RCT  448 family physicians in 43 primary care units (clusters)  Intervention group: 242 family physicians in 21 units overseeing  20832 patients with diabetes and 57369 patients with hypertension  Control group: 217 family physicians in 22 units overseeing 19588 patients with diabetes and 51985 patients with hypertension  Nurses, n=NR | Context: Quality improvement  To evaluate the effectiveness of a multifaceted tailored intervention in the implementation of 3 cardiovascular-risk-related guidelines in primary care compared to usual implementation methods | Theory: Grol’s 10-step model (Grol et al., 2005), which brings together elements from diffusion of innovation theory, reasoned action theory, and social cognitive theory (from study protocol, Etxeberria et al., 2013)  Study protocol (Etxeberria et al., 2013) mentions barriers to and facilitators of guideline implementation identified in previous study (Pérez et al., 2009) by the study team using the Delphi technique. The pre-identified barriers occurred at different levels and were both internal and external (Etxeberria et al., 2013):  Level: Presentation of the guidelines:   - The teaching method used by speakers - Choice of speaker   Level: Format of the guidelines:   - Lack of summarized version of the guideline - Need for online version of the guideline which would mean ease in searching terms and accessing related links - Need for greater user participation (discussion fora, asking questions, debates, etc.)   Level: Use and utility facilitators:   - The need for action plans and other tools attached to the guideline   Internal barriers:   - Physician willingness to adhere to guidelines - Time and effort required to understand CPGs - Lack of acceptance of guidelines as a tool - Culture of not using CPGs at other healthcare levels   External barriers:   - “Specialized practice does not follow the guidelines” - Lack of CPG dissemination and implementation in specialized care - Pressures from the pharmaceutical industry that could interfere with putting the guidelines into practice - “Methodology followed to learn the guidelines”   Tailored to pre-identified barriers  Co-designed by patients or professionals: NR | | Target: Professionals  Intervention: Multifaceted  Type: PROFESSIONAL: Distribute guideline information + Present guideline materials at meetings + Educate groups about guideline intent/benefits; STRUCTURAL CHANGES: Information/communication technology  Content:  Resources available to control group + Presentation of guideline content by physicians who developed guidelines; access to special website containing guideline content including recommendations for monitored lab testing and prescriptions, drug-related information for professionals; cardiovascular risk workshops; four diabetic foot workshops    Format: 8 cardiovascular risk workshops for clinicians, 8 cardiovascular risk workshops for nurses, 4 diabetic foot workshops for nurses, websites, clinical meetings  Delivery: In-person, online  Timing: 2.5-month intervention period; 1-year post-intervention period  Personnel: Physicians involved in the development of each guideline  Control: Guidelines sent by email, published on intranet, presented in meetings in every primary care unit by local physicians | Mixed results  There was no improvement in the percentage of patients with diabetes having at least 1 annual HbA1c measurement (weighted mean difference (WMD) 3.83 [95% CI -3.49 to 11.15], intracluster correlation (ICC) 0.0309, *p* = 0.297), or in the number of patients with hypertension having annual general lab tests (WMD 16.22 [95% CI -7.35 to 39.80], ICC 0.0039, *p* = 0.172) between intervention and usual care groups.  There was a significant difference in the number of eligible patients with a coronary risk assessment in the intervention group compared to the usual care group:  Weighted mean difference between number of dyslipidemia patients with a coronary risk assessment in the intervention group compared to the control group (13.58 [95% CI 5.00 to 22.16], ICC 0.0223, *p* = 0.003 in women; 12.91 [95% CI 5.24 to 20.57], ICC 0.0000, *p* = 0.001 in men) was significant in favour of guideline concordance in intervention group  The intervention group had significantly more people newly prescribed statins with a previous coronary risk assessment (WMD 23.09 [95% CI 7.26 to 38.92], *p* = 0.005) and significantly fewer low-risk women newly prescribed statins (WMD -3.08 [95% CI -5.20 to -0.94], *p* = 0.006), in accordance with the dyslipidemia guideline |
| Karlsson [46]  2018  Sweden  Anticoagulant therapy in patients with atrial fibrillation at risk of stroke | Cluster RCT  43 primary care clinics  Intervention group: 22 clinics  Control group: 21 clinics | Context: Quality improvement  To investigate if an electronic-health-record-integrated clinical decision support system could  improve adherence to stroke prevention guidelines in patients with atrial fibrillation in the primary care setting | Theory: NR  Barriers mentioned in the paper’s text (method used to identify them NR) include:   - Difficulties identifying atrial fibrillation - The conditions warranting the use of anticoagulants in patients at increased risk of stroke - Reluctance to use potent anticoagulants due to increased risks of falling and bleeding   Barriers identified in the literature include:   - Patient and physician awareness and education levels   (Oldgren et al., 2014)  Tailored to pre-identified barriers: NR  The decision-support system was developed in collaboration with cardiologists and primary care professionals | | Target: Professionals  Intervention: Single  Type: STRUCTURAL CHANGES: Information/communication technology; PROFESSIONAL: Provide reminders to individuals about intent/benefits + Provide alerts when practice deviates  Content: The decision support system is activated when a patient with a diagnosis of atrial fibrillation or atrial flutter is  being logged into the electronic health record. If the patient has additional risk factors for stroke according to the  CHA2DS2-VASc algorithm (based on age, sex, and ICD codes listed in the supplemental text) and is without anticoagulant therapy according to the medication list, a pop-up screen warning appears. Clicking on the warning guides user to an overview of patient’s risk factors and a recommendation to consider anticoagulant therapy  Format: Clinical decision support tool  Delivery: Electronic health record  Timing: Study duration was 12 months  Point-of-care decision support tool  Personnel: Professionals in primary care clinics  Control: Not specified (but presumably no CDSS) | Modest positive outcomes  The present study demonstrates that a clinical decision support tool can increase guideline adherence for anticoagulant therapy in patients with atrial fibrillation  No difference in the baseline proportions of eligible patients prescribed anticoagulants in the intervention and control groups, respectively: 70.3% [5,186/7,370; 95% CI 62.9% to 77.7%] vs. 70.0% [4,187/6,009; 95% CI 60.4% to 79.6%], *p* = 0.83  At the 12-month mark, there was a significant difference in anticoagulant prescription in the intervention group vs. the control group (73.0% [95%CI 64.6% to 81.4%] vs. 71.2% [96%CI 60.8% to 81.6%]), *p* = 0.013  Over the course of the study, intervention group had a lower incidence of significant bleeding compared to the control group: 12/1000 patients [95%CI 9 to15]) vs. 16/1000 patients [95%CI 12 to 20], *p* = 0.04  Over the study period, there were no clinically significant differences between intervention and control groups in the incidence of stroke, transient ischemic attack, or systemic thromboembolism: 49 [95% CI 43 to 55] per 1,000 patients with atrial fibrillation in the intervention group vs. 47 [95% CI 39 to 55] per 1,000 patients with atrial fibrillation in the control group, *p* = 0.64) |
| Knappe [47]  2018  Germany  Unipolar depression | RCT (pre/post)  46 GP practices in intervention (665 patients), 42 in control group (526 patients) | Context: Quality improvement  To determine whether accessible materials improve adherence to the guideline for diagnosis and treatment of unipolar depression | Theory: NR  Tailored to pre-identified barriers: NR  Co-designed with patients or professionals: NR | | Target: Patients and professionals  Intervention: Multifaceted  Type: PROFESSIONAL: Distribute guideline material; PATIENT: Print material; STRUCTURAL CHANGES: Information/communication technology  Content and Format:  Flowcharts for diagnostics and suicidality, access to online consultation, case management forms, documentation, patient reported questionnaires  Delivery: In person  Timing: 15 minutes  Personnel: NR  Control: Care as usual | No change  No difference in recognizing depression nor in treatment.  The proportion of correctly identified depression cases was similar in the intervention (47.2 %) and the control group (42.3 %,  *p* = 0.537  No effects were observed regarding the usage of the tools, practitioners’ attitudes towards national depression guidelines, and depression treatment procedures. |
| Luitjes [48]  2018  Netherlands  Hypertensive disorders in pregnancy | Cluster RCT  Sixteen hospitals (clusters) with  obstetricians, gynecologists, residents, and clinical midwives; n = NR  Enhanced intervention group: 8 hospitals consisting of 270 patients pre-implementation and 947 patients post-implementation  Minimal intervention group: 8 hospitals consisting of 262 patients pre-implementation and 815 patients post-implementation | Context: Quality improvement  Determine utility of audit and feedback in addition to a computerized decision support system designed to reduce major maternal complications, increase adherence to guideline recommendations for management of hypertension in pregnancy compared to audit and feedback alone | Theory: NR  Decision-support system adapted to pre-identified barriers from:  (1) the literature  (2) Interviews  (3) Questionnaires  (2) and (3) from a pilot study by this study team (Luitjes et al., 2010)  The pilot study revealed barriers at the level of the guideline itself, including, in order of prevalence:  2a) Layout of the guideline  2b) Lack of clarity about the algorithm underpinning the guideline  2c) Doubts about guideline accuracy  2d) Lack of clarity about guideline contents themselves  2e) Suspicion that the guideline could be misused under medical disciplinary law  2f) Guideline did not afford enough room to consider patients’ wishes  The decision-support system was meant to address, and was therefore was tailored to, barriers 2a) Layout of the guideline, 2b) Algorithm underpinning the guideline, 2e) Misuse of guideline under medical disciplinary law, and 2f) Room to consider patients’ wishes  Co-designed with patients and professionals: Quality indicators from guideline for use in the decision-support system were extracted by a panel consisting of experts in clinical obstetrics, maternal death, guidelines, guideline implementation and quality, quality of care; and a patient organization for pre-eclampsia and HELLP syndrome | | Target: Professionals  Intervention: Multifaceted  Type: PROFESSIONAL: Provide feedback about compliance + Provide feedback from healthcare professionals; STRUCTURAL CHANGES: Quality improvement/performance measurement system+ Information/communication technology  Content:  Minimal intervention group:  (1) Audit and feedback – Electronic report containing scores based on 12 quality indicators that assessed adherence to guideline and corresponding feedback; bar chart showing range of performance per quality indicator – all pertaining to that hospital only  Enhanced intervention group:  (1) Audit and feedback -  Report on hospital’s quality indicator scores and associated data as in minimal intervention group, comparison to all other hospitals at aggregate level during a meeting  (2) Electronic decision support system – customized patient management recommendations as per guideline  Format: Emailed audit and feedback reports, multidisciplinary meetings, electronic decision support tool  Delivery: Email, in person, electronic decision support system  Timing: In-person multidisciplinary meeting 4 weeks after delivery of feedback report (intervention group) or after data collection for post-implementation measurement was started (control group)  Personnel: NR  Control: Emailed audit and feedback report alone | No change  There was a significant decrease in major maternal complications in both groups compared to the pre-implementation period, *P* = NR:  Enhanced intervention group: 10.6% vs. 14.1%, OR (95%CI): 0.64 (0.42, 0.96)  Minimal intervention group: 12.8% vs. 16.8%, OR (95%CI): 0.70 (0.55, 0.89)  The minimal intervention group saw a significant increase in one unfavourable patient-related secondary outcome (of four secondary outcomes):   - Neonatal mortality: 0.38% vs. 0.99%, OR (95%CI): 3.30 (1.83, 5.96), P < 0.0001   And a significant decrease in another unfavourable patient-related secondary outcome:   - Cesarean section: 38.9% vs. 26.5%, OR (95%CI): 0.47 (0.50, 0.76), *P* < 0.0001   These trends were not observed in the decision-support intervention group.  There were no significant differences between enhanced and minimal intervention groups when comparing their respective pre-intervention and post-intervention scores for the following process-related secondary outcome measures of guideline adherence:   - Stabilization of patients - Appropriate treatment with magnesium sulphate - Appropriate treatment with antihypertensive drugs - Maintenance of target blood pressure values - Providing key information to patients if condition is worsening - Completion of laboratory tests - Urine test |
| Mellin [49]  2018  USA  Use of antiemetics for chemotherapy-induced nausea and vomiting | Retrospective, cohort, pre- post  6 nurse practitioners and physician assistants (advanced practice providers), 83 oncology unit nurses | Context: Quality improvement  To determine if education sessions for healthcare providers increased adherence to antiemetic guidelines; determine rates of nausea and vomiting in patients receiving highly emetogenic chemotherapy | Theory: Plan-Do- Study-Act (PDSA) method  Tailored to pre-identified barriers: NR  Co-designed with patients or professionals: NR | | Target: Professionals  Intervention: Single  Type: PROFESSIONAL: Educate groups about guideline intent/benefits  Content: Case studies and questions related to guidelines for management of chemotherapy-induced nausea and vomiting  Format: In-person or voice-recorded educational sessions  Delivery: In person  Timing:  Pre-intervention, post-intervention, and 3-months post--intervention  5 day-shift education days and 3 night-shift education days, spacing of education days NR; 30-minute education sessions  Personnel: NR | Mixed results  Post-intervention knowledge scores increased for both advanced practice providers (61% to 72%,  n = 5) and nurses (58% to 66%,  n = 41) compared to pre-intervention  Three-month post-intervention scores remained higher in the nurse group (66%) but dropped from the immediate post-intervention score in advanced practice providers (72% vs. 70%)  Overall average scores in pre-, post-, and 3-month post-intervention periods (nurse group: 58%, 66%, and 66%; advanced practice provider group: 61%, 72%, and 70%) were not significantly different (*p* = NR for all) but individual domains were significantly improved in the nurse group. The advanced practice providers only demonstrated improvement in one domain but small sample sizes precluded determination of statistical significance.  Chemotherapy-induced nausea and vomiting was present in 14/37 patients in the post-intervention period vs. 42/86 in the pre-intervention period, not a significant difference (*p* = 0.13).  Of the 37 patients at post-intervention (of 86 at pre-intervention), 8 received guideline-concordant medication for highly emetic chemotherapy, a 15% significant increase, *p* = 0.01 |
| Pauwels [50]  2018  Indonesia  Diagnosis and triaging of patient suspected of having dengue | Cluster RCT  Intervention group: Nurses  (n = NR) and 4 GPs overseeing 38 pre-intervention and 105 post-intervention patients  Control group: Nurses (n = NR) and 3 GPs overseeing 50 pre-intervention and 126 post-intervention patients | Context: Quality improvement  To investigate the effectiveness of an educational intervention based on an algorithm adapted from the WHO dengue guideline on guideline adherence among primary  healthcare providers | Theory: NR  Tailored to pre-identified barriers: NR  Co-designed with patients or professionals: NR | | Target: Professionals  Intervention: Multifaceted  Type: PROFESSIONAL: Present guideline material + Educate groups about guideline intent/benefits + Print material (handout, poster)  Content: Education session based on WHO guideline algorithm for dengue – emphasizing history taking and physical examination; 5 example cases that had to be solved by group, handouts of algorithm, posters of algorithm  Format: Education session, paper handouts, poster  Delivery: In person, printed material  Timing:45-minute education session  Personnel: NR  Control: Unclear | No change/impact  Statistical analyses showed a significantly better  outcome in correct diagnosis in the intervention group vs. control group after the intervention (75.2% vs. 62.7%, *p* = 0.041), particularly in nurses (81.0% vs. 62.2%, *p* = 0.033)  However, completeness of history taking (measured by 9 items) and physical examination (13 items) showed no post-intervention improvement in the intervention group, with % of uninvestigated cases of some dengue symptoms being higher post-intervention, and others higher pre-intervention  Results are considered  inconclusive due to incompleteness of relevant information, which most probably leads to many false positive correct diagnoses and triaging |
| Pinto [51]  2018  Portugal  Prescribing of non-steroidal anti-inflammatory drugs, acid secretion modifiers, and antiplatelets in primary care | Cluster RCT  239 physicians in 38 practices  Intervention group: 120 practices  Control group: 119 practices | Context: Quality improvement  To determine if educational outreach visits improve family physician prescribing  compared with passive guideline dissemination | Theory: Template for Intervention Description and Replication (TIDieR) checklist  Tailored to pre-identified barriers: NR  Barriers and facilitators were identified as part of the intervention during academic detailing  Co-designed by patients or professionals: NR | | Target: Professionals  Intervention: Multifaceted  Type: PROFESSIONAL: Educate individuals about guideline intent/benefits + Print material (brochure, handout)  Content: Academic detailing: one-on-one discussions of one of three guidelines, assess educational needs of physician, key messages about guideline, addressing benefits of adherence, and barriers and enablers of change; visual aid to highlight each guideline’s key-messages, point-of-care summary handout  Format: Educational outreach visits, brochure, point-of-care summary  Delivery: In person, print material  Timing:  Each physician had three 20-minute visits, one per guideline  1-2 visits per month  All visits pertaining to a given guideline had to be delivered to all physicians in a given practice within the same month.  Study took place over 18 months  Personnel: The detailing team consisted of three of the authors (two trained as family physicians and one as a pharmacist), six family physicians, and three family medicine residents in their fourth and final year of training (from Additional File 1 of paper)  Control: Passive dissemination of guidelines via a website | No change/impact  Educational outreach visits were unsuccessful in improving compliance with guideline recommendations  among Portuguese family physicians. No effects were observed at 1, 6, and 18 months after the intervention, and there  were no associated cost savings  No significant difference in percentage of patients prescribed acid secretion modifier omeprazole (%, 95%CI) between intervention and control groups, respectively, at:   - 1 month: 46.86 (44.34, 49.39) vs. 47.36 (44.81, 49.91), *p* = 0.744 - 6 months: 48.02 (45.58, 50.46) vs. 47.90 (45.01, 50.79), *p* = 0.696 - or 18 months: 46.28 (43.77, 48.79) vs. 47.15 (44.39, 49.91), *p* = 0.971   No significant difference in percentage of non-steroidal anti-inflammatory drug cycloxigenase-2 prescribed (%, 95%CI) between intervention and control groups, respectively, at:   - 1 month: 11.70 (9.83, 13.57) vs. 15.38 (12.87, 17.90), *p* = 0.131 - 6 months: 11.59 (9.28, 13.89) vs. 15.74 (13.42, 18.05), *p* = 0.061 - or 18 months: 12.07 (9.75, 14.41) vs. 13.08 (10.75, 15.41), *p* = 0.085   No significant difference in percentage of non-steroidal antiplatelet clopidogrel prescribed (%, 95%CI) between intervention and control groups, respectively, at:   - 1 month: 0.098 (0.886, 0.107) vs. 0.103 (0.094, 0.112), *p* = 0.456 - 6 months: 0.090 (0.082, 0.098) vs. 0.099 (0.089, 0.108), *p* = 0.230 - or 18 months: 0.091 (0.083, 0.098) vs. 0.091 (0.082, 0.100), *p* = 0.840 |
| Presseau [52]  2018  UK  Management of type 2 diabetes in primary care | Cluster RCT  44 practices  Intervention group: 22 practices with 153 GPs, nurses, and healthcare assistants  Control group: 172 GPs, nurses, and healthcare assistants  1,138,105 prescriptions assessed | Quality improvement  To evaluate the effectiveness of an intervention in improving six guideline-recommended  health professional behaviours in type 2 diabetes management:   1. Prescribing for blood pressure and 2. Glycemic control, 3. Providing physical activity and 4. Nutrition advice and 5. Providing updated diabetes education and 6. Foot examination | Theory: UK Medical Research Council framework for complex interventions (Eccles et al., 2011), behaviour change techniques specified within: Social Cognitive Theory, Health Action  Process Approach, Dual Process Model, theory-based  approaches to multiple goal pursuit  Tailored to pre-identified barriers: Healthcare providers were asked to report discrepancies between current performance and goals during group meetings, then helped to identify barriers and formulate if-then plans to work around them.  They were also provided with patient-developed materials (method used by patients to develop materials NR) that pre-identified barriers and solutions  to assist in providing physical activity and healthy eating  support to patients.  Optional short videos contained practice-based examples of patient-clinician interactions that illustrated barriers to initiating insulin and providing physical activity advice, and solutions for how to manage these barriers  Co-designed with patients and professionals as detailed above | | Target: Professionals  Intervention: Multifaceted  Type: PROFESSIONAL: Educate groups about guideline intent/benefits + Educate individuals about guideline intent/benefits  Content: Facilitating behaviour change by helping healthcare providers pre-identify barriers to their own behaviour change and develop plans to circumvent barriers, provide patient perspective of barriers to receiving proper healthcare, examples of other providers encountering barriers and how to sidestep them  Format: Outreach visits containing facilitated discussion, reflection, short videos  Delivery: In person, video  Timing: 90 minutes  Personnel: Content expert (nurse or doctor) and a behaviour change expert  Control: No intervention | No change/impact  There was no measurable benefit to practices’ participation in this intervention as per patient report  Intervention (29% to 37%) and control practices (31% to 35%) increased frequency of insulin initiation relative to baseline but did not differ from each other at follow-up (IRR: incidence rate ratio, 95% CI, *p*-value): IRR 1.18, 0.95 to 1.48, *p* = 0.13  Intervention (45% to 53%) and control practices (45% to 50%) increased frequency of blood pressure prescriptions relative to baseline but did not differ from each other at follow-up (IRR: incidence rate ratio, 95% CI, *p*-value): IRR 1.05, 0.96 to 1.16, *p* = 0.29  Both intervention (75% to 78%) and control groups (74% to 79%) increased rates of performing foot examination relative to baseline, but **control** practices increased this significantly more (OR 0.84, 95CI 0.75 to 0.94, *p* < 0.01)  Fewer patients in the intervention group (33%) compared to the control group (40%) reported receiving updated diabetes education (OR 0.74, 95%CI 0.57 to 0.97, *p* = 0.03) at 12-month follow-up  Intervention and control groups did not differ in frequency of discussions about nutrition (intervention = 73%, control = 72%; OR 0.98, 95% CI 0.59 to 1.64, *p* = 0.95) or physical activity (intervention = 57%, control = 62%; OR 0.79, 95% CI 0.56 to 1.11, *p* = 0.17) |
| Quanbeck [53]    2018  USA  Opioid prescribing in primary care | Randomized matched-pairs design  Intervention group: 4 clinics  Control group: 4 clinics | Context: Quality improvement  To determine whether systems consultation strategy was feasible, acceptable, and effective  in improving adherence to opioid-prescribing guidelines and  reducing morphine-equivalent daily dose for  patients on long-term opioid therapy | Theory: Reach, Effectiveness, Adoption, Implementation, Maintenance (RE-AIM) framework, Plan-Do-Study-Act (PDSA) cycles, nominal group technique (Delbecq et al., 1975), integrative group process (Gustafson et al., 1992)  Tailored to pre-identified barriers: NR  Co-designed with professionals using organizational coaching/external facilitation at the clinic level | | Target: Professionals  Intervention: Multifaceted  Type: PROFESSIONAL: Tailor guideline + Provide feedback on compliance + Provide feedback from healthcare professionals + Print material (flowchart, PDSA forms); FINANCIAL (Health professional): Incentive (individual financial reward or benefit for compliance) + Grant or allowance to group/institution (not tied to compliance) + ORGANIZATIONAL (Health professional): Create an implementation/multidisciplinary team  Content:  Formulation of checklist based on opioid guideline recommendations using integrative group process and structured Delphi process  Systems consultation:  (1) Audit and feedback:  Baseline information, performance feedback, improvement opportunities – re-enactment of opioid prescription process at each clinic (walkthrough), which was summarized in a flowchart to expose inefficiencies and point out areas for improvement  (2) Academic detailing: expert in addictions medicine provides feedback on clinical practice improvement – each clinic’s designated change teams reported on work, results, key learnings, used nominal group technique and PDSA cycles to identify and respond to problems  (3) Organizational coaching/external facilitation: tailoring guideline to specific clinical context – Clinic’s change teams reviewed data on the guideline concordance of their clinical performance and PDSA forms prepared by study facilitator; sought advice from consultants on how to implement various guideline components; a final review of their performance data was conducted  Format: Audit and feedback, academic detailing, organizational coaching/external facilitation, meetings, site visits,  Delivery: In-person, videoconference or teleconference, print material (flowchart, PDSA forms)  Timing:  Creating the checklist: Full-day meeting  1-hour physician consultant and change team meetings at months 1, 2, and 6; tele/videoconferences in months 3, 4, and 5  Estimated 9 hours spent/change team member on the study  Staggered 6-month intervention period for clinics  Personnel: Organizational coaches trained physician assistants. Two physician consultants certified in family and addiction medicine respectively provided academic detailing and external facilitation; study facilitator provided external facilitation, meetings, site visits, directed use of tools; change team members per clinic: 6-8 staff comprising at least 1 physician, 1 registered nurse, 1 medical assistant or practical nurse, and 1 administrative staff member  Control: Nature of care NR | Mixed results  The implementation strategy demonstrated feasibility, acceptability, and  effectiveness  At 6 months, significant differences between intervention and control groups in (slope of intervention group minus control group trend [95%CI], *p*-value):   - Proportion with urine drug screen (0.029 [0.050 to .008], *p* = 0.011) - Proportion with treatment agreement (0.03 [0.051 to 0.008], *p* = 0.012) - Proportion with co-prescribed benzodiazepines (-0.002 [0.000 to -0.003], *p* = 0.019) - Proportion with mental health screen (0.029 [0.053 to 0.005], *p* = 0.024)   Proportion co-prescribed benzodiazepines (-0.002 [0.000 to -0.003], *p* = 0.019)  At 6 months, no difference between groups in (slope of intervention group minus control group trend [95%CI], *p*-value):   - Proportion of patients with a consistent opioid prescription (-0.0001 [0.0000 to -0.0002], *p* = 0.237) - Average morphine-equivalent daily dose (0.581 [0.75 to -1.92], *p* = 0.425) - Proportion of patients with a morphine-equivalent daily dose > 120 (-0.001 [0.003 to -0.006], *p* = 0.624)   At 12 months, significant differences between intervention and control groups in (slope of intervention group minus control group trend [95%CI], *p*-value):   - Proportion with a mental health screen (0.017 [0.028 to 0.006], *p* = 0.003) - Average morphine-equivalent daily dose (-1.261 [-0.425 to -2.097], *p* = 0.003) - Proportion with morphine-equivalent daily dose > 120   (-0.003 [-0.001 to -0.006], *p* = 0.018)  At 12 months, no difference between groups in (slope of intervention group minus control group trend [95%CI], *p*-value):   - Proportion of patients with a treatment agreement (0.005 [0.0012 to -0.002], *p* = 0.146) - Proportion with urine drug testing (0.005 [0.013 to -0.002], *p* = 0.153) - Proportion with co-prescribed benzodiazepines (0.001 [0.003 to -0.001], *p* = 0.353) - Proportion of patients with a consistent opioid prescription (0.0000 [0.0001 to -0.0001], *p* = 0.975)   Study period coincided with release of CDC opioid guidelines as well as increased attention to opioids in the general public, and therefore, likely resulted in motivation within clinics to change opioid-prescribing practices.  Differences between intervention and control clinics narrowed between the 6- and 12-month marks for several outcomes, but rate of change was greater for intervention clinics at both these time points.  At the end of the intervention, more than 80% of staff agreed or strongly agreed with the statement, “My clinic’s workflow for opioid prescribing is easier” |
| Ranta [54]  2018  New Zealand  Transient ischemic attack in primary care | Cluster RCT secondary analysis  291 GPs clustered by practice  Of GPs belonging to practices randomized to electronic decision support: 71 GPs had attended an education session, 101 GPs had not attended  Of GPs belonging to practices randomized to no decision support: 69 GPs had attended an education session, 50 GPs had not | Context: Quality improvement  To determine whether attendance at an education session differentially influenced management and outcomes of transient ischemic attack, including reducing early recurrent stroke, by non-specialists (GPs) randomized to receive or not receive an electronic decision support tool | Theory: NR  Barriers to GP management of transient ischemic attack pre-identified in the literature were:   - Lack of diagnostic confidence due to infrequency of seeing transient ischemic attack - Deferral of specialist referral or initiation of medical management due to unfamiliarity with condition   (Lasserson et al., 2008; Ferro et al., 1996; Ranta and Cariga, 2013; Ranta et al., 2015)  Tailored to pre-identified barriers: NR  Co-designed by patients or professionals: NR | | Target: Professionals  Intervention: Multifaceted  Type: PROFESSIONAL: Present guideline materials at meetings; STRUCTURAL CHANGES: Information/communication technology  Content: Education session: Transient ischemic attack diagnosis, triage,  and management. Common symptoms and symptoms that mimicked the condition were reviewed  Electronic decision support: Clicking on icon in a desktop tool opens an electronic checklist for relevant aspects of the condition, which then issues guideline-concordant recommendations, prescriptions, radiology access, referral forms  Format: Slide presentation  Delivery: In person, PowerPoint presentation, Desktop application  Timing: 90-minute presentation  Personnel: Stroke neurologist  Control: No electronic decision support | No change/impact  Purpose of study was to determine presence, if any, of interactions between education sessions and the provision of the decision support tool  No significant interaction of education sessions and randomization condition on guideline adherence (*P* = 0.91) or reduction of patient outcomes: 90-day stroke or vascular events following the transient ischemic attack (*P* = 0.31)  Electronic decision support tool had an effect of unreported significance on guideline adherence at the 90-day mark:  53/71 patients (74.6%) who had been treated by GPs randomized to receive the decision support tool and who had also chosen to attend the education session made guideline-concordant decisions vs. those treated by GPs with no decision support tool but who had also attended the session (27/69, 39.1%)  78/101 patients (77.2%) who had been treated by GPs randomized to receive the decision support tool but who had not attended the education session made guideline concordant decisions vs. those treated by GPs with no decision support tool but who had also not attended the session (22/50, 44.0%)  1/71 patients (1.4%) who had been treated by GPs randomized to receive the decision support tool and had also chosen to attend the education session, experienced a stroke, vascular event or death vs. those treated by GPs with no decision support tool but who had also attended the session (9/69, 13%)  6/101 patients (5.9%) who had been treated by GPs randomized to receive the decision support tool but who had not attended the education session. Experienced a stroke, vascular event or death vs. those treated by GPs with no decision support tool but who had also not attended the session (8/50, 16%) |
| Safatly [55]  2018  Australia  Management of patients on anticoagulant warfarin with supratherapeutic International Normalised Ratio (INR) results | Cohort, pre- post  Emergency medical and nursing staff,  n = NR  Pre-intervention: 60 patients  Post-intervention: 52 patients | Context: Quality improvement  To evaluate the efficacy of an educational intervention aimed at improving compliance with guidelines for management of emergency department patients with supratherapeutic INR levels | Theory: NR  Tailored to pre-identified barriers: NR  Co-designed with patients or professionals: NR | | Target: Professionals  Intervention: Multifaceted  Type: PROFESSIONAL: Educate groups about guideline intent/benefits + Print material; STRUCTURAL CHANGES: Quality improvement, performance measurement system  Content: Education on management of patients presenting to emergency department with supratherapeutic INR, emailed guidelines, and provided printed pocket-size copy of guidelines  Format: Formal and informal educational sessions  Delivery: In-person, print material  Timing:  Pre-intervention: 1 July 2014-30 June 2015 (12 months)  Educational sessions over 1 July 2015-31 December 2015 (6 months)  Post-intervention: 1 January 2016-31 December 2016 (12 months)  Personnel: NR | No change  Non-compliance with various aspects of management (e.g., Vitamin K dosing, reversal of INR with significant bleeding [individual *p*-values for these variables NR]) were similar pre-intervention compared to post-intervention – cumulative non-compliance over all 7 variables measured: 17/60 (28.3%) vs. 14/52 (26.9%), *p* = 0.87 |
| Suman [56]  2018  Netherlands  Low back pain management | Stepped-wedge cluster-randomized controlled trial structure analyzed as controlled pre- post study  25 practices with 53 GPs  Intervention group: 12 practices with 24 GPs containing 2677 patient-GP contacts for 1354 patients  Control group: 13 practices with 29 GPs containing 2453 patient-GP contact for 1195 patients | Context: Quality improvement  To ascertain the impact of a multifaceted intervention to improve guideline-recommended care for low back pain in general practices, compared to usual care | Theory: NR  This barrier was identified by patients in previous studies by the study team:   - Poor communication and collaboration between healthcare providers   (from study protocol, Suman et al., 2015; van Tulder et al., 2010; Anema et al., 2009)  The intervention was tailored to this barrier  using strategies described by Grol and Wensing (2013) (from process evaluation study carried out alongside present study, Suman et al., 2017)  A barriers and facilitators “carousel” in which small groups of healthcare providers gathered to discuss this barrier and strategies to overcome it was conducted (from study protocol, Suman et al., 2015)  Co-designed by professionals: The “carousel” was led by a member of the research team as well as one healthcare provider – strategies to overcome professional-identified barriers were sent by email as a reminder, as well as a social contact map of other healthcare providers | | Target: Patients and professionals  Intervention: Multifaceted  Type: PROFESSIONAL: Educate groups about guideline intent/benefits + Provide reminders to individuals/groups about intent/benefits; ORGANIZATIONAL (Health professional): Create an implementation/multidisciplinary team + Communication between distant health professionals; STRUCTURAL CHANGES: Quality improvement, performance management; PATIENT/CONSUMER: Education  Content: (Professional) Education emphasizing multidisciplinary communication and patient-physician communication to reduce referrals to specialists, reduce diagnostic imaging, and increase consideration of patient risk factors; (Patient) topics related to low back pain (e.g., work, daily life), exercises  Format: Multicomponent, multidisciplinary continuing medical education and collaboration, email reminder about strategies to surmount barriers; social media platforms, video messages  Delivery: In person, email, online - social media, patient-oriented video messages  Timing: 3-month baseline period (Control group: October –December 2013, Intervention group: January-March 2014)  Similarly consecutive follow-up periods 1 year later  Personnel: NR/authors performed retrospective review of patient records  Control: Usual care | Mixed results  Referrals to neurology decreased by half (guideline-concordant) after intervention (100/1426 (7%) vs. 50/1251 (4%),) but remained consistent in the usual care group (109/1211 (9%) vs. 99/1242 (8%), *p* < 0.01)  Overall referral rates to medical specialists were not significantly different at baseline and follow-up between intervention (171/1426 contacts (12%) vs. 100/1251 contacts (8%) and usual care groups (109/1211 contacts (9%) vs. 99/1242 contacts (8%), *p* = NR  Overall rate of imaging decreased within both groups over time but was not significantly different between groups (intervention: 200/1426 (14%) vs. 138/1251 (11%); usual care: 145/1211 (12%) vs. 137/1242 (11%)  Improvement in health professional satisfaction was reported in the corresponding process evaluation study, Suman et al., 2017 |
| Sun [57]  2018  USA  Colorectal cancer screening | Pilot RCT  Early intervention: 20 primary care physicians, 2020 patients  Delayed intervention: 22 primary care physicians,  1098 patients | Context: Quality improvement  To determine whether an intervention initiated by a physician network had an impact on colorectal cancer screening rates among Chinese American individuals | Theory: Health Behavior Framework  Tailored to pre-identified barriers: Intervention guided by barriers to colorectal cancer screening among Asian Americans identified in the literature:   - Lower educational attainment - Limited proficiency in English - Recent immigration and lower acculturation - Absence of health insurance   (Ma et al., 2012)   - Lack of physician recommendation   (Yip et al., 2006)   - Fear of abnormal results - Lower perceived susceptibility   (Sun et al., 2004)   - Lack of knowledge, time, transportation   (Ma et al., 2012)   - Language barrier between patient and physician   (Thompson et al., 2014)  through provision of bilingual letter and returnable fecal occult blood test kit  A component of the intervention, the mailed package, was designed by incorporating feedback from a layperson focus group in the translation to Chinese of the mailed package | | Target: Patients and professionals  Intervention: Multifaceted  Type: PROFESSIONAL: Educate groups about guideline intent/benefits; PATIENT/CONSUMER: Print material, STRUCTURAL CHANGES: Method of service delivery  Content: Continuing medical education seminars focused on national guidelines for colorectal cancer screenings and treatments, with a focus on fecal occult blood tests (primary care physician intervention); Patient mailed package covering a variety of topics relevant to colorectal cancer screening and treatment (patient intervention)  Format: Educational seminars; patient mailed package (mailer)  Delivery: in-person (seminars)  Timing: Series of 3 seminars within a 3-year period (primary care physician intervention); one-time patient mailed package in last year of study (early intervention) or year after last seminar (delayed intervention)  Personnel: Specialist physicians in colorectal surgery, oncology, and gastroenterology (speakers for seminars)  Control: Delayed intervention group (primary care physicians with no continuing medical education and patients receiving intervention mailed package in year 2) | Mixed impact  Uptake of fecal occult blood test screening improved significantly from baseline to year one in early-intervention group vs. delayed intervention group (29.2% difference in improvement, chi-square 35.3; *P* < 0.0001)  Uptake of fecal occult blood test screening improved significantly more from baseline to year 2 in the delayed intervention group: 23.9% difference in improvement, chi-square 23.8; *P* < 0.0001  Although patients in the early intervention group were given more colonoscopy or sigmoidoscopy screening compared to those in the delayed intervention group (chi-square 4.5, *P* = 0.034), and that the screening rates were significantly different among all study periods including baseline (early intervention - baseline: 10.5%, year 1: 13.2%, year 2: 6.1%; delayed intervention - baseline: 6.0%, year 1: 9.5%, year 2: 3.1%; chi-square 11.4, *P* = 0.0034), the study reports there being no impact of either the early intervention (chi-square 0.1; *P* = 0.80) or the delayed intervention (chi-square 0.4; *P* = 0.55)  Effect sizes of early and delayed interventions, respectively: 29.2 percentage points vs. 23.9 percentage points, chi-square 0.4, *P* = 0.54  Overall effect size of the mailed component of the intervention, with or without continuing medical education was estimated at a difference of 26.6 percentage points (95%CI 22.0 to 31.2 percentage points) |
| Witt [58]  2018  USA  Opioid therapy for chronic noncancer pain in rural primary care | Non-randomized stepped wedge  23 primary care providers overseeing 435 patients who completed the study (clusters NR) | Context: Quality improvement  To determine whether the implementation of process changes improved adherence to chronic opioid therapy prescribing and monitoring guidelines, and resulted in decreased chronic opioid therapy for chronic noncancer pain, in a primary care health system | Theory: Used an approach from Parchman et al. (2017) that identifies “6 building blocks of clinic change”  A barrier pre-identified in the literature is that:   - Some clinicians regarding urine drug tests as law enforcement rather than medical tools   (Krebs et al., 2014)  An anecdotal barrier reported by the study authors is:   - Limited rural availability of pain medicine, behavioural health, and addiction medicine specialists   The development of local guidelines was accomplished with the input of physicians, nurse practitioners and physician assistants in family medicine, general medicine, urgent care, and pediatrics through a survey and focus groups that identified areas of improvement | | Target: Patients and professionals  Intervention: Multifaceted  Type: PROFESSIONAL: Tailor guideline +Achieve consensus that guideline should be implemented + Educate groups about guideline intent/benefits + Provide reminders to individuals/groups about intent/benefits + Provide feedback from healthcare professionals + Provide feedback on compliance + Enable self-audit (patient registry); PATIENT/CONSUMER: Reminder (opioid treatment agreement); ORGANIZATIONAL (Health professional): Create an implementation/multidisciplinary team + Communication between distant health professionals; STRUCTURAL CHANGES: Quality improvement, performance measurement system + Method of service delivery    Content: Educational sessions to primary care providers and nursing staff as well as other clinics; Opioid Use Review Panel was formed and provided on-request consultation to physicians for difficult cases through chart review and written recommendations; A prescribing registry of those undergoing chronic opioid therapy created; measures related to chronic opioid therapy extracted from electronic health record;  screening tools identified and/or designed and then utilized; opioid therapy agreement between primary care provider and patient instituted; standard workflow for clinics developed  Format: Implementation team, academic detailing, education sessions, practice facilitation, chart review, new patient registry and performance monitoring system, opioid therapy agreement  Delivery: In-person, mail, printed material  Timing: Implementation period: December 1, 2015-October 31, 2016  Post-implementation: November 1, 2016-April 30, 2017  Personnel: Registered nurse was coordinator and educator, Opioid Use Review Panel composed of members from primary care, pharmacy, behavioural health, and physical medicine | Positive changes  96/465 patients (22.1%; 95% CI, 18.4 to 26.2) had either discontinued chronic opioid therapy or were using average daily doses below 5mg (morphine-equivalent daily dose)  Pre- to post-implementation,  *p* = NR:   - 22% fewer patients taking morphine-equivalent daily doses ≥5mg - 17% fewer patients taking morphine-equivalent daily doses 5mg to 59mg - 4% fewer patients taking morphine-equivalent daily doses ≥60mg to 89mg - 1% fewer patients taking ≥90mg |
| Al Kalaldeh [59]  2017  Jordan  Enteral nutrition | Prospective observational study  127 (/210) ICU nurses overseeing 192 patients at four tertiary referral hospitals in Jordan  Pre-intervention: 80 patients  Post-intervention: 112 patients | Context: New guideline (existing guideline made available)  To assess the impact of nurse-led intensive care delivery on adherence to enteral nutrition guidelines in the context of the Jordanian healthcare system | Theory: NR  The main barrier identified by first author in previous works is the absence of guidelines (Al Kalaldeh, 2014; 2015)  The present study is tailored to pre-identified barriers by making the guideline available  Co-designed with patients or professionals: NR | | Target: Professionals  Intervention: Multifaceted  Type: PROFESSIONAL: Distribute guideline material + Advertise guideline material + Educate groups about guideline intent/benefits    Content: Interactive learning sessions based on 14 adopted recommendations from ASPEN enteral nutrition guidelines; wall poster that summarized guidelines  Format: Two interactive learning sessions, wall poster  Delivery: In person, print material  Timing:  Sessions were 6 hours in total and delivered to each hospital  Personnel: Principal researcher (Al Kalaldeh) | Positive impact  During the post-intervention phase, nurses exhibited higher adherence to:  Aspiration reduction measures (% cases):   - Measuring gastric residual volume: 67.9% vs. 26.3%,   *P* = 0.001   - Maintaining a certain head-of-bed elevation: 79.5% vs. 28.8%, *P* < 0.001 - Checking displacement of tube: 67.9% vs. 26.3%,   *P* = 0.001   - Continuous feeding methods: 42.9% vs. 20%, *P* = 0.027 - Twice daily use of chlorhexidine mouthwash: 49.1% vs. 10%, *P* < 0.001   Detection of enteral nutrition complications (% cases):   - Pulmonary aspiration assessment: 81.3% vs. 45.0%, *P* = 0.003 - Feeding-related diarrhea assessment: 87.5% vs. 51.3%, *P* = 0.004 - Feeding intolerance assessment: 76.8% vs. 26.6%, *P* < 0.001 - Naso-pharynx injury assessment: 65.2% vs. 43.8%, *P* = 0.029   Nutritional adequacy measures (% cases):   - Body weight: 54.4% vs. 26.3%, *P* = 0.005 - Calculation of nutritional requirements: 68.8% vs. 50%, *P* = 0.015 - Enteral nutrition starting point in median days since admission: 3.15 vs. 4.3, *P* = 0.053 - Inappropriate cessation of feeding in hours over 5 days: 12.7 vs. 32.2, *P* < 0.001 |
| Aloush [60]  2017  Jordan  Prevention of ventilator-acquired pneumonia | RCT with post-test only data  120 ICU nurses  Intervention group: 59 nurses  Control group: 47 nurses | Context: Quality Improvement  To determine the effect of educational sessions on nurse compliance with ventilator-acquired-pneumonia prevention guidelines, and factors that influence compliance | Theory: NR  Barriers pre-identified in the literature pertaining to ventilator-acquired pneumonia include:   - Lack of resources - Below-par compliance with infection-control standards   (Al-Rawajfah, 2014)   - Inadequate knowledge about ventilator-acquired pneumonia   (Hassan and Wahsheh, 2016)  Tailored to pre-identified barriers: NR  Co-designed with patients or professionals: NR | | Target: Professionals  Type: Single  PROFESSIONAL: Present guideline material at meetings + Educate groups about guidelines + Print material   Content: Mechanical ventilation management, information on updated guidelines: oral care, handwashing, use of suction, management of ventilator, patient position, antibiotic prophylaxis, deep vein thrombosis prophylaxis  Format: Educational sessions: discussions, videos, hard copies of slides  Delivery: In person, print material  Timing: Data collected over 6 months  4 education sessions of 2 hours each – 8 hours in total  Personnel: NR | No effect  No significant difference in compliance with guidelines when comparing mean compliance scores of intervention (14.1 ± 4.4) and control groups (12.8 ± 3.7, *P* = 0.15) |
| Coenen [61]  2017  Belgium  Prevention, diagnosis, and management of opportunistic infections in inflammatory bowel diseases in tertiary care | RCT  346 patients with inflammatory bowel diseases, lacking guideline-concordant vaccination status  Intervention group: 140 patients  Control group: 206 patients | Context: Quality improvement  To investigate the effect of a brief patient-geared educational intervention on vaccination rates | Theory: NR  A barrier preventing adequate vaccination identified in the literature was (Melmed et al., 2006):   - Patient nonawareness   Tailored to pre-identified barriers: NR  Component of intervention co-designed by a professional: Print material in intervention co-designed by an inflammatory bowel diseases nurse and the study team | | Target: Patients  Intervention: Multifaceted  Type: PATIENT/CONSUMER: Education (single) + Counselling + Print material (information brochure)  Content: Patient education consisting of discussion about previous vaccinations, recommended vaccinations, importance of vaccination as per international guidelines with the aid of the vaccination card and information brochure; prescriptions and vaccinations provided when requested by patient  Format: One-on-one patient education between nurse and patient, vaccine card, information brochure  Delivery: In person, print material  Timing: Post-intervention data collected 8 months after patient randomization  Patients enrolled between December 2014 and March 2015 (4 months)  15-minute-long patient education with nurse  Personnel: Inflammatory bowel diseases nurse  Control: Routine clinical care and vaccination without education by nurse | Positive impact  Vaccination rates for the following opportunistic infections were significantly higher in the intervention group than the control group at 8 months, *P* ≤ 0.001 for all:   - Influenza: 36% (18/50) vs. 10% (5/52) - Pneumococcus: 62% (53/86) vs. 23% (25/107) - Hepatitis B: 27% (24/89) vs. 5% (7/151) - Tetanus: 33% (11/33) vs. 2% (1/56)   Significantly more patients in the intervention group had taken all possible vaccines at 8 months (33%, 46/140) vs. patients in the control group (6%, 13/206),  *P* < 0.001 |
| Cummings [62]  2017  USA  Diagnosis of severe illness in low-income countries – Uganda | Staggered, pre-post, quasi-experimental  5759 patients  Pre-intervention period: 1633 patients  Intervention period: 4126 patients | Context: Quality Improvement  To describe the development, implementation, and impact of the intervention on vital sign collection and diagnosis of four severe illness conditions:  1.Undifferentiated shock  2. Sepsis  3. Severe respiratory distress  4. Altered consciousness  among patients hospitalized at four inpatient health facilities in western Uganda | Theory: Behavior Change Wheel Framework, COM-B (Capability, Opportunity, Motivation - Behavior) model, Plan-Do-Study-Act (PDSA) cycle, Template for Intervention Description and Replication (TIDieR) Checklist  Tailored to pre-identified barriers: Barriers identified were divided into opportunity barriers and motivation barriers.  Opportunity barriers identified through an assessment of physical resources using a standardized tool, and activity mapping exercises and direct observation of patient flow were:  1. Lack of designated areas for emergency care;  2. Limited staffing, and limited equipment for vital sign collection  Motivation barriers identified through focus groups were: 1. Sub-par skills in recognition and resuscitation of severely ill patients  Staff at each facility developed a site-specific quality improvement plan to address the barriers they’d identified  during collaborative improvement meetings:  There was clinic-level input from local clinicians on site-specific quality improvement plans | | Targets: Professionals  Intervention: Multifaceted  Type: PROFESSIONAL: Educate groups about guideline intent/benefits + Provide feedback on compliance + Recruit an opinion leader who recommends implementation + Tailor guideline; FINANCIAL (Health professional): Grant or allowance to group/institution (not tied to compliance); STRUCTURAL CHANGES: Information/communication technology  Content:  Training phase: Training on the WHO’s Quick Check triage tool  Post-training:  A) Collaborative improvement meetings  B) Clinical performance audits and feedback  C) Clinical mentoring  Training phase: Recognition and management of emergency vital signs, management of severe illness Post-training: A) Systematic assessment of local resources for severe illness management, externally supervised goal setting by facility stakeholders, group problem-solving B) Monitoring of clinical performance through medical record, mentored review of regular performance indicator reports c) Bedside teaching rounds and assistance in reviewing clinical cases; simulation sessions for medical ward teams  Format: Collaborative improvement meetings, clinical performance audits and feedback, clinical mentoring  Delivery: In-person, chart review, email, SMS  Timing:  Training Phase: 5 days Post-training: A) One hour every 2-4 weeks/site B) Email and SMS reports 1x/week C) Ever 4-6 months/site  Personnel: Training phase: Local facilitators trained by an international NGO Post-training: A) Local champion at the hospital, clinical leader B) Onsite data collectors and intervention program managers using data collection platform delivered to clinical leaders via email, and via SMS to individual staff C) Shadowing the visiting expert clinician at bedside teaching rounds | Positive results  Significant increases in collection of all vital signs, and diagnosis of 3 of 4 severe illness conditions  Significant increases during intervention period in collection of (% increase [95% CI], *P* value):   - Temperature (27% [11, 43%], *P* = 0.001) - Heart rate (22% [12, 32%], *P* < 0.001) - Blood pressure (15% [8, 21%], *P* < 0.001) - Respiratory rate (5% [2, 8%], *P* = 0.002) - Pulse oximetry (19% [19, 20%], *P* < 0.001) - Mental status (4% [2, 7%],   *P* = 0.002)  Significant increases in diagnoses of 3 severe illness conditions in intervention period (% comparison, risk ratio [95%CI], *P* value):   - Sepsis: 0.4% vs. 4.3%, RR 10.1 [3.3, 30.7], *P* = 0.001 - Severe respiratory distress: 0.9% vs. 3.9%, RR 4.5 [1.8, 10.9], *P* = 0.001 - Shock: 10.8% vs. 16.7%, RR 1.5 [0.9, 2.5], *P* = 0.09   Significantly higher diagnosis of altered mental status in pre-intervention period: 5.2% vs. 3.5%, RR 0.7 [0.6, 0.8], *P* < 0.001 |
| Eccleston [63]  2017  Australia  Secondary prevention strategies (statins and anti-platelet therapy) after percutaneous coronary intervention in private hospitals | Cross-sectional study with real-time benchmarking within inter- and intra-hospital system  Registry on the whole collected data from 2009 onwards from 6720 patients who underwent 7167 coronary interventions  Study compared only 2010 and 2014 patients  Baseline patients (year 2010): 1744  2014 patients: 1467 | Quality Improvement  To investigate the impact of a national clinical quality registry for percutaneous coronary interventions on patient outcomes | Theory: Comparative Effectiveness Research  Tailored to pre-identified barriers: NR  Co-designed by patients or professionals: NR | | Target: Professionals  Intervention: Single  STRUCTURAL CHANGES: Information/communication technology + Quality improvement, performance measurement system  Content: Collaborative, centralized national quality register providing information on clinical characteristics, management, in-hospital and long-term outcomes, compliance with secondary prevention treatments in patients undergoing percutaneous coronary interventions.  Hospital case report forms aggregated and sent back with accompanying metrics: benchmarked key performance indicators and process measures  Format: National clinical registry  Delivery: Online  Timing: Initiated in 2009  Personnel: A steering committee | Mixed results  Effect of the registry was positive on patient medication compliance at discharge and 1 year post-discharge  However, no significant differences between 2010 (n = 1744) vs. 2014 (n = 1467), respectively, in the following patient outcomes:   - In-hospital death: 0.7% vs. 0.3%, *P* = 0.13 - In-hospital myocardial infarction: 1.1% vs. 0.84%, *P* = 0.39 - In-hospital hemorrhage: 0.68% vs. 0.70%, *P* = 0.94 - In-hospital major adverse cardiac events: 1.3% vs. 1.2%, *P* = 0.52 - 30-day unplanned readmission: 3.6% vs. 3.2%, *P* = 0.28 - 30-day major adverse cardiac events: 1.3% vs. 1.2%, *P* = 0.75   Significantly more patients treated in 2014 vs. 2010 were fitted with a drug-eluting stent: 68.8% vs. 75.7%, *P* < 0.001  Significant differences between 2010 and 2014 in at-discharge medication compliance rates for:   - Statins: 92.1% vs. 94.4%,   *P* < 0.03   - Beta-blockers: 57.1% vs. 61.5%, *P* < 0.01 - ACE inhibitors/ARBs: 73.5% vs. 76.2%, *P* = 0.06   No significant differences between 2010 and 2014, respectively, in at-discharge medication compliance rate for antiplatelet drugs: 98.7% vs. 99.1%, *P* = 0.25  Significant differences between 2010 and 2014 in 1-year post-discharge medication compliance rates for:   - Statins: 87.0% vs. 92.2%,   *P* < 0.001   - Antiplatelets: 90.7% vs. 94.3%, *P* < 0.001 |
| Jordan [64]  2017  UK  Osteoarthritis or joint pain in primary care | Cluster RCT  8 practices with GPs and nurses seeing 1960 patients with peripheral joint pain  Intervention group: 4 practices with GPs and nurses seeing 1118 patients  Control group: 4 practices with GPs and nurses seeing 842 patients | Context: Quality improvement  To determine the effect of new mode of osteoarthritis consultation on GP and nurse provision of 14 care quality indicators compared to usual care | Theory: Whole Systems Informing Self-Management Engagement (WISE) Model, Behaviour Change Wheel, Theoretical Domains Framework, Normalisation Process Theory (from study protocol Dziedzic et al. 2014), Calgary-Cambridge Framework (from study protocol Dziedzic et al. 2014), Grol’s approach to translating evidence to practice (from study protocol Dziedzic et al. 2014; Grol, 1997)  Tailored to pre-identified barriers: NR  The intervention itself was co-designed by patients and professionals through consensus rounds on what they considered important elements of an osteoarthritis consultation  Lay advisors from a local arthritis support group and a Research User Group (composed of people who have chronic musculoskeletal conditions) co-designed the osteoarthritis guidebook that formed part of the intervention, through 3 group discussions (component) (Grime and Dudley, 2014) | | Target: Patients and professionals  Intervention: Multifaceted  Type: PROFESSIONAL: Distribute guideline material; PATIENT: Education + Counselling + Print material (guidebook); STRUCTURAL CHANGES: Information/communication technology + Method of service delivery  Content:  Patient-geared:  Explanation of osteoarthritis in layperson language, prescription of first-line analgesics as appropriate, support for self-management, promotion of core treatments, accounts of how other people live with osteoarthritis, provision of guidebook; explanation of guidebook, negotiation of patient-specific goals, discussion of other lifestyle changes  General practitioner and nurse-geared:  Practice updates on diagnosis, advice, patient self-care, overview of guidebook  Format: Enhanced consultations, practice-based sessions, training sessions, guidebook  Delivery: In person, printed material  Timing: 6-month run-in period  General-practitioner-geared: 4 practice-based sessions (Three 2-hour and one 1-hour sessions)  Nurse-geared: 4 training days  Patient consultation with nurse within 3 months of general practitioner consultation  Personnel: Study coordinators  Control: No training, guidebook or dedicated nurse-led osteoarthritis clinic | Mixed results  There were no statistically significant differences between intervention and control practices in the recorded achievement of 13/14 quality indicators (*P* = NR for all) except for X-ray requests, which reduced in the intervention arm (25 to15%), in line with guidelines, and increased in the control arm (3-6%, OR 0.45; 95% CI 0.12 to 1.72)  Among the 6 quality indicators based on NICE recommendations, there were significant (guideline-concordant) improvements in 3 indicators. There were increases in written exercise advice and written weight loss advice provided to those overweight in intervention practices (baseline: n = 615, trial period: n = 698) compared to control practices (baseline: n = 470, trial period: n = 439), respectively:   - Written exercise advice: (Baseline: 59% vs. 38%; Trial period: 50% vs. 32%; OR 1.53 (95% CI 0.75 to 3.13)) - Written weight loss advice: (Baseline: 53% vs. 34%; Trial period: 49% vs. 31%; OR 1.24 (95% CI 0.61 to 2.52))   As well, the rate of the healthcare provider supplying written information specifically on osteoarthritis, another NICE-based quality indicator, increased in intervention practices from 4% of patients in the baseline period to 28% in the trial period with no change in the control practices (1-2%, OR 23.60, 95% CI 7.39 to 75.40)  Paracetamol prescribing increased from the baseline period in the intervention arm (16% to 22%) and decreased in the control arm (19 to 14%, OR 1.74; 95% CI 1.27 to 2.38) |
| Kersten [65]  2017  Netherlands  Expectant management in fertility care | Cluster RCT  25 clinics in the Netherlands (number of fertility doctors, GPs, and in-training gynecologists NR) treating 544 couples  Intervention group: 300 couples  Control group: 244 couples | Context: Quality improvement  To determine the effectiveness of a multifaceted implementation strategy compared to usual care on adherence to guideline recommendations for expectant management in couples with unexplained fertility | Theory: NR  Barriers and facilitators were pre-identified by the study team through a barrier analysis starting with a nationwide survey. Specific barriers and facilitators were identified through questionnaires sent to both patients and professionals. The barriers identified were many (van den Boogaard et al., 2012), but the main ones among couples include:   1. Lack of confidence in natural conception 2. Perception that expectant management was a waste of time 3. Unrealistic expectations prior to the first secondary care consultation 4. Overestimation of success rates of treatment   (van den Boogaard et al., 2013)  The main barriers identified relating to professionals include:   1. Limited knowledge about prognostic models 2. Inadequate communication skills to convince the couple of the utility of not pursuing treatment   (van den Boogaard et al., 2013)  Barriers identified by both patients and professionals relating to patients was:   1. Lack of patient informational materials   (van den Boogaard et al., 2013)  The intervention was tailored to pre-identified barriers.  Co-designed by patients and professionals:  The counselling skills of the professional, and elements of intervention targeted at patients were developed with patients; clinic-level action plans were developed with professionals during the educational outreach visits that were part of the intervention | | Target: Patients and professionals  Intervention: Multifaceted  Type: PROFESSIONAL: Educate individuals about guideline intent/benefits + Provide feedback on compliance + Tailor guideline; PATIENT: Education + Counselling + Print material (leaflet); ORGANIZATIONAL (Patient): Consumer feedback, suggestions, complaints; STRUCTURAL CHANGES: Information/communication technology + Method of service delivery  Content:  Patient-geared: Access to an informational website, leaflet on what could be found on website  Professional-geared: Audit and feedback of the clinic-level baseline performance; discussion of feedback, background information on expectant management, local barriers, generation of action plan; access to patient-geared website; summary of the guideline recommendations; training on counselling skills on expectant management; local protocol based on the guideline recommendations.  Format: Educational outreach visit to each clinic, Website, Pocket cards with guideline summary, E-learning with videos on patient counselling, Patient leaflets  Delivery: In-person, online, static and dynamic online content  Timing: Intervention period March 2013-May 2014 (15 months)  Personnel: NR  Control: Usual care | No change  No significant increase in the adherence to the guideline regarding expectant management compared to care as usual in the primary outcome:   - Percentage of couples that were advised an expectant management period of at least 6 months and completed the advised period or had a natural conception in the expectant period: (Intervention group: 48.7% at baseline (N = 300) vs. 69.2% (N = 247) post-intervention; Control group: 48.8% at baseline (N = 244) vs. 60.9% (N = 238); Adjusted OR of intervention: 1.31 [95% CI 0.67, 2.60])   Secondary outcomes: there was a significant improvement in 1/3 quality indicators:   - Prognosis of natural conception was calculated: (Intervention group: 58.7% at baseline vs. 85.0% post-intervention; Control group: 74.6% at baseline vs. 80.7%; Adjusted OR of intervention: 2.61 [95% CI 1.44, 4.71])   There were no significant improvements in the other 2 quality indicators:   - Correct course of treatment was advised: (Intervention group: 58.0% at baseline vs. 76.5% post-intervention; Control group: 57.4% at baseline vs. 67.6%; Adjusted OR of intervention: 1.26 [95% CI 0.63, 2.52]) - Correct course of treatment was advised and couples did *not* start fertility treatment within 6 months: (Intervention group: 85.1% at baseline vs. 90.5% post-intervention; Control group: 85.0% at baseline vs. 90.1%; Adjusted OR of intervention: 1.2 [95% CI 0.52, 2.73])   No difference in treatment outcomes measures (of which there are several) between intervention and control groups |
| Lesuis [66]  2017  Netherlands  Treatment of rheumatoid arthritis | Pilot RCT  20 clinicians (rheumatologists, residents, physician assistants), random sample of 4648 unique patients: 990 patients altogether  Extended intervention group: 10 clinicians, 482 patients/visits  Standard intervention group: 10 clinicians  508 patients/visits | Context: Quality improvement  To determine the effect of education, feedback, and electronic decision support vs. education and feedback alone on proper treatment of rheumatoid arthritis | Theory: NR  Tailored to pre-identified barriers: As noted in Supplement 1, a Cochrane framework was (Cochrane et al., 2007) used for an informal barrier analysis, as a result of which up-to-date treatment guidelines were presented, and an electronic decision support was instituted in response to the persistence of Cochrane’s ‘barriers related to support or resources’  The same study centre had previously invested in investigating determinants of change and had created updated guidelines, improved knowledge and attitudes among staff, and had created a safe learning environment.  Co-designed by patients or professionals: NR | | Target: Professionals  Intervention: Multifaceted  Type: PROFESSIONAL: Present guideline material at meetings + Educate groups about guideline intent/benefits; STRUCTURAL CHANGES: Information/communication technology  Content: Education and feedback session:  Education—Background information on importance of tight control strategies for rheumatoid arthritis, importance of guideline adherence, content of local treatment guideline especially biological dose optimization  Feedback—Department-specific performance feedback and note made of existing feedback systems in the hospital in which department was located  Electronic decision support: Reorganization of existing computerized physician order entry system, with guidelines and follow-up made easier and more easily accessible, plus a reminder to keep up correspondence with patient’s GP  Format: Education sessions, electronic decision support  Delivery: In person, online  Timing:  Pre-intervention period: July-December 2013  Intervention period: January-April 2014  1-hour group session for education and feedback, 1.5-hour training and additional assistance for electronic clinical decision support system  Personnel: Education session: PhD student and rheumatologist, additional assistance from decision support system developers  Control or standard intervention group: Education session and feedback but no electronic decision support | Mixed results  Aggregate effect of both standard intervention and extended intervention: There was no significant difference in standardized sum scores pre- and post-intervention, when taking into account all indicators, between the standard intervention and extended intervention groups (mean standardized sum score difference 0.02 [95% CI -0.04, 0.08; *p* = 0.60)  There was a significant increase pre- vs. post-intervention in the standardized sum score of all indicators in the standard intervention group (mean standardized sum score difference [95% CI], *p*-value): 0.06 [0.02, 0.11], *p* < 0.01  Significant differences (mean standardized sum score difference [95% CI], p­-value) pre- vs. post-intervention within *standard intervention group* in clinical-decision-support-only-related indicators (0.08 [0.03, 0.13], *p* < 0.01) (*though the standard intervention had no decision support*) and follow-up-and-referral-only indicators (0.06 [0.002, 0.12], *p* = 0.04); no differences in rheumatologist-only, monitoring-and-treatment-only, or administration-only indicators  There were no differences when comparing the pre- vs. post-intervention scores of the standard intervention and extended intervention groups in any of the above indicators  Three of thirteen guideline indicators showed significant post-intervention improvement in the standard intervention group (there were no significant differences between the standard and extended interventions):   - DAS28 measurement: 66.8% (n = 216) vs. 80.3% (n = 228), OR 2.0 (95% CI 1.3 to 3.1) - Referral to a Physician Assistant: 22.1% (n = 140) vs. 39.2% (n = 97), OR 2.4 (95% CI 1.3 to 4.4) - Yearly Health Assessment Questionnaire: 68.5% (n = 216) vs. 79.4% (n = 228), OR 1.8 (95% CI 1.1 to 2.7)   and one worsened significantly post-intervention in the standard intervention (no difference between the standard and extended intervention):   - Radiographs: 48.4% (n = 64) vs. 13.2% (n = 53), OR 0.2 (95% CI 0.1 to 0.4)   Results suggest that the decision support system did not have an effect on guideline adherence, whereas education and  feedback led to a small but significant improvement on adherence |
| Liebschutz [67]  2017  USA  Long-term opioid therapy in primary care | Cluster RCT  4 sites with 53 primary care physicians and 985 patients on undergoing long-term opioid therapy  Intervention group (randomized by site): 28 primary care physicians overseeing 586 patients  Control group: 25 primary care physicians overseeing 399 patients | Context: Quality improvement  To determine whether a multicomponent intervention consisting of:   1. A nurse care manager 2. Electronic registry 3. Academic detailing 4. Electronic decision tools   improves adherence to opioid-prescribing guidelines and reduce rates of early refills among chronic pain patients, as compared to an electronic decision tool alone | Theory: Nurse care management model, motivational interviewing  Barriers to guideline-concordant care identified on an individual basis with each primary care physician during academic detailing (from study protocol, Lasser et al., 2016)  Co-designed with patients or professionals: NR | | Target: Professionals  Intervention: Multifaceted  Type: PROFESSIONAL: Provide feedback on compliance + Provide feedback about patients + Provide feedback from healthcare professionals+ Educate individuals about guideline intent/benefit + Recruit an opinion leader who recommends implementation; ORGANIZATIONAL: (Health professional): Additional human resource (nurse care manager); STRUCTURAL CHANGES: Information/communication technology + Method of service delivery  Content:  Component 1, Nurse care management: Primary care physicians received nurse care management – nurse would prepare prescriptions to sign, collect of urine drug tests, conduct pill counts, check prescription drug monitoring program, assist with patient issues.  Component 2, Electronic registry:  Electronic registry producing reports related to workflow (e.g., lists of patients with opioid prescriptions due at a certain time), and supporting academic detailing; change of workflow for obtaining opioid refills  Component 3, Academic detailing:  A one-on-one academic detailing session with a physician expert in public health with discussion of safe opioid prescribing and monitoring, details and advice on certain patient cases  Component 4, Electronic decision tools:  Orientation and access to electronic decision support tools for assessment of opioid misuse risk and assistance with test ordering and interpretation  Format: Nurse care management, electronic registry, academic detailing, electronic decision support  Delivery: In person, electronic health record, online  Timing:  Academic detailing consisted of one 45- to 60-minute visit per primary care provider 2-3 months after study implementation  Primary outcomes observed over 12 months  Study took place between January 2014-March 2016  Personnel: Nurse care management provided by a nurse; academic detailing delivered by experts in addiction and pain medication management (one of 3 study authors who are medical doctors with Master’s degrees in public health)  Control: Orientation and access to component 4: electronic decision support tool only | Positive impact  Intervention resulted in significant differences in intervention group in all primary outcomes except frequency of early refills  At 12 months, intervention patients were more likely to have had (% comparison, *P*-value; adjusted odds ratio [95%CI]):   - Guideline-concordant care: 65.9% vs 37.8%; *P* < 0.001; 6.0 [3.6, 10.2] - Treatment agreement (of 376 without an agreement at baseline): 53.8% vs. 37.8%; *P* < 0.001; 11.9 [4.4, 32.2] - At least 1 urine drug test: 74.6% vs. 57.9%; *P* < 0.001; 3.0 [1.8, 5.0]   No difference between intervention and control groups in odds of early refills (24.7% in intervention group vs. 20.1% in control group; *P* = 0.82; adjusted odds ratio 1.1 [95% CI 0.7-1.8])  Secondary outcomes:  Significantly greater rates in intervention group compared to control group in (% comparison, *P*-value; adjusted odds ratio [95% CI]):   - Opioid dose reduction: 32.8% vs. 22.9%; *P* = 0.002; 1.6 [1.1-2.4] - Opioid dose reduction or discontinuation: 47.1% vs. 35.8%; *P* < 0.001; 1.6 [1.3-2.1]   Adjusted analyses showed that intervention group patients had a mean morphine-equivalent daily dose 6.8 mg (standard error 1.6) lower than that of the control group in the last 30 days of the intervention period (*P* < 0.001)  No significant differences between groups in (% comparison, *P*-value; adjusted odds ratio [95% CI]):   - Discontinuation of opioid prescription: 21.3% vs. 16.3%; *P* = 0.08; 1.5 [1.0-2.1] |
| Lilih [68]  2017  Netherlands  Gastrointestinal bleeding prophylaxis | Cross-sectional, pre- post  1898 inpatients and outpatients with 4333 prescriptions warranting gastrointestinal prophylaxis  Pre-intervention period: 932 patients with 2064 prescriptions  Post-intervention period: 966 patients with 2269 prescriptions | Context: Quality improvement  To determine the whether a clinical decision support system improved compliance with guideline for gastrointestinal prophylaxis, and resulted in lower numbers of drug safety alerts in the hospital’s electronic system | Theory: NR  Tailored to pre-identified barriers: NR  Co-designed by patients or professionals: NR | | Target: Professionals  Intervention: Single  Type: STRUCTURAL CHANGES: Information/communication technology  Content: Alert indicating the risk for gastrointestinal bleeding resulting from a risk-conferring medication and the risk factor of patient age, with advice to order a gastrointestinal bleeding prophylactic, and an order set for the prophylactic, with an option to override the alert with a reason  Format: Best practice pop-up alert, order set  Delivery: Clinical decision support system within electronic health record  Timing: Pre-intervention period: 1 May 2014-1 August 2014 (3 months)  Post-intervention period: 1 December 2014-1 March 2015 (4 months)  Personnel: NR  Control: Pre- data | Positive impact  Pre-intervention, gastrointestinal bleeding prophylactics were co-prescribed with risk-conferring medication in 84.0% of the 2064 prescriptions warranting prophylaxis  Post-intervention, there was a significant increase in the rate of gastrointestinal bleeding prophylactic co-prescription (94.5%) among the 2269 prescriptions warranting prophylaxis,  *p* < 0.001  Gastrointestinal prophylaxis was also prescribed more frequently post-intervention (44.7%) in response to a drug safety alert compared to pre-intervention (4.4%)  During the pre-intervention period, 812 drug safety alerts popped up in the hospital system, and 91 (11.2%) were correct according to the guideline  Of the 2064 prescriptions assessed during the pre-intervention period, 244 (11.8%) would have warranted a drug safety alert pop-up, but no alert was shown to the provider  After the new decision support system was implemented, 217 drug safety alerts popped up, and all were correct according to the guideline  Of the 2269 prescriptions assessed during the post-intervention period, only 4 (0.2%) warranted a drug safety alert pop-up, with no alert shown  Pre-intervention:  False positives: 88.8%  Misses: 100%  Post-intervention:  False positives: 0%  Misses: 0.2%  There was also a 78.2% reduction in the number of drug safety alerts post-intervention, which the authors note is good because it ensures providers remain sensitive and responsive to the appearance of such alerts |
| Lin [69]  2017  USA  Opioid prescribing for chronic pain | Interrupted time-series analysis  Patients seen at 141 veterans’ health facilities | Context: Quality improvement  To determine whether a point-of-care electronic tool – part of a nationwide veteran opioid surveillance system – improved safe opioid prescribing, including reducing prescriptions of high opioid dosages, or co-prescribing benzodiazepines | Theory: NR  Tailored to pre-identified barriers: NR  Co-designed by patients or professionals: NR | | Target: Professionals  Intervention: Multifaceted  Type: PROFESSIONAL: Recruit an opinion leader who recommends implementation + Enable self-audit + Provide feedback from healthcare professionals + Provide feedback on compliance; STRUCTURAL CHANGES: Information/communication technology + Quality improvement/performance measurement system  Content: Aggregation of electronic health record data, information on high-dosage opioid prescribing and benzodiazepine co-prescribing; key leader at each facility championed the opioid safety initiative, identifying prescribing irregularities and giving feedback to providers to promote safe opioid prescribing while taking into account the unique needs and case mix of patients in a particular facility  Format: Electronic data-capture tool, performance audit and tailored feedback  Delivery: Electronic, in person  Timing: Baseline to study end: October 2012-September 2014 (2 years)  Pre-intervention period: October 2012-September 2013  Post-intervention period: October 2013-September 2014  Personnel: One key leader per facility (qualifications NR)  Control: Pre-intervention data | Positive impact  Prior to the intervention, there was already a declining trend in opioid prescriptions of over 100 morphine-equivalent mg, and in concurrent benzodiazepine prescribing  The paper reports significant decreases in all 3 outcomes examined, though *p­*-values are not reported for any  Of all outpatients being prescribed opioids, a 0.04%/month [95%CI -0.06 to -0.02] decrease in the proportion of those receiving >100 morphine-equivalent mg can be attributed to the intervention  Similarly, a 0.02%/month [95%CI -0.03 to -0.01] decrease in the proportion of opioid patients receiving >200 morphine-equivalent mg can be attributed to the intervention  The intervention was also associated with a 0.11%/month decrease in the percentage of opioid patients being co-prescribed benzodiazepines with opioids |
| Löwe [70]  2017  Germany  Treatment of somatoform disorders | Controlled cluster cohort study  Mental health network made up of primary care providers, psychotherapists and mental health clinics  18 intervention practices with 41 primary care providers, 35 psychotherapists, 7 inpatient mental health clinics with 119 patients  15 control practices including 26 primary care providers with 100 patients | Context: Quality improvement  To assess effect of the institution of a guideline-based specialized mental healthcare network for patients at risk of somatoform disorder | Theory: Stepped, collaborative, coordinated care model  Barriers related to somatoform-type disorder were pre-identified in a previous study through a systematic review process and included patient-related, physician-related, doctor-patient interactional, situational, and conceptual and operational barriers (Murray et al., 2016)  Collaborative care model was tailored to pre-identified barriers  Co-designed by patients or professionals: NR | | Target: Patients and professionals  Intervention: Multifaceted  Type: PROFESSIONAL: Present guideline material at meetings; PATIENT/CONSUMER: Education + Print material (booklet); ORGANIZATIONAL: (Health professional): Additional human resources (access to broader team and patient handoff to another professional if referred) + Communication between distant health professionals; STRUCTURAL CHANGES: Organizational structure (including reorganization) + Information/communication technology + Method of service delivery + Integration of services  Content:  1. Creation of network structure and elements:   - Early diagnosis: Screening in primary care practices - Early treatment: Guideline-informed stepped care approach; outpatient clinic consultations within 4 weeks; Psychotherapy or inpatient treatment referrals within 8 weeks - Network pathways: Patient referrals through email or directory; Enhanced communication efficacy - Network-wide treatment improvement: Coordinating centre; network and quality centre meetings - Patient education: Patient booklet; special website and access, group psychoeducation   2. Inducting network partners into structure: Introduction to structure, network elements, guidelines for somatoform disorders; visits to each practice  Format: Mental healthcare network, modified workflow: regimented patient visits and specialized referrals, meetings, booklet, website, group education sessions  Delivery: In person, online, printed material  Timing: Control practices studied before intervention practices, Follow-up at 6 months  Personnel: Primary care providers, psychotherapists  Control: Care as usual | Mixed results  One healthcare-provider-related primary outcome, receipt of mental health treatment since the start of the network and follow up (30 months) was higher among patients in intervention practices: 47.9% vs. 31.0%, *p* = 0.029, OR 1.96 [95%CI 1.07 to 3.58]  No significant differences between intervention and control practices, respectively, in second healthcare-provider-related primary outcome, receipt of mental health treatment between screening and follow-up (6 months): 14.3% vs. 10.2%, *p* = 0.755, OR 1.15 [95%CI 0.48 to 2.76]  No significant differences between intervention and control practices, respectively, in any secondary outcomes (all pertaining to number of visits to each care provider in past 6 months):   - Primary care physician visits: 5.7 [95%CI 4.5 to 6.9] vs. 5.5 [95%CI 4.1 to 6.9], *p* = 0.353, OR 1.14 [95%CI 0.86 to 1.51] - Somatic specialist visits: 6.8 [95%CI 5.3 to 8.3] vs. 6.8 [95%CI 5.3 to 8.4], *p* = 0.128, OR 1.26 [95%CI 0.93 to 1.71] - Mental health specialist visits: 3.0 [95%CI 1.7 to 4.3] vs. 2.5 [95%CI 1.1 to 4.0], *p* = 0.674, OR 1.13 [95%CI 0.64 to 2.00]   At patient level, no significant differences in 6-month score changes from baseline between intervention and control practices respectively in 2 of 3 secondary outcomes, somatic symptom burden (-3.4 [95%CI -4.2 to -2.5] vs. -5.0 [95%CI -6.0 to -4.0], *p* = 0.110, between-group effect size Cohen’s *d* = 0.22) or anxiety severity (-2.8 [95%CI -3.8 to -1.9] vs. -3.0 [95%CI -4.2 to -1.8], *p* = 0.300, between-group effect size Cohen’s *d =* 0.18), but significantly lower depression rates in *control practices* (-3.7 [95%CI -4.6 to -2.7] vs. -5.0 [95%CI -6.0 to -3.9], *p* = 0.011, between-group effect size Cohen’s *d* = 0.36) comparing baseline and six-month points  Healthcare network was effective at the healthcare provider level as high-risk patients were referred to mental healthcare more often than in the usual care practices  Of the 82.5% of professionals who participated in rating the network, 80% were satisfied with the network |
| Patil [71]  2017  India  Antiemetics during chemotherapy at tertiary cancer centre in India | Retrospective cross-sectional, pre- post  Pre- data: 1211 consecutive antiemetic prescription records  Post data: 201 consecutive prescriptions | Context: Quality improvement  To determine whether clinician education and a revision of hospital antiemetic policy would improve appropriate use of antiemetics in chemotherapy patients | Theory: NR  Tailored to pre-identified barriers: NR  Co-designed by patients or professionals: NR | | Target: Professionals  Intervention: Multifaceted  Type: PROFESSIONAL: Present guideline material at meetings + Educate groups about guideline intent/benefits + Provide alerts when practice deviates + Provide feedback on compliance + Provide feedback from healthcare professionals + Enable self-audit; ORGANIZATIONAL (Health professional): Reallocated or new role (double-check mechanism); STRUCTURAL CHANGES: Quality improvement, performance measurement system  Content:  1. Audit: Initial audit of 1211 consecutive antiemetic prescriptions, those prescriptions classified as guideline adherent were classified as optimal or overuse (when an additional unnecessary antiemetic was given), or when non-adherent, classified as a major deviation (no antiemetic, or only in part) or minor deviation (insufficient dose);  2. Clinician education: Audit results shared and feedback provided, with guideline recommendations revised during the feedback sessions;  3. Hospital policy revised: All relevant protocols updated to be guideline-concordant; use of antiemetics made compulsory;  4. Double-check mechanism: Two additional clinicians to check prescriptions, re-sent to original prescribing clinician for rectification if non-compliant  Format: Chart review, audit and feedback, education session, revised hospital policy, self-audit mechanism  Delivery: Chart review, in person, printed material  Timing: Initial audit data collected July-August 2015  Re-audit (post) data collected September-October 2016  Personnel: NR  Control: Initial audit (pre-) data | Positive impact  Of 1211 prescriptions initially audited, 770 (63.6%; [95% CI 60.8% to 66.2%]) prescriptions considered guideline-adherent, of which 588 (48.6%; [95% CI 45.7% to 51.4%]) considered guideline-adherent but with overuse  Initial audit (pre- data) showed that compared to non-adherent prescriptions, guideline-adherent prescriptions were significantly associated with lower rate of vomiting (6.6% vs. 21.9%;  *P* < 0.001), emergency visits (2.6% vs. 5.8%; *P* = 0.006), and hospitalization for emesis (0.9% vs. 4.9%; *P* < 0.001)  Re-audit (post data) showed 98.5% of prescriptions were guideline-adherent, a significant improvement from initial audit (63.6%, *P* < 0.001)  At re-audit, proportion of patients in whom antimetics were guideline-adherent but overused was restricted only to patients who’d had chemotherapy with a low risk of emesis potential to begin with. When compared with initial audit levels of overuse prescriptions for patients who’d had such chemotherapy, there was nonetheless a significant reduction such prescriptions: (68.3% vs. 41.3%; *P* = 0.001) |
| Tahvonen [72]  2017  Finland  Use of spine radiography, CT and MRI in primary care | Prospective cross-sectional, pre- post  140 practitioners in health centres and staff (number NR) working in a radiology department | Context: New guideline  To determine whether guideline distribution + education could reduce requests for spine imaging in primary care and whether the examinations performed post-intervention would be justified | Theory: NR  Tailored to pre-identified barriers: NR  Co-designed with patients or professionals: NR | | Target: Professionals  Intervention: Multifaceted  Type: PROFESSIONAL: Educate individuals about guideline intent/benefits + Distribute guideline material + Provide reminders to individuals/groups about guideline intent/benefits + Provide feedback on compliance (cancellation of radiography request)  Content: Guideline distribution; written information on harmful effects of ionizing radiation, guideline intent, cancellation of unjustified spine radiography requests; lecture on risks and doses of radiation, spinal exam indications, referrals, justification process for radiography requests, legislation on radiation protection, email reminders about project and guidelines  Format: Educational ‘cover letter’, Educational lectures, electronic format guidelines, informational pocket cards, email reminders  Delivery: In person, hospital intranet, email, printed material  Timing: 1-hour educational lectures weekly from February to March 2011  Email reminders about guideline February 2011, June 2011; guideline redistribution October 2011, June 2012  6-month study periods—  Pre-intervention period: 1 May 2010-31 October 2010  Post-intervention periods: 1 May 2011-31 October 2011 and 1 May 2012-31 October 2012  Personnel: Educational lecture by two radiologists; radiographer evaluated justification of radiography requests | Positive impact  Significant differences in rates of all radiography; spine radiography; and cervical, thoracic, and lumbar spine radiography from 2010 to 2011, and 2010 to 2012, all  *P* < 0.001, except for all radiography rates between 2010-2011, which were not significantly different (% change -0.7, chi-square 0.204, *P* = 0.652)  Number of spine examinations decreased by 8% from 2010-2012 (*p* = NR)  Of radiography of cervical spine, thoracic spine, and lumbar spine, only lumbar spine radiography showed a significant increase in proportion of justified cases, from 32% in 2010 to 64% in 2012,  *P* = 0.005 |
| Trietsch [73]  2017  Netherlands  Anemia, dyslipidemia, rheumatic complaints, prostate complaints, chlamydia, thyroid dysfunction, urinary tract infections, perimenopausal complaints, stomach complaints, diabetes mellitus 2 | Cluster RCT  21 local quality improvement collaboratives/groups with 197 GPs  Intervention arm A groups picked 3 of 5 clinical guidelines: 10 groups with 86 GPs  Intervention arm B picked 3 of 5 different clinical guidelines: 9 groups with 107 GPs | Context: Quality Improvement  To determine whether audit and feedback with peer review can modify test-ordering and prescribing behaviour | Theory: Plan-Do-Study-Act (PDSA) cycles, social interaction theories which show behaviour is strongly mediated by peers  Individual- and group-level barriers were self-identified by groups in advance through discussion and independently acted upon (from study protocol, Trietsch et al., 2009)  Tailored to pre-identified barriers as noted above  Co-designed by patients or professionals: NR | | Target: Professionals  Intervention: Multifaceted   Type: PROFESSIONAL: Present guideline material at meetings + Educate groups about guideline intent/benefits + Recruit an opinion leader who recommends implementation + Achieve consensus that guideline should be implemented + Provide feedback on compliance + Enable self-audit (material); FINANCIAL (Health professional): Grant or allowance to individual (not tied to compliance); ORGANIZATIONAL (Health professional): Reallocated or new role (community pharmacist and medical coordinators) + Create an implementation/multidisciplinary team; STRUCTURAL CHANGES: Quality improvement, performance measurement system  Content:  a) Training of local pharmacist and medical coordinator to conduct feedback and planning sessions  b) Feedback and planning sessions (pharmacotherapeutic audit meetings): First sessions contained explanation of the chosen topics; subsequently, aggregated, de-identified GP-level diagnostic and prescription audit feedback was sent to groups and compared against guideline recommendations and other groups’ performance; peer review of each other’s work within groups; discussion of barriers to change; working agreements made and goals set  Feedback and planning sessions were either diagnostics-oriented or prescriptions-oriented  Format: Pharmacotherapeutic audit meetings, Written feedback  Delivery: In person, Print material  Timing:  Baseline 6 months pre-intervention  Intervention January 2008 – December 2010  Follow-up 6 months post-intervention  Training to conduct sessions: 2-3 hours  Feedback and planning sessions: 90-120 minutes each x 6 sessions  Personnel: Sessions headed either by community pharmacist or medical coordinator of each group, depending on diagnostics or prescription orientation of meeting – the other would act as a moderator | No change  GPs from practices with already-high test-ordering and prescribing quality showed the largest improvements  Comparing differences in adjusted (corrected for baseline) rates of test-ordering between intervention groups (those who had chosen to treat a certain condition) and control groups (those who had not chosen to treat that condition) at the end of the intervention showed no significant differences between groups for the following conditions:   - Anaemia: -9.1 (95% CI -96.1, 77.9), *p* = 0.829 - Rheumatic complaints: 3.2 (95% CI -8.1, 14.6), *p* = 0.551 - Prostate complaints: 6.9 (95% CI -0.7, 14.6), *p* = 0.072 - Chlamydia infections: -5.7 (95% CI -12.0, 0.5), *p* = 0.069   Comparing differences in adjusted rates of test-ordering between intervention groups (those who had chosen to treat a certain condition) and control groups (those who had not chosen to treat that condition) at the end of the intervention showed *significant guideline-concordant reductions* in test-ordering in the intervention groups for the following conditions:   - Thyroid dysfunction: 36.6 (95% CI 10.5, 62.7), *p* = 0.007 - Perimenopausal complaints: 3.2 (95% CI 0.1, 6.4), *p* = 0.046   Significant differences (baseline-adjusted) in overall volumes of prescribed drugs between intervention and control groups were observed for:   - Prostate complaints: 28.5 (95% CI 6.5, 50.7), *p* = 0.016 - Stomach complaints: 61.1 (95% CI 15.0, 107.2), *p* = 0.014 - Thyroid dysfunction: 12.6 (95% CI 0.7, 24.4), *p* = 0.040   where reduced prescribing was desired in the case of prostate complaints and stomach complaints, and intervention groups showed a comparatively smaller increase in prescribing volumes. Prescribing for thyroid medication was expected to remain the same, but increased more in the control groups relative to the intervention groups  Authors identify lack of power as a major issue in their study  Test-ordering showed guideline-concordant reductions in the intervention groups for thyroid dysfunction and perimenopausal complaints, but prescribing for prostate and stomach complaints, and thyroid dysfunction did not reduce in intervention groups, as recommended by the guidelines, but only increased less in comparison to control groups  Participant confidence in the scale and complexity of the project was low  Working agreements generated at the end of each session were not sufficiently specific, achievable, realistic, or measurable  Topics were selected for quality improvement in which group already showed good performance |
| Tunney [74]  2017  USA  Statin therapy after acute coronary syndrome | Prospective, cohort with retrospective control  238 total participants  Prospective cohort (intervention group) = 113 acute coronary syndrome patients  Historical cohort (control group) = 125 acute coronary syndrome hospitalizations | Context: Quality improvement  To evaluate the effect of a pharmacist intervention on guideline-directed statin therapy during an acute coronary syndrome hospitalization | Theory: NR  Tailored to pre-identified barriers: NR  Co-designed with patients or professionals: NR | | Target: Professionals  Intervention: Multifaceted  Type: PROFESSIONAL: Provide alerts when practice deviates + Provide feedback on compliance; ORGANIZATIONAL (Health professional): Reallocated or new role  Content: Chart review to optimize dosing and following of a telephone script during call with primary provider; rationale for lack of optimal statin dose titration documented  Format: Chart review, telephone script, phone call  Delivery: Written material, phone call  Timing: Retrospective data from November 2013-January 2014  Prospective data from December 2014- January 2015  Personnel: Pharmacists  Control: Retrospective analyses of acute coronary syndrome patients from medical records | Modest positive results  Pharmacy intervention group showed higher compliance with guideline directed statin therapy vs. historical control group at discharge, though not significant:   - Including patients with relative contraindications (86.7% vs. 77.4%, *P* = 0.06) - Excluding patients with relative contraindications: (84.8% vs. 74.5%, *P* = 0.1) |
| Vander Weg [75]  2017  USA  Smoking cessation in hospital setting | Quasi-experimental pre-post study  898 patients: veteran smokers (1+ cigarettes/day) at 4 sites  Pre-intervention: 503 patients  Intervention: 395 patients | Context: Quality improvement  To evaluate the impact of a nurse-initiated tobacco cessation intervention on provision of guideline-recommended care to hospitalized smokers | Theory: Chronic Care Model, 5As framework of smoking cessation counselling (ask, advise, assess, assist, arrange follow-up), motivational interviewing  Barriers pre-identified in the literature from study protocol (Katz et al., 2009), pertaining to veteran facilities in particular, were:   1. Inadequate education, resources and feedback for staff (Katz et al., 2009) 2. A focus on smoking cessation counselling in which participants are referred to an outpatient program (Sherman et al, 2006; Smoking and Tobacco Use Cessation Report, 2005) 3. Lack of continuity of care post-discharge, with little opportunity to promote continued abstinence (Orleans et al., 1993) 4. Varying policies in dispensing drug therapy which may contribute to underuse of effective drug therapy (Jonk et al., 2005) 5. Patients identify access problems, lack of commitment to quitting, and dissatisfaction with the group-based format of classes as responsible for lack of follow-up (Goldman et al., 2004)   Literature-identified barriers pertaining to non-veteran smokers was:   1. Delay between referral an initial smoking cessation appointment (Sherman et al., 2006); the majority of smokers relapse within a week of quitting (Zhu et al., 1996; Kenford et al., 1994) 2. Few hospitalized smokers attend smoking cessation classes after discharge (France et al., 2001)   Barriers were also pre-identified through focus groups at the site level (from study protocol, Katz et al., 2009)  Intervention was tailored to all 7 pre-identified barriers  Co-designed with patients or professionals: NR | | Target: Patients and professionals  Intervention: Multifaceted  Type: PROFESSIONAL: Educate individuals about guideline intent/benefits + Distribute guideline material + Provide feedback from healthcare professionals + Print material (pocket card); PATIENT: Education + Counselling + Print material; ORGANIZATIONAL (Health professional): Reallocated or new role (peer leaders); STRUCTURAL CHANGES: Integration of services + Information/communication technology  Content:  1. Nurse-geared academic detailing and online tutorial: performance feedback, check-ins with managers and leaders, promotion of 5As framework in <5 minutes, stage-based cessation counselling, motivational interviewing,  individualized support from research team, group feedback; pocket card with smoking cessation algorithm  2. Adaptation of electronic medical record/provision of decision support: Cues prompting 5As completion, links to patient education materials and referral forms, pre-populated order forms  3. Patient self-management support: Smoking cessation brochure, motivational video, brief bedside counselling, pharmacotherapy, access to telephone counselling with more relapse-sensitive call schedule arranged for this study’s participants  4. Peer leaders designated in each participating hospital unit and assisted colleagues in intervention implementation, problem-solving  Format: On-site and online academic detailing, adaptation of the computerized medical record, brochures, videos, brief counselling, telephone counselling via a tobacco quitline  Delivery: In person, online, video, telephone, printed material  Timing: Each clinic underwent:  Pre-intervention period: 6 months  Training and adaptation: 2 months  Intervention period: 6 months  30-minute on-site academic detailing session, 30-minute online tutorial  Group feedback provided at beginning, middle, and end of intervention period  Personnel: Physicians, nurses and health psychologists on the research team | No change  The primary outcome was 7-day point prevalence abstinence at the 6-month mark  7-day point prevalence abstinence rates during the intervention period did not differ significantly from the pre-intervention period at either 3 months (% of participants, adjusted odds ratio [95% CI] = 15.5% vs. 12.7%, AOR 0.78 [0.51, 1.18], *p* = NR) or 6 months (% of participants, adjusted odds ratio [95% CI] = 15.3% vs. 14.5%, AOR 0.92 [0.62, 1.37], *p* = NR)  No significant differences pre- vs. post-intervention in 30-day point prevalence abstinence rates at 3 months (% of participants, adjusted odds ratio [95% CI] = 11.0% vs. 8.8%, AOR 0.78 [0.49, 1.23], *p* = NR) or 6 months after the intervention (% of participants, adjusted odds ratio [95% CI] = 10.8% vs. 9.8%, AOR 0.89 [0.55, 1.44], *p* = NR)  No pre- vs. post-intervention differences in four other similar secondary outcome measures, nor in cigarettes smoked/day at the 3-month or 6-month marks |
| Wright [76]  2017  USA  Dialysis vascular access in end-stage renal disease | Quasi-experimental pre-post study  15 nurses working at outpatient hemodialysis facility (3 registered nurses, 4 licensed practical nurses, and 8 patient care technicians) | Context: Quality improvement  To examine the impact of a vascular access educational program on knowledge, beliefs, and self-confidence, and intent to abide by vascular access management guidelines; and impact on quality measures among care providers | Theory: Theory of Reasoned Action/Planned Behavior (Azjen, 2017)  Tailored to pre-identified barriers: NR  Barriers were identified by questionnaire after the intervention.  Co-designed with patients or professionals: NR | | Target: Professionals  Intervention: Single  Type: PROFESSIONAL: Educate individuals about guideline intent/benefits + Distribute guideline material  Content: Didactic component comprised management of arteriovenous fistulas/grafts and clinical guidelines; Practice component covered cannulation skills, access preservation, assessment, monitoring and surveillance techniques  Format: On-site education session and workshop  Delivery: In person  Timing: 1.5-hour session  Personnel: | Positive impact  Only 13% of participants attended the practice component of the intervention  Increase in mean knowledge scores post-intervention (12.29 vs. 9.60, *p* = 0.001)  Out of 15 knowledge items, significant increase in knowledge of:   - Hemodialysis adequacy: 26.67% vs. 86.67%, *p* = 0.003 - Alcohol antiseptic: 60.00% vs. 86.67%, *p* = 0.046   28% decrease in the incidence of vascular access complications in the 2 months post intervention (*p* = NR); urea reduction ratio (URR) for hemodialysis adequacy increased 1% (*p* = NR).  There was a significant improvement in normative beliefs (*p* = 0.036), and borderline significant improvements in subjective beliefs (*p* = 0.099) and control beliefs (*p* = 0.097) and no change in self-confidence or intentions, as measured by questionnaires  However, there were significant correlations between intentions and subjective beliefs (*r* = 0.688,  *p* = 0.004), normative beliefs  (*r* = 0.712, *p* = 0.003), and control beliefs (*r* = 0.571, *p* = 0.026) |
| Aakhus [77]  2016  Norway  Managing elderly patients with depression | A pragmatic cluster randomised trial  One hundred twenty-four general practitioners participated,  51 in the intervention group and 73 in the control group. 134 patients completed the questionnaire. Patients were home-dwelling elderly, 65 years or older, with a diagnosis of mild,  moderate or severe depressive episode, recurrent depression  or dysthymia, who had consulted their practitioner within  the last 6 months before the intervention. | Context: New guideline  To assess whether a tailored implementation strategy would increase the extent to which general practitioners adhere to evidence-based recommendations  for managing elderly patients with depression  compared to no intervention. | Theory: A logic model was used to develop the multifaceted implementation strategy and to establish the hypothesised causal links between the components, the determinants, the recommendations and the expected improved adherence  Tailored to pre-identified barriers: The authors identified determinants  of practice for six guideline recommendations and subsequently tailored interventions to address these determinants  57 determinants of practice were grouped into 7 domains:  1. Guideline factors  2. Individual health professional factors  3. Patient factors  4. Professional interaction factors  5. Incentives and resources  6. Capacity for organizational change  7. Social, political and legal factors  These determinants were identified through:  (1) Brainstorming  (2) Structured focus groups with researchers, clinicians, nurses, and patients  (3) Open and structured individual interviews with clinicians, nurses, and patients  (4) Mailed survey of clinicians and nurses  (Aakhus et al., 2014)  Co-designed with professionals: Intervention strategies tailored to pre-identified barriers were identified partly with the input of clinicians, carers, and other stakeholders (Aakhus et al., 2014) | | Target: Patients and professionals  Intervention: Multifaceted  Type: PATIENT/CONSUMER: Print material + Education (single or group); PROFESSIONAL: Distribute guideline material + Educate groups about guideline intent/benefits + Print material + Tailor guideline  Content: Guideline-based information  Format: Outreach visits, a website presenting the recommendations and the underlying evidence, tools to manage depression in the elderly and other web- and paper-based resources for patients, their relatives, general practitioners, other healthcare professionals and health administrators, including a continuous medical education course for general practitioners.  Delivery: In-person  Timing: NR  Personnel: A reference group was developed for this project  Control: Usual care | No change  Of the 124/900 (14%) general practitioners who provided data, adherence to the recommendations was a non-significant 1.6 percentage points higher in the intervention group than in the control group (95% CI −6 to 9, *p* = 0.67).    The effectiveness of the intervention is uncertain, due to the low response rate in the data collection. However, it is unlikely that the effect was large. |
| Almatar [78]  2016  Australia  Antibiotics for community-acquired pneumonia | Cross-sectional, pre- post  120 medical doctors and 40 emergency department doctors; 21 emergency department doctors were residents or interns  398 patient records reviewed over 3 study periods:  Baseline period: 130 patients, average 26 patients/month  Hospital-wide general educational intervention period: 90 patients, average 18/month  Period during which intervention targeting the emergency department was implemented: 178 patients, average 25/month | Context: Quality improvement  To compare the effectiveness of a general educational intervention and an intervention targeted to a hospital’s emergency department in improving physician adherence to community-acquired pneumonia guidelines | Theory: NR  Barriers identified through the literature include  (Schouten et al., 2007):   - Concerns about insufficient antibiotic cover - Conflicting guidelines - Poor integration of guideline into practice - Poor organizational support of guideline integration   Barriers identified in a previous study by the study team pertaining to the hospital in the present study are (Almatar et al., 2015, Almatar et al., 2014):   - Conflicting guidelines - Difficulties calculating pneumonia score - Influence of senior doctors - Lack of guideline awareness   Intervention tailored to pre-identified barriers with the help of reviewed literature, which recommended:   - Use of an educational program (Buising et al., 2008, Serisier and Bowler, 2007) - Use of computerized decision support (Buising et al., 2008) - Implementing a clinical pathway (Marrie et al., 2000) - Feedback on concordance (Hysong et al., 2006)   and   - Face-to-face education on guidelines - Engaging opinion leaders - Providing audit and feedback (Simpson et al., 2005)   as the most effective implementation strategies for community-acquired pneumonia guidelines  The emergency-department-focused component of the intervention involved the development of a local guideline (Antibiotic Expert Group, 2010) based on national antibiotic guidelines; this guideline was developed with professionals from the respiratory, infectious diseases, general medicine and emergency departments at this hospital | | Target: Professionals  Intervention: Multifaceted  Type: PROFESSIONAL: Distribute guideline material + Advertise guideline material + Present guideline material at meetings + Educate groups about guideline intent/benefits + Provide feedback on compliance + Print material (wall posters, lanyard card); STRUCTURAL CHANGES: Method of service delivery  Content:  (1) Development and distribution of local guideline through hospital intranet, with email informing staff of the guideline’s release  (2) Educational intervention:   - On new local guideline   (3) Emergency-department-focused intervention:   - New clinical pathway with clear information about appropriate empirical antibiotic therapy for community-acquired pneumonia - Feedback on guideline adherence with detailed explanations   Format:  (1) Development and distribution of local guideline: Hospital intranet, email  (2) Educational intervention: 6 presentations, 11 group discussions, wall poster with guideline recommendations and key messages in all hospital departments, lanyard card containing guideline summary, pneumonia severity assessment tool  (3) Emergency-department-focused intervention: One-page clinical pathway, two educational sessions for nursing staff, email to all medical and nursing staff informing them of pathway, written feedback and explanations  Delivery: In person, intranet, email, printed material  Timing:  Data collected monthly between July 2012 – November 2013 (17 months)  Baseline period: 5 months  Education intervention: 5 months  Emergency-department-focused intervention: 7 months  (1) Development and distribution of local guideline: Development from November – December 2012; distribution in second week of December 2012  (2) Educational intervention: 4 presentations and 6 group discussions in second week of December 2012; 2 presentations and 5 group discussions in February 2013  (3) Emergency-department-focused intervention: took place from May – November 2013, education sessions in second week of May 2013, monthly feedback  Personnel: Education sessions led by research team with support of hospital’s antimicrobial stewardship team | Positive impact of one intervention component  Guideline adherence rate at the beginning of the baseline period was 22.2%  Guideline adherence rates fluctuated between 40.9% and 77.3% after the emergency-department-focused intervention  Mean guideline adherence rate for:   - Baseline period: 28.1% (SEM ± 1.82; SD ± 4.1) - General education intervention: 31.2% (SEM ± 3.4; SD ± 6.8) - Emergency-department-focused intervention: 61.5% (SEM ± 5.42; SD ± 14.3)   Post-hoc analysis showed significant increases in the mean guideline adherence rate after the emergency-department-focused intervention compared to the baseline period (*p* < 0.001) and the general education period  (*p* < 0.001)  There were no significant differences in mean guideline adherence rates between the baseline period and the general education period (*p* = 0.626) |
| Bautista [79]  2016  Colombia  Thromboprophylaxis after major orthopedic surgery | Systematic monitoring of quality indicators  Data from 773 patients undergoing primary joint  replacement of the hip, knee and shoulder | Context: Quality improvement  To describe the impact of a quality improvement  program for the adherence to the CPG recommendations, through a systematic monitoring and measurement of performance indicators. | Theory: NR  The study team identified barriers and devised tailored strategies to overcome them over the course of the study, but neither of these was done in advance  Co-designed with patients or professionals: NR | | Target: Patients and professionals  Intervention: Multifaceted  Type: PROFESSIONAL: Distribute guideline material + Present guideline materials at meetings + Educate groups about guideline intent/benefits + Provide feedback on compliance; PATIENT/CONSUMER: Education (single or group) + Print material; STRUCTURAL CHANGES: Quality improvement, performance measurement system  Content: Guideline-based information and related performance indicators.  Format: Team meetings, conference, educational modules and printed materials  Delivery: In-person  Timing: Monthly monitoring of indicators and 2-day staff education  Data collected between February 2012-August 2014 (19 months)  Personnel: A working group of nurses, physicians and other staff | Positive change  The combined quality improvement strategies were associated with higher compliance with the clinical practice guideline.  In the first trimester, the average of adherence was: 98.3% for medical order in the post-operative note, 60.3% for opportune administration and 67% for adherence to therapy at home. In the second trimester, the rates of adherence were 100%, 95.7% and 100% respectively.  No statistics were reported in this study |
| Bhushan [80]  2016  USA  Psoriasis | Pre-post  There were 370 dermatologists from 5 sessions, 66 of whom participated in the follow-up resulting in a response rate of 18%. | Context: New guideline  To determine if educational sessions were effective in enabling  dermatologists to improve patient care | Theory: NR  Tailored to pre-identified barriers: Barriers pre-identified from the literature (Berends et al., 2007; Wakkee et al., 2008; Lugtenberg et al., 2009; Lugtenberg et al., 2011) concerning guideline implementation in general included guideline-specific barriers (e.g., lack of knowledge, non-congruence with guidelines from other specialties, oversimplification of practice, lack of access, overly complicated or unclear guidelines)  The use of presentations was tailored to the lack of knowledge barrier, and reference materials were meant to address access and clarity concerns  Co-designed with patients or professionals: NR | | Target: Professionals  Intervention: Single  Type: PROFESSIONAL: Educate groups about guideline intent/benefits  Content: Guideline-based information and skills to treat patients.  Format: These sessions featured presentations on guideline topics, and the participants were given reference materials based on the guideline recommendations.  Delivery: In-person  Timing: NR  Personnel: Psoriasis guideline expert workgroup faculty | Positive change  Pre-intervention average rate of correct responses to case-based questions was 52.8% vs. 74.1% immediately post-intervention,  *p* = NR  54.8% of case-based questions (23/42) showed significant improvement from pre-intervention to immediately post-intervention  (*p* < 0.05)  Self-reported assessment of disease severity using various scales improved from pre-intervention to ≥6-month follow-up, *p* = NR for all:   - Body surface area: 62.4% to 73.1% - Psoriasis Area and Severity Index: 27.5% to 38.5% - Physician Global Assessment: 10.1% to 23.1%   Self-reported confidence in treating psoriasis changed from 86.7% to 89.7% to 93.8%, *p* = NR, and for psoriatic arthritis from 40.7% to 68.5% to 66.7%, *p* = NR,  from pre-intervention to immediately post-intervention to ≥6-month follow-up |
| Chen [81]  2016  Taiwan  Managing hepatitis B and C | Quasi-experimental  43286 propensity-score matched patients with chronic hepatitis B or C  Intervention group and control groups: 21643 patients each | Context: Quality improvement  To assess if financial incentives for physician adherence to guidelines improves the preventive care provided to hepatitis B or hepatitis C patients | Theory: NR  Patient barriers to getting regular ultrasound and blood tests identified in the literature include:   - Patients unaware of disease status - Limited knowledge and understanding of disease - Fear of stigma - Reluctance to receive undesired test results   (Wai et al., 2004; Tan and Cheah, 2005)  Co-designed by patients or professionals: NR | | Target: Professionals  Intervention: Single  Type: FINANCIAL (Health professional): Incentive (individual financial reward or benefit for compliance); STRUCTURAL CHANGES: Quality improvement, performance measurement system  Content: US$3 per initial enrollment; US$3/visit for maximum 2 follow-up visits per year; ultrasonography and aspartate aminotransferase/alanine aminotransferase (AST/ALT) tests required at all visits; US$15 for further screening, referral and early detection of abnormalities; $US30 for screening, referral and early detection of hepatocellular carcinoma  Format: Financial remuneration program instituted  Delivery: Electronic  Timing: 2009 to 2011  Initial enrollment + two follow-up visits/year per patient  Personnel: NR  Control: 21 643 patients who were not enrolled in the program | Positive impact  In the post-intervention period, patients enrolled with intervention doctors compared with control doctors were  significantly more likely to attend twice-a-year visits (OR = 1.23; 95% CI: 1.17 to 1.29), and receive recommended ultrasonography examinations (OR = 1.18; 95% CI: 1.12 to 1.24)  and AST/ALT tests (OR = 1.13; 95% CI: 1.07 to 1.19)  than were patients in the control group, *p* = NR  Intervention-enrolled patients were significantly more likely to receive all three recommended services (regular follow-up visits, ultrasonography, AST/ALT tests) than were the non-enrollees (OR = 1.13; 95% CI: 1.07 to 1.19,  *p* = NR)  There was no change before and after the intervention in the proportion of intervention-group patients who attended twice-a-year visits (65.3% vs. 65.3%, *P* = 1.000)  There was a significant difference before and after the intervention in the proportion of intervention-group patients who received recommended ultrasonography examinations (27.7% vs. 28.5%, *P* = 0.017)  There was a significant difference before and after the intervention in the proportion of intervention-group patients who received recommended AST/ALT examinations (46.4% vs. 41.0%, *P* < 0.001)  The proportion of patients receiving all three recommended services in the intervention group underwent no change (25.0% pre- to 25.2% post, *P* = 0.540)  In the control group, the proportion of patients receiving all three services significantly decreased from 22.7% to 21.0%  (*p* < 0.0001) |
| Goodfellow [82]  2016  UK  Weight loss for patients who have overweight or obese status | Cluster RCT  Intervention group: 12 general practices seeing 17,728 patients with obesity or overweight (number and type of professional NR)  Control group: 16 general practices seeing 32,079 patients with obesity or overweight (number and type of professional NR) | Context:  Quality improvement  To determine whether a multifaceted intervention tailored to determinants of change corresponding to four guideline recommendations:   1. Determining degree of obesity of overweight 2. Assessment of lifestyle and willingness to change 3. Management of obesity and overweight 4. Referral   would improve adherence to those guideline recommendations | Theory: NR  Barriers pre-identified through the literature were:  1. Patient barriers (NICE guidelines, 2006):   - Patient perceptions of weight loss - Social pressures such as marketing of unhealthy foods - Poor access to weight loss facilities   2. Provider barriers (Maryon-Davis, 2005):   - Psychological complexity of cases - High relapse rate - Lack of time and resources - Lack of referral options   Barriers pre-identified by the study team were (Gunther et al., 2012):   - Provider sense of helplessness   Local barriers identified during an intervention workshop were used to adapt the intervention to the local context    Determinants of change corresponding to each guideline recommendation were identified in a pilot study by the study team (Krause et al., 2014), and interventions were tailored accordingly  These determinants were identified through brainstorming, structured group discussion, and questionnaires by implementation experts, heath professionals, and patients (Krause et al., 2014); and through semistructured interviews with 7 GPs, 7 practice nurses, and 9 patients with overweight or obesity (Gunther et al., 2012)  Determinants with tailored interventions are as follows:  Provider-identified determinants:   - Acceptable ways to raise and discuss the subject of weight loss - How to measure waist circumference - How to assess willingness to change - Resources to motivate and inform patients - Lack of knowledge about proper diet and exercise - Lack of information about referral pathways   Patient-identified determinants:   - Need for a more prescriptive or structured weight loss plan   Co-designed by patients and professionals:  Interventions tailored to the above determinants were selected by a new round of implementation experts, professionals and patients; the intervention was sometimes adapted to the local context by a practice’s health professionals | | Target: Patients and professionals  Intervention: Multifaceted  Type: PROFESSIONAL: Present guideline material at meetings + Educate groups about guideline intent/benefits + Provide reminders to groups about intent/benefits + Recruit an opinion leader who recommends implementation + Print material (posters, BMI charts, weight loss plans); PATIENT/CONSUMER: Education (single) + Counselling + Print material (leaflet, information booklet with prescriptive diet, worksheets)  Content:  **Professional-geared training and resources**   - Guiding the local opinion leader - Practice-level action plan for delegation and role for practice nurses   Training sessions comprising:   - Summary of guidelines, - How to delicately discuss the topic of weight with patients, - Training in waist measurement with a live demonstration and opportunity for practice, the risks of high waist circumference, - Discussion of alternative approaches to managing patients with obesity and overweight compared to the practice’s usual methods - How to assess patient readiness for lifestyle change, sample scripts - How to calculate patient’s energy needs   Print material   - Posters with information on waist circumference measurement - Sample script for assessment of motivation and willingness to change lifestyle - Prescriptive weight loss plan to offer patients - BMI charts, dietary guidance - List of local services and referral pathways that were not known to the professionals   **Patient-geared resources**   - Poster + patients leaflet to remind patients of benefit of losing 5-10% of weight - Poster about discussing weight loss with a professional - Information booklet with portion sizes, prescriptive diet - Worksheets to record food intake   Format: Guidance of local opinion leaders through telephone calls, one practice visit, opinion-leader-led intervention workshop, training sessions + demonstration, electronic and printed posters, sample script, weight loss plan, leaflet, BMI charts, information booklet, worksheets  Delivery: In person, print material, electronic  Timing: Intervention offered between November 2013 – January 2014 (3 months)  Follow-up undertaken 9 months after intervention  Monthly telephone calls with local opinion leaders  Personnel:  Intervention workshop led by local opinion leader  Training sessions led by registered dietitian  Control: Usual care | No change  No significant difference between intervention and control groups, respectively, in primary outcome:   - number of patients offered a weight management program (13.2% vs. 15.1%, *p* = 0.53)   No differences in secondary outcomes between intervention and control groups, respectively, in:   - BMI and waist circumference measured (39.6% vs. 42.7%, *p* = 0.28) - Referral to external weight loss services (3.7% vs. 5.1%, *p* = 0.21) - Patient provided with an internal weight management program (8.7% vs. 9.6%, *p* = 0.81) - Lifestyle assessment (23.9% vs. 23.1%, *p* = 0.88) - Loss of 1kg of body weight (41.7% vs. 42.2%, *p* = 0.67)   Adjusted means for BMI and weight changes slightly favoured intervention group, though not significantly  Providers reported greater confidence in managing obesity and appreciated the resources they were provided |
| Hinds [83]  2016  USA  Blood cholesterol in patients with diabetes | Retrospective comparative study  583 patients participated in this study. The patients were 40 to 75 years old and were diagnosed with diabetes. 19% of them were exposed to the intervention. | Context: New (updated) guideline  To assess guideline adherence between 2 comparison groups: (1) patients only seen by their physicians (control group) and (2) patients seen by their physicians as well as having individual pharmacist visits (intervention group). | Theory: NR  Tailored to pre-identified barriers: NR  Co-designed with patients or professionals: NR | | Target: Patients  Intervention: Single  Type: ORGANIZATIONAL (Health professional): Additional human resources (physician only or physician + pharmacist)  Content: Additional individual pharmacist visits with medication assistance  Format: NR  Delivery: NR  Timing: Those with appointments between 1 December 2013-1 October 2014 (11 months)  Personnel: Pharmacist  Control: Patients only seen by their physicians | Mixed results  71% of those seen by a physician only (338/475) were prescribed statin therapy whereas the rate of the same was 88% in patients seen by a pharmacist + physician (95/108), chi-square 13.00, *P* = 0.003  There was no statistically  significant difference between the appropriate intensity  of statin prescribed by pharmacist/physicians (35%; 38/108) versus  physicians (32%; 154/475, chi-square 0.30, *P* = 0.58) |
| Høgli [84]  2016  Norway  Appropriate antibiotic prescribing in respiratory medicine | Pre- post  Physicians at an 18-bed respiratory medicine department in Norway in charge of 155 patients treated for community-acquired pneumonia and acute exacerbations of chronic obstructive pulmonary disease | Context: Quality improvement  To investigate whether the intervention increased prescribing of the appropriate empirical antibiotics, reduced high-dose prescribing of benzylpenicillin and shortened treatment duration, in keeping with national guidelines | Theory: NR  Barriers identified in the literature pertaining to antibiotic prescribing for hospitalized patients with community-acquired pneumonia were:   - Prescriber discomfort with short treatment duration - Lack of prescriber knowledge about studies supporting short-term treatment   (Avdic et al., 2012)  Tailored to pre-identified barriers: NR  Co-designed with interdisciplinary team of health leaders and clinicians | | Target: Professionals  Intervention: Multifaceted  Type: PROFESSIONAL: Distribute guideline material + Present guideline materials at meetings + Print material (guideline) + Provide feedback on compliance  Content: Pre-intervention audit period: Review of medication charts and patient records, determining appropriateness of:  i) empirical antibiotic prescription  ii) dose of benzylpenicillin  iii) total treatment duration  (Yes/No) as per CPGs  Feedback and implementation period: Presentation of the study goals, CPG recommendations, results of audit, discussion of audit results with a focus on areas of divergence from CPGs, distribution of pocket version of national guideline for hospital usage of antibiotics  Post-intervention audit period: Identical to pre-intervention audit, plus assessment of change in treatment as a result of intervention  Format:  Pre- and post-intervention audit periods: Paper and electronic health record review  Feedback period: In-person feedback session consisting of presentation and discussion, pocket version of guidelines  Delivery: In-person, written material  Timing:  Pre-intervention phase: 9 months  Feedback phase: 1 day-long session  Post-intervention phase: 6 months  Personnel: Principal investigator of study led feedback session, infectious disease specialist presented audit results and recommendations and facilitated discussion | Mainly positive results  i) Appropriate prescribing of empirical antibiotics:  Increase from 61.7% to 83.8%  (*P* < 0.001)  Non-significant downward pre-intervention-trend  Trend immediately post-intervention: Non-significant downward trend of 1.4%/month, *P* > 0.05  Post-intervention trend: Significant increase of 4.1% per month  (*P* = 0.02)  Trend six months post-intervention: Significant estimated increase of 45.4% (*P* = 0.002)  ii) Dose of benzylpenicillin:  CPG-concordant non-significant decrease from 48.8% to 38.6% *(P* = 0.125*)* in prescribing of high-dose benzylpenicillin  iii) Total treatment duration:  Overall mean reduction in total treatment duration from 11.2 to 10.4 days (*P* = 0.015)  Non-significant downward pre-intervention-trend (-0.07 days/month, *P* > 0.05)  Trend immediately post-intervention: Significant decrease by 1.4 days/month (*p* = 0.04)  Post-intervention trend: Significant increase by 0.27 days/month  (*p* = 0.03)  Trend six months post-intervention: Non-significant upward trend of +0.57 days, *P* > 0.05 |
| Krassioukov [85]  2016  Canada  Autonomic dysreflexia  management | Cohort, pre- post  108 health care providers (mainly physicians and nurses)  working at emergency departments of trauma centres  across Canada | Context: Quality improvement  To assess the efficacy of an  educational seminar in improving short- and long-term recognition, diagnosis and  management knowledge of autonomic dysreflexia  according to available practice guidelines | Theory: NR  Tailored to pre-identified barriers: NR  Co-designed with patients and professionals:  A component of the intervention, the seminar, was co-developed by spinal cord injury clinicians and clinician-scientists with input from patients, specialists in rehabilitation medicine, and other Canadian emergency department colleagues | | Target: Professionals  Intervention: Single  Type: PROFESSIONAL: Present guideline materials at meetings + Educate individuals about guideline intent/benefits; FINANCIAL (Health professional): Grant or allowance to individual (not tied to compliance (gift cards, refreshments, or continuing medical education credits))  Content: Guideline-informed overview of recognition and diagnosis of autonomic dysreflexia - dangers of not recognizing it, signs and symptoms, case studies; latest pharmacologic and non-pharmacologic evidence on prevention and management  Format: Educational seminar  Delivery: In person, PowerPoint  Timing:  Seminar given at various times between April 2010- January 2011  45-minute slide presentation in seminar  Post-test immediately after seminar, follow-up test 3 months post-seminar  Personnel: NR | Positive impact  Autonomic dysreflexia knowledge test scores significantly decreased between the post-seminar assessment and 3-month follow-up; however, 3-month scores remained significantly higher than baseline  Significant improvement in knowledge at pre-seminar to post-seminar (n = 108): 11.85 ± 3.88 vs. 18.95 ± 2.39, *t* = -21.357, Cohen’s *d* = 2.21, *P* < 0.001  Significant decline in knowledge post-seminar (n = 108) to 3-month follow-up (n = 23): 18.95 ± 2.39 17.04 ± 3.28, *t* = 3.264, Cohen’s *d* = -0.70, *P* = 0.004  Nonetheless, still a significant improvement from baseline (pre-seminar) knowledge scores to 3-month follow-up scores: 11.85 ± 3.88 vs. 17.04 ± 3.28,  *t* = -6.022, Cohen’s *d* = 1.38,  *P* < 0.001 |
| Lu [86]  2016  USA  Care for incidental pulmonary nodules | Retrospective chart review of cohort, pre- post data  Post- intervention group: 141 patients; the intervention tool had been used in the treatment of 57 patients, and not used in 84 patients  Pre-intervention group: 268 patients | Context: Quality improvement  To retrospectively evaluate the impact of a point-of-care clinical decision support tool on radiologist adherence to department guidelines for follow-up on pulmonary nodules detected incidentally on abdominal CTs | Theory: NR  Tailored to pre-identified barriers: NR  Co-designed by patients or professionals: NR  International guidelines were refined and aggregated by department’s thoracic and abdominal radiologists due to the number and unwieldiness of original guidelines | | Target: Professionals  Intervention: Single  Type: STRUCTURAL CHANGES: Information/communication technology + Quality improvement, performance measurement system   Content:  (i) Clinical decision support tool inserts recommendations directly into radiologist report based on size of largest incidental lung nodule; inserted text is highlighted and can be edited by radiologist; tool is also integrated with department’s dictation system  (ii) Educational sessions introducing tool and encouraging its use  Format: Clinical decision support tool, educational sessions  Delivery: Electronic health record, in person  Timing: Consecutive patients with first abdominal CT between 1 January 2012-4 April 2013 (15 months)  Pre-decision-support tool:  1 January 2012 - 21 October  2012 (9.5 months)  Post-decision-support tool: 22 October 2012- 4 April 2013 (6.5 months)  Two educational sessions of 30 minutes each  Personnel: NR  Control: Pre-intervention data | Positive impact  Significant improvement in proportion of guideline concordant radiologist recommendations post-intervention compared to pre-intervention: 65% (92/141) vs. 50% (133/268), *P* = 0.003  Radiologists made a significantly greater proportion of guideline-concordant recommendations when the decision support tool was available and was used (95% (54/57)) compared to when it was available and not used (45% (38/84), *P* < 0.001), and compared to the pre-intervention control group (50% (133/268), *P* < 0.001)  There was no significant difference between the proportion of guideline-concordant radiologist recommendations post-intervention when the decision support tool was not used (45% (38/84))and pre-intervention proportions of the same in the control group (50% (133/286), *P* = 0.48) |
| Mader [87]  2016  USA  Breast, cervical, and colorectal cancer screening | Cohort, pre- post  23 primary care practices including physicians, nurse practitioners, physicians, nursing staff and administrative staff  210 clinical providers attended the initial academic detailing session at their practice | Context: Quality improvement  To evaluate the efficacy and  feasibility of an intervention combining practice facilitation and academic detailing in improving breast, cervical, and colorectal cancer screening in primary care practices | Theory: TRANSLATE (set your Target, use Registry and Reminder systems, get  Administrative buy-in, Network information systems, Site coordination, Local Physician Champion,  Audit and feedback, Team approach, and Education)  Tailored to pre-identified barriers: NR  Barriers to cancer screening were identified through focus groups and key informant interviews after the intervention  Co-designed with professionals (through meetings with practice facilitator): Professionals (e.g., medical directors, practice managers) at each  practice selected specific interventions based on their practice’s priorities and constraints (e.g., practice size, location, administrative structure, performance priorities); selected interventions were reviewed to ensure that they were evidence-based | | Target: Professionals  Intervention: Multifaceted  Type: PROFESSIONAL: Present guideline material at meetings + Educate groups about guideline intent/benefits + Provide feedback on compliance; PATIENT: Education; FINANCIAL (Health professional): Grant or allowance to individual (continuing medical education credit); STRUCTURAL CHANGES: Information/communication technology + Method of service delivery  Content:  Academic detailing: Evidence-based strategies to increase breast cancer, cervical cancer, and colorectal screening; Practice facilitation: Review of workflow and policies, and clinic performance in cancer screening; feedback on provider-level and practice-level performance; addressing documentation procedures; streamlining reminder systems and other workflows; institution of patient education and outreach  Barriers identified through focus groups AFTER intervention  Format: Academic detailing sessions, practice facilitation  Delivery: In person  Timing:  1-hour academic detailing session  6 months of practice facilitation following the initial academic detailing session, from 2014 to 2015 totalling 889 hours cumulatively  Personnel: Primary care physician with cancer prevention expertise, practice facilitators with formal training in quality improvement coaching | Positive impact  Mean screening rates were significantly higher for breast cancer (36.96% vs. 49.96%;  *P* < 0.001) and colorectal cancer (32.74% vs. 38.30%; *P <* 0.001) after the intervention, though not for cervical cancer  However, variation was wide in screening rates among practices, with standard deviations ranging from 16-21 percentage points  Practices showed significant improvement in TRANSLATE-model elements overall (mean score 28.20 (SD 3.56) vs. 26.28 (SD 3.68), *P* < 0.001), and in 5 of 9 TRANSLATE-model elements (mean (standard deviation), *P*-value):   - Practice-facilitator-rated achievement of target: 2.70 (0.95) vs. 3.00 (1.02),   *P* = 0.031   - Registry and reminder systems: 3.22 (0.72) vs. 3.39 (0.48), *P* = 0.001 - Administrative buy-in: 3.15 (0.75) vs. 3.43 (0.59),   *P* = 0.029   - Network information systems: 3.22 (0.86) vs. 3.48 (0.70),   *P* = 0.007   - Audit and feedback: 2.63 (0.96) vs. 2.85 (0.85),   *P* = 0.022   - Practice-facilitator-rated education: 2.70 (0.67) vs. 2.91 (0.63), *P* = 0.057 |
| Paulus [88]  2016  Netherlands  Manual hyperinflation in intubated mechanically ventilated patients | Controlled pre- post  A random sample of 80 ICU nurses | Context: New guideline  To investigate the effect of guideline-based educational meetings and individual feedback on manual hyperinflation performance | Theory: NR  Tailored to pre-identified barriers: NR  Co-designed with patients or professionals: NR | | Target: Professionals  Intervention: Multifaceted  Type: PROFESSIONAL: Educate groups about guideline intent/benefits + Provide feedback on compliance  Content: Information about the guideline  Format: Educational modules + one-on-one instruction in the skills  laboratory  Delivery: In-person  Timing: 30-min educational module  Personnel: Investigator nurses | Mixed results  Manual hyperinflation performance improved: the peak inspiratory flow to peak expiratory flow ratio decreased from 1.4 (95% CI 1.1, 1.7) to 0.8 (95% CI 0.6, 1.1; *P* < 0.01)  Although peak inspiratory flow decreased from pre-intervention to post-intervention, the difference was small and not statistically significant (flow in L/min NR, *P* = NR)  Peak expiratory flow significantly increased (L/min NR, *P* = NR), in keeping with guidelines |
| Riis [89]  2016  Denmark  Low back pain | Cluster RCT  54 general practices stratified by size with 1101 patients  Intervention group: 28 practices with 539 patients  Control group: 26 practices with 562 patients | Context: New guideline  To assess whether a multifaceted implementation strategy would be more effective in reducing secondary care referral of low back pain patients within 12 weeks compared to a passive implementation strategy | Theory: Biopsychosocial model of illness  Tailored to pre-identified barriers: The study protocol (Riis et al., 2013) mentions deploying intervention components that address pre-identified barriers (how they were identified NR), and testing these components in 3 practices; the components were subsequently rejected or adjusted for use in this study  Co-designed by patients or professionals: NR | | Target: Professionals  Intervention: Multifaceted  Type: PROFESSIONAL: Present guideline material at meetings + Educate groups about guideline intent/benefits + Provide reminders to individuals/groups about intent/benefits + Provide feedback on compliance + Print material (posters, mousepads); FINANCIAL: Grant or allowance to individual (not tied to compliance); STRUCTURAL CHANGES: Information/communication technology + Quality improvement, performance measurement system  Content:  Passive implementation: Invitation to information meeting about new guideline; Regional low back pain website; Guideline-related information and further learning opportunities; Referral opportunities for patients with psychosocial problems; Guideline-concordant best practice pop-up alerts in electronic medical record; Financial incentives to participate +  Multifaceted implementation: Specialized outreach education on low back pain guidelines comprising lecture and role-playing components; Provision for follow-up contact with outreach educator; Feedback reports (frequency of referrals + new appointments); Information folder with low back pain guidelines; Posters and mouse pads containing reminders about guideline; Risk stratification tool integrated into electronic medical record and embedded in best practice pop-up alert, Additional psychosocial questions incorporated into pop-up alert  Format: Information meetings, continuing medical education, educational outreach visits, feedback reports, electronic pop-up, electronic risk stratification tool, information folders, website  Delivery: In person, electronic, online, written feedback, printed material  Timing: January 2013 to July 2014  Patient follow-up at 4, 8, and 52 weeks  Initial 1- to 2-hour outreach visit  After 4 weeks of patient inclusion, one 0.5-hour visit  Personnel: Outreach visitors who were primary care physiotherapists  Control: Passive implementation strategy noted above only | Mixed  Fewer patients in the intervention group were referred to secondary care for low back pain within 12 weeks compared to the control group: 27/539 patients (5.0%) vs. 59/562 patients (10.5%, adjusted OR 0.52 [95% CI 0.30-0.90]; *p* = 0.020)  Sensitivity analysis showed similarly lower estimates of patients in intervention group referred to secondary care at 8, 16, and 52 weeks compared to control group  No significant differences in employment status, sick leave within 14 days, back pain intensity, or disability measures between groups at 4, 8, and 52 weeks  However, the intervention also resulted in less satisfied patients after 52 weeks (adjusted OR 0.50 [95% CI 0.32 to 0.81], *p* = 0.004) |
| Sacco [90]  2016  USA  Pain management and sedation in trauma intensive care | Pre- post  400 charts of patients admitted to a trauma intensive care unit were reviewed | Context: New guideline  To determine whether the new guideline would decrease the use of continuous drips in the ICU while achieving patient comfort and the prevention of negative patient outcomes while maintaining or decreasing nursing and provider workload | Theory: NR  Barriers pre-identified in the literature and confirmed by observation in the intensive care unit were:   - Fear of ventilator compromise - Fear of self-extubation - Clinical instability - Increased nursing workload   (Hughes et al., 2013; Mehta et al., 2012; Rose et al., 2015)  Tailored to pre-identified barriers  Co-designed with professionals: The guideline on which this study is based was developed by nurses, and the interprofessional team, including unit champions, developed the educational component of this intervention | | Target: Professionals  Intervention: Multifaceted  Type: PROFESSIONAL: Educate groups about guideline intent/benefit + Recruit an opinion leader who recommends implementation; ORGANIZATIONAL (Health professional): Create an implementation/multidisciplinary team  Content: Information about the guideline  Format: Educational modules + an interprofessional implementation team, including RN champions, facilitated practice change.  Delivery: In-person and/or online  Timing: Study took place over a 9-month period in 2010-2011  Development of mandatory staff education within 2 months of guideline development  Staff education implemented in 1 month  Post-implementation: 6 months  Personnel: Unit champions, seven RNs, nurse practitioners and physician assistants  Control: NR | Positive change  A reduction in ventilator days (-17.81%, *P* > 0.05), ICU length of stay (-4.16%, *P* = NR), average sedation days (number of days NR, p = 0.007), and increases in the use of antipsychotics for delirium management (34.5% post-intervention vs. 10.5% pre-intervention, *p* = 0.01) and improvements in nursing satisfaction. |
| Thomas [91]  2016  Australia  Fall risk in older adults in acute care | Pre- post  Acute care physical therapists and physical  therapist students (n = NR)  Pre-intervention audit: 159 case notes  Post-intervention audit: 154 case notes | Context: Quality improvement  To assess an intervention to improve physical therapists’ adherence  to key guideline recommendations for managing risk of falls following hospital discharge | Theory: Theoretical Domains Framework, Behaviour Change Wheel, French et al. (2012) 4-step process  The study protocol (Thomas and Mackintosh, 2014) states that barriers were identified through a 4-step process described by French et al., 2012, which included in-person interviews, meetings with stakeholders, reviews of hospital data, case note audits, and in-person and phone interviews with patients  Tailored to pre-identified barriers:  Key practices that fell short of standards were identified:  (1) Patients at risk for falls upon discharge were not identified  (2) Irregular documentation of clinical information on handover of such at-risk patients  (3) Patients not involved in self-identifying for fall risk  (Thomas and Mackintosh, 2014)    The Theoretical Domains Framework was applied to the above identified problem areas to yield specific barriers and enablers, which were used to inform interventions  Co-designed with professionals: A committee consisting of members of the physical therapy department provided feedback on barriers and enablers, identified and tested implementation strategies, and adapted education materials (Thomas and Mackintosh, 2014) | | Target: Professionals and patients  Intervention: Multifaceted  Type: PROFESSIONAL: Distribute guideline material + Educate individuals about guideline intent/benefits + Provide feedback on compliance + Print material (booklet) + Enable self-audit + Tailor guideline; PATIENT/CONSUMER: Print material (booklet); ORGANIZATIONAL (Health professional): Additional human resources + Create an implementation/multidisciplinary team + (Patient) Consumer feedback, suggestions, complaints; STRUCTURAL CHANGES: Information/communication technology + Quality improvement, performance measurement system + Method of service delivery  Content: Review and feedback on proposed interventions; Information about guideline recommendations  and the consequences of failing to meet them; Development of clinical pathway for guideline-recommended identification and management of patients at risk for falls; Modification of existing hospital assessment to include prompts containing fall-risk querying, identified risks, and action plans; Development of processes for high-quality clinical handover upon discharge; Distributing a fall risk booklet; Establishing a fall risk committee; Allocating staff time for audit of fall-risk processes; Audit and feedback process compiling and distributing performance data to relevant parties  Format: Medical record audits, telephone interviews, focus groups, establishment of committees, education sessions, clinical pathway, modified/new protocols, updated clinical handover/discharge papers, informational booklet  Delivery: In person, hospital electronic system, written materials  Timing: Pre-implementation data captured up to 2 years prior to the intervention; post-implementation data  captured 6 weeks post- and 12 months post-intervention  Personnel: NR | Positive impact  The intervention resulted in significant improvements in physical therapy practice concordance with guideline recommendations, including:   - Increased proportion of patients who were identified to be at risk of falls prior to discharge (94.8% vs. 6.3%, chi-square=241.65, *P* < 0.001) - Increased documentation of clinical handover at discharge (90.9% vs. 68.6%, chi-square=22.68, *P*< 0.001) - Improvement in the quality of documentation for clinical handover (34.9% vs. 92.9%, chi-square=117.04,   *P* < 0.001), among other significant measures  Proportion of patients reachable by telephone who were able to recall a discussion about fall risk was 15/47 (32%) pre-intervention vs. 23/36 (64%) post-intervention (chi-square=7.16, *P* = 0.007) |
| Vellinga [92]  2016  Ireland  Antimicrobial prescribing for urinary tract infections | Cluster RCT  30 practices with 71 GPs with 3341 consultations for urinary tract infection  Intervention arm A: 10 practices with 1124 consultations coded urinary tract infection  Intervention arm B: 10 practices with 1047 consultations coded urinary tract infection    Control arm: 10 practices with 1143 consultations coded urinary tract infection | Context: Quality improvement  To assess the effect of evidence-based prescribing workshops on antimicrobial  prescribing, particularly first-line antimicrobials, for urinary tract infection | Theory: NR  Tailored to pre-identified barriers: NR  Co-designed by patients or professionals: NR | | Target: Patients and professionals  Intervention: Multifaceted  Type: PROFESSIONAL: Distribute guideline material + Educate groups about guideline intent/benefits + Provide reminders to individuals/groups about intent/benefits + Provide feedback on compliance; PATIENT/CONSUMER – Education; STRUCTURAL CHANGES: Information/communication technology + Quality improvement, performance measurement system  Content:  Baseline coding workshop (all arms): Importance of consultation coding for audit report;  Phase 2 workshop + reminder (arms A and B): Review of national guidelines for antimicrobial prescribing and underlying evidence; audit report of practices from 3-month baseline period; pop-up reminder outlining guidelines when consultation coded as urinary tract infection;  Phase 2 workshop (arm B only): Additional evidence in favour of delaying antimicrobial prescriptions for suspected urinary tract information  Phase 3 (arms A and B): Multimedia informational application/infomercial  Format: Workshops, audit and feedback, multimedia educational application/infomercial  Delivery: In-person, electronic, email  Timing: 3-month baseline period followed by 6-month intervention period. 5-month passive data collection period  Personnel: NR  Control: 10 practices received the baseline workshop only | Positive impact  The proportion of guideline-concordant antimicrobial prescribing for urinary tract infections increased in intervention arms relative to control. Improvements in guideline-based prescribing were sustained at 5 months after the intervention, however no *p* values reported  First-line antimicrobial prescribing pre- vs. post-intervention improved within intervention arms A (22.8% absolute increase) and B (16.7% absolute increase) and worsened in the control arm  (-1.7%), *p* = NR  Adjusted ORs of 2.7 (95% CI 1.8-4.1) for intervention arm A and 2.0 (95% CI 1.3-3.0) for intervention arm B to calculate the effect of the intervention meant a person visiting an intervention practice was 2.3 times more likely to have been prescribed a first-line antimicrobial compared to the control arm  The multimedia application was calculated to have not had a significant effect on any outcome (statistics NR) |
| Barnes [93]  2015  USA  Obesity guideline in primary care centres | Quality improvement project  100 health records of adult patients aged 18–64 who attended visits at the academic health centre were examined. | Context: Quality improvement  To increase adherence to obesity guideline (documentation of BMI, appropriate diagnosis of obesity and documentation of weight loss plan) | Theory: Theory of  Planned Behaviour  Patient barriers identified through the literature were:   - Provider blame - Lack of desire for assistance with weight loss from provider   (Ruelaz et al., 2007)   - Being unaware of the chronic nature of obesity - Socioeconomic status - Lack of time - Lack of social support - Comorbidities - Medications - Substance abuse   (Mauro et al., 2008)  Provider barriers identified through the literature were:   - Lack of confidence in managing obesity - Concerns about treatment efficacy - Frustration with prior attempts at treating obesity - Negative attitudes towards patients with obesity   (Jay et al., 2009)   - Perception that patients lack the time for exercise - Perception that patients lack the self-control to avoid unhealthy eating   (Ruelaz et al., 2007)   - Providers not utilizing objective screening tools to treat obesity contributes to decreased management, and worse in patients with overweight or obese status   (Bardia et al., 2007; Ma et al., 2009)  Tailored to pre-identified barriers: The education sessions were designed to target previously identified barriers to obesity management.  Co-designed with patients or professionals: NR | | Target: Patients and professionals  Intervention: Multifaceted  Type: PROFESSIONAL: Educate groups about guideline intent/benefits + Provide reminders to individuals/groups about intent/benefits + Provide feedback on compliance + PATIENT/CONSUMER: Print material  Content: Guideline-based information  Format: Educational modules, reminder system, feedback and printed materials  Delivery: in-person and online  Timing: Weekly reminders and halfway the project, audit and feedback.  Personnel: NR | Mixed results  Primary care providers did not significantly improve in their documentation of diagnosis and planned management of obesity for patients with BMI greater than or equal to 30 (2 documentations pre-intervention to 4 post-intervention, *P* > 0.05  Medical assistants recorded height, weight and BMI in the patient record by 13% more often, which was significant (*P* < 0.01) |
| de Beurs [94]  2015  Netherlands  Proper patient care in suicide assessment and prevention | Cluster RCT  303 mental health practitioners (nurses, psychologists, and psychiatrists) from 45 psychiatric departments across the Netherlands  Intervention group: 199 mental health practitioners from 18 psychiatric departments  Control: 104 mental health practitioners from 16 psychiatric departments | Context: Quality improvement  To investigate the effect of an e-learning supported Train-the-Trainer program on adherence to national suicide assessment and treatment guidelines | Theory: Adult Learning Theory, Diffusion of Innovation Theory  Tailored to pre-identified barriers: NR  Co-designed with patients or professionals: NR | | Target: Professionals  Intervention: Multifaceted  Type: PROFESSIONAL: Present guideline materials at meetings + Educate individuals about guideline intent/benefits + Tailor guideline; FINANCIAL (Health professional): Incentive (individual financial reward or benefit for compliance)  Content: modelling the implementation of a guideline for the assessment and treatment of suicidal behaviour using Chronological Assessment of Suicidal Events interview, which resulted in the grading of risk and protection factors in suicide based on outcome, which in turn should result in a structured diagnosis, treatment plan, and safety plan for the patients  Format: 1 e-learning module, in-person roleplay exercise using CASE interview as framework  Delivery: In-person and online  Timing: 1-day-long roleplay exercise (presumed 8 hours), 1-hour e-learning module for a total of 9 hours, follow-up after 3 months  Personnel: Masters (mental healthcare experts affiliated with study), Trainers (nurses, psychologists or psychiatrists affiliated with their department), and graduate psychology students  Control: Implementation as usual, i.e., the dissemination of guidelines via websites, journal articles, conference presentations, books, and manuals | Positive impact  Significant difference in primary outcome: guideline adherence, in intervention group vs. control group, respectively:  (70.5 vs. 66.0, *B* = 4.6 [95% CI1.6, 7.5], *p* = 0.02, Cohen’s *d* = 0.4)  Significant difference in secondary outcome: provider’s confidence in abilities, in intervention group vs. control group, respectively: (7.7 vs. 6.9, *B* = 0.8 [95% CI 0.4, 1.2], *p* < 0.001, Cohen’s *d* = 0.7)  No significant difference in recognition of appropriate responses to suicidal behaviour (55 in intervention group vs. 53 in control group, *B* = 1.6 [95% CI-1.7, 4.9], *p* > 0.05)  Significant improvement in guideline adherence (*p* < 0.001) among intervention group nurses (*B* = 6.6 [95% CI 3.2, 10.0], *p* = 0.009, Cohen’s *d* = 0.6), but not among intervention group psychologists or psychiatrists (*B* = -1.2 [95% CI -6.1, 3.7], *p* > 0.05), indicating that the intervention is most effective when delivered to nurses  Self-assessment of knowledge about suicidal behaviour is reported in the study, but will not be included in this table because it is an exclusion criterion of our scoping review |
| Bouaud [95]  2015  France  Breast cancer treatment | Retrospective chart review  Chart review data based on 394 physician decisions, with an optimal decision for each physician decision used as a control | Context: Quality improvement  To determine the effect of an electronic clinical decision support system on adherence to breast clinical cancer guidelines and/or adherence to clinical decision support system recommendations | Theory: Concept of “psychological reactance” from cognitive engineering (Vashitz et al., 2009), concept of “automation bias” (Goddard et al., 2014)  Tailored to pre-identified barriers: NR  Co-designed with patients or professionals: NR | | Target: Professionals  Intervention: Single  Type: STRUCTURAL CHANGES:  Information/communication technology + Quality improvement, performance measuring system  Content: A sequence of closed-ended questions that lead to system-generated guideline-based advice for a recommended care plan for breast cancer  Format: Electronic clinical decision-support system; IT interface either partially integrated or fully integrated with physician and electronic health records  Delivery: Electronic health record  Timing:  10 months (June 2009 to April 2010)    Personnel:  Clinical research assistant    Control:  Decisions made by clinical research assistant using OncoDoc2 (clinical decision-support system) | Positive results  Overall CPG compliance rate:  86.8%  Overall compliance with clinical decision support system: 75.4%  Reactance is absence of compliance.  Overall reactance rate (24.6%):  Sum of rates of:   - compliance to guidelines but noncompliance to decision support system - noncompliance to guidelines and noncompliance to decision support system   68.8% of decisions were made when guideline matched recommendation from decision support system  Guideline compliance rate  (χ^2^, p < 10^-9^):   - 92.3% when recommendations from system matched guidelines - 65.5% when no system recommendations matched guidelines - 94.9% when at least one system recommendation matched the guideline   Decision support system compliance rate (χ^2^, p < 10^-37^):   - 92.3% when recommendations from system matched guideline - 21.4% when no system recommendation matched any guideline - 74.4% when at least one system recommendation matched guidelines   Decision-making distribution:   - 70.8% of cases were compliant with system recommendations and guidelines - 16.0% of cases were compliant with guidelines but not with system recommendations - 4.6% of cases were compliant with system recommendations but not with guidelines - 8.6% of cases were compliant with neither system recommendations nor guidelines |
| Breimaier [96]  2015  Austria  Fall prevention in acute care hospital settings | Pre- post  110 nurses in two departments at a hospital | Context: Quality improvement  To assess the effectiveness and required time investment of multifaceted, tailored strategies for implementing fall-prevention guidelines in nursing | Theory: **Participatory action research approach** (Greenhalgh et al., 2004; Waterman et al., 2001; Carr and Kemmis, 1990; Smith, 2008; Dick, 2000; Kemmis, 2006); **Consolidated Framework for Implementation Research** (Damschroder et al., 2009; Ilott et al., 2013); **implementation interventions/strategies framework provided by Cochrane Effective Practice and Organisation of Care Review Group** (EPOC, 2002; Mäkelä and Thorsen, 1999)  Six implementation strategies were tailored to department-level barriers (nature of barriers NR) as pre-identified by participant nurses during steering committee meetings  Co-designed during steering committee meetings with nurse representatives and other department nurses, who discussed falls prevention, aims for guideline implementation, gaps in preventative measures, and who selecting and tailoring implementation strategies to each department’s needs | | Target: Professionals  Intervention: Multifaceted  Type: PROFESSIONAL: Distribute guideline material + Present guideline material at meetings + Educate groups about guideline intent/benefits + Recruit an opinion leader who recommends implementation + Achieve consensus that guideline should be implemented + Provide alerts when practice deviates + Provide feedback on compliance + Provide feedback from healthcare professionals + Print material (summary, algorithm, referral forms, etc.) + Enable self-audit; STRUCTURAL CHANGES: Physical structure, facilities or equipment + Information/communication technology  Content: Fall-prevention guideline, discussions on fall prevention, how to implement intervention  Format: educational meetings, steering group meetings, guideline material, steering group member leadership, audit and feedback protocols, altering documentation conventions (“adaptation of nursing record systems”), modifying facilities and changing equipment  Delivery: In-person, written feedback, hard-copy or intranet-accessible guideline material  Timing: Average 1-hour-long educational meetings at baseline, six to seven 1.5-hour-long meetings from January 2011-July 2011, weekly/regular audit and feedback for each of a nurse’s patients from January 2011 to September-November 2011  Personnel:  Selection of department – Nurse director of hospital  Local opinion leaders – 5 to 8 representatives  Audit and feedback – ward managers, specially-appointed nurse, head nurse | Positive changes on two of three objectives  Significant improvement in knowledge of how to access fall prevention guidelines (52.4% of n = 105 to 81.8% of n = 110, *p* < 0.001), by whom guidelines had been developed (37.4% of n =99 to 61.5% of n = 104, *p* < 0.001), knowledge of guidelines (65.6% (standard deviation (SD) = 8.221, n = 106) to 69.7% (SD = 9.150, n = 110, *p* = 0.001), attitude toward guidelines (mean = 3.014, SD = 0.0353, n =81 vs. mean = 3.188, SD = 0.0344, n = 101, *p* = 0.001), no data on improved implementation of guidelines.  Greatest participant-identified changes as reported in group interviews were, in order of magnitude:  1) increased awareness of fall prevention  2) greater availability of fall prevention equipment/devices  Study reported participants being satisfied with the approach and the results |
| Elder [97]  2015  USA  Acute stroke care | Retrospective chart review – comparison  120 patient charts from 3 hospitals  Intervention tool (an order set) was used in 70 patients, and not used in the remaining 50 patients | Context: Quality improvement  To determine the impact of physician use of a medication order set on adherence to eight standards of stroke care, and to determine its impact on length of hospitalization and patient discharge disposition | Theory: NR  Tailored to pre-identified barriers: NR  Co-designed by patients or professionals: NR | | Target: Professionals  Intervention: Single  Type: STRUCTURAL CHANGES:  Information/communication technology + Quality improvement/performance measurement system  Content: Medication order set with 8 standards of acute stroke care:  1. Venous thromboembolism prophylaxis  2. Anticoagulants for atrial fibrillation or flutter  3. Thrombolytic therapy  4. Antithrombotics by end of hospital day 2  5. Statins on discharge  6. Antithrombotics on discharge  7. Stroke education  8. Assessed for rehabilitation  Smoking cessation counselling also examined  Format: Order set within electronic health record  Delivery: Electronic health record  Timing: Chart review for hospital A: 1 January 2007 – 31 August 2011, other two hospitals 1 September 2009 – 31 August 2011  Personnel: NR  Control: Comparison group with no order set use (n = 50) | Some positive results  Use of the order set (vs. non-use of order set) increased compliance with quality standards, significantly so with three standards: venous thromboembolism (91.4% vs. 74.0%, *p* = 0.01) prophylaxis, stroke education (81.4% vs. 54.0%, *p* = 0.001), and statin therapy at discharge (91.4% vs. 68.0%, *p* = 0.001)  Other measures with non-significant improvement in the order set cases were antithrombotics on discharge (100.0% vs. 98.0%, *p* = 0.417), assessment for rehabilitation (91.4% vs. 84.0%, *p* = 0.211), and dysphagia screening (84.3% vs. 70.0%, *p* = 0.061)  Anticoagulants for atrial fibrillation or flutter (98.6% vs. 98.0%, *p* = 1), thrombolytic therapy (98.6% vs. 100.0%, *p* =1), antithrombotics by end of hospital day 2 (91.4% vs. 92.0%, *p* = 1), and smoking cessation counselling showed no change (98.6% vs. 98.0%, *p* = 1) |
| Erickson [98]  2015  USA  Obesity guidelines in primarily rural interprofessional settings | Retrospective pre- post  Four public health departments who selected 10 sites from:  A) 2 health care systems,  B) 1 migrant health service,  C) 1 federally qualified health care centre, all  comprising 39 participants:   - 1 supervising administrator per site   (n = 10)   - 2-3 of each from each site: public health nurses, registered nurses, nurse practitioners, physicians, dietician, physical therapist, occupational therapist, physical therapy assistant, IT specialists, billing and coding specialists   (n = 29) | Context:  Quality improvement  To evaluate whether a collaborative system-level intervention had an impact on the extent of obesity guideline translation across different organizations, and to assess whether the Omaha method was effective as mode of translating guidelines and measuring outcomes | Theory: Omaha System Care Plan, 5As system for barriers assessment (Ask, Advise, Assess, Assist, Arrange), motivational interviewing  Barriers were assessed over the course of the intervention using the 5As framework  Co-designed with patients or professionals: NR | | Target: Professionals  Intervention: Multifaceted  Type: PROFESSIONAL: Recruit an opinion leader who recommends implementation + Distribute guideline material + Provide feedback on compliance + Print material; PATIENT/CONSUMER:  Education (group); FINANCIAL  Grant or allowance to group/institution (not tied to compliance); ORGANIZATIONAL (Health professional): Reallocated or new role + Create an implementation/multidisciplinary team + Communication between distant health professionals; STRUCTURAL CHANGES: Organizational structure + Physical structure, facilities or equipment + Quality improvement/performance measurement systems + Integration of services  Content:  Successful translations of system-wide adaptations of obesity guidelines  Nurses role in implementing teaching:  🡪 Developing a plan with quantifiable goals, obesity guideline, “organizational readiness to change," strategies for quality improvement “motivational interviewing”  Employment of scales and stadiometers, distribution of obesity-related information, implementation of alerts and reminders in medical records  Addition of “employee wellness” and “worksite wellness” programs  Electronic tracking systems,  Relay progress of 5As (Ask, Advise, Assess, Assist, Arrange) to public health nurse,  Public health nurse uses 5As to help sites as well as identify barriers, assess progress and provide feedback  Format: face-to-face and web-based interactive training, electronic tools  Delivery:  In person, online, electronic health record  Timing:  First 12 months – interactive training  Quarterly for 2 years – PHN feedback  Personnel:  Public health nurse, ICSI consultants, research team | Positive impact  Omaha System Problem Rating Scale for Outcomes  was used to measure progress  Nurse-reported behaviour aspect of Knowledge-Behaviour-Status measure improved from 1.10 (not appropriate) at baseline to 3.00 at follow-up (on a scale of 1-5)  Knowledge aspect (from perspective of public health nurse) of Knowledge-Behaviour-Status measure improved from 1.20 (not appropriate) to 3.90 at follow-up (on a scale of 1-5)  Participants in the intervention were also given the opportunity to rate themselves on Knowledge-Behaviour-Status dimensions, with substantial agreement between the nurse’s assessment and the organization’s self-assessment of Knowledge, and moderate agreement in Behaviour and Status    Comments made by clinicians matched Omaha scores  Omaha tool was seen as a promising method in delivering system-wide implementation of guidelines |
| Gervera [99]  2015  USA  Diabetes outpatient care | Cohort, pre- post  Five registered nurses overseeing 20 veterans with diabetes | Context: Quality improvement  To determine the effect of the integration of a diabetes assessment tool into the electronic health record on:  (i) evidence-based care  (ii) standardization of care coordination  (iii) promotion of self-management and proper resource use among patients | Theory: Plan-Do-Study Act (PDSA) cycle  Tailored to pre-identified barriers: NR  No co-design, as the study’s registered nurses were only solicited for feedback on the tool after the intervention.  The tool was adjusted as per their feedback. | | Target: Professionals  Intervention: Single  Type: STRUCTURAL CHANGES: Information/communication technology + Method of service delivery  Content: Diabetes assessment tool utilizing some biometric data drawn from a home telehealth monitoring program, and embedded within the electronic medical record, consisting of 30 data points in 3 areas:  (1) Diabetes care indicators, e.g., annual exams, labs  (2) Disease management components, e.g., meal planning, exercise  (3) Goal setting  Tool is meant to evaluate the patient’s need for diabetic services such as annual screenings and diabetes education, and to evaluate the patient’s self-management; and for the registered nurse to facilitate the required care on the basis of the above  Format: Electronic-health-record-integrated assessment tool  Delivery: Electronic health record  Timing: Assessment tool given to care providers for use with 4 patients who had diabetes evaluations coming up in the next 180 days  Personnel: Registered nurses  Control: Pre-introduction of tool data; only use of information recorded from home monitoring device and responses to diabetes assessment questions, which the care provider would interpret and judge, and then provide interventions as needed | Positive changes  Use of the tool improved assessment of all annual exams and labs by 88.12%, *P* = NR  Prior to introduction of tool, checking for the completion of annual exams other than HbA1c was not routinely done by care providers  Assessment tool increased disease management assessment and offering of diabetes management services by 16.5%, *P* = NR  Tool increased goal-setting activity by 50%, *P* = NR |
| Giuliani [100]  2015  Australia  Preoperative fasting among burn patients | Retrospective pre- post  39 medical staff and nursing staff in Burns Unit of major urban hospital | Context: Quality improvement  To determine whether intervention improves rate of guideline-concordant fasting instructions that would minimize unnecessarily long preoperative fasting in light of increased caloric requirements of burn patients | Theory: GRiP (Getting Research into Practice)  Intervention was tailored to barriers identified through meetings, informal conversations and email correspondence (anecdote), and focus groups. The barriers were later entered into a GRiP module. Barriers were:   - Poor documentation of preoperative fasting instructions - Lack of knowledge of best practice for fasting - Outdated hospital-wide protocol for preoperative fasting   The intervention was tailored to the pre-identified barriers.  Co-designed with professionals through working groups and meetings:  Project design, development and implementation of intervention, and staff education were accomplished with involvement of Burns Unit staff, senior and ward nursing staff, and other hospital departments | | Target: Professionals  Intervention: Multifaceted  Type: PROFESSIONAL: Present guideline materials at meetings + Educate groups about guideline intent/benefits + Achieve consensus that guideline should be implemented + Provide feedback from healthcare professionals + Tailor guideline; ORGANIZATIONAL (Health professional): Create an implementation/multidisciplinary team + Communication between distant health professionals  Content:  1a) Baseline audit  1b) Addressing of barriers  2. Education on preoperative fasting (audit criterion 3)  3a) Coordination and consensus-establishing between healthcare professionals on audit results, improvement strategies, and fasting guidelines  3b) Development of working party/steering group  4a) Development of feeding protocol (audit criterion 2)  4b) Improvements to documentation (audit criterion 1)  Format:  1a) Retrospective data collection  1b) One-on-one meetings, conversations, email  2. Education sessions  3a) Meeting, Presentation  3b) NR  4a) NR  4b) Stickers with fasting instructions pasted to patient’s door  Delivery: In person, email, written/printed material  Timing:  1a) 2 months  1b) NR  2. 1x/week over 4 weeks  3a) NR, but presumed 1 day in total  3b) NR, ongoing  4a) 5 years in the making  4b) NR  Project took place over 6 months  Personnel:  1a) Principal Investigator  1b) Burns Unit medical staff and senior and ward nursing staff   1. Delivered by Principal Investigator to nurses   3a) Meeting between Principal Investigator, Burns Unit Fellow and Registered Medical Officers; Presentation by Principal Investigator and Senior Dietitian to anesthetists  3b) Anesthetist, surgeons, intensive care specialist, dietitian, nurses  4a) Burns Unit: Director, Dietitians, Fellows; Nurse Practitioner candidate; Allied Health Project Managers; Intensive Care Unit; Gastroenterology and Anesthetics hospital departments  4b) NR | Positive impact  Audit criterion 1—Documented fasting instructions available prior to operation:  Baseline vs. post-implementation: 67% (42/63) vs. 86% (24/28); 19% improvement (*p* = NR)  Audit criterion 2—Documented fasting instructions were in line with protocol:  Baseline vs. post-implementation: 5% (3/63) vs. 57% (16/28); 52% improvement in compliance  (*p* = NR)  Audit criterion 3—Staff exposure to education sessions about reducing preoperative fasting times:  Baseline vs post-implementation: 3% (only 22 staff asked) vs. 54% (21/39 staff), 51% increase  (*p* = NR)  Pre-implementation:  63 data points from 16 patients: Average total fasting time **14 hours** (range 3 to 39.5 hours), preoperative fasting time **9 hours** (range 0 to 16 hours)  Post-implementation:  28 data points from 4 patients: Average fasting time per procedure **10 hours** (range 2.5 to 17 hours), preoperative fasting time **6 hours** (range 0 to 13 hours)  29% reduction in total fasting time (*p* = NR)  33% reduction in preoperative fasting time (*p* = NR) |
| Güder [101]  2015  Germany  Appropriate pharmacotherapy after heart failure | RCT  706 adult patients hospitalized for decompensated heart failure randomized 1:1 to intervention and control groups | Context: Quality improvement  To ascertain the effect of a nurse-coordinated management program on changes in prescription rates and daily dosages of heart failure drugs | Theory: NR  Tailored to pre-identified barriers: NR  Co-designed with patients or professionals: NR | | Target: Patients and professionals  Intervention: Multifaceted  Type: PROFESSIONAL: Provide feedback from healthcare professionals; PATIENT: Education (single) + Print material (booklets on heart failure drugs, material related to self-monitoring)  Content: Recommendations to uptitrate heart failure medications; Training patients to control their own blood pressure, heart rate, heart rhythm and body weight, and to recognize worsening symptoms of heart failure  Format: Written recommendations for prescriptions and uptitrations, In-person coaching of patients  Delivery: Written instructions, in-person  Timing: Telephone calls 1x/week in first month, changing according to patient’s need until 18-month endpoint of study  Personnel: Nurses, cardiologist supervising nurses, patients’ physicians  Control: Usual care | Positive impact  Statistically significant differences between intervention (n = 343) and control groups (n = 363), respectively, in the following measures at 18 months:  Prescriptions rates of:   - ACE inhibitors/ARBs   (95.0% vs. 90.1%, *p* < 0.05)   - beta blockers (93.9% vs. 86.5%, *p* < 0.05)   Age-adjusted mean equivalence doses of:   - ACE inhibitors/ARBs   (68.6 (95% CI 65.1, 72.1) vs. 51.7 (95% CI 48.3, 55.2), *p* < 0.001)   - beta blockers (60.7 (95% CI 57.3, 64.2) vs. 45.3 (95% CI 41.9, 48.6), *p* < 0.001)   Percentage of patients on target dose of:   - ACE inhibitors/ARBs   (49.6% vs. 24.5%, *p* < 0.001)   - beta blockers (39.1% vs. 15.2%, *p* < 0.001)   No significant differences (*p* > 0.05 for all) between intervention and control groups, respectively, at 18 months in prescription rate (50.6% (173/342) vs. 44.9% (162/361)), age-adjusted mean equivalence doses (40.5 (95% CI 35.8, 45.2) vs. 37.5 (95% CI 33.0, 42.1)), and percentage of patients on target doses (36.3% (124/342) vs. 36.0% (130/361)) of mineralocorticoid receptor blockers  Odds ratio calculations indicated that of 15 significant independent predictors of achieving target dose of ACE inhibitors/ARBs at 18 months, randomization arm was the strongest predictor of target dose achievement  Likewise, odds ratio calculations indicated that of 13 independent predictors of achieving target dose of beta blockers at 18 months, randomization arm was the strongest predictor of target dose achievement  Serial quantitative echocardiography indicated significant differences between intervention and control groups at 18 months in:  🡪left ventricular ejection fraction (*p* = NR)  🡪end diastolic size (*p* = NR)  Significant differences between intervention and control groups at 18 months in:  🡪heart failure symptoms (*p* = NR)  🡪Short Form-36 Health Survey scores (*p* = NR)  Above variables were positively correlated (*r* = NR) with ACE inhibitor/ARB and beta blocker equivalence dosages |
| Liddy [102]  2015  Canada  Quality of cardiovascular care | Stepped-wedge cluster RCT  182 primary care physicians from 84 practices | Context: Quality improvement  To ascertain whether a practice-facilitation-focused intervention improved adherence to evidence-based measures of cardiovascular care | Theory: Plan-Do-Study-Act Cycle, elements of the Chronic Care Model  Tailored to pre-identified barriers: NR  Co-designed by patients or professionals: NR | | Target: Professionals  Intervention: Multifaceted  Type: PROFESSIONAL: Distribute guideline material + Present guideline materials at meetings + Educate groups about guideline intent/benefits +  Provide feedback on compliance + Print material (patient self-management material, newsletters); STRUCTURAL CHANGES: Information/communication technology + Method of service delivery  Content:  1. Practice outreach facilitation  a) Audit and feedback consisting of retrospective chart review, presentation/evaluation of results, comparison with other practices in project  b) Consensus-building by identifying areas for improvement, ways of incorporating chronic care model approach into such areas; goal-setting, planning using Plan-Do-Study-Act; support in driving changes  c) Interactive collaborative meetings addressing challenges in encouraging self-management in patients, finding and linking patients to appropriate community resources,  diabetes care, knowledge translation on topics such as diabetes management, smoking cessation, hypertension, and resources for self-management  2. Chronic care model  a) Decision support through provision of the guideline proper  b) Provision of community resources attached to guideline  c) Support for patient self-management comprising tailored plans, which could include goal-setting, action planning  d) Redesign of delivery system on a tailored basis  Format:  1a) Audit results presented, evaluated, and compared during PowerPoint presentation  1b) Regular meetings/communication over course of study  1c) Collaborative meetings consisting of brainstorming sessions, breakout groups, supplemented by knowledge translation newsletters  2a) In-person overview of guideline, paper copy of guideline, directions to appropriate website containing guideline  2b) List of community programs with area-specific referral information; online version of list updated annually  2c) Pocket cards, Questionnaires, Flow charts, Action plan forms, all available online  2d) Examples include use of registries, recall systems, group visits for those with conditions in common, through Excel documents or electronic medical record, depending on practice  Delivery: In-person, phone, email, online, printed material  Timing (per practice):  24 months in total  Year 1/intensive phase: Outreach Facilitator makes 13-18 visits (original plan)  Year 1/intensive phase (actual): Average 6.6 visits (range 2-11)  Year 2/sustainability phase: Outreach Facilitator visits every 6-12 weeks (original plan)  Year 2/sustainability phase (actual): Average 2.5 visits (range 0-10)  1a) 30- to 60-minute PowerPoint presentation  1b) Quarterly to monthly regular meetings (duration NR)  1c) Several (number NR) half-day-long collaborative meetings  2a) Overview of guideline given at start of intervention  2b) NR  2c) NR  Personnel: Four Outreach Facilitators (professionals with master’s degrees, clinical and management experience)  1b and c) Participating primary care providers and office staff  Control: Later-stage wedges served as controls for preceding wedges  (much of the above information is from Additional File 1 of this paper) | Negative results  After adjusting for patient/provider characteristics, least square mean differences showed an absolute decrease of 1.9 % (95%CI: −2.9 to −0.9 %, *P* < 0.0003) in mean adherence from baseline to intensive phase, and then a decrease of 4.2 % in sustainability phase (95%CI: −5.7 to −2.6 %, *P* < 0.0001)  Authors note that none of the practices involved attained level of Outreach Facilitator visits originally intended, note “complexities…of scaling up research studies into sustainable programs” (Liddy et al., 2015) |
| Peiris [103]  2015  Australia  Cardiovascular disease risk measurement and medication management for high-risk patients | Parallel arm cluster RCT  60 primary healthcare centres serving 38 725 active health service users (Aboriginal Australians and Torres Strait Islanders ≥35 years old and others ≥45 years old)  Intervention group: 30 primary healthcare centres with 19 385 active health service users  Control: 30 primary healthcare centres with 19 340 active health service users | Context: Quality improvement  To test whether a multifaceted intervention improved guideline-recommended measurement of cardiovascular disease risk factors, and appropriate medication of those at high risk for, and those with, cardiovascular disease | Theory: NR  Barriers associated with the use of decision support systems in general were pre-identified through semi-structured interviews (from study protocol, Peiris et al., 2012). Nature of barriers NR.  Tailored to pre-identified barriers: NR  Co-designed with patients or professionals: NR | | Target: Professionals  Intervention: Multifaceted  Type: PROFESSIONAL: Provide alerts when practice deviates + Provide feedback on compliance + Enable self-audit  FINANCIAL (Health professional): Grant or allowance to group/institution (not tied to compliance)  STRUCTURAL CHANGES: Informational/communication technology + Quality improvement, performance measurement system + Integration of services  Content:  1. New guideline-concordant electronic data support system consisting of:  a) Assistance with point-of-care risk assessment  b) Patient-clinician risk communication tool  c) Data extraction tool to enable audit within clinic  d) Access to online portal for comparison of six performance indicators against other clinics  Format: Electronic decision support system, online quality improvement portal, support for implementation of electronic decision support system:   1. On-Site Support: in-person 2. Remote Clinical Support: webinar, remote desktop connection, phone 3. Technical Support: Remote desktop connection, phone   Delivery: Electronic health record, online, in-person, phone  Timing: Median support times:  On-Site Support: 300 minutes  Remote Clinical Support: 38 minutes for 14 of 30 sites  Technical Support: 320 minutes for 28 of 30 sites  Total median time investment of 658 minutes or 10.96 hours over  12 months  Personnel: Project staff (qualifications NR)  Control: Care for cardiovascular disease as usual | Positive changes, with non-significant results slightly in favour of intervention  Cardiovascular disease risk measurement screening:  1. Primary outcome – Significantly larger proportion of intervention group receiving proper and timely cardiovascular disease risk measurement (62.8% vs. 53.4%, risk ratio (RR) = 1.25 (95% CI 1.04, 1.50), *p* = 0.02)  2. Secondary outcomes – no significant differences between proportions of patients in intervention group and control group, respectively, in:  i) Recorded smoking status  (90.8% vs. 89.1%, RR = 1.04 (95% CI 0.96, 1.13), *p* = 0.35)  ii) Systolic blood pressure recorded during study (84.8% vs. 80.6%, RR = 1.08 (95% CI 0.99, 1.18), *p* = 0.09)  iii) Body mass index measured during study (50.5% vs. 49.4%, RR = 0.97 (95% CI 0.77, 1.23), *p* = 0.79)  iv) Urinary albumin-to-creatinine ratio measured in previous 24 months (26.8% vs. 22.1%, RR = 1.23 (95% CI 0.84, 1.80), *p* = 0.29)  v) estimated glomerular filtration rate measured in previous 24 months (83.7% vs. 80.1%, RR = 1.06 (95% CI 0.97, 1.15), *p* = 0.20)  Significantly larger proportion of patients in intervention group had had their total and high-density lipoprotein cholesterol recorded in the previous 24 months (75.5% vs. 66.5%, RR = 1.19 (95% CI 1.03, 1.37), *p* = 0.02)  Medication management of patients with cardiovascular disease and those at high risk:  1. Primary outcome – No significant difference in proportion of patients in intervention and control groups, respectively, receiving guideline-indicated medication prescriptions (56.8% vs. 51.2%, RR = 1.11 (95% CI 0.97, 1.27), *p* = 0.12)  2. Secondary outcomes- no difference between intervention and control groups in proportions of patients:  i) At high risk and with current prescription for ≥ one blood-pressure-lowering medication and a statin (58.3% vs. 54.1%, R= 1.09 (95% CI 0.97, 1.22), *p* = 0.16)  ii) With cardiovascular disease and with ≥ one blood-pressure-lowering medication, a statin, and an antiplatelet medication  (55.3% vs. 48.4%, RR = 1.14 (95% CI 0.97, 1.35), *p* = 0.10)  Significantly greater proportion of patients in intervention group treated by:  i) Escalating antiplatelet medications in those with cardiovascular disease (17.8% vs. 2.7%, RR = 4.79 (95% CI 2.47, 9.29), *p* < 0.001)  ii) escalating lipid-lowering medication (19.2% vs. 4.7%, RR = 3.22 (95% CI 1.77, 5.88), *p* < 0.001)  iii) escalating blood-pressure-lowering medications (23.3% vs. 12.1% RR = 1.89 (95% CI 1.09, 3.28), *p* = 0.02) |
| Peter [104]  2015  The Netherlands  Physiotherapy guideline on hip and knee osteoarthritis | RCT  237 physiotherapists | Context: new guideline  To determine, on the national level, the effectiveness of a guideline-based educational intervention | Theory: NR  Tailored to pre-identified barriers: NR  Barriers pre-identified at baseline, and then assessed again at 2 more points in the study through a self-developed questionnaire (van der Wees et al., 2013) specifically pertaining to the Dutch physiotherapy guideline.  Co-designed with patients or professionals: NR | | Target: Professionals  Intervention: Single  Type: PROFESSIONAL: Educate groups about guideline intent/benefit  Content: Information about the guideline  Format: Educational course (developed and evaluated in previous pilot study)  Delivery: In-person  Timing: T1 – 1 week prior to intervention  T2 – Immediately after intervention  T3 – 3 months after intervention  May 2011 to January 2012  Personnel: Expert physiotherapist, 3 to 4 patients and 3 to 4 physiotherapy teachers  Control: The control group received the same educational course 4 months after the first course in every region. | Mixed results - positive change in the self-reported items measured by the study; Satisfaction comparable to control  It was an effective intervention to enhance self-reported knowledge of and self-reported adherence (mean difference in change scores [95% CI]: T1 – 1.4 [0.7, 2.0]; T2 – 0.9 [0.2, 1.7], *p* < 0.005) to a physiotherapy guideline on hip and knee osteoarthritis.  An effect on perceived barriers was only seen on the longer term.  The overall satisfaction with the intervention was good (comparable to satisfaction levels in the control group). |
| Shelton [105]  2015  USA  Prostate cancer screening | Prospective interrupted time- series  Outpatient clinics in greater Los Angeles with  30,150 male veterans aged 75+  Clinician qualifications NR | Context: Quality improvement  To determine impact of a highly specific computerized decision support alert on unwarranted prostate-specific-antigen-based (PSA-based) screening | Theory: NR  Tailored to pre-identified barriers: NR  Co-designed with project team and leaders in primary care | | Target: Professionals  Intervention: Single  Type: PROFESSIONAL: Provide alerts when practice deviates;  STRUCTURAL CHANGES:  Information/communication technology + Quality improvement/performance measurement system  Content:  Pop-up alert/reminder order check in compliance with guidelines for PSA-based prostate cancer screening; option of continuing or cancelling the order for screening, no justification required    Format: Electronic decision support tool  Delivery: Electronic health record  Timing:  Implementation over 30 months (March 2011-August 2013)  Periods of no reminder:  15-month-period prior to intervention (baseline) [March 2011-May 2012]  1^st^ 3-month “off” period (September –December 2012)  2^nd^ 3-month “off” period (May-August 2013)  Periods of reminder:  Two periods between above periods  Personnel: Project team consisted of two internists, Chief of Informatics, RN Clinical Applications Coordinator, a urologist and informaticist, Chief of Urology  Control: Intervention arm was its own control during off periods | Positive effect  Mean monthly screening rate before intervention: 8.3%  Mean monthly screening rate after intervention: 4.6%  Decrease in screening rates from “off” periods to “on” periods   - first: 6.7% to 2.7% - second: 5.0% to 3.5%   Increase in screening rates from “on” periods to “off” periods:   - first: 2.7% to 5.0% - second: 3.5% to 6.6%   Changes in screening rate ratio within a period:  First reminder “on” vs. baseline   - 0.78 vs. 0.97, *p* < 0.0001   Second reminder “on” vs. baseline   - 0.90 vs. 0.97, *p* = 0.056     First reminder “off” vs. baseline   - 1.16 vs. 0.97, *p* < 0.0001   First reminder “off” vs. first reminder “on”   - 1.16 vs. 0.78 (*p* < 0.0001)   Second reminder “off” vs. baseline   - 1.16 vs. 0.97, *p* < 0.0001   Second reminder “off” vs second reminder “on”   - 1.16 vs 0.90 (*p* < 0.0001) |
| Sherrard [106]  2015  Canada  Acute coronary syndrome | RCT  640 patients were in the intervention group and 707 patients received usual care. | Context: Quality improvement  To determine whether acute coronary syndrome patients who are contacted using interactive voice response technology are more likely to be receiving care as recommended by best practice guidelines at one year, as compared to patients who received usual care | Theory: NR  Barriers identified in the literature were general barriers to guideline adherence across medicine:   - Lack of time - Lack of resources - Lack of guidelines   (Cabana et al., 1999)  Tailored to pre-identified barriers: NR  Co-designed with professionals: The IVR system uses a series  of questions that were developed by an expert  multidisciplinary working group | | Target: Patients  Intervention: Single  Type: STRUCTURAL CHANGES: Information/communication technology  Content: Interactive voice response uses a speech recognition technology where patients receive automated phone calls at regular intervals based on a pre-set series of questions. The technology allows the patients to respond to questions in their own voice, receive health information or request services or care.  Format: Patients in the interactive voice response group received automated telephone calls at one, three, six, nine, and 12 months post discharge.  Delivery: Phone calls  Timing: Each call lasted five to eight minutes.  Personnel: Cardiac nurses, RNs  Control: Usual care | Positive change  A significant difference (66.1% vs. 54.9%, relative risk (RR) 1.60, 95% CI 1.29 to 2.00, *p* < 0.001) in the primary composite outcome of ‘increased compliance with medications and decreased adverse events’ was found in the interactive voice response group compared to the usual care group.  There was also a significantly lower likelihood with interactive voice response for unplanned visits to a primary care physician (6.3% vs. 9.6%, RR 0.63, 95% CI 0.42 to 0.94, *p* = 0.023).  There were no significant differences between the interactive voice response and usual care groups, respectively, in the secondary outcomes of ER visits (14.7% vs. 18.3%, RR 0.77, 95% CI 0.58 to 1.03, *p* = 0.079) and hospitalization (13.0% vs. 13.0%, RR 1.00, 95% CI 0.73 to 1.37, *p* = 0.981).  Patient satisfaction with interactive voice response was high. |
| Terasaki [107]  2015  USA  Outpatient management of stable chronic obstructive pulmonary disease | Cross-sectional, pre- post  144 pre-intervention and 267 post-intervention patients (diagnosis based on spirometry findings) at a tertiary care academic clinic | Context: Quality improvement  To determine whether a guideline-derived flowsheet would improve adherence to guidelines for the care of chronic obstructive pulmonary disease | Theory: NR  Barriers pre-identified in the literature were:   - 24% of physicians unaware of guideline for this disease   (Overington et al., 2014)   - View that this chronic obstructive pulmonary disease is self-inflicted with no effective treatment, resulting in less aggressive measures to manage it   (Sharif et al., 2013)  Tailored to pre-identified barriers: NR  Co-designed with professionals: Flowsheet was reviewed by pulmonary specialists for consensus approval (method NR) | | Target: Professionals  Intervention: Single  Type: STRUCTURAL CHANGES: Information/communication technology + Integration of services  Content: Electronic flowsheet containing guideline-concordant prompts for clinician investigation and blank fields for pertinent metrics  Format: Incorporation of flowsheet into existing electronic health record  Delivery: Electronic health record  Timing: Pre-intervention data from July 2011-June 2012, Post-intervention data from March 2013-February 2014  Personnel: Physicians  Control: Pre-intervention patients | Mostly positive results  Average forced expiratory volume in 1 second was more severe in post-intervention group (N = 267) compared to pre-intervention group (N = 144, 48.9 (standard deviation (SD) 18.7) vs. 55.7 (SD 20.1), *p* < 0.001), consistent with study’s finding of moderate or advanced disease in most post-intervention group patients  Significant increase between pre- and post- periods in the completion of the following assessments:   - Modified Medical Research Council Dyspnea Scale   (11.8% vs. 50.9%, *p* < 0.001)   - Body mass index, airflow obstruction, dyspnea and exercise capacity score   (13.2% vs. 32.2%, *p* < 0.001)   - Osteoporosis screening   (20.8% vs. 44.9%, *p* < 0.001)  No difference between pre- and post-intervention rates of following assessments:   - Chest CT (64.6% vs. 63.6%, *P* = 0.85) - Depression screening (17.4% vs. 23.2%, *P* = 0.17) - Alpha-1-antitrypsin screening (20.8% vs. 15.7%, *P* = 0.19)   Significant increase between pre- and post- periods in following interventions:   - Documented inhaler technique (35.4% vs. 65.2%, *p* < 0.001) - Influenza vaccination (74.3% vs. 83.5%, *p* = 0.03) - Referral to pulmonary rehabilitation program (13.2% vs. 19.4%, *p* = NR) - Short acting beta-agonist prescribed (88.8% vs. 95.5%, *p* = 0.01) - Short acting muscarinic agonist prescribed (40.9% vs. 52%, *p* = 0.03) - Long acting beta agonist prescribed (1.3% vs. 10.8%, *p* < 0.001)   No difference between pre- and post- periods in exacerbation of disease in one year prior to clinic visit as measured by:   - Visit to primary care physician (24.3% vs. 31.2%, *P* = 0.15) - Emergency room visit (18.1% vs. 24%, *P* = 0.17) - Hospitalization (22.2% vs. 25.5%, *P* = 0.67)1 |
| Ballesca [108]  2014  USA  Acute care for myocardial infarction | Retrospective cohort study  5879 patients hospitalized with acute myocardial infarction  Those whose admitting physician used the acute myocardial infarction order set: 3531 patients  Those whose admitting physician selected individual orders: 2348 patients | Context: Quality improvement  To determine whether use of an electronic order set for acute myocardial infarction resulted in better hospital processes and patient outcomes; and whether associations remained after adjusting for risk using a special scoring system | Theory: NR  Tailored to pre-identified barriers: NR  Co-designed by professionals: The order set was developed by a multidisciplinary expert panel (number and qualifications NR) based on the literature and national guidelines | | Target: Professionals  Intervention: Single  Type: STRUCTURAL CHANGES: Information/communication technology + Quality improvement, performance measurement system + Integration of services  Content: Order set provides a list of:  (i) 4 required medication activities  (ii) 6 optional medications/medications as needed activities  (iii) 6 laboratory tests  (iv) 3 other tests  (v) Cardiology consultation automatically initiated when order set is opened  Format: Electronic order set  Delivery: Electronic health record  Timing: Patients hospitalized between 28 September 2008 – 31 December 2010  Personnel: NR  Control: Patient data (n = 2348) in which admitting physician selected orders individually rather than using the order set | Positive impact  Order set patients were more likely to received evidence-based medication types, all *P* < 0.0001:   - Aspirin (98.3% vs. 93.8%) - Anticoagulants (81.7% vs. 78.6%) - Beta-blockers (90.5% vs. 82.0%) - ACE inhibitors or ARBs (67.8% vs. 53.0%) - Statins (94.5% vs. 84.1%)   Order set patients had, all  *P* < 0.0001:   - Lower in-hospital mortality (3.51% vs. 6.52%) - Lower mortality within 30 days (5.66% vs. 8.48%)   Significantly more order set patients received, all *P* < 0.0001):   - ≥1 medication types (100.0% vs. 99.2%) - ≥2 medication types (99.7% vs. 96.5%) - ≥3 medication types (97.4% vs. 88.8%) - ≥4 medication types (85.4% vs. 70.1%) - all 5 medication types (50.3% vs. 35.9%)   ≥3 medication types (AOR 0.49, 95%CI 0.33, 0.79); ≥4 medication types (AOR 0.29, 95%CI 0.20, 0.42); ≥5 medication types (AOR 0.17, 95%CI 0.11, 0.25) reduced inpatient mortality  However, significantly fewer order set patients were admitted into the hospital at full code (86.1% vs. 88.0%, *P* = 0.0379), having had an ST-segment elevation myocardial infarction (4.7% vs. 15.7%,  P < 0.0001) and significantly fewer had ever had to undergo intensive care (36.3% vs. 49.8%,  *P* < 0.0001)  Order set patients also had, all  *P* < 0.0001):   - Lower severity of illness (Laboratory-based Acute Physiology Score, version 2 mean ± SD scores 35.6 ± 43.5 vs. 40.9 ± 48.1) - Fewer comorbidity burdens (Comorbidity Point Score version 2 mean ± SD scores 29.8 ± 31.7 vs. 34.3 ± 34.4; Charlson Comorbidity Index score mean ± SD scores 2.5 ± 1.5 vs. 2.7 ± 1.6) - Lower predicted percentage mortality risk (mean % ± SD scores 3.2% ± 7.4% vs. 4.8% ± 10.8%)   Order set patients happened to be lower risk, but even after the authors controlled for confounding variables and adjusted for bias using propensity scores, the use of the order set was still associated with increased use of evidence-based therapies and reduced mortality. |
| Cabilan [109]  2014  Australia  Peripheral intravenous devices in general surgery | Cross-sectional, pre- post  40 inpatients in an acute care general surgery ward, convenience samples of 20 each in first and second audits | Context: New guideline  To investigate whether intervention promotes adherence to best practice in monitoring and optimal replacement of peripheral intravenous devices, and whether nursing documentation improves | Theory: NR  Barriers identified by staff during first educational meeting were:  (1) Lack of awareness of best practice  (2) Hospital policy not in line with latest guidelines  (3) Lack of standard documentation  Intervention was tailored to pre-identified barriers.  Co-designed with professionals:  Strategies developed in collaboration with staff to overcome (1) were audit and feedback, educational meetings, and written reminders; to (2): updating of hospital policy; communication of policy update in educational meetings, an education session in May 2012, and in a quiz; to (3): documentation of intravenous line change in care pathway and audit progress notes | | Target: Professionals  Intervention: Multifaceted  Type: PROFESSIONAL:  Present guideline materials at meetings + Provide feedback on compliance + Educate groups about guideline intent/benefits + Educate individuals about guideline intent/benefits + Print material (reminders) + Enable self-audit (material)  Content: Guideline-derived criteria related to intravenous cannula and administration sets; creation of audit tool; results of baseline audit; update of hospital policy; changed documentation  Format: Educational meetings (group) or one-on-one meetings, audit and feedback, reminders, modified documentation  Delivery: In person, printed material  Timing:   - Baseline audit Monday-Wednesday, 6:30am-3:00pm, December 2011-January 2012 until sample size of 20 was met - 15-minute educational meetings or one-on-one meetings (n = NR) - Second audit: Same days and times, March 2012-August 2012     Personnel: Clinical facilitator ensured communication of updated hospital policy | Mixed results  Moderate compliance in monitoring and optimal replacement (*p*-values NR for all)  Guideline-recommended practices at baseline audit vs. second audit:   - Date of insertion of cannula documented: 75% vs. 90% - Cannula re-siting at 48h-72h: 40% vs. 25% - Cannula re-siting at 96h: 25% vs. 35% - Documentation of cannula removal/re-siting: 15% vs. 45% - Sets replaced: 0% vs. 25% - Daily site checks (self-report by nurses): 10% vs. 5% - Cannula flushes: 20% vs. NR   Compliance with documentation remained a challenge |
| Cahill [110]  2014  Canada  Nutrition adequacy in critically ill patients requiring enteral feeding | Pre- post feasibility study  5 sites comprising 7 ICUs in the USA and Canada with138 patients in total | Context: Quality improvement  To determine whether a multifaceted, interdisciplinary, tailored intervention would improve adherence to critical care nutrition guidelines | Theory: Knowledge-to-action model (Graham et al., 2006), Cahill et al.’s framework for understanding barriers (Cahill et al., 2010), Barriers Identification and Mitigation Tool (Gurses et al., 2009)  Tailored to barriers pre-identified through:   - A previous study by the study team (not related to present project) (Jain et al., 2006) - General literature on overcoming barriers (Cochrane et al., 2007)) - A self-developed questionnaire and barrier analysis   The questionnaire contained 21 potential barriers within 5 subscales, and was later analyzed in a barrier analysis.  The action plans that were part of the intervention were tailored to each site’s identified barriers and the results of an audit.  Co-designed with professionals through a 1-day meeting: Prioritization of barriers, brainstorming of feasible solutions, and the development of a site-specific plan were done with the targets of the intervention | | Target: Professionals  Intervention: Multifaceted  Type: PROFESSIONAL: Present guideline materials at meetings + Educate groups about guideline intent/benefits + Recruit an opinion leader who recommends implementation + Provide feedback on compliance (audit stage) + Provide feedback from healthcare professionals + Print material (poster, checklist); ORGANIZATIONAL (Health professional): Reallocated or new role (local implementation leaders are also opinion leaders) + Create an implementation/multidisciplinary team; Tailored plan could allow for STRUCTURAL CHANGES  Content:  1. Audit/assessment and feedback: Site-specific nutrition performance data compared against guidelines and other ICUs’ performance  Sites could tailor their intervention to any or all of 2, 3, 4, 5.  2. Educational outreach visit:  i) Evidence supporting nutrition guidelines  ii) ways to optimize enteral nutrition  iii) rationale for tailored intervention  iv) feedback on performance  v) informal discussion  3. Tailored action plan: Site-specific interventions selected by identifying and prioritizing which barriers to target, brainstorming of solutions, development of step-by-step action plan  4. Performance coaching: Support to local guideline implementation team during implementation  5. Local opinion leaders (local guideline implementation team): their guidance influences attitudes and behaviours of peers  6. Networking meeting: Discussion of successes and challenges  Format:  1. Barriers to Feeding Critically Ill Patients questionnaire, benchmarked performance report, small group discussion  2 i), ii), iii) Interactive presentation  iv) and v) Grand rounds with providers and face-to-face discussions  3. Conference, workshops, bedside huddles, information sheets, posters, checklist  4. Teleconference calls  5 and 6. Informal discussion  Delivery: In-person, telephone/video conference, printed material  Timing:  Study lasted 24 months  Pre-implementation phase: 3 months  Implementation phase: 12 months  1. Completed during pre-implentation phase  2 i), ii), iii) 1-hour interactive presentation  3. Over 1 month: 1-day meetings per site during which there were noon-hour workshops  4. Bimonthly teleconference calls during implementation phase  5. Throughout study  6. Half-day meeting  Personnel:  1. NR  2. External nutrition expert  3. External research team met with local implementation team and key stakeholders (e.g., ICU manager, nurse manager, nurses, intensivists, dietitians)  4. External research team and local implementation team  5. Local opinion leaders (local implementation team): At least 1 of each: physician, dietitian, and nurse who were knowledgeable in nutrition  6. Local implementation teams of all participating sites | No change: positive but non-significant impact on 12 nutrition practice indicators  Non-significant increase between pre-implementation (n = 140) and implementation phase (n = 138) in all indicators (indicators where *p* is closest to 0.05 are reported here):   - Patients who achieved >80% caloric adequacy within 72 hours of admission (36 (26%) vs. 44 (32%), *p* = 0.45) - Caloric adequacy from total nutrition (42.9 (29.6%) vs. 49.0 (31.2%), *p* = 0.23) - Enteral nutrition initiated within 48 hours of admission (71 (65%) vs. 77 (75%), *p* = 0.16) |
| van Dijk [111]  2014  Netherlands  Treatment of anxiety disorders in specialized mental health care | Observational (intervention condition plus control condition in naturalistic cohort)  Psychologists, psychiatrists and psychiatric nurses overseeing 177 patients at two specialized mental healthcare centres  Intervention condition (95 patients with attrition to 78 at 1-year follow-up, and 58 at 2-year follow-up)  Control condition (82 patients with attrition to 65 at 1-year follow-up and 68 at 2-year follow-up) | Context: Quality improvement  To examine the effect of an intervention on adherence to guideline-prescribed treatment steps for psychotherapy and pharmacotherapy for generalized anxiety disorder, panic disorder with or without agoraphobia, or social phobia | Theory: Barriers to guideline adherence questionnaire based on Theory of Planned Behaviour  Tailored to pre-identified barriers: The implementation plan was based on a “diagnostic analysis of possible barriers to implementation”. The nature of the barriers was not reported, nor the method of determining them.  A qualitative questionnaire based on the Theory of Planned Behaviour was delivered to professionals in both conditions prior to the start of the intervention.    Co-designed with patients and professionals: Choice of treatment based on a series of options was determined jointly by the patient and the professional during a meeting held before the start of treatment | | Target: Patients and professionals  Intervention: Multifaceted  Type: PROFESSIONAL: Distribute guideline material + Educate groups about guideline intent/benefits + Print material (treatment steps, guidelines) + Provide feedback on compliance; PATIENT/CONSUMER: Education (single) + Counselling + Print material (treatment options); ORGANIZATIONAL (Health professional): Reallocated or new role; (Patient): Consumer feedback/suggestions/complaints  Content:  1. Shared decision-making between patient and treatment coordinator yielding a guideline-concordant treatment plan  2. Informational materials for patients (descriptions of care recommendations, from which patients could choose preferred treatment) and professionals (treatment steps, evidence-based recommendations)  3. Discussion of guidelines  4. Training of professionals in skills required to provide guideline-concordant care  5. Monitoring of guideline adherence    Format:  1. One-on-one meetings  2. Hard copy folders for patients, Desktop files and hard copy folder for professionals  3. Three educational meetings  4. NR  5. Chart review  Delivery: In person, electronic files, print material  Timing: Approximately 51-55 months, comprising 1 year of preparation, barriers assessment and data collection over the remaining 39-43 months  Each patient was followed for 2 years  3. Three educational meetings took up 12 hours total  Personnel: Treatment coordinator (experienced cognitive behavioural therapist), other psychologists, psychiatrists, psychiatric nurses, senior professionals from Dutch Knowledge Centre for Anxiety and Depressive Disorders, two independent assessors  Control: Passive guideline dissemination only | Mixed results  Data analyzed took into account significant differences at baseline, but no differences between raw and adjusted scores, so only raw scores reported  Treatment was guideline-adherent in 50/81 (61.7%) of cases in the intervention condition, whereas this figure was 28/69 (40.6%) in the control condition (chi-square = 6.68, *P* = 0.01)  There was a significantly greater reduction (by 4.1 points) in anxiety scores from baseline to 1-year follow-up in the intervention condition compared to the control condition (95% CI 0.84, 7.34; *P* < 0.05; Cohen’s *d* = 0.45)  Howev  The intervention condition also showed a difference of 3.0 points from baseline to 2-year follow-up in anxiety scores, compared to control, but this difference was not significant (95%CI 0.35, 6.44; *P* = 0.08; Cohen’s *d* = 0.36)  There was a significantly greater reduction (by 5.6 points) in fear scores from baseline to 1-year follow-up in the intervention condition compared to the control condition (95% CI 0.10, 11.02; *P* < 0.05; Cohen’s *d* = 0.34)  At 2 years, the decrease from baseline was 11.0 points greater in the intervention condition compared to control (95% CI 5.27, 16.74; *P* < 0.05, Cohen’s *d* = 0.68)  There were no significant differences between intervention and control groups in depression scores when comparing baseline to 1-year (2.26 point greater decrease in intervention condition (95% CI -0.95, 5.48; *P* = 0.167) and 2-year points (1.3 point greater decrease in intervention condition (95% CI -2.05, 4.69; *P* = 0.441)  Adherence to both psychotherapeutic and pharmacotherapeutic guidelines were assessed conservatively, on all-or-nothing basis (yes/no)  A significantly greater percentage of patients indicated for cognitive intervention received it in intervention group (77.5% vs. 59.3%, *p* = 0.028)  No differences between intervention and control groups, respectively, in adherence to the following recommended treatment steps: Exposure intervention (50% vs. 41.7%, *P* = 0.577), medication step 1 (66.7% vs. 71.9%, *P* = 0.675), medication step 2 (44.4% vs. 44.4%, *P* = 0.999), medication step 3 (40.0% vs. 25.0%, *P* = 0.999)  Percentage of patients responding to treatment at 1-year follow-up significantly higher in intervention condition (52.6% vs. 33.8%, chi-square = 5.04, *P* = 0.025)  Percentage of patients achieving remission at 1-year follow-up significantly higher in the intervention condition (33.3% vs.16.9%, chi-square = 4.98, *P* = 0.026)  At 2-year follow-up, there were no significant differences between intervention and control groups in percentage responding to treatment (48.3% vs. 44.1%, respectively; chi-square = 0.22, *P* = 0.641) and percentage of patients achieving remission (32.8% vs. 29.4%, respectively; chi-square = 0.16, *P* = 0.685)  Authors interpret study as showing that intervention yields faster improvements |
| Franx [112]  2014  Netherlands  Antidepressant prescribing in primary care | Quasi-experimental with a non-equivalent naturalistic control  135 GPs from 58 practices with 4356 patients with new depressive symptoms  Intervention group (20 GPs from 17 practices with 400 patients)  Control group (115 GPs from 41 practices with 3956 patients) | Context: Quality improvement  To determine whether a multifaceted intervention has an effect on antidepressant prescribing as first-step treatment, and referral to mental healthcare | Theory: Quality Improvement Collaborative approach/breakthrough method, Stepped care model for depression treatment, SMART goal setting  Barriers pre-identified in the literature pertaining to this model of care (quality improvement collaborative) for depression are:   - Staff resistance - Time constraints - Information technology difficulties   (Meredith et al., 2006)  Tailored to pre-identified barriers: NR  Co-designed with professionals as part of the collaborative approach | | Target: Patients AND professionals  Intervention: Multifaceted  Type: PROFESSIONAL:  Provide feedback on compliance (“Written feedback on improvement reports and data charts”) + Print material (Excel worksheet, instruments, treatment protocols); FINANCIAL (Health professional) Grant or allowance to group/institution (not tied to compliance); ORGANIZATIONAL (Health professional): Create an implementation/multidisciplinary team  Content:  Professional intervention:  1. Access to a multidisciplinary team  2. Teaching of stepped care model  3. Teaching of SMART goal setting: indicators that help monitor results  4. Training on breakthrough method  5. “Exchange and learning”  6. “Intensive exchange”  10. Written feedback on areas for improvement and data charts (sender, recipient NR)  11. Provision of virtual network for discussion of best practices  12. Training in problem-solving  13. Workflow improvement  Format:  Professional intervention:  1, 2, 4, 12, 13. In-person trainings/workshops  3. Training and Excel worksheet  5, 6. Conference days  7. Five meetings  8. Team visits  9. Telephone contact  10. Written feedback  11. Virtual network, instruments and treatment protocols  Delivery:  Professional intervention:  In person, telephone, online, online tools/documents  Timing:  Intervention over 15 months (2006-2008)  Professional intervention:  5. Four conference days for all intervention teams  6. One conference day  11. Two-day training  Personnel:  Professional intervention:  1. Multidisciplinary teams  2.. Expert team  4. Training for local team coordinators  5. Conference day for local team coordinators  6. Meetings between local team coordinators, with expert team presence  7. Telephone contact between local and national coordinators  Control: Usual care | Positive impact  Proportion of patients aged 18-35 higher in intervention group (37.5%) vs. control group (32.3%) (*p* = NR)  There was a decrease of 23.3% (49.4% to 26.1%) in antidepressant prescriptions for newly diagnosed patients with depressive symptoms in the intervention group, whereas no difference was found in the control group (50.3% to 52.6%), and the comparative change between the two groups was found to be significant (OR 0.44, 95% CI 0.21, 0.92; *P* < 0.05)  Intervention group:  Number of patients receiving antidepressant prescription in year 2 of study (2007) significantly lower than year 1: 32.2/152 vs. 49.4/87 (*p* < 0.05)  Number of patients receiving antidepressant prescription in year 3 of study (2008) significantly lower than year 1: 26.1/161 vs. 49.4/87 (*p* < 0.05)  No significant differences (*p* = NR) in number of intervention patients referred to mental healthcare within a month of appointment (2007 vs. 2006: 16.4/152 vs. 11.5/87, 2008 vs. 2006: 11.2/161 vs. 11.5/87)  Control group:  No significant differences in number of patients receiving an antidepressant prescription (2007 vs. 2006: 47.0/996 vs. 50.3/1261, 2008 vs. 2006: 52.6/1699 vs. 50.3/1261)  Number of control patients referred to mental healthcare within a month of appointment significantly higher in year 2 (2007) vs. year 1: 13.0/996 vs 10.1/1261 (*p* < 0.05)(*p* < 0.05) |
| Gupta [113]  2014  USA  Use of head CT scan for mild traumatic brain injury | Prospective cross-sectional, pre- post  Random sample of 400 patients with mild traumatic brain injury  Baseline period: 200 patients identified by chart review  Intervention period: 200 patients | Context: Quality improvement  Determine the impact of a clinical decision support on use of head CT for mild traumatic brain injury | Theory: NR  Tailored to pre-identified barriers: NR  Co-designed by patients or professionals: NR | | Target: Professionals  Intervention: Single  Type: PROFESSIONAL: Provide alerts when practice deviates + Tailor guideline (amalgamated and selected decision rules from various guidelines); STRUCTURAL CHANGES: Information/communication technology + Integration of services + Quality improvement/performance measurement system  Content: Question prompts that evaluated presence of mild traumatic brain injury, and that addressed guideline criteria to justify imaging  Format: Electronic clinical decision support system integrated with hospital’s computerized provider order entry system  Delivery: Electronic health record  Timing: Baseline period of 27 months (1 August 2007-31 October 2009)  Intervention period of 27 months (1 December 2009-29 February 2012)  Personnel: 2 attending physician chart abstractors/reviewers (to confirm mild traumatic brain injury against EMR)  Control: pre- data | Positive results  Documented guideline adherence was 49% (98/200) vs. 76.5% in (153/200) in baseline and intervention periods, respectively   - Absolute effect size: 27.5%   (*p* < 0.001)   - Relative effect size: 56.1%   (*p* < 0.001)  Sample of 50 charts from intervention period showed that 35 charts were concordant for guideline adherence in both the manual clinician note and the corresponding electronic decision support chart |
| Mold [114]  2014  USA  Management of chronic kidney disease | Prospective cohort study  89 primary practices comprising physician(s), and possibly physician assistants and nurse practitioners, serving patients with evidence of chronic kidney disease aged between 50-84 years  Wave I practices:  31 primary practices were members of 4 practice-based research networks  Wave II practices:  Each Wave I practice recruited 2 other primary practices, for a total of 58 Wave II practices  Each wave I practice and its 2 corresponding Wave II practices were known as local learning collaboratives | Context: Quality improvement  Determine:  (i) feasibility of ‘early adopter’ practices recruiting other practices, and helping the newly recruited practices implement guideline recommendations;  (ii) successfulness of the diffusion of 8 guideline-recommended processes of care:  (1) Chronic kidney disease defined as an estimated glomerular filtration rate <60 for 3 months  (2) Annual A1c measurement  (3) ≥Annual LDL cholesterol measurement  (4) ≥Annual microalbumin measurement  (5) Annual hemoglobin measurement if estimated glomerular filtration rate <45  (6) ACE inhibitor or ARB initiation  (7) Discontinuation of NSAIDs  (8) Nephrologist referral for estimated glomerular filtration rate <30 | Theory: Cooperative Extension (from agriculture), peer-to-peer learning, Chronic Care Model  Tailored to pre-identified barriers: NR  Co-designed by patients or professionals: NR | | Target: Professionals  Intervention: Multifaceted  Type: PROFESSIONAL: Present guideline material at meetings + Distribute guideline material + Educate groups about guideline intent/benefits + Provide feedback on compliance + Provide feedback from healthcare professionals (Wave I practices🡪Wave II practices) + Print material (one-page decision support tool); FINANCIAL (Health professional): Grant or allowance to individual (not tied to compliance: maintenance of certification/continuing medical education credit)  Content:  Training for personnel:   - Academic detailers were provided training on guideline recommendations and suggestions on implementation - Practice facilitators were provided training on quality improvement concepts, practice facilitation and associated skills, chart auditing and feedback, Chronic Care Model, complex adaptive systems, maintenance of certification, chronic kidney disease guidelines (including tool and implementation suggestions), facilitation of local learning collaborative meetings   Intervention for Wave I + Wave II practices: (31 practices + 58 practices)  (i) Performance evaluation,  (ii) Feedback reports,  (iii) Academic detailing: review of guideline recommendations, review and discussion of printed decision-support tool with specific guideline recommendations,  (iv) Practice facilitation: implementing changes in care processes through PDSA cycles, quality improvement teams, staff meetings; collecting unofficial baseline and monthly performance data  (v) Local learning collaborative meetings: after Wave II practices were involved, discussion of lessons learned, mutual review of performance data, sharing of electronic health record templates/order sets/other tools and materials developed  Format: In-person performance evaluation, academic detailing, and practice facilitation; printed one-page decision support tool; tools such as templates and/or order sets and/or patient handouts  Delivery: In person, print material  Timing:  Intervention was delivered in two separate waves, but precise timing of study components (e.g., practice facilitation) pertaining to each wave, and degree/presence of overlap between each wave’s post-intervention periods NR  Wave I   - Pre-intervention period: 12-month period before intervention; - Post-intervention period: 12-month period following the *beginning* of the intervention - 31 practices underwent 2-4 hours/week practice facilitation for 6 months   Wave II   - Pre-intervention period: 12-month period before the beginning of the intervention; - Post-intervention period: *9 months* following the *beginning* of the intervention - 58 practices underwent 2-4 hours/month practice facilitation   Waves I and II practices:  1-hour/month local learning collaborative meetings  Personnel: 1 project coordinator, 1 academic detailer, and 1 full-time equivalent practice facilitators for *each of 4* practice-based networks  A lead clinician from each wave I practice (n = 31) was responsible for recruiting wave II practices  In local learning collaborative meetings, wave I clinicians facilitated discussions | Mixed results  Results pertaining to Objective (ii)  Of 374 patients with glomerular filtration rates *<60* seen by *Wave I* practices in both pre- and post-intervention periods, a significantly greater proportion (69% vs. 63%, *p* = 0.002) were prescribed ACE inhibitors or ARBs in the *post-intervention period*  Of 138 patients with glomerular filtration rates *<45* seen by *Wave I* practices in both pre- and post-intervention periods, a significantly greater proportion had their hemoglobin measured (72% vs. 61%, *p* = 0.02) and had vitamin D testing/were provided a supplement (60% vs. 37%,  *p* < 0.001) in the *post-intervention period*  Of 105 *Wave I* patients with estimated glomerular filtration rate *<30* during the pre-intervention period, 66% (95% CI: 56%, 75%) were referred to a nephrologist, whereas 71% (95% CI: 62%, 79%) of 124 with estimated glomerular filtration rate *<30* during the post-intervention period were referred, *p* = NR  Of 660 patients with glomerular filtration rates *<60* seen by *Wave II* practices in both pre- and post-intervention periods, a significantly greater proportion (63% vs. 55%, *p* < 0.001) were prescribed ACE inhibitors or ARBs in the *post-intervention period*  Of 233 patients with glomerular filtration rates *<45* seen by *Wave II* practices in both pre- and post-intervention periods, a significantly greater proportion had vitamin D testing/were provided a supplement (57% vs. 37%, *p* < 0.001) in the *post-intervention period* (hemoglobin measurement was not required in Wave II)  Of 60% (95% CI: 51%, 68%) of 129 *Wave II* patients with estimated glomerular filtration rate *<30* referred to a nephrologist during the pre-intervention period reduced to 55% (95% CI 47%, 63%) of 176 with estimated glomerular filtration rate *<30* referred during the post-intervention period, *p* = NR  There were no significant differences pre- and post-intervention in either *Wave I* patients or *Wave II* patients with glomerular filtration rates *<60* in:   - Taking NSAIDs - Performance of A1c testing - Performance of urine microalbumin testing - Performance of LDL cholesterol testing - A1c test results - Mean LDL cholesterol levels (*Wave I* only) - Systolic blood pressure - Diastolic blood pressure   There was a significant improvement in mean LDL cholesterol levels post-intervention (92.0 mg/dl vs. 96.3 mg/dl,  *p* = 0.004) in *Wave II* patients with estimated glomerular filtration rates *<60*  However, there was sometimes an interaction between the effect of the intervention and when a patient’s chronic kidney disease was detected. Data below is adjusted for timing of recognition of chronic kidney disease (i.e., pre-intervention or post-intervention)  *Wave I* patients showing signs of chronic kidney disease were significantly more likely to:   - Be prescribed ACE inhibitors/ARBs (OR 1.3; 95%CI 1.1, 1.6;   *p* = 0.001)   - Not be taking NSAIDs (OR 0.7; 95%CI 0.4, 1.0;   *p* = 0.030)   - Have hemoglobin measured (OR 1.7; 95%CI 1.1, 2.6;   *p* = 0.014)   - Have vitamin D measured (OR 2.6; 95%CI 1.7, 3.8;   *p* < 0.0001)  in the *post-intervention period* even after adjusting for timing of recognition of chronic kidney disease  For *Wave I* patients with evidence of chronic kidney disease, the interaction between *the intervention* and *recognition + timing of recognition* of chronic kidney disease had a significant impact on:   - A1c testing (OR 1.4; 95% CI 1.0, 1.9; *p* = 0.037) - Measurement of microalbumin   (OR 1.6; 95%CI 1.1, 2.1;  *p* = 0.004),  with patients recognized as having chronic kidney disease pre-intervention significantly more likely to be tested for both in the post-intervention period  *Wave II* patients showing signs of chronic kidney disease were significantly more likely to:   - Be prescribed ACE inhibitors/ARBs (OR 1.4; 95%CI 1.2, 1.6; *p* < 0.001) - Have vitamin D measured (OR 2.2; 95%CI 1.7, 2.9;   *p* < 0.0001)  in the *post-intervention period* even after adjusting for timing of recognition of chronic kidney disease  No change in measurement of hemoglobin among *Wave II* patients pre- vs. post-intervention, but significant effect of timing of detection of chronic kidney disease; those recognized as having chronic kidney disease pre-intervention were significantly less likely to have hemoglobin measured in the post-intervention period than those recognized as having chronic kidney disease in the post-intervention period itself (OR 0.4; 95%CI 0.2, 0.8; *p* = 0.006)  Probability of *Wave II* patients having microalbumin measured increased in the post-intervention period, but not significantly: OR 1.2; 95% CI 1.0, 1.5; *p* = 0.069), after adjusting for timing of chronic kidney disease recognition  No significant difference pre- vs. post-intervention in *Wave II* patients taking NSAIDs (OR 0.9; 95%CI 0.7, 1.2; *p* = 0.35) |
| Peng [115]  2014  China  Secondary prevention of ischemic stroke | Cluster RCT  47 tertiary referral centres and hospitals across China comprising 2717 patients (at 12-month follow-up) having had a stroke  Intervention group (23 hospitals comprising 1287 patients at 12-month follow-up)  Control group (24 hospitals comprising 1430 patients at 12-month follow-up) | Context: Quality improvement  To determine whether a neurologist- managed intervention had an impact on patient adherence to secondary stroke prevention measures | Theory: NR  Barriers identified in the literature are (from study protocol, Peng et al., 2011):   - Patient adherence - Physician knowledge - Socioeconomic development   (Hasdai et al., 2002; Fox et al., 2002)  Tailored to pre-identified barriers: NR  Co-designed by patients or professionals: NR | | Target: Patients  Intervention: Multifaceted  Type: PATIENT/CONSUMER: Education (single) + Counselling + Print material (educational); ORGANIZATIONAL (Health professional): Reallocated or new role (study neurologist)  Content:  i) Pharmaceutical treatment by neurologists geared toward specific stroke subtypes  ii) Patient education on lifestyle modification: healthy diet, tobacco cessation, regular exercise  iii) Patient education on importance of adherence to program: prevention of ischemic stroke, risk-factor control, lifestyle changes  Format: Neurologist prescriptions, Patient-clinician discussions, Interactive patient education consisting of access to study’s stroke-prevention website, Written material  Delivery: In-person, online, printed material  Timing: Recruitment and randomization over 1.5 years (April 1 2008 – December 31 2010)  Outcome measures collected 12 months post-discharge  Personnel: Study neurologist/s  Control: Care as usual (care by the control clinic’s neurologist-clinician) | Mixed results  No significant differences in proportions of medications (antiplatelet, antihypertensive, antidiabetic, statin, anticoagulant)  prescribed at discharge between intervention and control groups  Medication adherence at 6 months post-stroke:  No significant differences between intervention and control groups in adherence to any medication class EXCEPT statins (*P* = 0.005) where adherence was higher in intervention group  Medication adherence at 12 months post-stroke:  No significant differences between intervention and control groups in adherence to any medication class EXCEPT statins (*P* = 0.006) where adherence was higher in intervention group  No significant differences were observed between intervention and control groups, respectively, in all end-point patient-related events (3.50% (63/1795) vs. 3.59% (73/2026), hazard ratio 0.982 [95% CI 0.694, 1.391], *P* = 0.921), comprising new-onset ischemic stroke, transient ischemic attack, hemorrhagic stroke, acute coronary syndrome, and all-cause death |
| Pimenta [116]  2014  Brazil  Management of hypertension in primary health care | RCT  41 physicians  ‘Active’ intervention group: 21 physicians and 181 patients  ‘Passive’ intervention group: 20 physicians and 136 patients | Context: Quality improvement  To investigate the effects of 2 educational interventions on the treatment of hypertension in primary care in low-resources settings | Theory: Transtheoretical model from the addictive behaviours field (Prochaska et al., 1992)  Tailored to pre-identified barriers: NR  Co-designed by patients or professionals: NR | | Target: Professionals  Intervention: Multifaceted  Type: PROFESSIONAL: Distribute guideline material + Present guideline materials at meetings + Educate individuals about guideline intent/benefits + Educate groups about guideline intent/benefits + Provide reminders to individuals/groups about intent/benefits + Provide feedback from healthcare professionals (cardiologist) + Print material (guideline, tool)  Content: Physicians were part of a continuing medical education program based on peer review groups.  (1) Workshop incorporated into meeting of peer review groups –each physician presented to the group a clinical case (prepared by the group supervisor) developed from a real patient of theirs; presented individual opinion on diagnosis, management and comparison with guidelines (copy provided); subsequent group discussion and drawing parallels with other clinical cases, comparing once more with guidelines  (2) Cardiologist visit to physician’s workplace: Discussion of doubts or questions posed by physician; supplied a hard-copy risk assessment tool  (3) Risk stratification reminders, measures for high-risk patients, guidance on treatment and advice to give patients  Format: (1) Workshop, (2) Cardiologist visit, risk assessment tool, (3) 3 email/text messages  Delivery: In person, printed material, email, SMS  Timing: Six-hour workshop, Cardiologist visit 2-3 weeks after workshop,  Personnel: Group supervisor (whether study team or not NR)  Control: Passive intervention – provision of risk assessment tool and copy of guidelines | Positive effects  The active intervention group outperformed the passive intervention group in several measures:   - Any improvement in treatment: 79.6% vs. 51.5%,   *p* < 0.001   - Improvement in use of anti-hypertensive drugs: 33.1% vs. 19.1%, *p* = 0.005 - Aspirin prescription: 44.2% vs. 25%, *p* < 0.001 - Aspirin prescription for high-risk patients: 18.2% vs. 5.9%, *p* < 0.001 - Lipid-lowering drug prescription: 38.7% vs. 21.3%, *p* = 0.001 - Lipid-lowering drug prescription for high-risk patients: 17.7% vs. 6.6%,   *p* < 0.001   - Diet counselling: 75.7% vs. 61%, *p* = 0.004 - Counselling about follow-up visit: 20.4% vs. 11.8%,   *p* = 0.040  The differences between groups in the above actions stemmed mainly from higher rates of treatment for high-risk patients in the active intervention group  However, neither intervention evinced changes in patient behaviour. |
| dos Santos [117]  2014  Sweden  Appropriate medication and cardiac function in cardiac intensive care | Quasi-experimental (patients assigned to cardiac units based on location and disease)  Intervention group: Between 146-251 patients in a single cardiac intensive care unit  Intervention unit: 26 physicians  Control group: Between 869-1771 eligible patients in 7-10 cardiac intensive care units  Control units: professionals NR | Context: Quality improvement  To determine whether a clinical decision-support system had an impact on five quality indicators of guideline adherence in the care of acute coronary syndrome | Theory: NR  Tailored to pre-identified barriers:  Barriers identified through the literature include (Wong et al., 2000; den Hertog et al., 2005):   - Difficulties securing funding - Insufficient time and knowledge - Irrelevance of guideline improvement initiative to career progression   Literature-identified barriers relevant to clinical decision support systems (Bates et al., 2001; Bates et al., 2003; Varonen et al., 2008):   - Initial investment - Number of reminders - Previous IT failures - Concerns about impact on the physician-patient relationship - Threats to professional autonomy   Clinical decision support system co-designed by a physician and IT developers (professionals, component) | | Target: Professionals  Intervention: Single  Type: STRUCTURAL CHANGES: Information/communication technology + Quality improvement, performance measurement system  Content: Clinical decision support system with information on:   - Patient current health status: Reports, patient background - Baseline characteristics of patient: Whether patient has gone various procedures, e.g., coronary angiography, echocardiography - National guidelines: Real-time, requested, or batch-delivered notifications of deviations; notification solicits a reason for the deviation   Format: Clinical decision support system integrated with electronic health record and national quality improvement registry  Delivery: Electronic health record  Timing: Pre-intervention: 2002  Post-intervention (short-term): 2003  Post-intervention (long-term): 2004-2008  Personnel: In intervention group, two specialists (6 doctors filled this position) and two assistant physicians (20 doctors filled this position)  Control: Care as usual | Positive impact  Measures of 4 quality indicators prior to the intervention (year 2002) among eligible acute myocardial infarction patients showed significant guideline-divergence in the intervention unit compared to control units in one indicator (all other indicators not significantly different):   - Lipid-lowering treatment after infarction: 49% in intervention unit (N = 251) vs. 66% in eight control units (N = 1768), *p* < 0.001   Short-term post-intervention (year 2004) quality indicators were significantly better in the intervention unit compared to control units (% of eligible patients reported):   - Coronary angiography values within range without ST elevation: 81% (N = 120) vs. 58% in seven control units   (N = 869), *p* < 0.001   - Subcutaneous/intravenous low-molecular-weight heparin or percutaneous coronary intervention within 24 hours without ST elevation: 92%   (N = 150) vs. 80% in seven control units (N = 970),  *p* < 0.001   - Lipid-lowering treatment after infarction: 90% (N = 227) vs. 73% in eight control units   (N = 1677), *p* < 0.001   - ACE-inhibitor/beta-blocker treatment after infarction: 89% (N = 163) vs. 64% in eight control units (N = 1203), *p* < 0.001   Long-term post-intervention (years 2004-2008) quality indicators were significantly better in the intervention unit compared to ten control units (average % and number of eligible patients/year reported, *p*-value, OR [95% CI]):   - Coronary angiography values within range without ST elevation: 83% (N = 99) vs. 62% (N = 948), *p* < 0.001, 1.2 [1.1, 1.2] - Subcutaneous/intravenous low-molecular-weight heparin or percutaneous coronary intervention within 24 hours without ST elevation: 96% (N = 100) vs. 89% (N = 1210), *p* < 0.001, 1.2 [1.2, 1.3] - Lipid-lowering treatment after infarction: 94% (N = 238) vs. 84% (N = 1978), *p* < 0.001, 1.3 [1.3, 1.4] - ACE-inhibitor/beta-blocker treatment after infarction: 91% (N = 180) vs. 74% (N = 1427), *p* < 0.001, 1.2 [1.1, 1.2] - Use of clopidogrel after infarction without ST elevation: 87% (N = 102) vs. 65% (N = 1031), *p* < 0.001, 1.7 [1.6, 1.7]   Annual change in odds of being prescribed guideline-concordant therapy in the intervention unit (% change, [95%CI]:   - Coronary angiography values within range without ST elevation: 13% [-5%, 33%] - Subcutaneous/intravenous low-molecular-weight heparin or percutaneous coronary intervention within 24 hours without ST elevation: 43% [2%, 101%] - Lipid-lowering treatment after infarction: 23% [4%, 46%] - ACE-inhibitor/beta-blocker treatment after infarction: 12% [-4%, 32%] - Use of clopidogrel after infarction without ST elevation: 32% [9%, 59%] |
| Sonstein [118]  2014  USA  Corticosteroid prescription in chronic obstructive pulmonary disease (COPD) | Pre- post  Residents and faculty (n = NR) at a tertiary care hospital, overseeing 420 patients hospitalized for acute exacerbations of COPD  Pre-intervention: 203 patients  Post-intervention: 217 patients | Context: Quality improvement  To determine whether the use of an order set had an impact on adherence to chronic obstructive pulmonary disease guidelines, as measured by disease-specific process of care and outcome variables, particularly reduced use of corticosteroids | Theory: NR  Tailored to pre-identified barriers: NR  Co-designed with professionals: The order set was developed with input from primary care physicians and pulmonary specialists (method NR) | | Target: Professionals  Intervention: Multifaceted  Type: PROFESSIONALS: Advertise guideline material; STRUCTURAL CHANGES: Information/communication technology + Method of service delivery + Integration of services  Content: Acute exacerbation of COPD electronic order set included management options during hospitalization, orders to schedule follow-up with patient’s primary care provider  Format: Electronic order set integrated into hospital’s electronic health record  Delivery: Electronic health record  Timing:  Study took place over 45 months (1 January 2009 – 30 September 2012)  Pre-intervention period: 26 months (1 January 2009 – 28 February 2011)  Post-intervention period: 19 months (1 March 2011 – 30 September 2012)  Personnel: NR | Positive results  Positive results particularly for primary outcome measure: corticosteroid use  Process of care variables:  Significantly lower (guideline-concordant) in post- vs. pre-intervention period:   - Corticosteroid use in first 48 hours of hospitalization, in prednisone-equivalent mg   (306.2 mg vs. 156.25 mg, *P* < 0.0001)   - Corticosteroid use during entire period of hospitalization, in prednisone-equivalent mg (352.5 mg vs. 175 mg, *P* < 0.0001)   Significantly higher rates of use in post-intervention vs. pre-intervention period in (relevance to guideline concordance NR):   - Long-acting muscarinic agonist (13% vs. 25%, *P* = 0.002) - Pneumococcal vaccination   (45% vs. 57%, *P* = 0.019)   - Mechanical ventilation   (0% vs. 3.7%, *P* = 0.02)  Outcome variables:  No significant differences in:   - Median length of hospital stay in days (3, interquartile range 2-4 vs. 3, interquartile range 2-4, *P* = 0.9909) - Rates of patient keeping of 15-day post-discharge follow-up appointments (47% vs. 48%, *P* = 0.8196) - Rates of patient keeping of 30-day post-discharge follow-up appointments (64% vs. 57%, *P* = 0.1817) - 30-day readmission for COPD (9% vs. 10%, *P* = 0.9170) - 30-day readmission for all causes (22% vs. 20%, *P* = 0.7230   between pre- and post-intervention periods |

NR = not reported

CI = confidence interval

OR = odds ratio

AOR = adjusted odds ratio

References

1. Fally M, Diernaes E, Israelsen S, Tarp B, Benfield T, Kolte L, Ravn P: **The impact of a stewardship program on antibiotic administration in community-acquired pneumonia: Results from an observational before-after study**. *Int J Infect Dis* 2021, **103**:208-213.

2. Ingram A, Valente M, Dzurec MA: **Evaluating Pharmacist Impact on Guideline-Directed Medical Therapy in Patients With Reduced Ejection Fraction Heart Failure**. *J Pharm Pract* 2021, **34**(2):239-246.

3. Akamike IC, Okedo-Alex IN, Uneke CJ, Uro-Chukwu HC, Chukwu OE, Ugwu NI, Agbo UN, Ezeoha AE: **Evaluation of the effect of an educational intervention on knowledge and adherence to HIV guidelines among frontline health workers in Alex-Ekwueme Federal University Teaching Hospital Abakaliki, Nigeria**. *Afr Health Sci* 2020, **20**(3):1080-1089.

4. Azizoddin DR, Lakin JR, Hauser J, Rynar LZ, Weldon C, Molokie R, Enzinger AC, Payvar S, Martin JL: **Meeting the guidelines: Implementing a distress screening intervention for veterans with cancer**. *Psychooncology* 2020, **29**(12):2067-2074.

5. Azubuike UC, Cooper D, Aplin-Snider C: **Using United States Preventive Services Task Force Guidelines to Improve a Family Medicine Clinic’s Lung Cancer Screening Rates: A Quality Improvement Project**. *The Journal for Nurse Practitioners* 2020, **16**(10):e169-e172.

6. Cassagnol M, Hai O, Sherali SA, D'Angelo K, Bass D, Zeltser R, Makaryus AN: **Impact of cardiologist intervention on guideline-directed use of statin therapy**. *World J Cardiol* 2020, **12**(8):419-426.

7. Ciprut SE, Kelly MD, Walter D, Hoffman R, Becker DJ, Loeb S, Sedlander E, Tenner CT, Sherman SE, Zeliadt SB *et al*: **A Clinical Reminder Order Check Intervention to Improve Guideline-concordant Imaging Practices for Men With Prostate Cancer: A Pilot Study**. *Urology* 2020, **145**:113-119.

8. Daud MH, Ramli AS, Abdul-Razak S, Haniff J, Tg Abu Bakar Sidik TMI, Mohd Hatta NKB, Mahmood S, Lakshmanan S: **Effectiveness of the EMPOWER-PAR Intervention on Primary Care Providers’ Adherence to Clinical Practice Guideline on the Management of Type 2 Diabetes Mellitus: A Pragmatic Cluster Randomised Controlled Trial**. *Open Access Macedonian Journal of Medical Sciences* 2020, **8**(B):470-479.

9. Gupta M, Maamoun W, Maher M, Jaffe W: **Ensuring universal assessment and management of vitamin D status in melanoma patients at secondary care level: a service improvement project**. *Br J Hosp Med (Lond)* 2020, **81**(10):1-5.

10. Holmes CE, Ades S, Gilchrist S, Douce D, Libby K, Rogala B, Parenteau E, Cushman M, Holm AK: **Successful Model for Guideline Implementation to Prevent Cancer-Associated Thrombosis: Venous Thromboembolism Prevention in the Ambulatory Cancer Clinic**. *JCO Oncol Pract* 2020, **16**(9):e868-e874.

11. Levi CR, Attia JA, D'Este C, Ryan AE, Henskens F, Kerr E, Parsons MW, Sanson-Fisher RW, Bladin CF, Lindley RI *et al*: **Cluster-Randomized Trial of Thrombolysis Implementation Support in Metropolitan and Regional Australian Stroke Centers: Lessons for Individual and Systems Behavior Change**. *J Am Heart Assoc* 2020, **9**(3):e012732.

12. Lipscomb J, Escoffery C, Gillespie TW, Henley SJ, Smith RA, Chociemski T, Almon L, Jiang R, Sheng X, Goodman M *et al*: **Improving Screening Uptake among Breast Cancer Survivors and Their First-Degree Relatives at Elevated Risk to Breast Cancer: Results and Implications of a Randomized Study in the State of Georgia**. *Int J Environ Res Public Health* 2020, **17**(3).

13. Marszalek D, Martinson A, Smith A, Marchand W, Sweeney C, Carney J, Lowery T, Clinton-Lont J: **Examining the Effect of a Whole Health Primary Care Pain Education and Opioid Monitoring Program on Implementation of VA/DoD-Recommended Guidelines for Long-term Opioid Therapy in a Primary Care Chronic Pain Population**. *Pain Med* 2020, **21**(10):2146-2153.

14. McGuinness R, Keevil H, Sharif A, Lau TK, Crookes W, Bhamm R, Ali S, Payne V, Hollinshead L, Cundy K *et al*: **Improving the percentage of HIV tests offered to patients admitted to an acute hospital trust with community-acquired pneumonia**. *BMJ Open Qual* 2020, **9**(4).

15. Nguyen N, Nguyen T, Truong V, Dang K, Siman N, Shelley D: **Impact of a tobacco cessation intervention on adherence to tobacco use treatment guidelines among village health workers in Vietnam**. *Glob Health Promot* 2020, **27**(3):24-33.

16. Roberts S, Busby E: **Implementing clinical guidelines into practice: The Osteoarthritis Self-management and Independent-living Support (OASIS) group-A service evaluation**. *Musculoskeletal Care* 2020, **18**(3):404-411.

17. Rust C, Prior RM, Stec M: **Implementation of a clinical practice guideline in a primary care setting for the prevention and management of obesity in adults**. *Nurs Forum* 2020, **55**(3):485-490.

18. Segala FV, Murri R, Taddei E, Giovannenze F, Del Vecchio P, Birocchi E, Taccari F, Cauda R, Fantoni M: **Antibiotic appropriateness and adherence to local guidelines in perioperative prophylaxis: results from an antimicrobial stewardship intervention**. *Antimicrob Resist Infect Control* 2020, **9**(1):164.

19. Silverberg ND, Panenka WJ, Lizotte PP, Bayley MT, Dance D, Li LC: **Promoting early treatment for mild traumatic brain injury in primary care with a guideline implementation tool: a pilot cluster randomised trial**. *BMJ Open* 2020, **10**(10):e035527.

20. Tramontt CR, Jaime PC: **Improving knowledge, self-efficacy and collective efficacy regarding the Brazilian dietary guidelines in primary health care professionals: a community controlled trial**. *BMC Fam Pract* 2020, **21**(1):214.

21. Trogrlic Z, van der Jagt M, van Achterberg T, Ponssen H, Schoonderbeek J, Schreiner F, Verbrugge S, Dijkstra A, Bakker J, Ista E: **Prospective multicentre multifaceted before-after implementation study of ICU delirium guidelines: a process evaluation**. *BMJ Open Qual* 2020, **9**(3).

22. Vani A, Kan K, Iturrate E, Levy-Lambert D, Smilowitz NR, Saxena A, Radford MJ, Gianos E: **Leveraging clinical decision support tools to improve guideline-directed medical therapy in patients with atherosclerotic cardiovascular disease at hospital discharge**. *Cardiol J* 2020.

23. Wu SY, Lazar AA, Gubens MA, Blakely CM, Gottschalk AR, Jablons DM, Jahan TM, Wang VEH, Dunbar TL, Wong ML *et al*: **Evaluation of a National Comprehensive Cancer Network Guidelines-Based Decision Support Tool in Patients With Non-Small Cell Lung Cancer: A Nonrandomized Clinical Trial**. *JAMA Netw Open* 2020, **3**(9):e209750.

24. Zgierska AE, Robinson JM, Lennon RP, Smith PD, Nisbet K, Ales MW, Boss D, Tuan WJ, Vidaver RM, Hahn DL: **Increasing system-wide implementation of opioid prescribing guidelines in primary care: findings from a non-randomized stepped-wedge quality improvement project**. *BMC Fam Pract* 2020, **21**(1):245.

25. Abbood SK, Assad HC, Al-Jumaili AA: **Pharmacist intervention to enhance postoperative fluid prescribing practice in an Iraqi hospital through implementation of NICE guideline**. *Pharm Pract (Granada)* 2019, **17**(3):1552.

26. Bernhardsson S, Larsson MEH: **Does a tailored guideline implementation strategy have an impact on clinical physiotherapy practice? A nonrandomized controlled study**. *J Eval Clin Pract* 2019, **25**(4):575-584.

27. Bosch M, McKenzie JE, Ponsford JL, Turner S, Chau M, Tavender EJ, Knott JC, Gruen RL, Francis JJ, Brennan SE *et al*: **Evaluation of a targeted, theory-informed implementation intervention designed to increase uptake of emergency management recommendations regarding adult patients with mild traumatic brain injury: results of the NET cluster randomised trial**. *Implement Sci* 2019, **14**(1):4.

28. Chen AKD, Duffy EJ, Ritchie SR, Thomas MG: **Diagnostic accuracy and adherence to treatment guidelines in adult inpatients with urinary tract infections in a tertiary hospital**. *Journal of Pharmacy Practice and Research* 2019, **49**(3):246-253.

29. Dhopte P, French SD, Quon JA, Owens H, Bussieres A, Canadian Chiropractic Guideline I: **Guideline implementation in the Canadian chiropractic setting: a pilot cluster randomized controlled trial and parallel study**. *Chiropr Man Therap* 2019, **27**:31.

30. Dreijer AR, Diepstraten J, Leebeek FWG, Kruip M, van den Bemt P: **The effect of hospital-based antithrombotic stewardship on adherence to anticoagulant guidelines**. *Int J Clin Pharm* 2019, **41**(3):691-699.

31. Gulayin PE, Lozada A, Beratarrechea A, Gutierrez L, Poggio R, Chaparro RM, Santero M, Masson W, Rubinstein A, Irazola V: **An Educational Intervention to Improve Statin Use: Cluster RCT at the Primary Care Level in Argentina**. *Am J Prev Med* 2019, **57**(1):95-105.

32. Huang KTL, Blazey-Martin D, Chandler D, Wurcel A, Gillis J, Tishler J: **A multicomponent intervention to improve adherence to opioid prescribing and monitoring guidelines in primary care**. *J Opioid Manag* 2019, **15**(6):445-453.

33. Jolliffe L, Morarty J, Hoffmann T, Crotty M, Hunter P, Cameron ID, Li X, Lannin NA: **Using audit and feedback to increase clinician adherence to clinical practice guidelines in brain injury rehabilitation: A before and after study**. *PLoS One* 2019, **14**(3):e0213525.

34. Lee CK, Lai CL, Lee MH, Su FY, Yeh TS, Cheng LY, Hsieh MY, Wu YW, Liu YB, Wu CC: **Reinforcement of patient education improved physicians' adherence to guideline-recommended medical therapy after acute coronary syndrome**. *PLoS One* 2019, **14**(6):e0217444.

35. Marcial E, Graves BA: **Implementation and Evaluation of Diabetes Clinical Practice Guidelines in a Primary Care Clinic Serving a Hispanic Community**. *Worldviews on Evidence-Based Nursing* 2019, **16**(2):9.

36. McAdam-Marx C, Tak C, Petigara T, Jones NW, Yoo M, Briley MS, Gunning K, Gren L: **Impact of a guideline-based best practice alert on pneumococcal vaccination rates in adults in a primary care setting**. *BMC Health Serv Res* 2019, **19**(1):474.

37. Moseng T, Dagfinrud H, Osteras N: **Implementing international osteoarthritis guidelines in primary care: uptake and fidelity among health professionals and patients**. *Osteoarthritis Cartilage* 2019, **27**(8):1138-1147.

38. Orchard J, Neubeck L, Freedman B, Li J, Webster R, Zwar N, Gallagher R, Ferguson C, Lowres N: **eHealth Tools to Provide Structured Assistance for Atrial Fibrillation Screening, Management, and Guideline-Recommended Therapy in Metropolitan General Practice: The AF - SMART Study**. *J Am Heart Assoc* 2019, **8**(1):e010959.

39. O'Sullivan CT, Rogers WK, Ackman M, Goto M, Hoff BM: **Implementation of a multifaceted program to sustainably improve appropriate intraoperative antibiotic redosing**. *Am J Infect Control* 2019, **47**(1):74-77.

40. Takaesu Y, Watanabe K, Numata S, Iwata M, Kudo N, Oishi S, Takizawa T, Nemoto K, Yasuda Y, Tagata H *et al*: **Improvement of psychiatrists’ clinical knowledge of the treatment guidelines for schizophrenia and major depressive disorders using the ‘Effectiveness of Guidelines for Dissemination and Education in Psychiatric Treatment (EGUIDE)’ project: A nationwide dissemination, education, and evaluation study**. *Psychiatry and Clinical Neurosciences* 2019, **73**:7.

41. Wilkins B, Hullikunte S, Simmonds M, Sasse A, Larsen P, Harding SA: **Improving the Prescribing Gap For Guideline Recommended Medications Post Myocardial Infarction**. *Heart Lung Circ* 2019, **28**(2):257-262.

42. Carter BL, Levy B, Gryzlak B, Xu Y, Chrischilles E, Dawson J, Vander Weg M, Christensen A, James P, Polgreen L: **Cluster-Randomized Trial to Evaluate a Centralized Clinical Pharmacy Service in Private Family Medicine Offices**. *Circ Cardiovasc Qual Outcomes* 2018, **11**(6):e004188.

43. Dodek P, McKeown S, Young E, Dhingra V: **Development of a Provincial initiative to improve glucose control in critically ill patients**. *Int J Qual Health Care* 2019, **31**(1):49-56.

44. Dziedzic KS, Healey EL, Porcheret M, Afolabi EK, Lewis M, Morden A, Jinks C, McHugh GA, Ryan S, Finney A *et al*: **Implementing core NICE guidelines for osteoarthritis in primary care with a model consultation (MOSAICS): a cluster randomised controlled trial**. *Osteoarthritis Cartilage* 2018, **26**(1):43-53.

45. Etxeberria A, Alcorta I, Perez I, Emparanza JI, Ruiz de Velasco E, Iglesias MT, Rotaeche R: **Results from the CLUES study: a cluster randomized trial for the evaluation of cardiovascular guideline implementation in primary care in Spain**. *BMC Health Serv Res* 2018, **18**(1):93.

46. Karlsson LO, Nilsson S, Bang M, Nilsson L, Charitakis E, Janzon M: **A clinical decision support tool for improving adherence to guidelines on anticoagulant therapy in patients with atrial fibrillation at risk of stroke: A cluster-randomized trial in a Swedish primary care setting (the CDS-AF study)**. *PLoS Med* 2018, **15**(3):e1002528.

47. Knappe S, Einsle F, Rummel-Kluge C, Heinz I, Wieder G, Venz J, Schouler-Ocak M, Wittchen H-U, Lieb R, Hoyer J *et al*: **Niederschwellige leitlinienorientierte supportive Materialien (NILS) in der primärärztlichen Versorgung**. *Zeitschrift für Psychosomatische Medizin und Psychotherapie* 2018, **64**(3):14.

48. Luitjes SHE, Hermens R, de Wit L, Heymans MW, van Tulder MW, Wouters M: **An innovative implementation strategy to improve the use of Dutch guidelines on hypertensive disorders in pregnancy: A randomized controlled trial**. *Pregnancy Hypertens* 2018, **14**:131-138.

49. Mellin C, Lexa M, Bryant AL, Mason S, Mayer DK: **Antiemetic Guidelines: Using education to improve adherence and reduce incidence of CINV in patients receiving highly emetogenic chemotherapy.** *Clinical Journal of Oncology Nursing* 2018, **22**(3):7.

50. Pauwels PPYT, Metsemakers JFM, Himawan AB, Kristina TN: **The Efficacy of Education with the WHO Dengue Algorithm on Correct Diagnosing and Triaging of Dengue-Suspected Patients; Study in Public Health Centre**. *Hiroshima J Med Sci* 2018, **67**:6.

51. Pinto D, Heleno B, Rodrigues DS, Papoila AL, Santos I, Caetano PA: **Effectiveness of educational outreach visits compared with usual guideline dissemination to improve family physician prescribing-an 18-month open cluster-randomized trial**. *Implement Sci* 2018, **13**(1):120.

52. Presseau J, Mackintosh J, Hawthorne G, Francis JJ, Johnston M, Grimshaw JM, Steen N, Coulthard T, Brown H, Kaner E *et al*: **Cluster randomised controlled trial of a theory-based multiple behaviour change intervention aimed at healthcare professionals to improve their management of type 2 diabetes in primary care**. *Implement Sci* 2018, **13**(1):65.

53. Quanbeck A, Brown RT, Zgierska AE, Jacobson N, Robinson JM, Johnson RA, Deyo BM, Madden L, Tuan WJ, Alagoz E: **A randomized matched-pairs study of feasibility, acceptability, and effectiveness of systems consultation: a novel implementation strategy for adopting clinical guidelines for Opioid prescribing in primary care**. *Implement Sci* 2018, **13**(1):21.

54. Ranta A, Dovey S, Gommans J, Tilyard M, Weatherall M: **Impact of General Practitioner Transient Ischemic Attack Training on 90-Day Stroke Outcomes: Secondary Analysis of a Cluster Randomized Controlled Trial**. *J Stroke Cerebrovasc Dis* 2018, **27**(7):2014-2018.

55. Safatly I, Singleton H, Decker K, Roman C, Bystrzycki A, Mitra B: **Emergency management of patients with Supratherapeutic INRs on Warfarin: a multidisciplinary education study**. *The Australian Journal of Advanced Nursing* 2018, **36**(2):8.

56. Suman A, Schaafsma FG, van de Ven PM, Slottje P, Buchbinder R, van Tulder MW, Anema JR: **Effectiveness of a multifaceted implementation strategy compared to usual care on low back pain guideline adherence among general practitioners**. *BMC Health Serv Res* 2018, **18**(1):358.

57. Sun A, Tsoh JY, Tong EK, Cheng J, Chow EA, Stewart SL, Nguyen TT: **A physician-initiated intervention to increase colorectal cancer screening in Chinese patients**. *Cancer* 2018, **124 Suppl 7**:1568-1575.

58. Witt TJ, Deyo-Svendsen ME, Mason ER, Deming JR, Stygar KK, Rosas SL, Phillips MR, Abu Dabrh AM: **A Model for Improving Adherence to Prescribing Guidelines for Chronic Opioid Therapy in Rural Primary Care**. *Mayo Clin Proc Innov Qual Outcomes* 2018, **2**(4):317-323.

59. Al Kalaldeh M: **The influence of implementing nurse-led enteral nutrition guidelines on care delivery in the critically ill: a cohort study**. *Gastrointestinal Nursing* 2017, **15**(6):9.

60. Aloush SM: **Does educating nurses with ventilator-associated pneumonia prevention guidelines improve their compliance?** *Am J Infect Control* 2017, **45**(9):969-973.

61. Coenen S, Weyts E, Jorissen C, De Munter P, Noman M, Ballet V, Vermeire S, Van Assche G, Ferrante M: **Effects of Education and Information on Vaccination Behavior in Patients with Inflammatory Bowel Disease**. *Inflamm Bowel Dis* 2017, **23**(2):318-324.

62. Cummings MJ, Goldberg E, Mwaka S, Kabajaasi O, Vittinghoff E, Cattamanchi A, Katamba A, Kenya-Mugisha N, Jacob ST, Davis JL: **A complex intervention to improve implementation of World Health Organization guidelines for diagnosis of severe illness in low-income settings: a quasi-experimental study from Uganda**. *Implement Sci* 2017, **12**(1):126.

63. Eccleston D, Horrigan M, Rafter T, Holt G, Worthley SG, Sage P, Whelan A, Reid C, Thompson PL: **Improving Guideline Compliance in Australia With a National Percutaneous Coronary Intervention Outcomes Registry**. *Heart Lung Circ* 2017, **26**(12):1303-1309.

64. Jordan KP, Edwards JJ, Porcheret M, Healey EL, Jinks C, Bedson J, Clarkson K, Hay EM, Dziedzic KS: **Effect of a model consultation informed by guidelines on recorded quality of care of osteoarthritis (MOSAICS): a cluster randomised controlled trial in primary care**. *Osteoarthritis Cartilage* 2017, **25**(10):1588-1597.

65. Kersten FAM, Nelen W, van den Boogaard NM, van Rumste MM, Koks CA, IntHout J, Verhoeve HR, Pelinck MJ, Boks DES, Gianotten J *et al*: **Implementing targeted expectant management in fertility care using prognostic modelling: a cluster randomized trial with a multifaceted strategy**. *Hum Reprod* 2017, **32**(8):1648-1657.

66. Lesuis N, van Vollenhoven RF, Akkermans RP, Verhoef LM, Hulscher ME, den Broeder AA: **Rheumatologists' guideline adherence in rheumatoid arthritis: A randomised controlled study on electronic decision support, education and feedback**. *Clinical and Experimental Rheumatology* 2017, **36**(1):8.

67. Liebschutz JM, Xuan Z, Shanahan CW, LaRochelle M, Keosaian J, Beers D, Guara G, O'Connor K, Alford DP, Parker V *et al*: **Improving Adherence to Long-term Opioid Therapy Guidelines to Reduce Opioid Misuse in Primary Care: A Cluster-Randomized Clinical Trial**. *JAMA Intern Med* 2017, **177**(9):1265-1272.

68. Lilih S, Pereboom M, van der Hoeven RT, Mantel-Teeuwisse AK, Becker ML: **Improving the effectiveness of drug safety alerts to increase adherence to the guideline for gastrointestinal prophylaxis**. *Int J Med Inform* 2017, **97**:139-144.

69. Lin LA, Bohnert ASB, Kerns RD, Clay MA, Ganoczy D, Ilgen MA: **Impact of the Opioid Safety Initiative on opioid-related prescribing in veterans**. *Pain* 2017, **158**(5):833-839.

70. Lowe B, Piontek K, Daubmann A, Harter M, Wegscheider K, Konig HH, Shedden-Mora M: **Effectiveness of a Stepped, Collaborative, and Coordinated Health Care Network for Somatoform Disorders (Sofu-Net): A Controlled Cluster Cohort Study**. *Psychosom Med* 2017, **79**(9):1016-1024.

71. Patil VM, Noronha V, Joshi A, Ramaswamy A, Gupta S, Sahu A, Doshi V, Gupta T, Rath S, Banavali S *et al*: **Adherence to and Implementation of ASCO Antiemetic Guidelines in Routine Practice in a Tertiary Cancer Center in India**. *J Oncol Pract* 2017, **13**(6):e574-e581.

72. Tahvonen P, Oikarinen H, Niinimaki J, Liukkonen E, Mattila S, Tervonen O: **Justification and active guideline implementation for spine radiography referrals in primary care**. *Acta Radiol* 2017, **58**(5):586-592.

73. Trietsch J, van Steenkiste B, Grol R, Winkens B, Ulenkate H, Metsemakers J, van der Weijden T: **Effect of audit and feedback with peer review on general practitioners' prescribing and test ordering performance: a cluster-randomized controlled trial**. *BMC Fam Pract* 2017, **18**(1):53.

74. Tunney Jr RK, Johnson DC, Wang L, Cox ZL: **Impact of Pharmacist Intervention to Increase Compliance With Guideline-Directed Statin Therapy During an Acute Coronary Syndrome Hospitalization**. *Annals of Pharmacotherapy* 2017, **51**(5):7.
[truncated: 11,359 more chars]
